# IPNA clinical practice recommendations for the diagnosis and management of children with steroid-sensitive nephrotic syndrome

**DOI:** 10.1007/s00467-022-05739-3

**Published:** 2022-10-21

**Authors:** Agnes Trautmann, Olivia Boyer, Elisabeth Hodson, Arvind Bagga, Debbie S. Gipson, Susan Samuel, Jack Wetzels, Khalid Alhasan, Sushmita Banerjee, Rajendra Bhimma, Melvin Bonilla-Felix, Francisco Cano, Martin Christian, Deirdre Hahn, Hee Gyung Kang, Koichi Nakanishi, Hesham Safouh, Howard Trachtman, Hong Xu, Wendy Cook, Marina Vivarelli, Dieter Haffner, on behalf of the International Pediatric Nephrology Association, Antonia Bouts, Claire Dossier, Francesco Emma, Markus Kemper, Rezan Topaloglu, Aoife Waters, Lutz Thorsten Weber, Alexandra Zurowska, Keisha L. Gibson, Larry Greenbaum, Susan Massengill, David Selewski, Tarak Srivastava, Chia-shi Wang, Scott Wenderfer, Lilian Johnstone, Nicholas Larkins, William Wong, Agnes A. Alba, T. S. Ha, Masoumeh Mokham, Xuhui Zhong, Riku Hamada, Kazumoto Iijima, Kenji Ishikura, Kandai Nozu, Nilzete Bresolin, Nilka De Jesus Gonzalez, Jaime Restrepo, Ifeoma Anochie, Mignon McCulloch

**Affiliations:** 1 Division of Pediatric Nephrology, Center for Pediatrics and Adolescent Medicine, University of Heidelberg, Heidelberg, Germany; 2 Department of Pediatric Nephrology, Reference Center for Idiopathic Nephrotic Syndrome in Children and Adults, Imagine Institute, Paris University, Necker Children’s Hospital, APHP, Paris, France; 3 Cochrane Kidney and Transplant, Centre for Kidney Research, The Children’s Hospital at Westmead, Sydney, Australia; 4 Division of Nephrology, Department of Pediatrics, All India Institute of Medical Sciences, New Delhi, India; 5 Department of Pediatrics, Division of Nephrology, University of Michigan, Ann Arbor, MI USA; 6 Section of Pediatric Nephrology, Department of Pediatrics, Alberta Children’s Hospital Research Institute, University of Calgary, Calgary, Canada; 7 Department of Nephrology, Radboud University Medical Center, Nijmegen, The Netherlands; 8 Pediatric Department, College of Medicine, King Saud University, Riyadh, Saudi Arabia; 9 Department of Pediatric Nephrology, Institute of Child Health, Kolkata, India; 10 University of KwaZulu-Natal, Durban, South Africa; 11 Department of Pediatrics, University of Puerto Rico-Medical Sciences Campus, San Juan, Puerto Rico; 12 Department of Pediatric Nephrology, Luis Calvo Mackenna Children’s Hospital, University of Chile, Santiago, Chile; 13 Children’s Kidney Unit, Nottingham Children’s Hospital, Nottingham, UK; 14 Division of Pediatric Nephrology, Department of Paediatrics, The Children’s Hospital at Westmead, Sydney, Australia; 15 Division of Pediatric Nephrology, Department of Pediatrics, Seoul National University Children’s Hospital & Seoul National University College of Medicine, Seoul, Korea; 16 Department of Child Health and Welfare (Pediatrics), Graduate School of Medicine, University of the Ryukyus, Okinawa, Japan; 17 Pediatric Nephrology Unit, Faculty of Medicine, Cairo University, Cairo, Egypt; 18 Department of Pediatrics, Division of Nephrology, University of Michigan, Ann Arbor, MI USA; 19 Department of Nephrology, Children’s Hospital of Fudan University, Shanghai, China; 20 Nephrotic Syndrome Trust (NeST), Somerset, UK; 21 Division of Nephrology and Dialysis, Department of Pediatric Subspecialties, Bambino Gesù Pediatric Hospital IRCCS, Rome, Italy; 22 Department of Pediatric Kidney, Liver and Metabolic Diseases, Hannover Medical School Children’s Hospital, Hannover and Center for Rare Diseases, Hannover Medical School, Hannover, Germany

**Keywords:** Steroid-sensitive nephrotic syndrome, SSNS, Children, Frequently relapsing nephrotic syndrome, Steroid-dependent nephrotic syndrome, Steroid toxicity, Pediatrics, Immunosuppressive treatment

## Abstract

Idiopathic nephrotic syndrome is the most frequent pediatric glomerular disease, affecting from 1.15 to 16.9 per 100,000 children per year globally. It is characterized by massive proteinuria, hypoalbuminemia, and/or concomitant edema. Approximately 85–90% of patients attain complete remission of proteinuria within 4–6 weeks of treatment with glucocorticoids, and therefore, have steroid-sensitive nephrotic syndrome (SSNS). Among those patients who are steroid sensitive, 70–80% will have at least one relapse during follow-up, and up to 50% of these patients will experience frequent relapses or become dependent on glucocorticoids to maintain remission. The dose and duration of steroid treatment to prolong time between relapses remains a subject of much debate, and patients continue to experience a high prevalence of steroid-related morbidity. Various steroid-sparing immunosuppressive drugs have been used in clinical practice; however, there is marked practice variation in the selection of these drugs and timing of their introduction during the course of the disease. Therefore, international evidence-based clinical practice recommendations (CPRs) are needed to guide clinical practice and reduce practice variation. The International Pediatric Nephrology Association (IPNA) convened a team of experts including pediatric nephrologists, an adult nephrologist, and a patient representative to develop comprehensive CPRs on the diagnosis and management of SSNS in children. After performing a systematic literature review on 12 clinically relevant PICO (Patient or Population covered, Intervention, Comparator, Outcome) questions, recommendations were formulated and formally graded at several virtual consensus meetings. New definitions for treatment outcomes to help guide change of therapy and recommendations for important research questions are given.

**Supplementary Information:**

The online version contains supplementary material available at 10.1007/s00467-022-05739-3.

## Introduction

Idiopathic nephrotic syndrome (INS), characterized by massive proteinuria, hypoalbuminemia, and/or concomitant edema is the most frequent glomerular disease in children. Its incidence ranges from 1.15 to 16.9 per 100,000 children and varies by ethnicity and region [1, 2]. Until the discovery of glucocorticoids as an effective treatment to induce remission in the 1950s, childhood nephrotic syndrome (NS) was associated with a high mortality (ca. 40%) due to acute kidney injury (AKI), chronic kidney disease (CKD), systemic infections, and thromboembolic events. The majority of affected children (ca. 85%) show complete remission of proteinuria within 4–6 weeks with daily prednisolone/prednisone (PDN) and have steroid-sensitive NS (SSNS). However, about 70–80% of patients will experience at least one relapse during follow-up. About 50% of patients have frequent relapses or are steroid-dependent [1, 3–5]. Childhood onset SSNS may resolve spontaneously following puberty; however, 10–30% continue to have a relapsing course into young adulthood [6–8]. Kidney biopsies are not routinely performed in children with SSNS because they have limited prognostic or clinical utility. If a biopsy is done the most common diagnoses are minimal change disease (MCD) showing either minimal changes, i.e., podocyte foot process effacement, or mild mesangial proliferation with IgM deposition, or less commonly focal-segmental glomerulosclerosis (FSGS) [9].

Management of relapsing SSNS is a great challenge. Long or frequent use of high-dose steroids is associated with steroid toxicity and reduction in quality of life (QOL) [10]. Several steroid-sparing agents are available but they can be associated with significant adverse effects [11–14]. The long-term goal of treatment of NS is to achieve freedom from recurrence, minimize side effects and improve QOL.

There are no international, evidence-based, systematically developed recommendations for the diagnosis and management of children with SSNS with the exception of a focused document from KDIGO [15]. Therefore, the International Pediatric Nephrology Association (IPNA) convened a clinical practice recommendation (CPR) workgroup in October 2019 to develop CPRs for the diagnosis and management of children with SSNS. This guideline provides evidence-based recommendations as well as a pragmatic approach to the management of SSNS. New definitions differing from previous ones, e.g., from KDIGO, for treatment outcomes are provided to help guide change of therapy in order to minimize the frequency of relapses and drug toxicity. Recommendations for future research to improve outcomes on children with INS are also presented.

## Methods

### Overview of the guideline project

We followed the RIGHT (Reporting Items for practice Guidelines in HealThcare) Statement for Practice Guidelines [16]. Three groups were assembled: a core leadership group, an external expert group, and a voting panel. The core group comprised 16 members of IPNA, including pediatric nephrologists and epidemiologists, an adult nephrologist, and a patient representative. The individual expertise and responsibilities of the core group members are given in Supplementary Table S1. The external expert group included three patient representatives, a general pediatrician, two pediatric endocrinologists, two experts in transition, and three dieticians. The patient representatives discussed the manuscript provided by the core group members within their local patient and family associations, and their suggestions were then incorporated into the manuscript. The voting panel included 32 pediatric nephrologists including 3–7 representatives of each IPNA Regional Society with expertise in the management of SSNS in children. Voting group members were asked by electronic questionnaire to provide a level of agreement on a 5-point scale (strongly disagree, disagree, neither agree/disagree, agree, strongly agree) (Delphi method). For topics that failed to achieve a 70% level of consensus, the recommendations were re-evaluated and modified by the core group and then reviewed again by the voting panel until a consensus level of > 70% was achieved.

### Developing the PICO questions

We developed PICO (Patient or Population covered, Intervention, Comparator, Outcome) questions as follows [17]: *Population:* Children (> 3 months and < 18 years) with SSNS; *Intervention and Comparators:* Treatment compared with no treatment, other treatment or placebo; *Outcomes Addressed:* Recommendations for the treatment, and follow-up of children with SSNS (including efficacy to induce remission and side effects of medications). Definitions of nephrotic syndrome were reviewed and new definitions of treatment outcomes were developed.

### Literature search

The PubMed database was searched for studies published by January 11, 2022; all systematic reviews of randomized controlled trials (RCTs) on the treatment of SSNS in children, prospective uncontrolled trials, observational studies, and registry studies on diagnosis and treatment of children with SSNS, restricted to human studies in English were retrieved. Where possible, risk ratios (RR) with 95% confidence intervals (CI) were cited from two Cochrane systematic reviews evaluating RCTs of interventions for childhood SSNS updated in 2020 [10, 12]. Further details and a summary of the publications used for this CPR are given in the supplementary material (Supplementary Tables S2-S10).

### Grading system

**Fig. 1:**
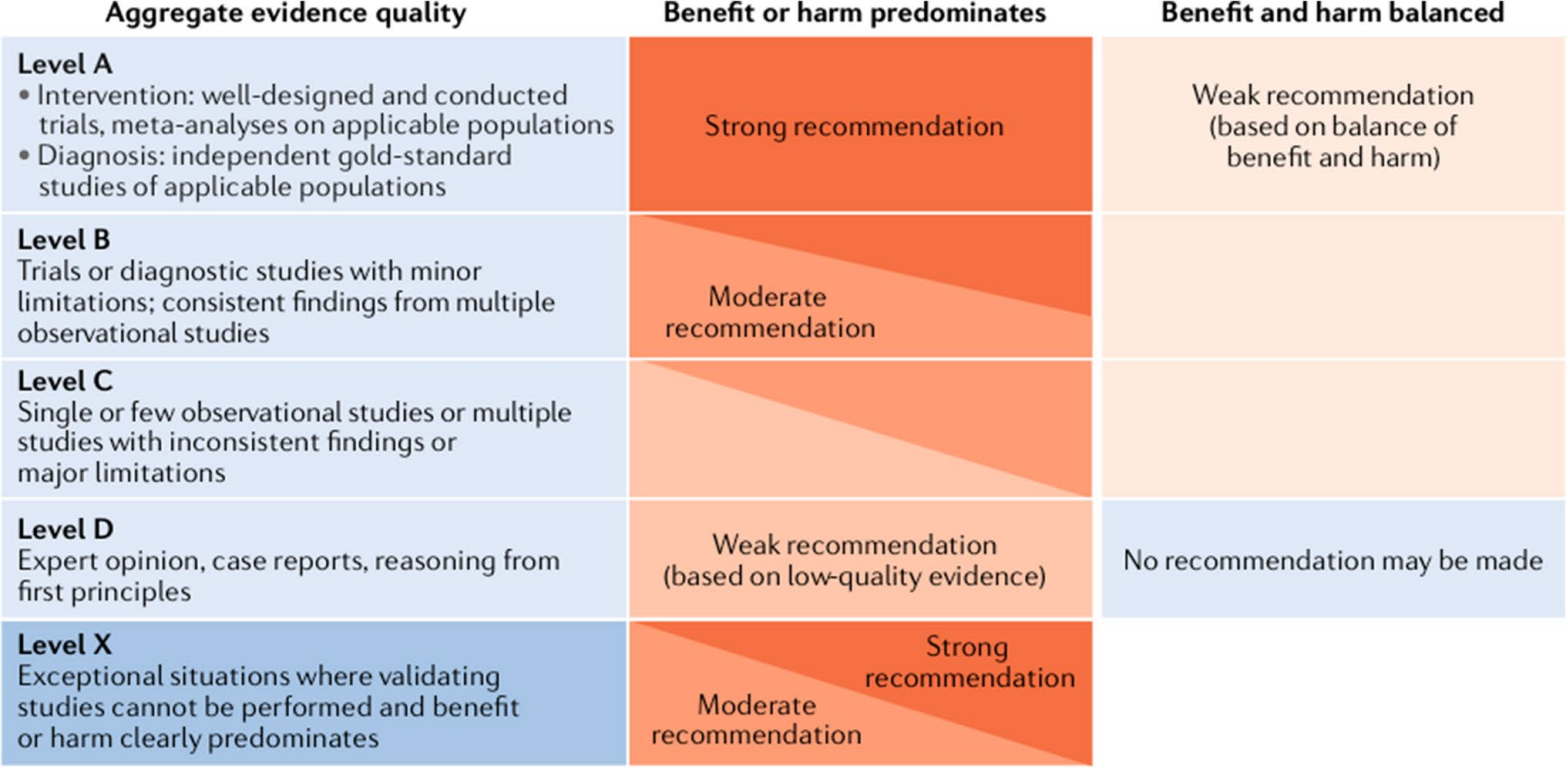
Matrix for grading of evidence and assigning strength of recommendations as currently used by the American Academy of Pediatrics. Reproduced with permission from [23]

We followed the grading system of the American Academy of Pediatrics [18] (Fig. 1). The quality of evidence was graded as High (A), Moderate (B), Low (C), Very low (D), or Not applicable (X). The latter refers to exceptional situations where validating studies cannot be performed because benefit or harm clearly predominates. The letter X was used to grade contra-indications of therapeutic measures and safety parameters. The strength of a recommendation was graded as strong, moderate, weak, or discretionary (when no recommendation can be made).

## Clinical practice recommendations

## Definitions and diagnostic work-up

### Definitions

- We recommend using the definitions given in Table 1 for the diagnosis and management of children with SSNS (grade X, moderate recommendation).

**Table 1:** Definitions

Term	Definition
Nephrotic-range proteinuria^a^	Urinary protein creatinine ratio (UPCR) ≥ 200 mg/mmol (2 mg/mg) in a spot urine, or proteinuria ≥ 1000 mg/m^2^ per day in a 24-h urine sample corresponding to 3 + (300–1000 mg/dL) or 4 + (≥ 1000 mg/dL) by urine dipstick
Nephrotic syndrome	Nephrotic-range proteinuria and either hypoalbuminemia (serum albumin < 30 g/L) or edema when serum albumin is not available
Complete remission	UPCR (based on first morning void or 24 h urine sample) ≤ 20 mg/mmol (0.2 mg/mg) or < 100 mg/m^2^ per day, respectively, or negative or trace dipstick on three or more consecutive days
Partial remission	UPCR (based on first morning void or 24 h urine sample) > 20 but < 200 mg/mmol (> 0.2 mg/mg but < 2 mg/mg) and serum albumin ≥ 30 g/L
Steroid-sensitive nephrotic syndrome (SSNS)	Complete remission within 4 weeks of PDN at standard dose (60 mg/m^2^/day or 2 mg/kg/day, maximum 60 mg/day)
Steroid-resistant nephrotic syndrome (SRNS)	Lack of complete remission within 4 weeks of treatment with PDN at standard dose
Confirmation period	Time period between 4 and 6 weeks from PDN initiation during which responses to further oral PDN and/or pulses of IV MPDN and RAASi are ascertained in patients achieving only partial remission at 4 weeks. A patient not achieving complete remission by 6 weeks, although partial remission was achieved at 4 weeks, is defined as SRNS
SSNS late responder	A patient achieving complete remission during the confirmation period (i.e. between 4 and 6 weeks of PDN therapy) for new onset NS
Relapse	Urine dipstick ≥ 3 + (≥ 300 mg/dl) or UPCR ≥ 200 mg/mmol (≥ 2 mg/mg) on a spot urine sample on 3 consecutive days, with or without reappearance of edema in a child who had previously achieved complete remission
Infrequently relapsing nephrotic syndrome	< 2 relapses in the 6 months following remission of the initial episode or fewer than 3 relapses in any subsequent 12-month period
Frequently relapsing nephrotic syndrome (FRNS)	≥ 2 relapses in the first 6-months following remission of the initial episode or ≥ 3 relapses in any 12 months
Steroid-dependent nephrotic syndrome (SDNS)	A patient with SSNS who experiences 2 consecutive relapses during recommended PDN therapy for first presentation or relapse or within 14 days of its discontinuation
Steroid toxicity	New or worsening obesity/overweight, sustained hypertension, hyperglycemia
	Behavioral/psychiatric disorders, sleep disruption
	Impaired statural growth (height velocity < 25th percentile and/or height < 3rd percentile) in a child with normal growth before start of steroid treatment
	Cushingoid features, striae rubrae/distensae, glaucoma, ocular cataract, bone pain, avascular necrosis
Sustained remission	No relapses over 12 months with or without therapy
SSNS controlled on therapy	Infrequently relapsing NS or sustained remission while on immunosuppression in the absence of significant drug-related toxicity
SSNS not controlled on therapy	Either frequently relapsing NS despite immunosuppression or significant drug-related toxicity while on immunosuppression
Secondary steroid resistance	SSNS patient who at a subsequent relapse does not achieve complete remission within 4 weeks of PDN at standard dose
Complicated relapse	A relapse requiring hospitalization due to one or more of the following: severe edema, symptomatic hypovolemia or AKI requiring IV albumin infusions, thrombosis, or severe infections (e.g., sepsis, peritonitis, pneumonia, cellulitis)

^a^In adults, nephrotic range proteinuria is defined by proteinuria > 3.5 g/24 h (or > 3000 mg/g or > 3 g/10 mmol creatinine) [15]. These cut-offs should also apply to adolescents (> 16 years)

#### Evidence and rationale

The definitions presented in this CPR agree with previously published IPNA Clinical Practice Recommendations for the diagnosis and management of children with steroid-resistant nephrotic syndrome (SRNS) [19] and the KDIGO 2021 Guideline for the Management of Glomerular Diseases [15, 20]. In addition, new definitions for treatment outcomes to help guide change of therapy, e.g., the introduction of steroid-sparing agents, are provided. Of note, patients with late response, i.e., remission between 4 and 6 weeks of PDN therapy, are defined as “SSNS late responder” and should be managed as SSNS but anticipating a potentially more severe course.

The proposed definition of frequently relapsing nephrotic syndrome (FRNS) differs from previous ones including those from KDIGO. The prescription for the first episode of SSNS usually amounts to a PDN exposure of ~ 115 mg/kg. Each relapse adds ~ 40–45 mg/kg; three relapses would mean 120–130 mg/kg, and four relapses would mean 160 mg/kg over 12 months. A child with 4 relapses in a year would thus be exposed to ~ 0.5 mg/kg/day PDN, which may not be acceptable in terms of toxicity risk. Therefore, we propose to revise the definition of FRNS to include children with *2 or more relapses in the first 6 months of the disease*, *or 3 or more relapses in any 12*-*month period*. The definition of FRNS as a disease classification serves as a clinical indicator that treatment strategies should be transitioned from responsive, ad hoc therapy to preventive or proactive therapy to reduce relapses and corticosteroid toxicity. Considering the spectrum of steroid-associated adverse effects, the anxiety that the fear of relapses causes in patients and families and the patient/family preferences for steroid minimization, the rationale for this change is two-fold. First, the new definition of FRNS promotes a discussion and selection of therapy for patients with FRNS, which incorporates patient/family preferences. Second, the new definition acknowledges the fact that many pediatric nephrology centers throughout the globe already implement this threshold in routine clinical practice to optimize steroid minimization.

Regarding steroid-dependent nephrotic syndrome (SDNS), the wording of the definition has been fine-tuned. The term “recommended PDN” has been added to promote a uniform steroid treatment in all children with NS both in relapse and in remission. Moreover, “PDN for first presentation or relapse” aims to clarify that patients relapsing during or 14 days after *low-dose* maintenance treatment with PDN are not steroid-dependent. It is only a relapse during or within 14 days after completing *high-dose* PDN (i.e., 2 mg/kg per day or 1.5 mg/kg on alternate days) discontinuation, that qualifies for this definition.

Regarding the definition of hypoalbuminemia, usually a cut-off of 30 g/L is used. However, there is significant variation between serum albumin assays in different laboratories. The 2021 KDIGO guideline states: “Laboratory-specific values: serum albumin should be measured by bromocresol purple (BCP; colorimetric) capillary electrophoresis (CE), or immunonephelometric (iMN) methods. Bromocresol green (BCG) methods can give erroneously high results” [20]. The values of serum albumin measured by BCG are about 5.5 g/L higher than those measured by the BCP, CE, or iMN methods [21], so the definition of the degree of hypoalbuminemia required to meet a definition of NS varies according to the method used for quantifying serum albumin concentration. The bias between different albumin assays may affect clinical decision-making [22]. However, as long as a specific method is used consistently based on local laboratory practice, changes in serial albumin concentration can be monitored over time.

Regarding statural growth, we suggest using the definition for impaired statural growth as recommended for children with CKD, i.e., a height velocity < 25th percentile and/or height < 3rd percentile [23]. Height velocity should be calculated based on an observation period of at least 6 months. We also suggest using the body mass index (BMI) cut off values for age and sex to define overweight (25–30 kg/m^2^) or obese (≥ 30 kg/m^2^) as recommended by the International Obesity Taskforce [24]. For all anthropometric analysis, national reference values should be applied, or if not available the World Health Organization (WHO) standards should be applied (https://www.who.int/tools/child-growth-standards/standards).

### Clinical assessment

- We recommend a work-up for the diagnosis of nephrotic syndrome (NS) in all children with gravity-dependent edema (grade A, strong recommendation).
- We recommend using spot urine samples, preferably a first morning void, or alternatively a 24-h urine sample to assess proteinuria (grade B, moderate recommendation).
- We recommend confirming nephrotic range proteinuria at least once by quantification of proteinuria before initiating treatment for the first episode (grade B, moderate recommendation).

#### Evidence and rationale

Periorbital edema is the leading clinical sign of NS in children with a typical presentation. It may be asymmetrical initially and is frequently misdiagnosed as allergy. Edema is gravity-dependent, localized to the lower extremities in the upright position, and to the eyelids and the dorsal part of the body in a reclining position. The edema is painless, soft and pitting, keeping the marks of clothes or finger pressure. Anasarca may develop with ascites, and pleural and pericardial effusions. Efforts are underway to standardize the assessment of edema. Complications of NS may be the presenting symptoms or signs of the disease (e.g., abdominal pain related to severe hypovolemia, ascites, peritonitis, or pneumonia, dyspnea as a consequence of pleural effusion, ascites, pneumonia, or pulmonary embolism).

Extrarenal causes of edema should be considered including hepatic (hepatocellular insufficiency, cirrhosis, Budd-Chiari syndrome), digestive (exudative enteropathy, coeliac disease, lymphangiectasis), severe malnutrition, heart failure, hereditary angioneurotic edema, capillary leak syndrome, and thyroid abnormalities.

The diagnostic laboratory finding in children with NS is nephrotic range proteinuria (Table 1) defined by 3 + on urine dipstick in a spot urine, a urinary protein creatinine ratio (UPCR) ≥ 200 mg/mmol (≥ 2 mg/mg) or proteinuria > 40 mg/m^2^/h or ≥ 1000 mg/m^2^/day in a 24-h urine collection (Table 1). The use of a spot urine may be preferred to avoid sampling error and because of its excellent correlation with 24-h urine protein excretion [25]. Although urinary dipstick analysis is useful for screening and home monitoring, we recommend confirming nephrotic range proteinuria at least once by quantification of proteinuria either by spot urine sampling (if possible, first-morning void) or on a 24-h sample before initiating treatment for the first episode. First morning urine samples help rule out orthostatic proteinuria during follow-up to diagnose relapses [25, 26]. Typical semiquantitative dipstick results are shown in Supplementary Table S11. UPCr is preferentially used in SSNS as the urinary albumin creatinine ratio, although more specific, is less relevant in nephrotic range proteinuria. In addition, there are no universally accepted definitions for nephrotic range proteinuria when using urinary albumin creatinine ratio.

### Initial diagnostic work-up

**Fig. 2:**
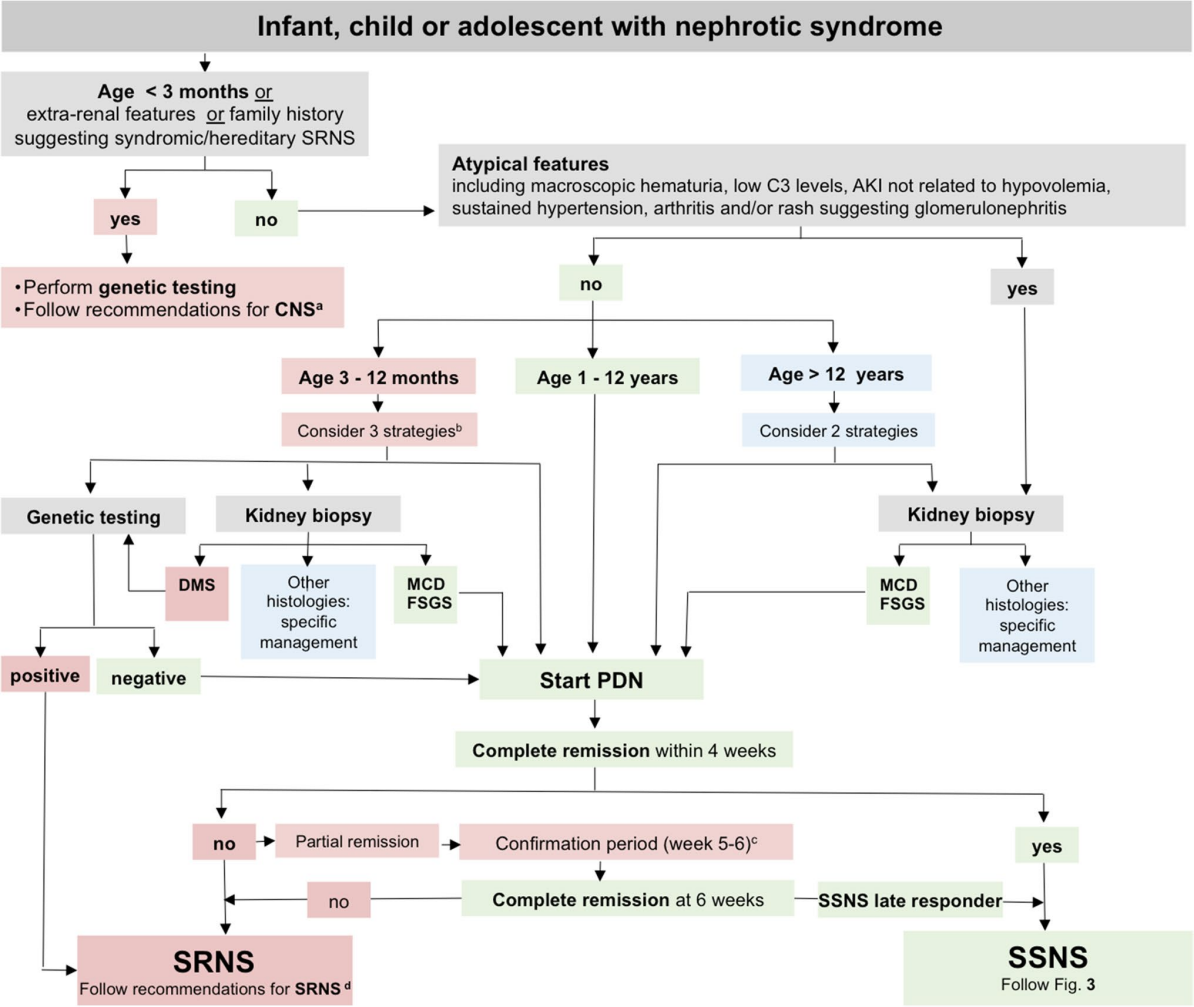
Algorithm for the initial management of a child with nephrotic syndrome. Patients are managed according to age, clinical presentation, and response to a 4-week treatment with oral prednisolone/prednisone (PDN). ^a^In children with congenital NS, we recommend following the published guideline for CNS [27]. ^b^In children between 3 and 12 months of age at onset (infantile NS), there is no evidence-based clear-cut approach to management. We suggest following one of the following three options in children without extrarenal manifestations: (i) primary genetic testing, if the results are rapidly available, with standard PDN treatment given if genetic testing is negative; (ii) primary kidney biopsy, followed by standard PDN treatment in the case of MCD and FSGS, genetic testing in the case of DMS, and specific treatment in the case of other underlying kidney histopathologies; (iii) starting standard PDN treatment, assessing at 4 weeks and then initiating genetic testing and kidney biopsy in case of SRNS. Patients > 1 year of age at onset are characterized according to response to a 4-week-treatment with oral prednisolone (PDN). We suggest that the decision of performing a kidney biopsy in older children (> 12 years) be made on a case-by-case basis. ^c^Patients showing incomplete remission at 4 weeks enter the confirmation period in which responses to further oral prednisolone (PDN) with or without methylprednisolone (MPDN) pulses in conjunction with either angiotensin-converting enzyme inhibitors (ACEi) or angiotensin-receptor blockers (ARBs) are ascertained, and genetic and histopathological evaluation is initiated [19]. ^d^In children with SRNS, we recommend following the published recommendations for SRNS [19]. Further details are given in Table 2 and in the text. *NS* nephrotic syndrome, *AKI* acute kidney injury, *CNS* congenital NS, *SSNS* steroid-sensitive NS, *SRNS* steroid-resistant NS, *MCD* minimal change disease, *FSGS* focal segmental glomerulosclerosis, *DMS* diffuse mesangial sclerosis

- We recommend that children presenting with NS undergo a diagnostic work-up as outlined in Fig. 2 and Table 2 (grades are given in the table).
- We do not recommend routine kidney biopsy and genetic testing in the initial diagnostic work-up of children with NS who present with typical features and age > 1 year (grade B, moderate recommendation).
- We recommend considering genetic testing and/or kidney biopsy in infantile onset NS (age 3–12 months) (grade B, weak recommendation).

**Table 2:** Initial work-up for a child with nephrotic syndrome

Investigations	Comments
**Clinical evaluation**	
*Relevant patient history*	
Presence of gravity-dependent edema	(grade A, strong recommendation)
Fever episodes, pain, abdominal discomfort, fatigue	
Search for risk factors for secondary causes (e.g., sickle cell disease, HIV, systemic lupus erythematosus, hepatitis B, malaria, parvovirus B19, medications) Screen for tuberculosis	Consider especially in patients from endemic areas before starting immunosuppressant medications (grade C, weak recommendation)
*Physical examination*	
Blood pressure, assess volume status and extent of edema (ascites, pericardial and pleural effusions), lymphadenopathy Signs of infection (respiratory tract, skin, peritonitis, urinary tract)	(grade A, strong recommendation)
Extrarenal features, e.g., dysmorphic features or ambiguous genitalia or eye abnormalities (microcoria, aniridia), rash, arthritis	Further work-up is recommended (grade A, strong recommendation)
*Anthropometry*	
Growth chart: height/length, weight, and head circumference (< 2 years)	We recommend comparing data with appropriate national standards or WHO-MGRS charts (grade A, strong recommendation)
*Vaccination status*	
Check/complete according to national standards esp., for encapsulated bacteria: pneumococcal, meningococcal, *Haemophilus influenzae*, Hep B, SARS-CoV2, influenza vaccine, and varicella	This is recommended before starting immunosuppressant medications other than PDN (grade B, moderate recommendation)
*Family history*	
Kidney disease in family members Extrarenal manifestations HIV or tuberculosis in endemic regions Consanguinity	(grade A, strong recommendation)
**Biochemistry**	
*Spot urine*	
Protein/creatinine ratio (in first morning void)	Recommended at least once before starting treatment of the first episode (grade B, moderate recommendation)
Urinalysis: including hematuria	
*Blood*	
Complete blood count, creatinine, eGFR, urea, electrolytes, albumin	eGFR (mL/min/1.73 m^2^) = k height (cm)/serum creatinine (mg/dl), where k is a constant = 0.413 or eGFR (mL/min/1.73 m^2^) = k height (cm)/serum creatinine (µmol/l), where k is a constant = 36.5 [300, 301]
Complement C3, C4, antinuclear and anti-streptococcal antibodies, and ANCA	Recommended in patients with macroscopic hematuria (grade A, strong recommendation)
Varicella and MMR specific IgG, in non-immunized children	Consider before start of PDN treatment (grade D, weak recommendation)
**Imaging**	
Kidney ultrasound	Consider a kidney ultrasound in all children with INS to exclude kidney malformations and venous thrombosis and in patients with reduced eGFR, hematuria or abdominal pain and always before kidney biopsy (grade D, weak recommendation)
Chest X-ray	Recommended in case of suspected lymphoma (grade D, weak recommendation)
**Histopathology**	
Kidney biopsy	Recommended in patients with atypical features including macroscopic hematuria, low C3 levels, AKI not related to hypovolemia, sustained hypertension, arthritis and/or rash (grade A, strong recommendation)
	Consider in patients with infantile onset NS if genetic screening is not available (age 3–12 months) (grade B, weak recommendation) (Fig. 2)
	Consider in patients > 12 years of age on a case-by-case basis (grade C, weak recommendation)
	Consider in patients with persistent microscopic hematuria in specific populations with a high incidence of glomerular diseases such as IgA nephropathy in East Asia (grade C, weak recommendation)
	Recommended in patients diagnosed with SRNS (grade A, strong recommendation)
**Genetic testing**	Recommended in patients with congenital NS, extrarenal features and/or family history suggesting syndromic/hereditary SRNS (grade A, strong recommendation)
	Consider in patients with infantile onset NS (age 3–12 months) (grade C, weak recommendation) (Fig. 2) Recommended in patients diagnosed with SRNS (grade A, strong recommendation)

*AKI* acute kidney injury, *eGFR* estimated glomerular filtration rate, *ANCA* antineutrophil cytoplasmic antibodies

#### Evidence and rationale–Syndromic and familial NS

A physical examination for extrarenal features suggestive of genetic conditions is recommended (Table 2). Patients with extrarenal features suggestive of monogenic SRNS should primarily undergo genetic testing. Diagnostic work-up in patients with congenital NS (age < 3 months) should be done according to recent clinical practice recommendations [27, 28]. After the neonatal period, if family history is positive for SSNS, PDN therapy should be started as per this SSNS guideline. If family history is positive for a monogenic cause of SRNS, we recommend primary genetic testing.

#### Impact of typical presentation and age

In children, NS with onset at age above 1 year and typical presentation is most often SSNS associated with MCD. The likelihood of MCD is highest between ages 2 and 7 and decreases thereafter [9, 29]. Kidney biopsy allows the exclusion of the differential diagnoses (e.g., membranous nephropathy) and the confirmation of a primary podocytopathy (MCD, FSGS, or diffuse mesangial sclerosis (DMS)). Findings of DMS or membranous nephropathy have therapeutic implications as these entities are treated with specific protocols (membranous nephropathy) or may require genetic testing (DMS). Moreover, it allows the detection and grading of tubular atrophy, interstitial fibrosis, and glomerulosclerosis as prognostic markers [9].

However, there is not enough evidence to identify a clear age limit above which the probability is high enough for non-MCD pathology (e.g., membranous nephropathy), and thus the need for a kidney biopsy in children with NS. Therefore, we suggest that the decision of performing a kidney biopsy in older children (> 12 years) be made on a case-by-case basis. Atypical features suggesting the need for a kidney biopsy include macroscopic hematuria, low C3 levels, sustained hypertension, low estimated glomerular filtration rate (eGFR) not related to hypovolemia, arthritis and/or rash, or other extrarenal findings suggesting glomerulonephritis.

We also suggest a kidney biopsy be performed in patients with nephrotic syndrome and persistent microscopic hematuria in populations with a high incidence of glomerular diseases such as IgA nephropathy in East Asia. To reduce unnecessary kidney biopsies, the finding of more than 30 RBCs/HPF of fresh voided urine may be used as a criterion for performing a kidney biopsy in clinical practice [30].

#### Infantile onset NS

About 50% of children with infantile onset NS (age 3–12 months) have a genetic cause of NS which usually does not respond to PDN treatment [31, 32]. The finding of DMS on kidney biopsy is highly suggestive for an underlying genetic defect, i.e., pathogenic variants in *WT1*, *PLCE1*, or *PDSS2* genes [33–36]. Therefore, we suggest following one of three strategies for infantile NS without extrarenal manifestations (Fig. 2): (i) primary genetic testing, if the results are rapidly available, with standard PDN treatment given if genetic testing is negative; (ii) primary kidney biopsy, followed by standard PDN treatment in the case of MCD and FSGS, genetic testing in the case of DMS, and specific treatment in the case of other underlying kidney histopathologies; and (iii) starting standard PDN treatment and then initiating genetic testing and kidney biopsy in case of SRNS.

### Indications for referral to a pediatric nephrologist

- We recommend referral to a pediatric nephrologist in case of:
  - Atypical features not consistent with idiopathic NS
  - Positive family history for NS
  - Congenital or infantile onset NS
  - Age at onset of NS above 12 years
  - Secondary NS
  - SRNS
  - SSNS late responder
  - FRNS or SDNS
  - SSNS patient with drug toxicities or complicated relapses (grade X, moderate recommendation)

#### Evidence and rationale

SSNS follows a chronic course in most children and ideally all children with SSNS should be cared for by or in conjunction with a pediatric nephrologist at the outset. In some countries, the scarcity of pediatric nephrologists or the distance from tertiary referral centres, require general pediatricians to take primary responsibility [37].

## Primary immunosuppressive treatment of idiopathic NS

### Dose, duration, and dosing strategy of PDN in the initial episode of NS

- After completing the initial diagnostic workup of a child presenting with nephrotic syndrome as outlined above, and a decision is made to start PDN, we recommend that infants > 3 months and children or adolescents (1–18 years) with their first episode of idiopathic NS should receive daily PDN for either:
  - 4 weeks at 60 mg/m^2^ or 2 mg/kg (maximum dose 60 mg/day), followed by alternate day PDN at 40 mg/m^2^ or 1.5 mg/kg (maximum dose of 40 mg on alternate days) for 4 weeks, or
  - 6 weeks at 60 mg/m^2^ or 2 mg/kg (maximum dose 60 mg/day), followed by alternate day PDN at 40 mg/m^2^ or 1.5 mg/kg (maximum dose of 40 mg on alternate days) for 6 weeks (grade A, strong recommendation).
- We recommend administering oral PDN as a single morning dose for the treatment of the initial episode and subsequent relapses (grade B, moderate recommendation).
- We do not recommend a tapering schedule during alternate day dosing (grade A, strong recommendation).
- We suggest that PDN dose should be calculated by either weight or body surface area based on the estimated dry weight (grade B, weak recommendation).

#### Evidence and rationale

Glucocorticoids are widely used in the treatment of NS, and their efficacy is well-established in children > 1 year of age with a typical presentation. In children between 3 and 12 months of age at onset, there is no evidence-based clear-cut approach to management. The management approach should consider the availability of time-sensitive genetic testing. In the absence of extrarenal features, priority may be given either to genetic testing, kidney biopsy or starting PDN, and assessing at 4 weeks (vide supra) (Fig. 2).

Because approximately 50% of children will develop FRNS or SDNS, the use of PDN in longer initial courses has been extensively studied for its efficacy to reduce relapses (Supplementary Table S3). Contrary to earlier evidence suggesting a benefit of longer courses of PDN [38], four recently published well-designed RCTs at low risk of bias, which evaluated 775 children, demonstrated that prolongation of PDN therapy beyond 2 or 3 months in the initial episode of SSNS does not reduce the risk of relapse [39–42]. Since there are no adequately powered well-designed RCTs comparing 2 months with 3 months of PDN therapy, we recommend either an 8-week or a 12-week course of treatment of the initial episode of SSNS in line with KDIGO [15, 20] (Supplementary Table S3). The recent PREDNOS 2019 identified no differences in behavioral effects between different treatment durations [42]. Based on the available evidence, we recommend single daily PDN dosing.

Adverse effects of PDN in children with SSNS are common. An analysis of the adverse effects with PDN in 14 RCTs evaluating PDN therapy in the initial episode of SSNS with observation periods of 12–24 months found that hypertension (13%), psychological disorders (21%), cushingoid appearance (41%), and infections (22%) were common regardless of the total PDN induction dose used [10] (Supplementary Table S4). Future research recommendations are given in Supplementary Table S12.

#### Single daily dosing

Two small RCTs [43, 44] and one observational study [45] have demonstrated no differences in efficacy with a lower toxicity profile when PDN is administered as a single morning dose rather than divided doses. The potential benefits of the single-daily dose regimen include better adherence to therapy, lesser risk of hypothalamic–pituitary–adrenal (HPA) axis suppression and sleep disturbances. Dividing the dose has some practical considerations for medication use in children by minimizing the number of pills or volume of the liquid with each dose.

We do not recommend a tapering schedule during alternate day dosing. None of the four RCTs cited above used a tapering schedule of PDN in the experimental arm. Of the 775 children enrolled, there was only one possible case of adrenal suppression and that occurred in the control arm [41]. Treatment regimens in these four RCTs are shown in Table 3.

**Table 3:** PDN treatment regimens in four well-designed RCTs at low risk of bias

		Initial dose and duration	Subsequent dose and duration (tapering)
Teeninga (2013) [40]	Arm 1 (3-month group)	60 mg/m^2^ daily for 6 weeks	40 mg/m^2^ AD for 6 weeks followed by placebo AD for 12 weeks
	Arm 2 (6-month group)	60 followed by *50 mg/m^2^daily for total 6 weeks *Switch to trial medication at remission	40 and 20 mg/m^2^ AD for 4 weeks each followed by 10 mg/m^2^ AD for 10 weeks
Sinha (2015) [39]	Arm 1 (3-month group)	2 mg/kg daily for 6 weeks	1.5 mg/kg AD for 6 weeks followed by placebo AD for 12 weeks
	Arm 2 (6-month group)	2 mg/kg daily for 6 weeks	1.5 mg/kg AD for 6 weeks followed by 1, 0.75, and 0.5 mg/kg AD for 4 weeks each
Yoshikawa (2015) [41]	Arm 1 (2-month group)	60 mg/m^2^ daily for 4 weeks (Max. 80 mg)	40 mg/m^2^ AD for 4 weeks (Max. 50 mg)
	Arm 2 (6-month group)	60 mg/m^2^ daily for 4 weeks (Max. 80 mg)	60, 45, 30, 15, and 7.5 mg/m^2^ AD for 4 weeks each (Max. 80, 60, 40, 20, and 10 mg each)
Webb (2019) [42]	Arm 1 (2-month group)	60 mg/m^2^ daily for 4 weeks (Max. 80 mg)	40 mg/m^2^ AD for 4 weeks (Max. 60 mg)
	Arm 2 (4-month group)	60 mg/m^2^ daily for 4 weeks (Max. 80 mg)	60, 50, 40, 30, 20, and 10 mg/m^2^ AD for 2 weeks each (Max. 80 at start)

*AD* on alternate days

#### Maximum dose of PDN

The traditional dose of PDN for induction of remission during the first episode of NS is 60 mg/m^2^ per day or 2 mg/kg per day. Most country-based or international guidelines [15, 46–48] recommend a maximum dose of 60 mg/day though the German guidelines recommend a maximum dose of 80 mg/day [46, 49]. No studies have formally evaluated the efficacy of doses higher than 60 or 80 mg/day in SSNS.

Although lower doses of PDN are associated with reduced risk of side effects, these doses may not be as effective. A single small RCT (*n* = 60) showed that a lower dose of PDN (40 mg/m^2^/day) during the initial episode of NS was associated with a longer time to remission compared to the standard dose (60 mg/m^2^ per day; 11.4 ± 4.0 vs. 9.6 ± 2.6 days) [50]. At 24 months, the sustained remission rate was lower in boys receiving 40 mg/m^2^ per day but there was no difference in girls [51]. A retrospective cohort of children with SSNS demonstrated that a lower cumulative dose of PDN (< 2500 mg/m^2^) used during the induction therapy for the first episode of NS is associated with shorter time to first relapse, higher rate of relapses and higher use of steroid-sparing agents, compared to higher doses (> 3000 mg/m^2^) [52]. Therefore, we recommend treating the first episode of NS with a dose of 60 mg/m^2^ per day (or 2 mg/kg per day).

#### Dosing by body surface area or weight

Younger children in particular will receive higher mg of PDN (up to 15% [53]) using a body surface area (BSA) compared to weight per kilogram dosing strategy. Limited knowledge exists regarding whether PDN dose should be calculated by weight or BSA. To avoid PDN overdosing in fluid-overloaded children, we suggest calculating the PDN dose based on the estimated dry weight. Two small RCTs [54, 55] with 146 participants compared weight-based dosing with BSA-based dosing in young children (weight < 30 kg, BSA < 1 m^2^) with their initial episode of SSNS and with relapse of SSNS. There were no statistically significant differences for efficacy or steroid toxicity when comparing weight-based versus BSA-based dosing of PDN but follow-up duration was short in both studies. One patient in the BSA group developed hypertensive encephalopathy [55]. Mean cumulative PDN dose was lower with weight-based dosing in both studies [54, 55]. When height is not available, PDN doses which approximate to 60 mg/m^2^ and 40 mg/m^2^ can be estimated from the formulae: 2 × weight + 8 and weight + 11, respectively [56].

### Combined treatment with steroids and a non-steroidal agent for the initial episode of SSNS

- We do not recommend adding other immunomodulatory or immunosuppressive drugs to PDN for the treatment of the initial episode of NS (grade C, weak recommendation).

#### Evidence and rationale

Studies aiming to reduce the number of relapses by adding a non-glucocorticoid immunosuppressive (steroid-sparing) agent to PDN therapy for the initial episode of NS are scarce. Zhang et al. studied the efficacy of adding azithromycin in combination with PDN therapy in children with their first presentation of NS [57]. The median duration before remission was 6 days in the group that received azithromycin in addition to PDN, and 9 days in the PDN alone group (*p* < 0.0001). There were no differences in terms of relapses at 6 months.

An RCT demonstrated that adding 8 weeks of cyclosporine (CsA) to PDN within the first 4 weeks of treatment of the first episode of NS (after establishing remission over 3 days) reduced the risk of first relapse within the first 6 months (RR 0.33, 95% CI 0.13–0.83), but no difference was observed at 12 months (RR 0.72, 95% CI 0.46–1.13) [58]. There are RCTs in progress in children studying the benefits of adding mycophenolate mofetil (MMF) [59] or levamisole (LEV) [60] to PDN during the initial episode of NS, as soon as children have entered remission, but there are no published results to inform the guideline. Moreover, a significant percentage of children with SSNS are infrequent relapsers and will never require a steroid-sparing agent. Therefore, due to the potential unnecessary side effects and to added cost, initial therapy combining steroids and a steroid-sparing agent cannot be currently recommended.

### Type of steroid agent to induce remission/maintaining remission in children with SSNS

- We recommend that prednisone and prednisolone be used interchangeably, and at the same dose, in both the initial presentation and relapse (grade B, moderate recommendation).

#### Evidence and rationale

For the management of childhood NS, both prednisone and prednisolone have been used interchangeably, and at an equivalent dose. Prednisone is a prodrug of prednisolone [61]. The conversion of prednisone to the biologically active prednisolone occurs mainly in the liver. This interconversion is not a limiting factor, even in patients with severely impaired liver function [62, 63]. NS does not influence the conversion of prednisone to prednisolone [64, 65]. Acute NS and the hypoalbuminemic state do not reduce absorption of PDN or the conversion of prednisone to prednisolone [65, 66]. In clinical practice, prednisolone and prednisone are usually given orally. Prednisolone is palatable and is the preferred choice for young children [67, 68].

#### Deflazacort vs. prednisone/prednisolone

Deflazacort is a synthetic glucocorticoid oxazoline derivative of prednisolone. Six milligrams of deflazacort have approximately the same anti-inflammatory potency as 5 mg of prednisolone or prednisone. There was no difference between deflazacort and PDN in the number achieving remission in the first episode of SSNS in two small RCTs [69, 70]. However, fewer children relapsed following deflazacort treatment compared with PDN [69, 71]. There is a report of toxic epidermal necrolysis in two children with NS who received deflazacort [72]. At this time, there are insufficient data to recommend the use of deflazacort rather than PDN in the treatment of NS.

Intravenous methylprednisolone at equivalent doses of oral prednisone (equivalent dose is 5 mg for every 4 mg of IV methylprednisolone) may be used in situations where a patient is unable to tolerate oral medications or if adherence may be a problem. Intravenous therapy should be limited to a short duration with the intent to switch back to oral medication at the earliest opportunity.

## Monitoring during the acute phase and follow-up

- We recommend educating families to monitor urine protein at home to enable early identification of response to PDN and of relapses (grade X, moderate recommendation).
  - We suggest using the heat coagulation or sulfosalicylic acid test as alternative methods for home monitoring if dipstick testing for proteinuria is not available (grade C, weak recommendation).
  - We recommend regular monitoring for patients with NS during the acute phase and during follow up as outlined in Table 4 (grades are given in the table).
  - We recommend considering a kidney biopsy in patients with SSNS during follow-up if the findings may influence therapy or clarify prognosis. This includes patients on prolonged CNI exposure (> 2 years) especially with high doses, and/or with signs of CNI toxicity such as unexplained decrease in eGFR (grade B moderate recommendation).

**Table 4:** Monitoring during the acute phase and follow-up of a child with NS

Investigations	Comments
**Home monitoring**	
Dipstick assessment (preferably in first morning void)	We recommend daily home urine dipstick testing until remission (grade X, moderate recommendation)
	We suggest home urine dipstick testing, at least twice weekly in the first year, individualize thereafter (grade D, weak recommendation)
	We recommend daily testing if 1 + or more or during episodes of fever, infections and/or suspected relapse (edema) (grade X, moderate recommendation)
**Clinical evaluation**	
*Frequency of outpatient visits*	We suggest outpatient visits every 3 months within the first year, individualized thereafter with more frequent visits in cases of relapse (grade D, weak recommendation)
*Patient history*	
Fever episodes, pain, abdominal discomfort, swelling, fatigue, increased appetite, weight gain, sleep disturbances, behavioral changes	Recommended at every visit. Points to infection or drug toxicity (grade A, strong recommendation)
*Physical examination*	
Blood pressure	Recommended at every visit (grade A, strong recommendation)
Assessment of volume status, including edema (ascites, pericardial and pleural effusions)	Recommended at every visit in patients in relapse (grade A, strong recommendation)
Drug toxicity (e.g., striae, Cushingoid features, avascular necrosis, acne, tremor, hirsutism, gum hyperplasia)	Recommended at every visit in patients on medication (grade A, strong recommodation)
Signs of infection (respiratory tract, skin, peritonitis, urinary tract)	Recommended at every visit (grade A, strong recommodation)
Ophthalmological exam (glaucoma, cataract)	Recommended yearly in patients on PDN (grade A, strong recommendation)
*Anthropometry*	
Growth chart: height/length, weight, and head circumference (< 2 years)	Recommended at every visit; data should be compared with appropriate national standards or WHO-MGRS charts (grade A, strong recommodation)
Calculation of BMI and annual height velocity	Recommended in patients who received PDN treatment within the last 12 months (grade A, strong recommendation)
*Vaccination status*	
Check/complete according to national standards esp., for encapsulated bacteria: pneumococcal, meningococcal, *Hemophilus influenzae*, Hep B, SARS-CoV2, influenza, and varicella-zoster	Suggested as appropriate (grade D, weak recommendation)
**Biochemistry**	
*Spot urine*	
Protein/creatinine ratio (preferably in first morning void)	Suggested as appropriate (pos. dipstick) (grade C, weak recommendation)
*Blood*	
Complete blood count, creatinine, eGFR, urea, electrolytes, albumin	Recommended as appropriate in patients on medication or with complicated relapses (grade A, strong recommendation)
MPA, CsA, TAC	We recommend (pharmacokinetic) blood monitoring in patients on medication as given in Table 5 (grade B, moderate recommendation)
25-OH-vitamin D	Annually in patients with SDNS or FRNS (after three months of remission); aiming for levels > 20 ng/mL (> 50 nmol/l) (grade C, weak recommendation)
**Imaging**	
Kidney ultrasound	Recommended before kidney biopsy (grade A, strong recommendation)
**Histopathology**	
Kidney biopsy	We recommend considering a kidney biopsy in patients with SSNS during follow-up if the findings may potentially influence therapy or help assess prognosis (grade X, moderate recommendation)

*MPA* mycophenolate acid, *CsA* cyclosporin A, *TAC* tacrolimus

### Evidence and rationale

Monitoring of disease activity and potential complications is mandatory for adequate management of relapses and prevention of complications including drug toxicity as given in Table 4. Secondary SRNS should lead to further diagnostic work-up as previously recommended [19].

The mainstay of disease surveillance lies with regular home monitoring, usually by urine dipstick. In case of non-availability of dipsticks, the heat coagulation test or semi-quantitative testing with sulfosalicyclic acid may be used for detecting urine protein [73–76]. Details of performing the heat coagulation test  are available in the supplementary material.

The main reason for clinical assessment during follow-up is to evaluate evidence of adverse effects of the disease and/or treatment. Chronic CNI exposure may result in nephrotoxicity, which is associated with dose and duration of CNI use [77]. Therefore, a kidney biopsy may influence therapy in patients with SSNS, i.e. transition to a non-CNI-based treatment regimen. This includes patients on prolonged CNI exposure (> 2 years) especially with high doses, and/or with signs of CNI toxicity such as unexplained decrease in eGFR.

## First line therapy of relapsing SSNS

- We recommend that SSNS relapse be treated with single daily dose of PDN (2 mg/kg per day or 60 mg/m^2^ per day, maximum 60 mg) until complete remission (UPCr ≤ 20 mg/mmol (0.2 mg/mg) or negative or trace dipstick on 3 or more consecutive days) and then decreased to alternate day PDN (1.5 mg/kg per dose or 40 mg/m^2^ per dose, maximum 40 mg) for 4 weeks (grade B, moderate recommendation).
- We do not recommend a tapering schedule during alternate day dosing (grade A, strong recommendation).

### Evidence and rationale

Children with uncomplicated, infrequent relapses are treated with daily PDN, 60 mg/m^2^ until complete remission followed by conversion to a reduced dose (40 mg/m^2^ per dose) on alternate days for 4 weeks [78] (see Supplementary Table S3.3). A single RCT assessed whether reducing the duration of alternate day PDN relapse therapy to 2 weeks after remission is non-inferior to the standard 4-week duration [79]. The time to first relapse, development of FRNS or SDNS, and adverse effects were similar in both groups. Cumulative dose of PDN was lower in the short duration group. Non-inferiority was not proven with this trial. A further RCT evaluated extension of the alternate-day treatment period from 36 to 72 days in children with FRNS/SDNS, with a comparable cumulative PDN dose in both groups [80]. The proportion of children relapsing within 6 months was not different between the study arms (58% long duration vs. 42% short duration, *p* = 0.26). A further study comparing a 2-week and 6-week period of alternate-day PDN with different cumulative PDN doses is ongoing [81] (Supplementary Table S3.3). As presented in the Section *Hypothalamic–pituitary–adrenal axis suppression*, below, the risk for adrenal suppression following limited use of PDN as prescribed for relapsing SSNS is very small and does not justify tapering of PDN following standard relapse treatment regimen as recommended.

### Daily PDN treatment at onset of infection to prevent relapse

- We do not recommend the routine use of a short course of low-dose daily PDN at the onset of an upper respiratory tract infection (URTI) for prevention of relapses (grade B, moderate recommendation).
- We suggest considering a short course of low dose daily PDN at the onset of an URTI in children who are already taking low dose alternate day PDN and have a history of repeated infection-associated relapses (grade D, weak recommendation).

#### Evidence and rationale

The PREDNOS 2 RCT [82], which was adequately powered, generalizable to the overall SSNS population, and at low risk of bias, evaluated 271 children with NS and URTI. The study found no benefit of administering five days of low dose PDN (15 mg per m^2^ BSA which is equivalent to 0.5 mg/kg) at the onset of URTI in preventing relapse. The finding was consistent among subgroups of children receiving alternate day PDN or children receiving alternate day PDN and other immunosuppressive agents, although the study was powered for whole group analysis only. In contrast, four smaller RCTs [83–86] including between 36 and 89 patients, reported that using low dose daily PDN at the onset of a URTI reduced the number of children with a subsequent relapse. These four studies were all at high risk of bias for one or more study attributes and were conducted in different geographic regions as compared to the low risk of bias study. Poorly designed RCTs at increased risk of bias are more likely to overestimate the efficacy of a treatment due to confounding, and/or selective or underreporting of outcomes in treatment groups [87, 88]. The baseline risk of an URTI triggering a relapse determines the number needed to treat to prevent one relapse with the intervention. Within most of the studies considered here [83–86] and in a demographic study [89], the risk is approximately 50%, but it was much lower (20%) in PREDNOS 2. Overall, there is insufficient evidence to recommend the routine use of a short course of low-dose daily PDN at the onset of an URTI for prevention of relapses. However, such an approach may be considered in children already taking low-dose alternate day PDN and at a greater risk of URTI triggering relapse. A cost-effectiveness analysis of PREDNOS 2 showed giving daily oral PDN to be dominant in health economic terms [90]. This was due to a small cost benefit driven largely by the low-cost of PDN, and reduced health-related quality-of-life associated with a relapse for the small (but clinically non-significant) additional number of children who relapsed in the placebo arm [90]. (Further information is given in Supplementary Table S5).

## Relapsing SSNS: second line treatment

### Optimal approach to children with FRNS and SDNS

- We recommend the use of maintenance treatment (see Table 5) in all patients with FRNS or SDNS (grade B, moderate recommendation).
- In patients with FRNS, we recommend either the introduction of a steroid-sparing agent as detailed below or low-dose maintenance PDN given as an alternate-day or a daily dose (grade A, strong recommendation).
- We recommend introduction of a steroid-sparing agent in children:
  - who are not controlled on therapy, or
  - who suffer a complicated relapse, or
  - with SDNS (grade B, strong recommendation)
- We recommend that the selection of the steroid-sparing agent be made in conjunction with patients or guardians in order to choose the most appropriate medication for each individual according to their values and preferences. This requires not only information on the efficacy of these medications, but also disclosure of possible side effects as listed in Table 5 (grade X, strong recommendation).
- We recommend the introduction of one of the following steroid-sparing agents (alphabetical order): calcineurin inhibitors (CNIs), cyclophosphamide (CYC), levamisole (LEV), and mycophenolate mofetil (MMF)/mycophenolic sodium (MPS) (grade A, strong recommendation).
- We recommend using RTX as a steroid-sparing agent in children with FRNS or SDNS who are not controlled on therapy after a course of treatment with at least one other steroid-sparing agent at adequate dose, especially in case of non-adherence (grade B, moderate recommendation).
- We recommend switching to a different steroid-sparing agent when a patient is not controlled on therapy with the initial agent (grade X, strong recommendation).
- We recommend considering tapering and discontinuation of maintenance treatment with PDN, LEV, MMF/MPS, or a CNI in all children in sustained remission for at least 12 months (grade X, moderate recommendation).

**Table 5:** Dose, monitoring, adverse effects, and cost of all agents used as maintenance in FRNS and SDNS patients

Therapeutic agent Dose	Monitoring	Adverse Effects	Cost
**Low Dose Alternate-Day PDN** ≤ 0.5 mg/kg/alt day, max 20 mg alt day	Quarterly: blood pressure, height, weight Yearly: ophthalmological examination	Obesity/weight gain, hypertension, diabetes mellitus, behavioral/psychiatric disorders, sleep disruption, growth failure, cushingoid features, striae rubrae/distensae, glaucoma, cataract, bone pain, avascular necrosis	Low
**Low Dose Daily PDN** ≤ 0.25 mg/kg/day, max 10 mg/day			
**Calcineurin inhibitors** **Cyclosporin A** Start: 3–5 mg/kg per day (maximum dose 250 mg) in 2 divided doses, Target: C_0_ 60–100 ng/mL or C_2_ 300–550 ng/mL (aiming for the lowest possible dose to maintain remission) **Tacrolimu**s Start: 0.1–0.2 mg/kg per day (maximum dose 10 mg) in 2 divided doses Target: C_0_ level between 3 and 7 ng/mL (aiming for the lowest possible dose to maintain remission)	Quarterly: Blood pressure CBC, creatinine, eGFR, K^+^ LFTs, lipids Uric acid (CsA) Mg^+^ (TAC) Fasting glucose (TAC) Drug levels Consider discontinuation or a kidney biopsy after 2–3 years to avoid/detect toxicity	Acute and chronic nephrotoxicity, hypertension, seizures, tremor, posterior reversible encephalopathy syndrome (PRES) Hirsutism (CsA), gum hyperplasia (CsA), diabetes mellitus (TAC) TAC drug levels can increase in case of intense diarrhea Consider risk of toxicity due to drug interactions (e.g., macrolide antibiotics, certain anti-epileptic agents, and grapefruit juice increase drug levels)	Intermediate price, CsA less than TAC
**Cyclophosphamide** 2 mg/kg per day (maximum dose 150 mg) over 12 weeks (oral) or 3 mg/kg per day (maximum dose 150 mg) over 8 weeks Single morning dose preferable No more than a single course (max TCD 168 mg/kg) Give in conjunction with alternate day oral PDN starting with a dose of 40 mg/m^2^ (1.5 mg/kg) and reducing to 10 mg/m^2^ (0.3 mg/kg) over the duration of treatment	CBC every 14 days during therapy	Leukopenia, severe infections, alopecia, nail discoloration, seizure, infertility, GI upset (abdominal pain, diarrhea), hemorrhagic cystitis, jaundice Fertile individuals must be warned of the need to avoid unplanned pregnancy (CYC can cause fetal malformation)	Low
**Levamisole** 2–2.5 mg/kg/alternate day (maximum dose 150 mg) In some cases, LEV is initially alternated with oral PDN on non-LEV days	Quarterly: CBC, LFTs Twice-yearly: ANCA titers (also at baseline)	Arthritis, vasculitic rash, neutropenia, abnormal LFTs	Low
**Mycophenolate mofetil (MMF)/mycophenolic sodium (MPS)** **MMF**: Start: 1200 mg/m^2^ per day in two divided doses every 12 hours^a^ (maximum dose 3000 mg) **MPS:** 360 mg corresponds to 500 mg of MMF Therapeutic drug monitoring using a limited sampling strategy: The most effective MPA AUC_0–12_ is above 50 mg × h/L^b^	Quarterly: CBC LFTs	Abdominal pain, diarrhea, weight loss (may be improved by the use of MPS). Leukopenia, anemia and abnormal LFTs Verrucae Fertile females must be warned of the need to avoid unplanned pregnancy (MMF/MPS can cause fetal malformations)	High; MPS more expensive than MMF
**Rituximab** 375 mg/m^2^ for 1–4 doses per course (maximum single dose 1000 mg) at weekly intervals Aim for CD19 depletion (< 5 cells/mm^3^ or < 1% total lymphocytes) Premedication is often used with antihistamine, paracetamol and steroids Repeated courses can be given Administer in remission after appropriate pre-medication under close supervision and monitoring Exclude hepatitis B and C, HIV, EBV, tuberculosis / any active infection	Quarterly: CBC LFTs CD19 counts and % IgG (at baseline, quarterly in the 1st year, then yearly)	Infusion reactions, infection, activation of latent viruses, transient or persistent IgG deficiency Serious adverse effects: tuberculosis, hepatitis B, or JC virus infection, myocardial dysfunction, risk of progressive multifocal leukoencephalopathy (PML) If infection is suspected, undertake diagnostic work-up including chest x-ray etc	High

*CBC* complete blood count, *C*_*0*_ trough level, *C*_2_ 2 h post dosing, *eGFR* estimated glomerular filtration rate, *CBC* complete blood cells, *LFTs* liver function test, *LEV* levamisole; cyclosporin A, CsA; TAC, tacrolimus; *GI* gastrointestinal, *AUC* area under the curve

Evidence and grading are given in the text

^a^Patients may be started on half dose. Dosage may be increased after 1 week in case of no side effects, e.g., leucopenia or GI discomfort

^b^A limited sampling strategy for assessing pharmacokinetic profiles was validated in children with NS being in remission on MMF monotherapy. It requires three measurements of plasma MPA at times 0 min (before administration, C_0_), 60 min (C_1_), 120 min (C_2_) after administration), and allows a good estimation of MPA-AUC_0-12_ using the formula eMPA − AUC_0−12_ = 8.70 + 4.63 * C_0_ + 1.90 * C_1_ + 1.52 * C_2_ [152]. Alternatively, the formula: eMPA—AUC_0−12_ = 7.75 + (6.49 * C_0_) + (0.76 * C_0.5_) + (2.43 * C_2_) which was originally established in adult heart transplant patients treated with concomitant CsA can be used [108, 152, 153]

#### Evidence and rationale

SSNS is a relapsing–remitting condition. Children with frequent relapses, who require frequent courses of oral PDN, particularly in the presence of comorbidities, may develop steroid toxicity (Table 5). In children with FRNS or SDNS, it is necessary to balance risks and benefits of the intervention on an individual basis. The objective is to keep each patient controlled on therapy with minimal adverse effects. In some centers, the initial approach in children with FRNS is low-dose maintenance oral PDN, while in other centers a steroid-sparing agent is immediately started.

#### Low-dose maintenance PDN

The use of low-dose PDN in children with FRNS to maintain remission is primarily based on two historic small single-arm, uncontrolled studies with alternate day [91] or daily dosing [92]. Alternate-day dosing has been more widely adopted, although this is not evidence-based. A single open-label RCT [93] involving 61 patients with FRNS found that low dose daily (0.25 mg/kg) compared with alternate-day (0.5 mg/kg) PDN reduced the risk for relapse during 12 months of follow-up (0.55 relapses/person-year compared with 1.94 relapses/person-year) and lowered one year of PDN exposure (0.27 ± 0.07 versus 0.39 ± 0.19 mg/kg/day) with no differences in adverse effects. There was some clinical evidence of reduced glucocorticoid toxicity with the daily dosing schedule. The preferred use of daily or alternate-day low dose PDN for relapse prevention in FRNS requires additional study. Transition to steroid-sparing agents is recommended in patients not controlled on therapy as defined in Table 1.

#### Steroid-sparing agents

Steroid-sparing agents used in children with SSNS include CNIs (cyclosporin A (CsA), tacrolimus (TAC)), cyclophosphamide (CYC), immune modulators (levamisole (LEV), anti-proliferative agents (mycophenolate mofetil (MMF)/mycophenolic sodium (MPS)), and anti-CD20 monoclonal antibodies, primarily rituximab (RTX). There is insufficient evidence to establish the best initial option and the optimal sequence of agents from least to most effective or least to most toxic. The choice of agent should be based on family and physician preferences and the risk profile for drug-associated complications. Factors to consider include disease type/severity, age—including onset of puberty, potential adherence, side-effect profile, comorbidities, cost and availability. In the following sections, we discuss the pros and cons of each available agent and provide a roadmap, based on the available evidence, of reasonable choices based on the clinical features of each patient with SSNS. Regarding a switch from one steroid-sparing agent to another, the same considerations apply. Moreover, we have added the definition of “controlled on therapy” to provide a timeframe for this decision.

In Table 5, we provide dose, monitoring, adverse effects, and considerations on cost for therapeutic agents that are currently used for relapsing SSNS patients. In Supplementary Table S6, we provide GRADE-based evidence, given the available RCTs (Supplementary Table S7), on the different steroid-sparing therapeutic agents. An overview of recent observational studies on steroid-sparing therapeutic agents is given in Supplementary Table S8.

### Calcineurin inhibitors

- When using CNIs, we recommend therapeutic drug monitoring to ensure optimal dosing (see below) (grade B, moderate recommendation).
- When using cyclosporin A (CsA), we recommend a starting dose of 3–5 mg/kg/day (maximum dose 250 mg) divided into 2 doses (every 12 h) to achieve trough blood levels of 60–100 ng/mL or 2 h post-dose levels of 300–550 ng/mL (grade B, moderate recommendation).
- When using tacrolimus (TAC), we recommend a starting dose of 0.1–0.2 mg/kg/day (maximum dose 10 mg) in 2 doses (every 12 h) to achieve trough blood levels of 3–7 ng/mL (grade C, moderate recommendation).
- We recommend that the lowest effective CNI dose should be given to maintain patients controlled on therapy (grade X, strong recommendation).
- We recommend avoiding prolonged use of CNIs for more than a total of 2–3 years (grade B, moderate recommendation).
- If CNIs have to be continued, we recommend that a kidney biopsy be considered after 2–3 years to exclude toxicity (grade B, moderate recommendation).

#### Evidence and rationale–Evidence for efficacy of CNIs in SSNS

CNIs have been used to treat relapsing SSNS for nearly 30 years [94–98]. Because of the lack of cosmetic side effects, TAC may be preferred to CsA. A Cochrane systematic review did not identify any RCTs comparing CsA with TAC in children with SSNS [12]. In Japan, an RCT comparing TAC and CsA is currently underway (jRCTs031180132, UMIN000004204).

#### Cyclosporin A

CNIs are effective in maintaining remission in children with FRNS and SDNS. A single RCT performed in Japan and including 108 children with FRNS/SDNS demonstrated that CsA compared with placebo reduced the risk of relapse (relapse rate ratio 0.55 (95% CI 0.37–0.82)) [99]. Observational studies have also demonstrated reduced relapse rates with CsA compared with PDN [95, 100–105]. However, many patients suffer relapses when CsA is ceased [101–104, 106]. Ishikura et al. reported that 84.7% of patients had a relapse within 2 years after completion of the 2-year CsA therapy and 59.2% of patients had regression to FRNS [106]. There are small RCTs comparing alkylating agents or MMF with CsA. Compared with alkylating agents, the number of patients relapsing by the end of therapy (6–9 months) on CsA may not differ (2 studies, 95 children: RR 0.91, 95% CI 0.55 to 1.48). However, following cessation of these medications and because the effect of alkylating agents but not CsA is prolonged after cessation, fewer children relapse after receiving alkylating agents compared with CsA alone (risk of relapse at 12–24 months; 2 studies, 95 children: RR 0.51, 95% CI 0.35 to 0.74) [12].

Two small RCTs suggested that the number of patients relapsing by 12 months may not differ between MMF and CsA (2 studies, 82 children: RR 1.90, 95% CI 0.66 to 5.46) but there is considerable imprecision in these findings. The addition of a third study to the meta-analysis indicated that the relapse rate/year may be higher with MMF than with CsA (mean difference 0.83 (95% CI 0.33 to 1.33) [12].

In RCTs, MMF is less likely to cause hypertrichosis and gum hypertrophy compared with CsA [12, 107–109] but no differences in other adverse effects (hypertension, impaired kidney function and infections) were identified. Three large observational studies [14, 110, 111] found higher efficacy in maintaining remission with CNIs compared with MMF. However, adverse effects were more common with CNIs.

#### Tacrolimus

The use of TAC in SSNS is based on the effectiveness of CsA in SSNS [95], on the results of observational studies [14, 97, 110] and the efficacy of TAC in pediatric kidney transplantation.

#### Cyclosporin A versus tacrolimus

There are no RCTs that compare TAC to CsA. A trial of TAC versus CsA for FRNS in children is being conducted in Japan (jRCTs031180132, UMIN000004204). Only small-number case series are available [98, 112–114]. Switching from CsA to TAC is only effective in reducing cosmetic side effects but warrants caution for the potential onset of diabetes mellitus [114].

#### Monitoring of CNIs

Patients should be monitored for side effects as indicated in Tables 4 and 5. Therapeutic drug monitoring indications are given below.

#### Cyclosporin A: toxicity profile

Nephrotoxicity is the most problematic side effect of CsA, and its risk is increased after use for > 2 years [115, 116]. CsA-induced chronic nephrotoxicity cannot be diagnosed based only on urinalysis or blood tests. It is advisable to avoid prolonged use of CsA and to consider its discontinuation or to perform a kidney biopsy after 2–3 years to avoid/detect toxicity. However, there is no definitive evidence supporting the necessity of kidney biopsy in SSNS treated with CNIs. Recent clinical studies of micro emulsified CsA [100, 117] have demonstrated a lower incidence of nephrotoxicity.

Cosmetic side effects, such as hypertrichosis and gum hyperplasia, are common with CsA [100–105]. Infections, hypertension, and posterior reversible encephalopathy syndrome (PRES) are also known complications of CsA therapy [100–105, 118].

#### Tacrolimus: toxicity profile

Among the side effects of TAC, new-onset diabetes mellitus is important. Particular caution is required when TAC is used in patients with a family history of diabetes mellitus or if risk factors for impaired glucose tolerance (e.g., obesity) are present [119]. Renal interstitial fibrosis has also been reported, as with CsA. One report described a significant association between higher TAC trough levels and renal interstitial fibrosis [112].

#### Cyclosporin A: therapeutic drug monitoring

The dose of CsA should be adjusted with drug monitoring based on assays validated against tandem mass spectrometry. According to a multicenter, prospective RCT of Sandimmun® conducted in Japan on 44 children with FRNS, sustained remission was significantly higher in the dose-adjusted group (initially the dose was adjusted to maintain blood trough levels within 80–100 ng/mL for the first 6 months, and then within 60–80 ng/mL for the next 18 months) compared with the 2.5 mg/kg fixed-dose group (initially the dose was adjusted to maintain blood trough levels within 80–100 ng/mL for the first 6 months, but then fixed at 2.5 mg/kg for the next 18 months) (50 vs. 15%; *p* < 0.01) [95]. A multicenter observational study assessed Neoral® [101], a microemulsified preparation of CsA, in 62 children with FRNS, with adjustment of the dose using the same target trough levels as stated above. This study reported that microemulsified CsA was effective and safe (relapse-free survival rate at month 24, 58%; incidence of nephrotoxicity, 8.6%), similar to conventional CsA [100].

The AUC_0–4_ (area under the time-concentration curve) of CsA is best predicted by C_2_ (CsA blood concentration at 2 h post-dose) in kidney transplant patients [120]. Similar findings were reported in children with NS [121]. A multicenter, prospective, RCT in Japan on 93 children with FRNS compared two different target C_2_ levels: a higher C_2_ group (target C_2_ 600–700 ng/mL for the first 6 months, followed by 450–550 ng/mL for the next 18 months) and a lower C_2_ group (target C_2_ 450–550 ng/mL for the first 6 months, followed by 300–400 ng/mL for the next 18 months) [94]. At 24 months, the relapse rate was significantly lower in the higher C2 group than the lower C_2_ group (0.41 vs. 0.95 times/person-year; hazard ratio, 0.43; 95% confidence interval, 0.19 to 0.84; *p* < 0.05). The rate and severity of adverse events were similar in both treatment groups [94].

Absorption of oral CsA after pre-meal administration (15–30 min prior to a meal) is greater than post-meal administration so it may be preferable to administer CsA before meals. The main priority is to give it in a consistent manner. Concomitant use with other drugs requires adequate attention since macrolide antimicrobials and many other drugs can affect metabolism. Grapefruit juice should be avoided as it inhibits metabolism of CsA and causes increased blood concentrations of the drug.

#### Tacrolimus: therapeutic drug monitoring

Tacrolimus requires adjustment of dosage by monitoring blood concentration. However, safe and effective dosage and mode of administration of TAC have not yet been established in children with SSNS. Suggested dosage and blood levels are extrapolated from data on kidney transplant recipients.

#### General considerations of benefit/risk of using CNIs

CsA is very effective in the treatment of FRNS/SDNS and allows steroid tapering and discontinuation in the majority of patients [95, 100–105]. The shortcoming of CsA therapy is that many patients experience relapse after termination of CsA therapy (CsA dependence) [101–104, 106]. Moreover, CNIs have a variety of side effects, including nephrotoxicity. In comparison to CsA, TAC has fewer cosmetic side effects.

#### Tapering and discontinuing of CNIs

If a child remains in sustained remission for at least 12–24 months and off steroids, CNI discontinuation should be considered to avoid nephrotoxicity [115, 116]. Tapering CNI dose to zero over about 3 months rather than discontinuing abruptly may be preferable because in case of a reappearance of proteinuria during tapering, reestablishing the initial CNI dose may be sufficient to avoid a relapse and a course of oral PDN while establishing that the patient still needs maintenance therapy.

### Cyclophosphamide

- When using cyclophosphamide (CYC):
- We recommend starting when the patient is in steroid-induced remission and using either a single course of 2 mg/kg per day (maximum dose 150 mg) given orally for 12 weeks (grade B, moderate recommendation). or a single course of 3 mg/kg per day (maximum dose 150 mg) for 8 weeks given orally (grade B, moderate recommendation).
- We recommend that the maximal cumulative dose of CYC not exceed 168 mg/kg (grade C, moderate recommendation).
- We recommend that, if adherence is uncertain, a single course of monthly intravenous CYC (500 mg/m^2^ per dose (max single dose 1 g) × 6 months) can be given (grade B, moderate recommendation).
- We suggest administering CYC in combination with alternate-day oral PDN starting with a dose of 40 mg/m^2^ (1.5 mg/kg) and reducing to 10 mg/m^2^ (0.3 mg/kg) over the course of treatment (grade D, weak recommendation).
- We recommend monitoring for neutropenia (absolute neutrophil count < 1500/µL) with complete blood counts every 2 weeks (grade D, weak recommendation) and ceasing CYC if the child develops leukopenia (< 4000/µL) or neutropenia (< 1500/µL) or significant thrombocytopenia (< 50,000/µL) (grade X, strong recommendation) and restarting after recovery of blood cell counts using a lower dose (grade X, strong recommendation).
- We recommend maintaining a high fluid intake to ensure a high urine output during treatment (grade C, moderate recommendation).

#### Evidence and rationale–Efficacy of CYC

A meta-analysis of 4 RCTs with 161 participants [12] comparing CYC with PDN or placebo showed a reduction in the number of relapses by 6 to 12 months (4 studies, 161 children; RR 0.47 [95% CI 0.34, 0.66]) [12]. A single course of monthly intravenous doses of CYC at a dose of 500 mg/m^2^ per dose (max single dose 1 g) × 6 months can be given when adherence is an issue [122, 123].

A review of 38 RCTs and observational studies assessing alkylating agents (CYC and chlorambucil) [13] including 1504 patients and 1573 courses and published between 1960 and 2000, indicated sustained remission rates of 72% after 2 years and 36% after 5 years for FRNS; the rates were 40% and 24%, for SDNS respectively. The maintenance of sustained remission declines with time, i.e., 44–57% at 1 year, 28–42% at 2 years, 13–31% at 5 years [124–128]. The effect may be lower in children below 3–5.5 years of age [125, 127, 129].

In comparison with CsA courses limited to 6–12 months (two RCTs), the actual percentage of sustained remission at 2 years for alkylating agents was higher, indicating that the effect of alkylating agents lasted longer than CsA after cessation of therapy [12]. One non-randomized comparator trial ([130], *n* = 46) suggests that RTX is non-inferior to CYC in maintaining remission over 1 year.

Cyclophosphamide treatment should be initiated after the patient has achieved remission and has been treated with the recommended dose of PDN for relapse. Published literature examining the use of CYC does not directly address whether co-intervention with PDN is necessary to reduce relapses or risk of adverse effects. Descriptions of continuation of PDN or concomitant administration of PDN while on CYC vary widely in the literature. Protocols ranged from PDN 10–40 mg/m^2^ either daily or alternate days, to 60 mg/m^2^ every other day. Tapering at the end of treatment was also highly variable [13, 96, 124, 131]. Due to substantial variation in practice, administering CYC in combination with alternate-day oral PDN starting with a dose of 40 mg/m^2^ (1.5 mg/kg) and reducing to 10 mg/m^2^ (0.3 mg/kg) over the duration of treatment was considered as reasonable practice by the guideline committee. Alternate-day oral PDN may help to reduce the risk of neutropenia when starting CYC initially.

#### Toxicity profile

Leukopenia occurred in 32.4% of patients on CYC and was more common with CYC alone than with CYC plus PDN protocols (22/38 vs. 8/52) [13]. The Latta meta-analysis reported reversible alopecia in 17.8%, infections in 1.5%, hemorrhagic cystitis in 2.2%, and malignancy in 0.2%. However, the cumulative dose used in many of the included studies was higher than current recommendations [13]. Studies using lower cumulative doses [124, 132] report transient leukopenia (7 to 23%) as the main adverse effect with transient alopecia and hemorrhagic cystitis occurring in ≤ 1%. However, long-term follow-up studies in patients who have been treated with these lower doses are lacking.

The incidence of gonadal dysfunction (amenorrhea and premature menopause in females and infertility for males and females) is dependent upon the patient’s age, sex, and cumulative dose of CYC, regardless of how the medication is administered [133–135]. Data compiled from 8 studies on 119 male patients [13] demonstrated a strong dose-dependent risk for infertility (see Supplementary Table S9).

*Females:* CYC may induce depletion of ovarian follicles and shrinkage and fibrosis of the ovaries. Women treated before the age of 25 are at a lower risk of infertility than those treated after the age of 30 [136]. CYC is associated with congenital (or fetal) malformations and should be avoided during the first 10 weeks of gestation.

Girls and younger women are less likely to experience ovarian failure with CYC exposure as they have a greater ovarian reserve. Thus, it appears that women < 20 years are unlikely to experience ovarian failure with an initial course of CYC (0 to 4%), whereas the risk is significant in women > 30 (23 to 54%) and > 40 (75%) [133, 137].

*Males:* CYC causes a decrease in sperm count and with higher doses and treatment duration can lead to irreversible azoospermia. The severity and risk of gonadal toxicity due to CYC depend on the gonadal activity at the time of treatment (prepubertal vs. sexually mature males) and the total cumulative dose. Testicular injury is reported to occur in boys and men after 7 to 9 g of CYC; recovery is documented in some patients [134]. Lentz et al. reported no increased risk of gonadal injury at total doses below 168 mg/kg [138]. Patients should be monitored for side effects as indicated in Tables 4 and 5.

#### Balance of risks and benefits

Alkylating agents, in particular CYC, have been used in pediatric NS for over 5 decades. Considering that other alkylating agents, i.e., chlorambucil, are currently rarely used for children with SSNS and showed a worse safety profile compared to CYC [13], we have focused on CYC. CYC is relatively inexpensive and monitoring requirements involve relatively inexpensive and readily available standard tests. Compared to agents like LEV, MMF and CNI, CYC is administered for a short-term course with a sustained effect after discontinuation. Thus, safety monitoring is needed for a shorter duration. The risk of gonadal toxicity is reduced with appropriate restriction of total cumulative dose. CYC should be used with caution in peri-pubertal males. The risk of hemorrhagic cystitis is very low with oral therapy at this recommended dose and with maintenance of fluid intake and diuresis. Leukopenia/neutropenia is the most common AE expected, and dose adjustment is a component of all protocols. Of note, CYC’s use requires additional treatment with oral PDN, which may promote further steroid toxicity. On balance, the potential risks of CYC may favor the use of other steroid-sparing agents, if available.

### Levamisole

- We recommend levamisole at a dose of 2–2.5 mg/kg given on alternate days (with maximum dose of 150 mg) after remission was achieved by PDN at recommended dose (grade B, moderate recommendation).
- We recommend ANCA measurement at baseline, if available and every 6–12 months during therapy (grade X, moderate recommendation).
- We recommend monitoring clinically for rash and measuring complete blood count and hepatic transaminases every 3–4 months (grade X, moderate recommendation).

#### Evidence and rationale–Efficacy evidence for levamisole

A recent international multicentre RCT has enhanced the quality of evidence for the effectiveness and safety of LEV. Gruppen (2018) [139] compared LEV therapy to placebo in 99 children with FRNS or SDNS and found a significant reduction in the number of relapses at 12 months (RR of relapses on LEV 0.77, 95% CI 0.61 to 0.97) [12]. Thus, 26% of children in the LEV group compared with 6% in the placebo group remained in remission at 12 months. Eight RCTs (474 participants) combined in a meta-analysis [12] indicated a benefit of LEV over PDN, placebo or no treatment (RR 0.52, 95% CI 0.33 to 0.82).

Small comparative RCTs comparing LEV with CYC [140, 141] showed no difference in efficacy but were not powered to show a difference. An RCT found no difference in efficacy between MMF and LEV but MMF levels were not measured [142]. The Gruppen 2018 [139] and Sinha 2019 [142] studies suggest that LEV may be more effective in FRNS than SDNS. These recent RCTs [139, 142] used a dose of LEV of 2.5 mg/kg/alternate day, maximum 150 mg, for 12 months. Most other recent studies used doses of 2–3 mg/kg on alternate days for 6–24 months. Some observational studies have used doses of 2–2.5 mg/kg daily for 4–24 months [143–149] with three studies [147–149] suggesting reductions in relapse rates in patients who had not responded to alternate-day LEV. These data require further larger RCTs, powered to detect a difference, if any, for confirmation.

#### Toxicity profile

Common adverse effects include rashes, leukopenia, and abnormal liver function tests. These are generally transient and reversible on discontinuation of therapy. Rarely ANCA positive arthritis (2% in Gruppen 2018 [139]), rash and other vasculitis symptoms have been reported, which resolve upon LEV discontinuation.

#### Balance of risks and benefits

While most adverse effects are transient and reversible on discontinuation, the main emerging threat is ANCA-positive vasculitis particularly with prolonged use. Regular monitoring as indicated in Tables 4 and 5 is advised with cessation of therapy if ANCA titers are positive.

#### Tapering/discontinuation

Available studies do not comment on this. Discontinuation without tapering should be considered once the patient is in sustained remission and off steroids for at least 12 months.

#### General considerations on the use of levamisole

LEV is an immunomodulant which has been used for over 3 decades in NS. Its low cost makes it a useful option, particularly in low resource settings. However, it is unavailable in some countries. Lack of nephrotoxicity and ease of monitoring are other major advantages. When introducing this agent, some physicians prefer to maintain low-dose alternate-day PDN on non-LEV days for a few months, then oral PDN is tapered and stopped, and the patient remains on LEV alone.

### Mycophenolate mofetil/mycophenolic sodium

- When using mycophenolate mofetil MMF, we recommend a starting dose of 1200 mg/m^2^ BSA (maximum dose 3000 mg) divided into two oral doses every 12 h (grade B, moderate recommendation).
- Alternatively, we recommend using the corresponding mycophenolic sodium (MPS) dose, i.e., 360 mg of MPS corresponds to 500 mg MMF (grade B, moderate recommendation).
- We suggest starting MMF/MPS therapy while the child is still receiving alternate-day steroid therapy since the immunosuppressive effect of MMF/MPS is delayed (grade C, weak recommendation). In most children, alternate-day steroids can then be tapered and discontinued within 6–12 weeks.
- We recommend using therapeutic drug monitoring, aiming for a 12-h mycophenolic acid (MPA) area under the curve above 50 mg h/L in patients not controlled on MMF therapy despite using recommended dosing (grade B, moderate recommendation).
- We recommend that sexually active adolescent females only receive MMF/MPS if they are using adequate contraception (grade X, strong recommendation).

#### Evidence and rationale–Dosing and therapeutic drug monitoring

The standard dose for MMF in RCTs is 1200 mg/m^2^/day divided into two doses every 12 h orally with a maximum daily dose of 3000 mg. Five hundred mg of MMF corresponds to 360 mg of MPS. Patients may be started on half dose and dosage may be increased after 1 week in case of no side effects, e.g., leukopenia or GI discomfort.

#### Monitoring of MMF/MPS

Patients should be monitored for side effects as indicated in Tables 4 and 5. Therapeutic drug monitoring indications are given below.

#### Therapeutic drug monitoring

Assessment of mycophenolic acid (MPA) trough levels is not recommended as there is a poor correlation with efficacy and safety using single pre-dose measurements [150, 151]. A limited sampling strategy for assessing pharmacokinetic profiles was established in children with NS on MMF monotherapy being in remission [152], whereas such a profile is not available for those on MPS. It requires three measurements of plasma MPA at times 0 min (before administration, C_0_), 60 min (C_1_), and 120 min (C_2_) after administration, and allows a good estimation of MPA-AUC_0-12_ using the formula eMPA − AUC_0−12_ = 8.70 + 4.63 * C_0_ + 1.90 * C_1_ + 1.52 * C_2_ [152]. In children with FRNS with MPA AUC_0-12_ > 50 mg × h/L estimated using the formula eMPA—AUC = 7.75 + (6.49 * C_0_) + (0.76 * C_0.5_) + (2.43 * C_2_) [108, 153], the efficacy of MMF was similar to that of CsA [108]. The latter formula was originally established in adult heart transplant patients treated with concomitant CsA. We recommend using therapeutic drug monitoring in patients not controlled on MMF therapy despite adequate dosing aiming for eMPA-AUC_0-12_ > 50 mg × h/L. For this purpose, either one of the above mentioned formulas can be used [108, 152, 153]. It should be noted that immunoassays for the determination of MPA plasma levels measure 10–20% higher MPA plasma levels than high-performance liquid chromatography (HPLC) or mass spectrometry (MS) due to cross-reactivity with MPA metabolites [154, 155].

#### Efficacy of MMF/MPS

No RCTs have compared MMF or MPS with PDN in children with FRNS or SDNS. However, numerous observational studies [156–160] (Supplementary Table S8) have reported that MMF or MPS are more effective than PDN in maintaining remission in children with FRNS or SDNS. These studies showed an approximately 50% reduction in the relapse rate on MMF/MPS, enabling reduction in dose or cessation of PDN. Studies have not specifically compared the relative efficacies of MMF/MPS in children with FRNS or SDNS.

Four RCTs compared MMF with other steroid-sparing agents in FRNS and SDNS. Three RCTs compared MMF with CsA in 142 children. Two RCTs [107, 108] combined in meta-analysis found no difference in the number of children with relapse between MMF and CsA (82 children: RR 1.90, 95% CI 0.66 to 5.46) [12]. However, the relapse rate/year was higher in children treated with MMF compared with CsA (3 studies, 142 children: mean difference 0.83, 95% CI 0.33 to 1.33) when a third study was included [12]. One RCT compared MMF with LEV and found no difference in the number of children with relapse at 12 months between treatments [142]. MPA levels were not measured in this study.

Three observational studies involving 312 children with FRNS or SDNS compared MMF with TAC [14, 110] or CsA [111]. MPA levels were not monitored in these studies. Two of these studies [14, 111] found better efficacy for maintaining remission with CNIs compared with MMF though adverse effects were more common with CNIs.

#### Toxicity profile

The most common adverse effects of MMF are abdominal pain, loss of appetite, diarrhea, and weight loss. This is less likely to occur with enteric-coated MPS. However, some individuals tolerate MMF better than MPS. Other adverse effects are leukopenia, anemia and elevated hepatic transaminases. These adverse effects are uncommon and usually mild. Monitoring for side effects should be done as indicated in Tables 4 and 5. MMF/MPS is teratogenic in the early months of pregnancy so effective contraception should be used by all sexually active female adolescents during MMF/MPS therapy. In males, recent evidence in patients receiving MMF/MPS after kidney transplantation and a large meta-analysis of different drugs [161] indicates that the risk of congenital malformations is comparable to that of the general population [162].

#### General considerations of benefit/risk of using MMF/MPS

There is now extensive documentation of the successful and safe use of MMF in children with FRNS and SDNS but studies did not differentiate between these groups. In clinical practice, MMF appears more effective in children with FRNS. Its advantages consist in lack of nephrotoxicity and of cosmetic side effects compared to CNIs.

#### Tapering and discontinuing of MMF/MPS

There are no studies on the duration of MMF/MPS use or on when to discontinue MMF/MPS. If the child achieves control on therapy for at least 12 months, then consideration may be given to tapering MMF over 3–6 months and then discontinuing it. As with CNIs, the advantage of tapering over abrupt discontinuation is that in case of proteinuria, re-establishment of MMF at initial dose may be sufficient to avoid a relapse while establishing that the child still requires maintenance treatment. The use of more extended periods may be considered, especially in peri-pubertal age or in the presence of previous severe steroid toxicity.

### Rituximab

- We recommend using RTX as a steroid-sparing agent in children with FRNS or SDNS who are not controlled on therapy after a course of treatment with at least one other steroid-sparing agent at adequate dose, especially in case of non-adherence (grade B, moderate recommendation). This is especially preferable, both in terms of safety and of effectiveness, above the age of 7–9 years (grade C, weak recommendation).
- When using RTX, we recommend a dosage of 375 mg/m^2^ for each infusion, ranging from 1 to 4 infusions (maximum single dose 1000 mg) preferably when the patient is in remission (grade C, moderate recommendation).
- We recommend monitoring CD19( +) total B cell counts at baseline and following RTX treatment at 7 days post-infusion to ensure adequate B cell depletion indicated by an absolute CD19 cell count < 5 cells/mm^3^ or < 1% of total lymphocytes (grade B, strong recommendation).
- We recommend monitoring IgG levels at baseline and periodically following RTX treatment to detect hypogammaglobulinemia (IgG below age-related normal range) (grade B, strong recommendation).
- We recommend premedication with paracetamol/acetaminophen, antihistamines and/or steroids (grade B, moderate recommendation).
- Following RTX infusion/s, we recommend tapering off oral PDN and other steroid-sparing agents within 2–3 months (grade B, strong recommendation).

#### Evidence and rationale

In terms of dosing regimen, the original course of RTX used for lymphoma patients required 375 mg/m^2^ given as an IV infusion weekly for 4 doses. The RTX protocols used in the available RCTs and observational studies in children with FRNS/SDNS included single, 2, 4, and 7 infusions. In addition to variability in the number of RTX infusions, there has been variation in RTX dosing, ranging from 375 to 1500 mg/m^2^ per treatment, although most studies used 375 mg/m^2^. The dose of 750 mg/m^2^ has not been associated with a better response rate than 375 mg/m^2^; however, a lower dose of RTX (100 mg/m^2^) has been associated with the risk of earlier relapse (reviewed in [163] and in [164]). In terms of infusion number per course of RTX treatment, the use of a single infusion at standard dose followed by monitoring of CD19 ( +) cells at 7 days is derived from studies performed in adults with ANCA-associated renal vasculitis and membranous nephropathy. If at 7 days post-infusion, the percentage of total B cells is < 1% of total lymphocytes this indicates adequate B cell depletion [165]. Reconstitution of B cells is defined when total B cell counts are > 5/mm^3^ in absolute number [166].

#### Efficacy of RTX

During the last decade, a number of RCTs have shown that RTX is reasonably safe in the short term and relatively effective when compared to other immunosuppressants as a steroid-sparing treatment. However, studies differ in terms of populations, number of doses of RTX, additional medications and comparators. Unlike other immunosuppressants, the lack of long-term follow-up in RTX-treated patients must be considered at the time of clinical decision.

Eight RCTs have evaluated the efficacy of RTX in children with FRNS or SDNS. Four RCTs evaluated 1 to 4 doses of RTX in children with SDNS and CNI dependence compared with placebo [167, 168] or CNIs [169, 170]. Four studies compared 1 to 2 doses of RTX in children with SDNS or FRNS on low-dose PDN compared with TAC [171], low dose PDN [172, 173], or low dose MMF [174]. A meta-analysis showed that the number of patients with relapse fell by 80% by 6 months and 50% by 12 months after treatment [12]. Longer duration of remission was seen in children whose relapses were previously managed with PDN alone [172, 173]. Moreover, a large retrospective study assessing RTX use in more than 500 children with FRNS/SDNS showed that patients were 19% more likely to relapse for each additional steroid-sparing agent received prior to RTX, and that younger age at first infusion was associated with earlier relapse [164, 175, 176].

#### Toxicity profile

Adverse events were generally limited to mild infusion reactions. There was no increase in infections. RTX-related neutropenia (RRN) has been well documented in the literature, although the exact mechanism is not well known. In children, RRN is usually not associated with serious bacterial or viral infections and most of the reported infections are self-limiting. Supplementation with granulocyte colony stimulating factor (G-CSF) may not be needed, especially in late onset neutropenia, i.e., neutropenia occurring 4 weeks after last RTX infusion [177–179].

No deaths or serious adverse reactions were recorded in RCTs on the use of RTX in children with SSNS. While there are case reports of fatal lung fibrosis, immune-mediated ulcerative colitis, fulminant myocarditis, *Pneumocystis jiroveci* pneumonia following RTX use in children with SSNS, a retrospective survey of 511 children with SSNS and treated with RTX [180] identified only two children with life-threatening but non-fatal complications (*Pneumocystis jiroveci* pneumonia, myocarditis). However, prolonged and significant reduction of total memory and switched memory B cells together with hypogammaglobulinemia has been demonstrated in patients following RTX, particularly in young patients with SSNS [181].

#### Monitoring

Exclusion of certain infections and monitoring for side effects should be done as indicated in Tables 4 and 5.

#### General considerations of risk and benefit

RTX treatment has proven reasonably safe and effective for both FRNS and SDNS. Given its uncertain long-term safety profile, it is advisable to use RTX as a second-line steroid-sparing agent in children who are not controlled on therapy with a first-line steroid-sparing agent. Since long-term side effects such as hypogammaglobulinemia appear to be more likely and efficacy appears to be less convincing in younger children, the use of RTX may be reserved for older children.

#### Repeat infusion treatment with RTX

Following the first course of RTX, diverse approaches to repeated courses have been proposed, based either on disease relapse, on B cell reconstitution or on time elapsed from the initial treatment. Evidence for the most correct approach is lacking [164]. Based on a recent retrospective survey, 30 of 346 included children tolerated up to 7 courses of RTX infusions (mainly dosed with 375 mg/m^2^/course) with an acceptable side effect profile (most common hypogammaglobulinema, followed by infections and neutropenia) and good efficacy [182].

#### Tapering and discontinuing of other immunosuppressive agents post-RTX

It is unknown to what degree other immunosuppressive agents should be tapered or discontinued following RTX administration. In most studies, PDN at alternate-day doses was tapered off within 2 months before CNIs were reduced and stopped. If patients were taking MMF and mizoribine, these drugs were discontinued after the first dose of RTX. A recent study [180] demonstrated that treatment response depends on both RTX dose and on the use of maintenance immunosuppression. The study documented that in complicated FRNS and SDNS patients, giving “low dose”, i.e., 375 mg/m^2^ RTX and maintaining immunosuppression (IS), most frequently with MMF but in some cases with either CNI or oral PDN, was equivalent in terms of median relapse-free period to giving higher doses without maintaining IS after RTX [180]. In SDNS, a small prospective cohort study found that relapse-free survival 12 months after RTX therapy was higher in children receiving MMF than in children not receiving MMF [183]. An RCT evaluating MMF post-RTX treatment in “complicated” FRNS and SDNS showed that this approach was helpful in preventing relapse in 80% of patients [166]. An RCT comparing maintenance MMF to repeated RTX infusions in children with SDNS is ongoing (RITURNS II Study, NCT03899103). The use of CNIs following RTX infusions may be equally helpful, but this has not been formally assessed. These data suggest that in children with SDNS not controlled on RTX alone, following subsequent RTX infusions, the strategy of maintaining an oral steroid-sparing agent (MMF or a CNI) for at least 6 months may promote sustained remission.

#### RTX discontinuation

As with all steroid-sparing agents and even more with RTX given its long-lasting effect, once the child is controlled on therapy, RTX infusions should be discontinued.

#### Other anti-CD20 monoclonal antibodies

In addition to RTX, other monoclonal antibodies targeting B cells or modulating their function or depleting plasma cells have been employed in the treatment of SSNS.

#### Ofatumumab

Ofatumumab, in contrast to rituximab, is a fully humanized anti-CD20 monoclonal antibody. A case report described two boys, aged 3 and 14 years, with persistent SSNS, who were allergic to rituximab. Both children achieved a prolonged remission exceeding 12 months following the administration of a single dose of ofatumumab [184]. However, a recent clinical trial comparing RTX and ofatumumab in randomized 140 children with SDNS and found that there was no difference in the percentage of participants who relapsed at 12 or 24 months [185].

### Combination of more than one steroid-sparing agent

- We recommend enrolling children with severe FRNS or SDNS who have failed to achieve stable remission or who present significant treatment toxicity despite at least one steroid-sparing agent at adequate dose, in a clinical trial, if available (grade X, strong recommendation).

#### Evidence and rationale

The combination of different steroid-sparing agents is not supported by adequate evidence. There are no RCTs that compare the combination of CNI plus MMF vs. CNI or MMF alone. There is a single observational study involving 130 Pakistani children with SSNS. Of these 20 had suboptimal response to MMF and CsA was added. Nineteen out of 20 benefited but only 4 had CR and 9 were CNI-dependent. In a retrospective publication on the use of RTX [180], the prolonged use of MMF or other steroid-sparing agents following a single cycle of RTX was found to induce stable remission in those receiving low-dose RTX (375 mg/m^2^ per course) but there was no increase in benefit in those receiving higher doses of RTX (750 mg/m^2^ or higher). We suggest that if children with FRNS or SDNS are controlled on therapy with more than one immunosuppressant (i.e., steroid-sparing agent plus maintenance PDN or CNI plus MMF), discontinuation of the most toxic agent be implemented.

### Other steroid-sparing agents

- We recommend that mizoribine, azithromycin, azathioprine or adrenocorticotropic hormone (ACTH) not be used to treat children with SSNS (grade B, moderate recommendation).

#### Evidence and rationale

A single RCT found no definitive benefit of azithromycin compared with PDN in the initial episode of SSNS [57]. Single RCTs found no benefit of azathioprine, ACTH or mizoribine in children with FRNS/SDNS [186–188].

## Adjunctive measures

### Management of volume status, edema, and blood pressure

#### General measures

- We recommend evaluating the volume status of a child in the acute nephrotic state (grade A, strong recommendation).
- We do not recommend routine fluid restriction in SSNS patients (grade C, moderate recommendation).
- We suggest fluid restriction in case of hyponatremia (< 130 meq/L) and/or severe edema in a hospital setting (grade C, weak recommendation).
- We recommend a low-salt diet (suggested maximum dose of 2–3 meq/kg/day) during relapses with moderate or severe edema, and normal salt intake while in remission (grade C, moderate recommendation).
- We recommend monitoring for hypertension in all children with SSNS and following current hypertension guidelines in children with confirmed, persistent hypertension (grade A, strong recommendation).
- We recommend against ACEi or ARBs administration in SSNS to control edema or high blood pressure in relapse (grade X, strong recommendation).

#### In case of hypovolemia or AKI

**Fig. 3:**
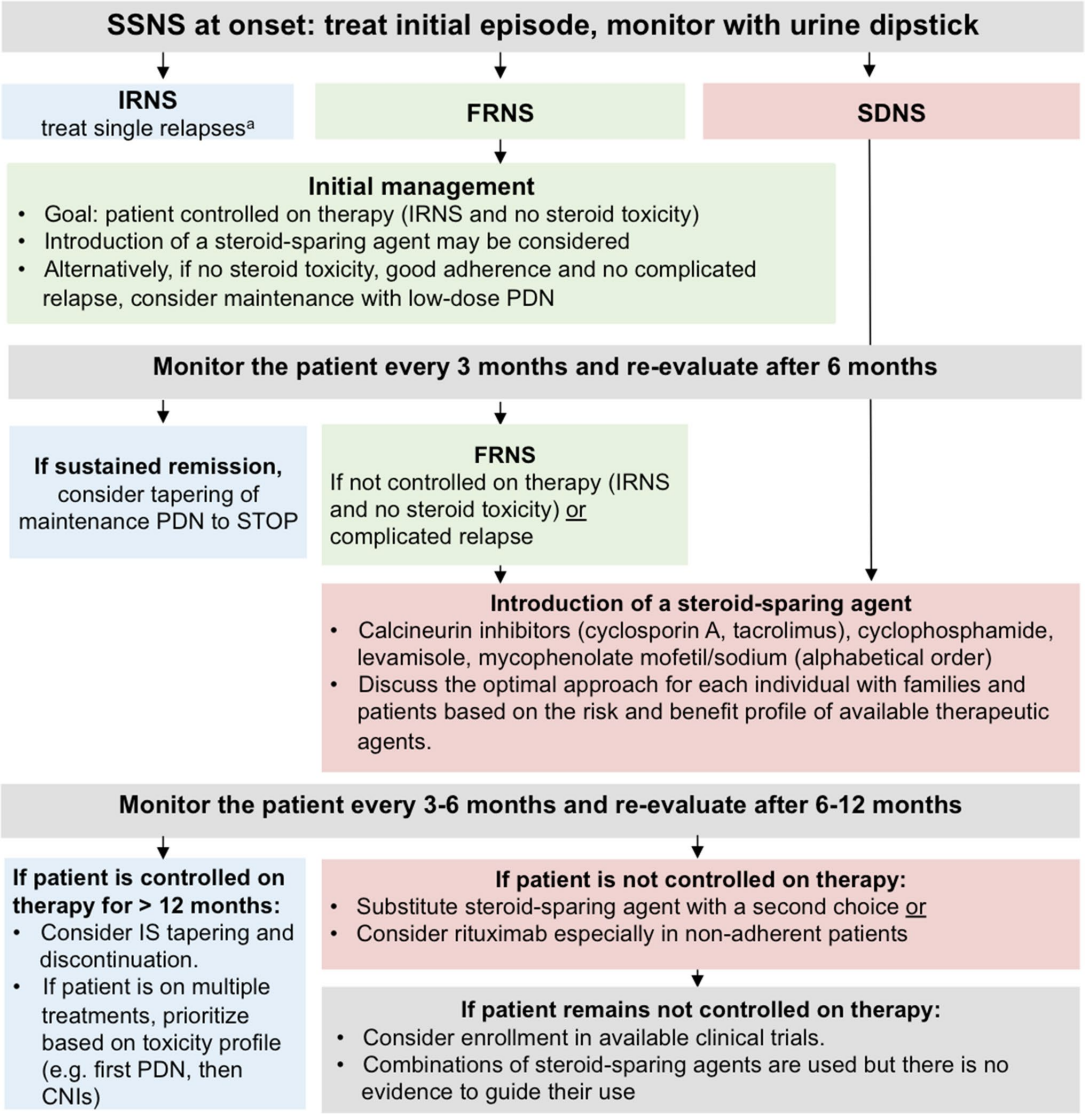
Algorithm for management of children with SSNS. Details on the risk and benefit profile of the various steroid-sparing agents are given in Table 5 and Supplementary Table S6. *IRNS* infrequently relapsing nephrotic syndrome, *FRNS* frequently relapsing nephrotic syndrome, *SDNS* steroid-dependent nephrotic syndrome, *PDN* prednisone/prednisolone, *CNI* calcineurin inhibitors. ^a^As recommended in the text

- In patients with signs of hypovolemia, we recommend withholding diuretics due to the risk of thrombosis, hypovolemic shock and AKI, and discontinuing ACEi or ARBs (grade X, strong recommendation).
- We recommend using 20% or 25% albumin infusions in patients with signs of hypovolemia (including oliguria, AKI, prolonged capillary refill time, tachycardia, and abdominal discomfort) and adding furosemide (1–2 mg/kg given i.v.) in the middle and/or at the end of the infusion if volume has been restored and urine output is insufficient (grade C, moderate recommendation).
- In cases of hypovolemic shock and/or hypotension, we suggest using 4% or 5% albumin without furosemide (grade C, weak recommendation).
- In cases of AKI without hypovolemia, we recommend general management of AKI including fluid management, avoidance of nephrotoxic agents and modification of medication dosage when appropriate (grade X, strong recommendation) (Fig. 3).

#### Management of severe edema

- In patients with severe edema, we recommend albumin infusions of 0.5–1 g/kg of 20% or 25% albumin given over a period of 4–6 h and adding furosemide (1–2 mg/kg given i.v. over 5–30 min) in the middle and/or at the end of the infusion in the absence of marked intravascular volume contraction and/or hyponatremia (grade C, moderate  recommendation).
- We recommend careful use of albumin infusions especially in hypertensive patients or those with decreased urine output to prevent hypervolemia and pulmonary edema (grade X, strong recommendation).
- In a fluid-overloaded, edematous, hypertensive child, we suggest considering antihypertensive treatment with diuretics combined with fluid and salt restriction (grade C, weak recommendation).

##### Evidence and rationale

**Fig. 4:**
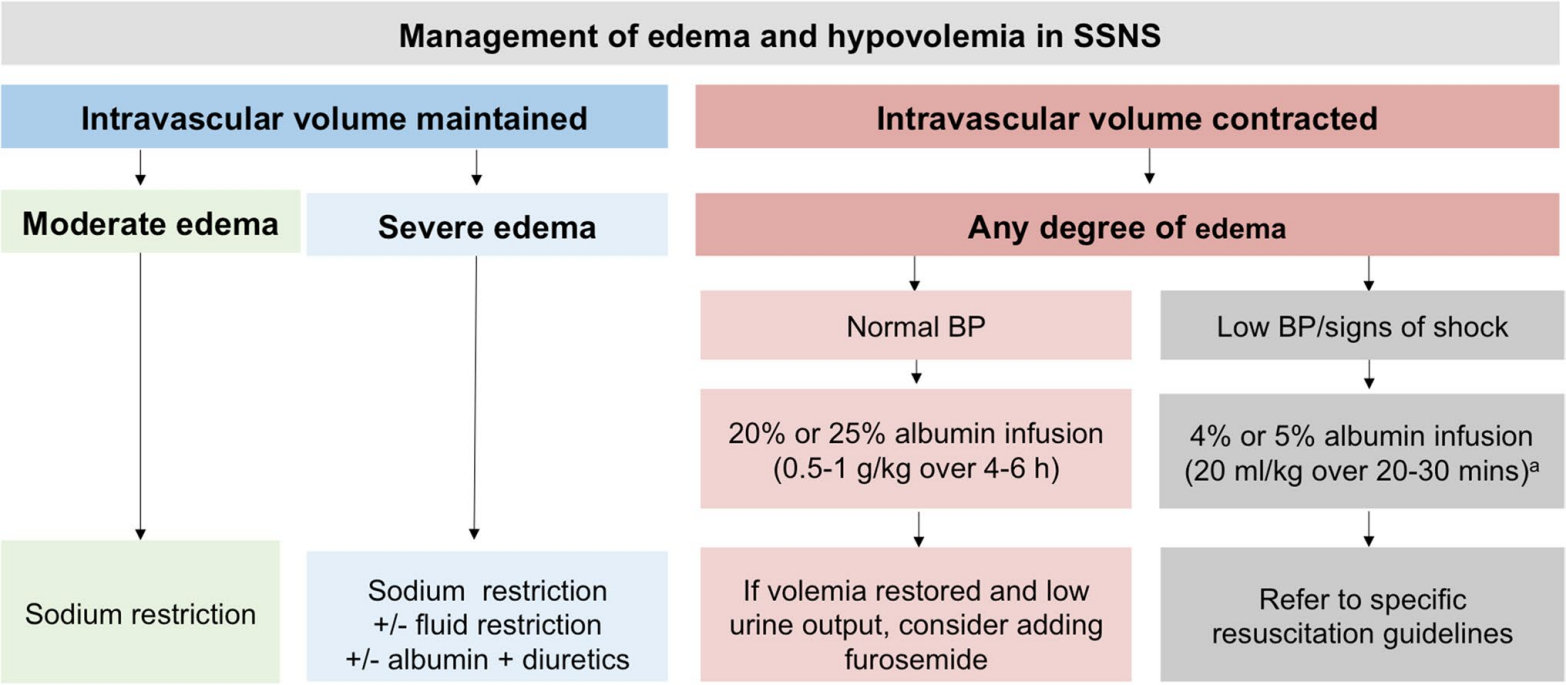
Algorithm for the management of edema and hypovolemia in SSNS. First, the volemic state of the child should be assessed. In case of maintained intravascular volume, we suggest treating moderate edema by low salt diet only, approximately 2 to 3 mEq per day (2000 mg/day in larger children), the amount of sodium required for a growing child, but not fluid restriction. In case of severe edema, fluid restriction is advocated in a hospital setting, with loop diuretics. Fluid restriction is also indicated in case of hyponatremia < 130 meq/L (considering false hyponatremia due to hyperlipidemia). In case of contracted intravascular volume but normal blood pressure, IV albumin infusion (20% or 25% to avoid fluid overload) should be administered over 4–6 h + / − furosemide if volemia is restored. Hypovolemic shock should be treated following specific resuscitation guidelines, starting with volume expansion by 20 mL/kg of 4% or 5% albumin over 20–30 min. ^a^Alternatively, isotonic saline can be used if 4% or 5% albumin is not readily available. *BP* blood pressure

Severe edema in SSNS may be associated with either intravascular volume contraction (hypovolemia, “underfilled patient”), maintained intravascular volume or hypervolemia (“overfilled patient”) [189–192]. All measures should be tailored according to the clinical assessment of the degree of edema and volume status (Fig. 4). Clinical indicators for intravascular volume contraction are peripheral vasoconstriction (prolonged capillary refill time), tachycardia, hypotension, oliguria, AKI, or reduced cardio-thoracic index on a chest X-ray. In contrast, hypertension would suggest an overfilled patient. Moderate edema is not harmful, but an inappropriate fluid restriction and/or use of diuretics may lead to AKI, hypovolemic shock and thromboses. Measurement of the fractional urinary excretion of sodium may be helpful in discriminating underfill vs. overfilled patients [193]. Fluid restriction is indicated in case of hyponatremia < 130 meq/L (after considering false hyponatremia due to hyperlipidemia [194]). When administering albumin infusions, we recommend careful monitoring of vital signs during and after albumin infusions, which can be complicated by pulmonary edema and high blood pressure.

Due to the risk of thrombosis and AKI in children with hypovolemia, we recommend not to administer diuretics in uncomplicated edema. If diuretics are required in severe edema, intravascular volume depletion should be excluded first, and diuretics should be used with caution and with careful monitoring of the volume status. Similarly, we recommend against ACEi or ARBs administration to control high blood pressure in SSNS.

The reported prevalence of hypertension in childhood SSNS is variable between 7 and 34% [195–200]. It occurs in children with SDNS and FRNS [200] and also in children in remission and/or 1–10 years off medication [196], especially in case of positive family history [195, 196]. The etiology is multifactorial and includes medication side effects, in particular gluco-corticoids and CNIs, and fluid overload due to inappropriate use of albumin infusion during relapses. The choice of antihypertensive agent in the acute nephrotic state and/or supportive measures (moderate fluid restriction and low salt-diet) should therefore be carefully adapted to the fluid status of the child. In children with chronic hypertension in remission, we refer to the current hypertension guidelines [201, 202].

### Prevention of thrombosis

- We recommend avoiding immobilization (grade X, strong recommendation), and intravascular volume contraction (grade C, moderate recommendation) during acute nephrotic episodes.
- We recommend counseling patients and families to make them aware of possible risk factors and of the symptoms of thromboembolic complications (grade X, moderate recommendation).
- We do not recommend routine prophylactic anticoagulation or antiplatelet treatment for children and adolescents in the acute nephrotic stage (grade C, weak recommendation).
- We suggest considering preventive anticoagulation during relapses in case of identified increased risks for thromboembolic complications (grade C, weak recommendation).
- We suggest that children with known familial thrombophilic predisposition and those with laboratory indicators suggesting possible familial predisposition be evaluated by a hematologist (grade D, weak recommendation).

#### Evidence and rationale

Children in the acute nephrotic state are at increased risk for venous and arterial thromboembolic events that disappears when the child achieves remission. The clinical spectrum includes cerebral venous thrombosis, deep venous thrombosis, pulmonary embolism, and arterial infarction but the majority of children have deep venous thromboses rather than arterial thromboses [203, 204]. The reported incidence of symptomatic thromboembolic events, mainly diagnosed within 3 months after disease onset [204], is about 3% in all forms of NS with peaks in infancy and adolescence (summarized in [205]) and is much lower than in adults (27%). The incidence is lower in children with SSNS (1.5%) than in complicated NS/SRNS (3.8%) [206]. Associated risk factors include the disease-related hypercoagulability, hypovolemia, immobilization, infections with hospitalization, indwelling central venous lines, and underlying hereditary thrombotic predisposition [204, 207, 208].

There is insufficient evidence to recommend routine prophylactic anticoagulation during the acute nephrotic state in children and adolescents. It is essential to assess the individual clinical risk profile of each child by taking a detailed history of previous thromboembolic events and hereditary predisposition, evaluating the volume status, and avoiding iatrogenic thrombotic risk factors. If preventive anticoagulation is needed, based on the individual clinical risk profile, we suggest using low-molecular weight heparin [209]. There are insufficient data to give recommendations on the use of antiplatelet treatment with aspirin in children with NS.

### Prevention and treatment of viral and bacterial infections

#### Antibiotics

- We suggest that antibiotic prophylaxis should not be given routinely to children with SSNS (grade C, weak recommendation).
- We recommend prompt antibiotic treatment in the case of a suspected bacterial infection (grade A, strong recommendation).
- We recommend treating peritonitis with IV antibiotics targeting *Streptococcus pneumoniae* (grade A, strong recommendation).
- We suggest giving cotrimoxazole prophylaxis to patients on RTX therapy during CD19^+^ B cell depletion, if receiving additional immunosuppressive co-medications (grade D, weak recommendation).

##### Evidence and rationale

Infections are a major concern in children with SSNS. These children are prone to infections not only during relapses because of urinary losses of IgG and complement opsonins (particularly encapsulated bacteria such as pneumococci), but also because of treatments (glucocorticoids or immunosuppressive agents) during remission. Thirty to 50% of infections are due to pneumococcal infection, with the rest due to gram-negative bacteria principally *Escherichia coli* [29, 210–214]. These infections may be severe and 60% of NS-associated deaths are due to infection [210]. However, prophylactic antibiotics are not indicated because they are not associated with a significant reduction in the occurrences of sepsis. Primary peritonitis is one of the most common major infections in hospitalized children with NS [215], with a reported incidence of 1.5–16% [211, 212, 216, 217] during relapses [218], or rarely occurring as the presenting feature of NS [219]. It may itself induce a relapse [220]. Immunosuppressive drugs and defects in humoral and non-specific immune mechanisms play a role [221, 222].

In patients with abdominal pain or discomfort and fever, diagnostic paracentesis with microbiological and biochemical analysis should be considered [211, 223, 224], especially in those with inadequate response to initial empirical antibiotic therapy. While waiting for the microbiological results of ascitic fluid, we recommend prompt treatment with IV antibiotics targeting *S*. *pneumoniae* such as cephalosporins or high doses of amoxicillin. IVIG in combination with parenteral antibiotics may be useful to treat septic episodes in children with low plasma IgG levels.

##### Peritonitis

There are no controlled trials on the use of penicillin prophylaxis to prevent peritonitis in children with NS [211].

##### Pneumocystis

Given the low incidence but high mortality of *Pneumocystis jirovecii* pneumonia and the drug side effects, we suggest giving cotrimoxazole prophylaxis in patients on RTX therapy during CD19^+^ B cell depletion if receiving additional immunosuppression [225]. Prophylactic cotrimoxazole dosing is recommended with 5–10 mg trimethoprim (TMP)/kg per day or 150 mg TMP/m^2^ per day in infants (at least 4 weeks of age) and children, given as single daily dose or in two divided doses every 12 h thrice weekly (on consecutive or alternate days) with a maximum TMP dose of 320 mg/day [226]. The oral dosing in adolescents is 80 to 160 mg TMP daily or 160 mg TMP 3 times per week [227]. A 50% dose reduction of cotrimoxazole is required when eGFR < 30 mL/min per 1.73 m^2^ and cotrimoxazole is not recommended when eGFR < 15 mL/min per 1.73 m^2^.

#### Immunoglobulin infusions

- We suggest considering preventive IVIG infusions in the case of persistent low plasma total IgG levels (e.g., related to RTX infusion) and recurrent and/or severe infections (grade D, weak recommendation).

##### Evidence and rationale

Children with SSNS can have extremely low levels of circulating IgG owing to urinary losses during relapses. The routine use of prophylactic intravenous immunoglobulins (IVIGs) is not indicated since levels quickly return to normal ranges after remission. However, preventive IVIG infusions may be considered in the case of low plasma total IgG levels and recurrent and/or severe infections, similar to the management of secondary hypogammaglobulinemia owing to causes other than SSNS [228]. For instance, we suggest considering prophylactic IgG substitution in case of RTX-induced hypogammaglobulinemia in patients presenting with recurrent and/or severe infections. Families of children on maintenance immunosuppression and low IgG levels should be counseled about the increased risks of infections, immediate medical evaluation in case of fever and consecutive prompt start of antibiotics in case of suspected bacterial infection and additionally IVIG in case of severe and/or bacterial infection [228].

#### Vaccinations

- We recommend reviewing the child’s vaccination status at disease onset and completing all inactivated vaccinations following the vaccination schedule that is recommended for healthy children without delay, especially for encapsulated bacteria (*pneumococcus*, *meningococcus*, *haemophilus influenzae*) (grade A, strong recommendation).
- We recommend administering inactivated influenza vaccine annually (grade A, strong recommendation).
- We recommend anti-COVID-19 vaccination in children with SSNS following the national recommendations (grade X, strong recommendation).
- We recommend following national vaccination guidelines for the administration of live attenuated vaccines in immunocompromised patients (grade A, strong recommendation).
- We do not recommend live vaccinations in patients on high-dose immunosuppression and in the first 6 months after RTX treatment (grade X, strong recommendation).
- We recommend vaccinating the household against influenza annually, against COVID-19 and with live vaccines if live vaccines are contraindicated in the child with SSNS (grade A, strong recommendation).

##### Evidence and rationale

Vaccination with inactivated vaccines should follow the recommended schedule for healthy children, including vaccinating against encapsulated bacteria (especially meningococcal, H. influenza, and pneumococcal). The risk of vaccine-induced relapses has been shown to be low in numerous studies [229–232]. We recommend annual vaccination against influenza [232–234].

Live vaccines should generally be avoided in immunocompromised children [235, 236]. However, the risk of live attenuated vaccine-induced infectious diseases in children with SSNS in relapse or who are receiving immunosuppressive drugs appears to be low in the literature and in pharmacovigilance databases. This includes children receiving low-dose PDN, possibly combined with immunosuppressive treatments provided that the immunological assessment is normal [237, 238]. Depending on the context and after specialized advice from infectious diseases specialists and/or immunologists, live attenuated vaccination may be considered in children with SSNS and immunosuppressive therapy if the doses/trough levels are low and immunological tests are normal [237].

Regarding the use of anti-CD20 monoclonal antibodies such as RTX which deplete antibody-producing cells, all efforts should be made to immunize children as fully as possible before administering these therapeutic agents, at least 1 month before infusion for live vaccines. Subsequently, vaccinations can be restarted 6–9 months following RTX, non-live vaccines before this timeframe if necessary [239]. Immunization titers may be affected by the use of these agents even many years post-infusion [240], therefore it may be prudent to verify vaccination titers in children who have received these monoclonal antibodies once B cells are reconstituted and they are in stable remission.

#### Varicella

- In case of exposure to chickenpox in children with immunosuppressive treatment who have not been immunized against VZV, we recommend prophylactic treatment with specific VZV IVIGs or oral acyclovir or valacyclovir for 5–7 days starting within 7–10 days of the exposure (grade A, strong recommendation).
- We suggest treatment of VZV infection with intravenous high-dose acyclovir for 7–10 days (grade C, weak recommendation).
- In the case of chickenpox, we suggest reducing doses of immunosuppressive drugs (grade D, weak recommendation).
- We recommend vaccinating non-immunized patients while in remission and not on high-dose immunosuppressive medications, as well as vaccinating non-immunized siblings and parents against VZV (grade A, strong recommendation).

##### Evidence and rationale

Varicella in an immunocompromised patient is a serious infection [241]. The severity of varicella in a PDN-treated patient depends on at least three factors, including the initial disease for which glucocorticoids were administered, the duration and dosage of PDN therapy, and the therapeutic manipulations of the clinician in managing the situation (e.g., abrupt discontinuation, increase or decrease of steroid dose) during various stages of varicella [242–244].

In case of exposure to chickenpox, we recommend treating susceptible patients (i.e., those with hypogammaglobulinemia who are not immunized against VZV and do not have a history of chickenpox) with VZV immunoglobulins (VZIGs) as soon as possible. This strategy may be effective for reducing the severity of chickenpox symptoms when VZIGs are given up to 10 days after exposure [245, 246]. If VZIGs are not available, we recommend prophylactic treatment with oral acyclovir (10 mg/kg four times a day for 7 days) within 7–10 days of exposure to chickenpox [19, 247, 248].

We recommend treatment of VZV infection with intravenous high-dose acyclovir (1500 mg/m^2^ per day in three doses) or oral acyclovir or valacyclovir for 7–10 days [244]. We suggest reducing immunosuppression in case of overt varicella infection, considering the risk of HPA axis suppression in case of abrupt reduction in steroid dosage.

#### COVID-19

- We recommend treating COVID-19 in children with SSNS as in the general pediatric population (grade X, strong recommendation).
- We suggest not reducing the immunosuppressive therapy in case of mild symptoms (grade C, weak recommendation).

##### Evidence and rationale

Children seem to have a lower incidence and milder clinical course of coronavirus disease 2019 (COVID-19) than adults [249, 250]. Immunosuppressive treatment does not seem to be a risk factor to develop COVID-19 in children and young adults with NS on immunosuppression, and most children with NS on immunosuppressive therapy who had COVID-19 experienced a mild disease course [251–253]. There is no evidence of any association between immunosuppressive medication number and the severity of COVID-19 in children.

### Preservation of bone health

- We recommend avoiding prolonged steroid exposure as a risk factor for osteopenia by administering the minimum effective dose, by changing to alternate-day therapy while in remission after relapses, by limiting the duration, and by considering steroid-sparing agents in case of emerging toxicity (grade X, strong recommendation).
- We recommend ensuring adequate dietary calcium intake in all children with SSNS and oral calcium supplementation in those with insufficient calcium intake (grade C, moderate  recommendation).
- We suggest assessing 25-OH-vitamin D levels annually in patients with SDNS or FRNS during the remission phase (after three months of remission, if possible) aiming for levels > 20 ng/mL (> 50 nmol/L) (grade C, weak recommendation).
- In case of vitamin D deficiency, we recommend following national treatment guidelines (grade A, strong recommendation).

#### Evidence and rationale

Conflicting data have been published on the risk of glucocorticoid-induced osteoporosis (GIO) in pediatric SSNS. Some studies reported low bone mineral density (BMD), correlating with disease severity and cumulative steroid intake [254–257]. In contrast, others have reported no change in BMD after initial, intermittent or long-term alternate-day therapy [258–262]. Children and adolescents with FRNS/SDNS seem to be at a higher risk of developing low BMD [263, 264]. In summary, bone mineral loss may occur early with high-dose daily PDN (which is usually given at start of therapy) but is less significant with subsequent intermittent or low-dose alternate-day regimens. The reported incidence of fracture is low (6–8%) [263, 264]. No data are available on the use of biphosphonates in children with NS. The prevention or limitation of GIO by minimizing steroid exposure to the lowest dose and shortest effective regimen is recommended. Nutritional and lifestyle measures to maintain bone strength should also be continued.

#### Calcium and vitamin D supplementation

Both the vitamin D-binding protein (VDBP) and albumin bound fractions of vitamin D are lost in urine in NS relapse, and several reports have documented low levels of total serum 25(OH)D in and after NS relapse [265–267]. The total serum 25(OH)D levels were shown to return to levels similar to healthy controls after 3 months of attaining remission by Banerjee et al. [268], whereas two other studies reported persistent low 25(OH)D levels at 3 months [267, 269]. In contrast, the biologically active fraction of free 25(OH)D levels were found to be similar to levels in healthy children both in remission and relapse of NS [270].

In patients with SSNS on steroid therapy, there are conflicting results about improvement of BMD when treated with vitamin D and calcium [271–274]. Calcium and vitamin D supplementation does not specifically treat GIO and there is insufficient evidence to recommend routine supplementation of vitamin D_3_ and oral calcium at onset of SSNS or during relapses of usually short duration. However, ensuring adequate calcium intake and normal 25(OH)D serum levels is suggested to optimise bone health. Vitamin D supplementation should be guided by serum levels, checked after remission of at least 3 months, and by national pediatric guidelines for vitamin D deficiency [275]. Excess supplementation has been associated with hypercalciuria [274, 276]. Note that higher 25(OH)D target levels are recommended in children with CKD stages 2–5D [277].

### Intermittent endocrine and metabolic changes during the acute nephrotic state

#### Hypothalamic–pituitary–adrenal axis suppression

- We recommend prevention measures for adrenal insufficiency including shortening the duration and lowering the dose of PDN as much as possible (grade X, strong recommendation).

##### Evidence and rationale

Supraphysiological and prolonged glucocorticoid therapy carries the risk of suppression of the hypothalamic–pituitary–adrenal axis with transient central adrenal insufficiency after abrupt withdrawal or discontinuation of glucocorticoid therapy. This risk is especially high during periods of stress such as febrile illnesses, surgery with general anaesthesia, or major trauma. Symptoms may include that of glucocorticoid deficiency but not of mineralocorticoid axis.

There are no relevant data available on the duration, frequency, and complications of transient adrenal insufficiency in childhood NS. Clinically apparent transient adrenal insufficiency seems to be a rare event. It was reported as suspected in only one child out of 775 patients included in 4 large RCTs evaluating steroid therapy for SSNS, presenting with transient fatigue and headache with spontaneous improvement.

The time required to achieve suppression depends upon the dose and varies among patients, likely due to differences in their rates of glucocorticoid metabolism. Risk factors for glucocorticoid-induced adrenal insufficiency include (1) daily steroid therapy for more than a few weeks, (2) evening/bedtime doses for more than a few weeks, and (3) any patient who has a Cushingoid appearance (also NS diagnosed before age 5 years and steroid dependence [278]. Children receiving daily PDN therapy for fewer than 3 weeks or on alternate-day PDN therapy are less likely to present adrenal insufficiency [279].

In at-risk children, the initial screening step in the laboratory diagnosis of adrenal insufficiency is measurement of serum cortisol in the early morning. Normal values depend on the patient age and assessment technique. If basal serum cortisol is low, adrenal insufficiency is likely. If the result is indeterminate (low-normal), then an early morning ACTH serum level or stimulation test is advisable to make a definitive diagnosis.

In case of confirmed adrenal insufficiency, patients should be referred to pediatric endocrinologists for a switch to hydrocortisone, patient information/education, and adrenal insufficiency card and emergency treatments. Hydrocortisone substitution in stress doses should be considered without delay in case of acute crisis especially when presenting with infections, fever, and/or acute symptoms of central adrenal insufficiency, which are more likely to occur in the first 8–12 weeks after end of PDN treatment. In case of acute adrenal crisis, emergency treatment with high-dose hydrocortisone, fluids and glucose is required.

Prevention measures for transient adrenal insufficiency include (1) shortening the duration and lowering the dose of PDN as much as possible, (2) in the case of prolonged use of PDN associated with steroid toxicity, slow tapering of PDN, and (3) informing patients and families of the risks and symptoms of adrenal insufficiency and crisis and of the emergency procedure in case of symptoms.

#### Transient abnormalities

- We do not recommend routine thyroid hormone substitution during SSNS relapses (grade D, weak recommendation).
- We do not recommend routine lipid-lowering agents during SSNS relapses (grade D, weak recommendation).

##### Evidence and rationale

Intermittent thyroid dysfunction can be observed during SSNS relapses due to urinary loss of albumin and thyroxine-binding proteins. Usually, thyroid hormone status normalizes with achieving remission and thyroxine replacement is not required.

Similarly, dyslipidemia occurs in SSNS during the initial episode and relapses but this abnormality usually resolves with remission of the NS. Therefore, treatment is not required unless these anomalies persist in remission. In case of prolonged nephrotic-range proteinuria, we recommend monitoring thyroid function and fasting lipids and referring to the recommendations for SRNS [19].

### Lifestyle and nutrition

- We recommend supporting regular physical activity in order to prevent thromboembolic events during relapses, weight gain on prednisolone treatment, and loss of muscle and bone mass (grade A, strong recommendation).
- We recommend healthy nutrition (avoiding high fat and/or high caloric food) while on steroids (grade A, strong recommendation).
- We recommend a low salt diet (suggested maximum dose of 2–3 meq/kg/day, 2000 mg/day in larger children) during relapse with moderate or severe edema, and normal salt intake while in remission (grade C, weak recommendation).
- We recommend a dietary protein intake as recommended for the general pediatric population (grade C, weak recommendation).
- When available, we suggest advice by a dietician to patients and families requiring suitable low salt and low fat foods during relapses (grade D, weak recommendation).

#### Evidence and rationale

Regular physical activity can prevent thrombosis and skeletal changes. Healthy nutrition is recommended and should be guided by a specialized dietician. Eating home-prepared meals using fresh ingredients instead of canned, frozen, or packaged meals is preferable, since the latter have a much higher salt content. As increased oral protein intake has not shown to improve serum albumin levels or patient outcomes, a regular oral protein intake is recommended [280].

### Sun protection

- We recommend using sun protection measures, especially in all children on maintenance immunosuppression with steroid-sparing agents (grade X, moderate recommendation).

#### Evidence and rationale

Sun protection as a general supportive measure is important in all children, especially in those on long-term immunosuppression. Measures include reducing exposure to UV radiation, avoiding sunbathing, covering the skin with adequate clothing, and using sun protection creams with high to very high sun protection factor.

## Childhood-adult transition

### Rate of transition, support of transition

- We recommend assessing the need for continued adulthood nephrology care in children with FRNS/SDNS at the age of 12–14 years, and at least 2–3 years before transition (grade X, moderate recommendation).
- We suggest regular assessment of the readiness of a patient for transition to adult care using standardized evaluation forms and questionnaires (grade D, weak recommendation).
- We suggest that the definitions and treatment advice for adolescents and young adults should be compatible with those for adults (grade D, weak recommendation).
- We suggest that a patient with childhood-onset SSNS transition to adult care when his/her medical condition is controlled on or off therapy and the patient and caregivers are prepared for transition (grade D, weak recommendation).
- We suggest that the decision regarding transition to primary care physician, local adult nephrology, or academic hospital care be based on the condition and history of the patient (grade D, weak recommendation).
- Upon transition, we recommend a complete review of the patient’s detailed medical history and proper transfer of all relevant information (grade X, moderate recommendation).

#### Evidence and rationale

While children are less likely to relapse as they grow older [281], more than 10% (6.8–42.2%) of childhood-onset SSNS patients still experience relapses during adulthood [6, 7, 282–286]. Risk factors of continued active disease during adulthood are earlier onset of NS [129, 282, 285], early relapse after onset [6, 287], FRNS or SDNS [6, 7, 284–287], and duration of remission < 6 years [283, 288]. Accordingly, some adolescents are still using maintenance immunosuppressive therapy [285, 289] (Supplementary Table S10). Many also have experienced comorbidity from the treatment or the disease, such as hypertension, short stature, obesity, osteoporosis, cataract, dyslipidemia, infertility, and even psychiatric illness and thrombosis [6, 285, 287, 289–292]. These conditions need to be cared for without interruption, necessitating appropriate transition when the patient becomes an adult. Since a long time may be required for patients and their caregivers to prepare for transition to adult care, plans for transition should be started when the patient becomes an adolescent.

Transition is defined as a “process that involves planned efforts to prepare the patient from caregiver-directed care to self-disease management in the adult unit” according to the consensus statement on transition endorsed by ISN and IPNA [293]. For a successful transition, a young adult should be competent in self-disease management, which can be evaluated by questionnaires such as the Ready Steady Go and the Transition scale. Examples are provided in Supplementary Tables S13 and S14. Risk of nonadherence at the time of transfer from pediatric to adult care is high [294, 295] which can be aggravated if treatment policy of adult care is different from that of pediatric care. Because disease definitions, treatment protocols, and monitoring and follow-up differ between adults and children [296–298] (Supplementary Table S15), the patient should be educated and made aware of these differences during the period of transition to ensure adaptation and adherence to adult care.

Upon transition, a decision should be made about whether to transfer the patient to a primary care physician, local adult nephrology practice, or an academic hospital center, based on the condition and history of the patient. If the patient is prepared for transition, in remission for a long period without any immunosuppressive therapy, without additional support of other members of the multidisciplinary team (psychologist, social workers, educators), and his/her kidney function and blood pressure are normal, he/she can be referred to primary care with instructions about management, health-care checks, and when to consult hospital physicians. Otherwise, the patient should be prepared for transition to adult nephrology care. Patients who require low-complexity care can be transitioned to a nephrologist in a regional center, when the treatment plan is defined and the clinical condition of the patient is stable. When in doubt, we suggest that patients be transitioned to a nephrologist in an academic center, who can decide to share management with his/her colleague in a regional center.

#### Evaluation on transition

For uninterrupted care, the adult nephrologist needs to know the patient thoroughly by comprehensive history-taking and evaluation (Table 6).

**Table 6:** Patient evaluation form to assist transition care

Category		To evaluate
Medical history	Disease characteristics	Age of onset, FRNS or SDNS, number of relapses, last relapse date, time of response to PDN
	Medication history	Dosage of PDN for remission induction, current medication, cumulative dosage of PDN, CNIs, cytotoxic agents, cytostatic agents, anti-CD20s, other biologics
	Complications of the disease	History of AKI, thrombosis
	Side effects of medications	Multiple; e.g., skin, growth, infections, mental problems
	Kidney biopsy	Date of biopsy, review of biopsy report; discuss with pathologist if in doubt
Physical examination	Blood pressure Anthropometry Body mass index General physical exam	Hypertension, growth failure, obesity, striae, skin problems, gum hypertrophy, hirsutism, hair loss/alopecia
Laboratory evaluation	Blood chemistry	Kidney function impairment
	Blood lipid	Dyslipidemia
	Blood cell count	Neutropenia
	IgG (antiCD20mAb user)	Hypogammaglobulinemia
	Blood glucose, Hb A1c	Diabetes mellitus
	ANCA (levamisole user)	Vasculitis
Radiologic evaluation	Consider DEXA in patients with low muscle mass, frail or low intensity fractures	Osteopenia/osteoporosis
Consultation; when indicated from medical history	Ophthalmologic evaluation	Cataract, glaucoma
	Cardiologic evaluation	Pulmonary hypertension, venous insufficiency (thrombosis history)
Social & other considerations	Education/occupation/lifestyle Quality of Life Ongoing support by psychologists, social workers etc Knowledge of self-management	Friends, partners, menstrual cycle Planned parenthood

### Implementation of supportive programs of transition

- We suggest that supportive programs of transition be implemented for childhood-onset SSNS patients (grade D, week recommendation).

#### Evidence and rationale

There are few data regarding transition care focusing on patients with SSNS [299]. Considering that quite a number of patients with childhood-onset NS persistently relapse during adulthood, a formal supportive program of transition is required.

#### Requirements for transition care

It is advised that the patient is seen jointly by the pediatric and adult nephrologist during one or more outpatient visits. A detailed history should be transferred, which should include various aspects of the disease history as listed in Table 6. Ideally, a specialized nurse or case manager is involved in transition. This person can be the person who is primarily the key liaison for the patient.

#### Patient education

While children are instructed to check their urine regularly, and to increase drug dose in case of a positive test, relapse during adulthood is usually not as frequent as during childhood, and the relapse rate decreases with age. Many patients may have low-grade proteinuria, or develop short-lasting proteinuria during fever, infections, or exercise. In addition, the risk of severe morbidity caused by a relapse, such as hypovolemia or thrombo-embolic events is low in adults. Therefore, patients need to be educated to rely on their own observation of signs and symptoms such as foamy urine, edema, abdominal pain, instead of relying on dipstick tests to detect a relapse, which accompanies urinary change (foamy urine) and edema at later stage. However, dipstick evaluations are recommended in any case of clinically suspected relapse.

#### Management strategy

There should be a discussion on overall management, including how to monitor and manage relapse and how to modify maintenance immunosuppression. Although many patients will experience a relapse, tapering of immunosuppressive therapy should be tried at least every 2 years, although it remains a matter of trial and error. In addition, it is important to discuss the strategy to prevent relapses during infections or stress. Likewise, information on prevention of glucocorticoid deficiency should be available and clear.

## Supplementary Information

Below is the link to the electronic supplementary material.

Supplementary file1 (PDF 1.95 MB)

Supplementary file2 (PPTX 121059 KB)
