# Supplementary material for: IPNA clinical practice recommendations for the diagnosis and management of children with steroid-sensitive nephrotic syndrome
Source: Pediatr Nephrol. 2022 Oct 21;38(3):877–919. doi: 10.1007/s00467-022-05739-3 (PMC9589698; doi:10.1007/s00467-022-05739-3)
Supplement: Supplementary file 1 — Supplementary file1 (PDF 1.95 MB) [file 467_2022_5739_MOESM1_ESM.pdf]

IPNA Clinical Practice Recommendations for the Diagnosis and Management of Children with Steroid Sensitive Nephrotic Syndrome

on behalf of the International Pediatric Nephrology Association

SUPPLEMENTARY MATERIAL:

CONTENT:

Table S1 Area or expertise and responsibilities of core group members

Table S2 Keywords for SSNS used for literature search in PubMed database

Table S3 Randomized controlled trials (RCTs) evaluating PDN therapy in SSNS
- **Table S3.1** Duration of PDN therapy for first episode of SSNS: ≥ 3 months *vs.* 2 months
- **Table S3.2** Duration of PDN in the first episode of SSNS: Five to 7 months *vs.* 3 months
- **Table S3.3** Other relevant regimens of PDN used in SSNS

Table S4 Adverse effects of corticosteroids in children with SSNS

Table S5 Studies evaluating daily PDN to prevent relapse with upper respiratory tract infection (URTI)
- **Table S5.1/ Figure S1** Number of children with relapse associated with URTI: low dose daily PDN given at the onset of URTI compared with placebo
- **Table S5.2/ Figure S2** Number of infection-related relapses per patient per year
- **Table S5.3/ Figure S3** The mean number of relapses/patient during two year follow up

Table S6 GRADE-based evidence for the different steroid-sparing agents used in children with FRNS/SDNS

Table S7 SSNS Randomized controlled trials – Steroid-sparing and immunomodulatory agents in FRNS and SDNS
- **Table S7.1** Outcome/ Relapses
- **Table S7.2** Adverse Events

Table S8 SSNS observational studies – Steroid-sparing and immunomodulatory agents in FRNS and SDNS
- **Table S8.1** Calcineurin-Inhibitors (CNI): Cyclosporin A (CsA) and Tacrolimus (TAC)
- **Table S8.2** Alkylating Agents: Cyclophosphamide (CPA) and Chlorambucil (CHL)
- **Table S8.3** Mycophenolate mofetil (MMF)/ Mycophenolate Sodium (MPS)
- **Table S8.4** Levamisol (LEV)
- **Table S8.5** Rituximab (RTX)
- **Table S8.6** Mizoribine (MZR)
- **Table S8.7** Other Agents (Vincristine, Saquinavir, ACTH, Azathioprine)

Table S9 Adverse effects and impact of alkylating agents on (peri)-pubertal children

Table S10 Studies of long-term outcome of childhood-onset SSNS

Table S11 Semiquantitative expression of typical dipstick results

Table S12 Future research recommendations

Table S13 Competencies expected in a young adult at the time of transition

Table S14 The Transition scale, a tool to monitor the progression in transition competence

Table S15 Definition differences between children and adults

Alternatives to dipstick: Unfortunately, this file includes a video and is therefore too large to be inserted in this word doc but will be available for the reader on the Springer website.

Table S1: Area or expertise and responsibilities of core group members

Name	Area of expertise	Responsibilities
Al Hasan, Khalid	Pediatric nephrology	Drafting of recommendations and evidence text and grading of recommendations and reviewing the manuscript before submission
Bagga, Arvind	Pediatric nephrology<br>Guideline development	Drafting of recommendations and evidence text and grading of recommendations and reviewing the manuscript before submission
Banerjee, Sushmita	Pediatric nephrology	Drafting of recommendations and evidence text and grading of recommendations and reviewing the manuscript before submission
Bhimma, Rajendra	Pediatric nephrology	Drafting of recommendations and evidence text and grading of recommendations and reviewing the manuscript before submission
Bonilla-Felix, Melvin	Pediatric nephrology	Drafting of recommendations and evidence text and grading of recommendations and reviewing the manuscript before submission
Boyer, Olivia	Pediatric nephrology<br>Guideline development	Co-coordinator of this project. Drafting of recommendations and evidence text and grading of recommendations. Incorporation of suggestions from the core group members, external experts and voting group members into the manuscript and reviewing the manuscript before submission
Cano, Francisco	Pediatric nephrology<br>Guideline development	Drafting of recommendations and evidence text and grading of recommendations and reviewing the manuscript before submission
Christian, Martin	Pediatric nephrology	Drafting of recommendations and evidence text and grading of recommendations and reviewing the manuscript before submission
Cook, Wendy	Patient representative	Drafting of recommendations and evidence text and grading of recommendations and reviewing the manuscript before submission

Name	Area of expertise	Responsibilities
Gipson, Debbie	Pediatric nephrology<br>Guideline development	Drafting of recommendations and evidence text and grading of recommendations and reviewing the manuscript before submission
Hahn, Deirdre	Pediatric nephrology	Drafting of recommendations and evidence text and grading of recommendations and reviewing the manuscript before submission.
Haffner, Dieter	Pediatric nephrology<br>Guideline development	Leading coordinator of this project. Coordination of work groups and the process of generating the manuscript. Drafting of recommendations and evidence text and grading of recommendations, incorporation of suggestions from the core group members, external experts and voting group members into the manuscript and reviewing the manuscript before submission
Hodson, Elisabeth	Pediatric nephrology<br>Guideline development<br>Epidemiology	Literature search and creation of evidence tables and meta-analysis. Drafting of recommendations and evidence text and grading of recommendations. Incorporation of suggestions from the core group members, external experts and voting group members into the manuscrip and reviewing the manuscript before submission. Liaison to Cochrane Kidney and Transplant.
Kang, Hee Gyung	Pediatric Nephrology	Drafting of recommendations and evidence text and grading of recommendations and reviewing the manuscript before submission
Nakanishi, Koichi	Pediatric nephrology<br>Guideline development	Drafting of recommendations and evidence text and grading of recommendations and reviewing the manuscript before submission
Safouh, Hesham	Pediatric nephrology<br>Guideline development	Drafting of recommendations and evidence text and grading of recommendations and reviewing the manuscript before submission

Name	Area of expertise	Responsibilities
Samuel, Susan	Pediatric nephrology<br>Guideline development<br>Epidemiology	Drafting of recommendations and evidence text and grading of recommendations and reviewing the manuscript before submission. Creation of evidence tables
Trachtman, Howard	Pediatric nephrology	Drafting of recommendations and evidence text and grading of recommendations and reviewing the manuscript before submission
Trautmann, Agnes	Pediatric nephrology<br>Guideline development<br>Epidemiology	Literature search and creation of evidence tables, drafting of recommendations and evidence text and grading of recommendations. Organization of Delphi process. Incorporation of suggestions from the core group members, external experts and voting group members into the manuscript and reviewing the manuscript before submission
Vivarelli, Marina	Pediatric nephrology<br>Guideline development	Drafting of recommendations and evidence text and grading of recommendations. Incorporation of suggestions from the core group members, external experts and voting group members into the manuscript and reviewing the manuscript before submission. Creation of evidence tables
Wetzels, Jack	Adult nephrology<br>Transition<br>Guideline development	Drafting of recommendations and evidence text and grading of recommendations and reviewing the manuscript before submission
Xu, Hong	Pediatric nephrology<br>Guideline development	Drafting of recommendations and evidence text and grading of recommendations and reviewing the manuscript before submission

Table S2: Keywords for SSNS

Nephrotic syndrome	Nephrotic syndrome (NS)<br>Idiopathic nephrotic syndrome (iNS)<br>Primary SSNS<br>Secondary forms of nephrotic syndrome (systemic disease…)
Steroid response	Steroid sensitive nephrotic syndrome (SSNS)<br>Steroid resistant nephrotic syndrome (SRNS)<br>Remission (complete/ partial)<br>Response to treatment (initial responder, initial non-responder, late responders)
Relapse	Relapse of nephrotic syndrome<br>Definition<br>Triggers of relapses
Disease courses	Infrequent relapsing nephrotic syndrome<br>Frequent relapsing nephrotic syndrome (FRNS)<br>Steroid dependent nephrotic syndrome (SDNS)<br>Secondary steroid-resistant nephrotic syndrome
Primary treatment/ Initial treatment	Steroid regimens<br>Glucocorticoids<br>Corticosteroids<br>Prednisone<br>Prednisolone<br>Administration (orally/ IV)<br>Duration<br>Dosage<br>Frequency (daily/ alternate daily)<br>Mycophenolate mofetil (MMF)<br>Mycophenolic acid<br>Pulse steroids – IV methylprednisolone<br>Complications of treatment: Steroid toxicity
Treatment of relapses<br><br><br><br>Prevention of relapses	Glucocorticoids<br>Corticosteroids<br>Prednisone/ prednisolone<br>Low-dose alternate-day steroid regimes<br>Increase of steroids during intercurrent illnesses<br>Steroid-sparing agents
Corticosteroid sparing treatments in FRNS/SDNS (“maintenance treatment”, prevention of relapses)	Indications<br>Steroid-sparing agents<br>Non-corticosteroid treatment<br>MMF<br>Levamisole<br>Calcineurin-Inhibitors (CNI)<br>Cyclosporin A (CsA)<br>Tacrolimus (Tac)<br>Rituximab<br>Alkylating agents<br>Cyclophosphamide<br>Chlorambucil<br>Ofatumumab<br>Mizoribine<br>Azithromycin

	ACTH<br>Fusidic acid<br>I.V. Immunoglobulins
Drug monitoring	Side effects<br>Pharmacokinetics<br>MPA-AUC<br>Trough levels<br>Pharmacogenetics
Clinical evaluation	Edema<br>Weight increase<br>Skin<br>Extra-renal symptoms<br>Age at onset<br>Trigger events (infection, insect bites, allergic episodes)
Etiology, pathophysiology	Immune-mediated disease<br>Immune dysregulation<br>Dysfunction/ dysregulation of T-lymphocytes<br>B-T cell cross-talk<br>Switched memory B cells<br>B cell subpopulations<br>EBV<br>IgM<br><br>Immunoglobulin<br>Systemic circulating factor disease<br>Circulating glomerular permeability factor<br><br>Trigger events (e.g. infections, allergic reactions)<br><br>Underlying glomerular pathology<br><br>Genetic risk<br>Genetic locus on chromosome 6p<br>SNP in HLA-DQA1<br>HLA-DQB1<br>Genome wide association studies
Biopsy/ Histopathology	Indications for biopsy<br>Age<br>Minimal change nephrotic syndrome (MCNS)/ minimal change disease/ glomerulopathy<br>IgA nephropathy<br>FSGS<br>Membranous nephropathy<br>Membranoproliferative GN (MPGN)<br>Mesangioproliferative GN (MesPGN)<br>Systemic diseases (e.g. SLE)
Complications of nephrotic syndrome - acute	Hypoalbuminemia<br>Edema<br>Generalized edema, Anasarca<br>Refractory edema<br>Effusions (pleural, pericardial)<br>Pulmonary edema<br>Umbilical/ inguinal hernia<br>Hypervolemia<br>Elevated blood pressure<br>Diarrhea<br>Invagination

	Acute kidney injury (prerenal)<br>Intravascular hypovolemia<br>Hypotension<br>Hypovolemic dysregulation/ shock<br>Electrolyte imbalances<br>Impaired glucose tolerance<br>Hemoconcentration<br>Hypercoagulability<br>Thrombosis<br>Infections<br>Increased risks of infections (viral, bacterial)<br>Peritonitis<br>Cellulitis<br>Septicemia<br>Meningitis<br>Pneumonia<br>VZV<br>Hyper-/dyslipidemia<br>Anemia<br>Vitamin D deficiency
Chronic complications of nephrotic syndrome and treatment-related	Steroid toxicity<br>Immunosuppression<br>Increased susceptibility to infection<br>Hypogammaglobulinemia<br>Osteopenia<br>Ophthalmologic complications<br>Cataract<br>Glaucoma<br>Growth impairment<br>Excessive weight gain/ obesity<br>Cushingoid features<br>Diabetes mellitus<br>Adrenal insufficiency<br>Arterial hypertension<br>Increased cardiovascular risk<br>Behavior disturbances (hyperactivity, depression, psychosis)
Supportive and preventive management	Fluid restriction<br>Salt restriction<br>Fluid balancing<br>Fluid management<br>Underfill/ overfill hypothesis<br>Diuretics<br>Furosemide<br><br>Thiazide diuretics<br>Aldosterone antagonists<br>Spironolactone<br>Albumin infusion<br>Evaluation of FENa (fractional excretion of sodium)<br>Vitamin D supplementation<br>Gastroprotection<br><br>Electrolyte imbalances<br>Hypocalcemia<br><br>Antihypertensive treatment<br>Calcium antagonists<br>Amlodipine<br>RAAS<br>ACE-inhibitor (ACEi)

	Angiotensin-receptor-blocker (ARB)<br><br>Prophylactic anticoagulation<br>Patient mobilization<br>Avoidance of immobilization<br>Heparin<br>Aspirin<br><br>Infection prophylaxis<br>Antibiotic prophylaxis<br>Penicillin<br>Hypogammaglobulinemia<br>Immunoglobulin substitution<br>Vaccination<br>VZV-IgG<br>Aciclovir<br><br>Hyperlipidemia<br>Life-style<br><br>School attendance<br><br>Diet therapy<br>Caloric intake<br>Protein intake<br><br>Exercise<br><br>Evaluation of bone mineral density (BMD)<br>Biphosphonates
Outcome	Long-term outcome<br>Renal function<br>Renal survival<br><br>Hospitalization<br><br>Quality of life<br>Patient-reported outcomes<br><br>Malignancies
Transition	Transition

Table S3: Randomized controlled trials (RCTs) evaluating prednisone in SSNS
Based on: Hahn et al. [1]

Table S3.1 Duration of prednisone (PDN) therapy for first episode of SSNS: ≥ three months versus 2 months

Outcome	N. studies	Total Participants	Intervention N events/N participants	Comparator N events/N participants	RR	95% CI	I^2
**RELAPSE/ FRNS**							
N with FRNS at 12-24 mths	8	976	186/469	228/507	0.86	0.71 - 1.06	33%
N with relapse at 12-24 mths	12	1309	346/648	427/661	0.77	0.63 - 0.95	77%
N with FRNS (low risk of bias)	*5*	*756*	*172/375*	*183/381*	*0.96*	*0.83 - 1.10*	*0%*
N with FRNS (unclear or high risk of bias)	*3*	*220*	*14/94*	*45/126*	*0.45*	*0.26 - 0.77*	*0%*
**ADVERSE EFFECTS**							
Psychological disorders	4	456	98/237	103/219	1	0.53 - 1.90	26%
Hypertension	7	548	25/268	14/280	1.78	0.55 – 5.73	50%
Eye complications	6	623	3/307	10/316	0.41	0.11 – 1.52	0%
Short stature	4	354	9/176	20/178	0.54	0.25 – 1.18	0%
Cushingoid features	5	547	124/288	104/259	1.12	0.76 – 1.65	46%
Infections	2	172	33/101	29/71	0.79	0.53 – 1.17	0%
Osteoporosis	3	233	1/123	5/110	0.47	0.06 – 3.36	0%

RR, Relative risk, 95% CI, 95% confidence intervals, I^2, measure of heterogeneity between studies (< 40%, not important, 30-60% moderate; >60% substantial). The section in italics demonstrates the difference in results between studies at low risk of bias for selection bias and those at unclear or high risk of bias

Table S3.2 Duration of PDN in the first episode of SSNS: Five to seven months versus three months

Outcome	N. studies	Total Participants	Intervention N events/N participants	Comparator N events/N participants	RR	95% CI	I^2
**RELAPSE/FRNS**							
N with FRNS at 12-24 mths	6	706	109/357	135/349	0.73	0.49 – 1.09	68%
N with relapse at 12-24 mths	7	762	182/387	261/375	0.62	0.45 – 0.85	83%
N with FRNS (low risk of bias)	3	376	84/192	81/184	0.99	0.74 – 1.33	35%
N with FRNS (unclear or high risk of bias)	3	330	25/165	54/165	0.48	0.32 – 0.72	0%
**ADVERSE EFFECTS**							
Psychological disorders	4	505	4/258	13/247	0.30	0.05 – 1.83	46%
Hypertension	6	752	53/380	47/372	1.11	0.71 – 1.74	27%
Eye complications	5	614	5/308	11/306	0.46	0.18 – 1.17	0%
Short stature	3	436	24/222	32/214	0.73	0.36 – 1.48	40%
Cushingoid features	6	762	131/386	141/376	0.86	0.60 – 1.23	70%
Infections	5	702	64/356	64/346	0.98	0.65 – 1.46	33%

RR, Relative risk, 95% CI, 95% confidence intervals, I^2, measure of heterogeneity between studies (< 40%, not important, 30-60% moderate; >60% substantial). The section in italics demonstrates the difference in results between studies at low risk of bias for selection bias and those at unclear or high risk of bias

Table S3.3 Other relevant regimens of PDN used in SSNS

Study	Population	Intervention Steroids	Comparator	Outcome	N. studies	Total participants	RR/MD	95% CI	Conclusions
APN 1988 [2]	SSNS<br>1st episode	Two months	One month	N. with relapse at 12-24 mths	1	61	RR 1.46	1.01 – 2.12	Two months more effective than one month
Ekka 1997 [3]<br>Li 1994 [4]	SSNS<br>**relapse**	Single daily dose	Divided daily doses	Days to remission	2	138	MD 0.04	-0.98 to +1.06	Single daily dose as effective as divided doses
Borovitz 2020 [5]<br>Sheikh 2019 [6]	SSNS<br>**relapse**	Reduced dose<br>1 mg/kg	Standard dose<br>2 mg/kg	Days to remission	2	79	MD 0.71	-0.43 to + 1.86	Reduced dose as effective as standard 2 mg/kg dose (small studies)
Borovitz 2020 [5]<br>Kansal 2019 [7]	SSNS<br>**relapse**	Reduced dose	Standard dose	N. with relapse	2	59	RR 0.66	0.16 – 2.68	Reduced dose as effective as standard dose (small studies)
Yadav 2019 [8]	SSNS<br>FRNS	Daily dose	Alternate day dose	Number of relapses/yea r	1	62	MD - 0.90	-1.33 to - 0.47	Daily dose more effective than alternate day dose
Raman 2016 [9]<br>Basu 2020 [10]	SSNS<br>**Initial & relapse**	Dose in mg/kg	Dose in $mg/1.73m^2$	Relapse at 6 mths	2	146	RR 1.03	0.71 to 1.49	No difference between regimens in relapse or adverse effects
Abeygunawardena 2008 [11]<br>Abeygunawardena 2014 [11]<br>Gulati 2011 [12]<br>Mattoo 2000 [13]	SSNS<br>**relapsing with URTI**	Daily dose during URTI	Alternate day dose in URTI	Number with relapse	4	219	Different analyse s used in each study		Reduced risk of relapse with daily doses of prednisone compared with alternate day prednisone in all studies
PREDNOS 2 2021 [14]	SSNS<br>**relapsing with URTI**	Daily dose during URTI	Placebo	1st URTI-related relapse; number of overall relapses	1	365	RD, −0.024	−0.142 to 0.095	No reduction of the risk of URTI-related relapse with daily doses of prednisone

PROPINE Study 2020 (Gargiulo 2020) [15]	SSNS in **relapse**	From day 5 after remission of relapse: Stable dose (40 mg/m^2) given on 18 alternate days over 36 days. (N=38)	From day 5 after remission of relapse: Tapering dose over 72 days. Taper after each group of 6 doses of alternate day steroid. (N=40)	Number with relapse at 6 months	1	78	RR 0.73	0.46 to 1.16	No difference in risk of relapse between stable dose of alternate day prednisone (40 mg/m^2) for 36 days (relapse rate 42%) and tapering dose over 72 days (relapse rate 58%) with same cumulative total prednisone dose
Kainth 2021 [16]	SSNS in **relapse**	After achieving remission of a relapse: "Short regimen" (40 mg/m^2 on alternate days for 2 weeks) (N=55)	After achieving remission of a relapse: "Standard regimen" (40 mg/m^2 on alternate days for 4 weeks) (N=62)	% of children with FRNS or SDNS at 12 months	1	117	HR 1.01	0.83 to 1.23 (p=0.98)	No difference in % of children developing FRNS or SDNS at 12 months between "short regimen" and "standard regimen" (23% vs. 24%, risk difference –1%, 95% CI –15% to 16%, p=0.9). Similar in both groups: time to relapse, relapse rate, steroid-related adverse events at 12 months.<br><br>Cumulative steroid exposure significantly lower in "short regimen".

RR, Relative risk, 95% CI, 95% confidence intervals. MD, mean difference. RD, risk differenz

Table S4 Adverse effects of corticosteroids in children with SSNS

Adverse effects of corticosteroids were recorded up to 12-24 months following PDN treatment for the first episode of SSNS. The duration of initial treatment in the high dose group ranged from three to seven months. The duration of initial treatment in the lower dose group ranged from two to three months. The data were obtained from randomised controlled trials published between 1993 and 2020 with outcomes reported at 12-24 months after initiation of treatment. Data from [1].

**Adverse effects**	**No. of trials**	**No. of . patients**	**% harm in high dose group**	**% harm in lower dose group**	**Risk difference (95% confidence intervals)**
Hypertension	14	1475	12.6	9.5	0.03 (-0.01 to 0.07)
Ophthalmological disorders	11	1237	1.3	3.4	0.00 (-0.02 to 0.01)
Growth retardation	7	790	8.3	3.2	-0.03 (-0.06 to 0.02)
Psychological disorders	8	962	20.6	24.9	-0.03 (-0.07 to 0.01)
Cushingoid appearance	11	1309	40.9	38.6	0.01 (-0.07 to 0.10)
Infections	7	874	21.2	22.3	-0.02 (-0.08 to 0.04)
Osteoporosis	3	233	0.81	4.5	-0.02 (-0.09 to 0.05)

Table S5 Studies evaluating daily PDN to prevent relapse with upper respiratory tract infection (URTI)

Five RCTs evaluated whether low dose daily PDN given for 5-7 days at the onset of URTI reduced the risk of relapse in children with SSNS. Studies presented their data in different formats limiting the opportunities for combining data in meta-analyses.

Table S5.1/ Figure S1: Number of children with relapse associated with URTI: low dose daily PDN given at the onset of URTI compared with placebo.

Study or Subgroup	Prednisolone Events	Prednisolone Total	Placebo Events	Placebo Total	Risk Ratio M-H, Random, 95% CI
**7.1.1 Abeygunawardena 2008 participants receiving daily PDN compared with alt day PDN + placebo**					
Abeyagunawardena 2008	4	18	10	22	0.49 [0.18, 1.30]
**7.1.2 PREDNOS 2 all participants**					
PREDNOS 2 2021	56	134	58	136	0.98 [0.74, 1.30]
**7.1.3 PREDNOS 2 participants receiving long term alt day PDN**					
PREDNOS 2 2021	16	39	15	31	0.85 [0.50, 1.43]
**7.1.4 PREDNOS 2 participants receiving long term alt day PDN + other immunosuppressive agents**					
PREDNOS 2 2021	14	41	19	48	0.86 [0.50, 1.50]

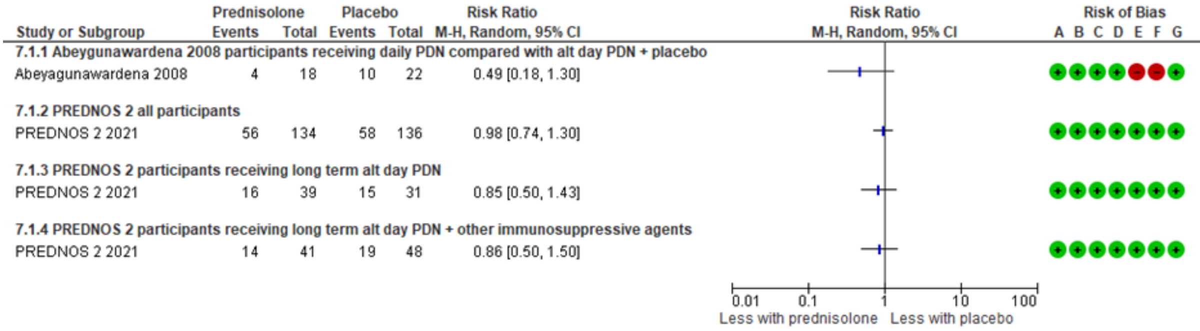

Risk of bias legend
(A) Random sequence generation (selection bias)
(B) Allocation concealment (selection bias)
(C) Blinding of participants and personnel (performance bias)
(D) Blinding of outcome assessment (detection bias)
(E) Incomplete outcome data (attrition bias)
(F) Selective reporting (reporting bias)
(G) Other bias

Figure S1 shows a forest plot of the number of children with SSNS and with URTI-related relapses in those receiving low-dose daily PDN for 5-7 days compared with those receiving placebo using data from two RCTs (15 mg per m^2 BSA which is equivalent to 0.5 mg/kg in PREDNOS 2, and 0.36 mg/kg for Abeyagunawardena 2008) [11, 14]. The data indicate that daily low-dose PDN did not reduce the risk of relapse with URTI in children with SSNS since the 95% confidence for each point estimate cross 1. The risk of bias attributes are listed below the forest plot and are shown to the left of the figure with green indicating low risk of bias and red indicating high risk of bias. PREDNOS 2 [14] is at low risk of bias for all attributes while Abeyagunawardena 2008 [11] is at high risk of bias for incomplete outcome data and selective reporting of outcomes. Abeyagunawardena 2008 [11] was also inadequately powered to demonstrate a difference between interventions as shown by the wide confidence intervals around the point estimate (relative risk 0.49, 95% confidence intervals 0.18 to 1.30).

Table S5.2/ Figure S2: Number of infection-related relapses per patient per year

Study or Subgroup	Daily PDN during URTI Mean	SD	Total	Alt day PDN during URTI Mean	SD	Total	Mean Difference IV, Random, 95% CI
**7.2.1 Number of infection-related relapses/patient/year in all children**							
Gulati 2011	0.7	0.3	50	1.4	0.5	50	-0.70 [-0.86, -0.54]
**7.2.2 Number of infection-related relapses/patient/year in children on PDN alone**							
Gulati 2011	0.6	0.2	35	1.5	0.4	33	-0.90 [-1.05, -0.75]
**7.2.3 Number of infection-related relapses/patient/yr in children on PDN + LEV**							
Gulati 2011	0.7	0.2	15	1.1	0.4	17	-0.40 [-0.62, -0.18]

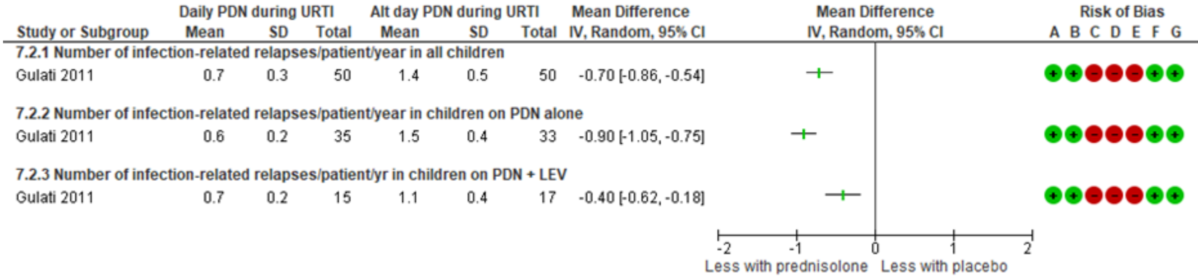

Risk of bias legend
(A) Random sequence generation (selection bias)
(B) Allocation concealment (selection bias)
(C) Blinding of participants and personnel (performance bias)
(D) Blinding of outcome assessment (detection bias)
(E) Incomplete outcome data (attrition bias)
(F) Selective reporting (reporting bias)
(G) Other bias

Figure S2 shows a forest plot of a single RCT [12], which evaluated the number of infection related relapses/patient/year in children given low dose daily PDN (0.6 mg/kg) for 7 days at the onset of infection compared with those continuing on alternate day PDN. In the experimental group, participants had their existing PDN alternate-day dose increased to a daily dose for 7 days at onset of viral infection. In all children and in subgroups, receiving PDN alone or PDN and levamisole, the number of infection related relapses/patient/year were reduced in children receiving daily PDN. The study was at high risk of bias for lack of blinding of participants, personnel and outcome assessment and for incomplete reporting of outcome data.

Table S5.3/ Figure S3: The mean number of relapses/patient during two year follow up

Study or Subgroup	Daily prednisone Mean	SD	Total	Alternate day prednisone Mean	SD	Total	Mean Difference IV, Random, 95% CI
Mattoo 2000	2.2	0.87	18	5.5	1.33	18	-3.30 [-4.03, -2.57]

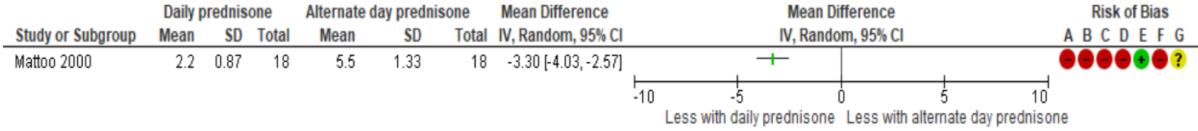

Risk of bias legend
(A) Random sequence generation (selection bias)
(B) Allocation concealment (selection bias)
(C) Blinding of participants and personnel (performance bias)
(D) Blinding of outcome assessment (detection bias)
(E) Incomplete outcome data (attrition bias)
(F) Selective reporting (reporting bias)
(G) Other bias

Figure S3 shows a single small RCT (n=36) [13] comparing low dose daily PDN (0.5 mg/kg) for 5 days at the onset of URTI with continuing alternate day PDN (0.5 mg/kg). It reported data on the mean number of relapses/patient during the 2 year follow up and did not separate relapses due to URTI from other relapses. Low dose daily PDN reduced the risk of relapse compared with continuing alternate day PDN. The study was at high risk of selection, performance and detection bias.

A fifth study [17] reported data from a cross-over study. Twenty seven children not receiving alternate day PDN were randomised to receive low-dose daily PDN (0.5 mg/kg) for 5 days at the onset of each URTI during 12 months while 21 children not receiving alternate day PDN were randomised to receive placebo for 5 days at the onset of each URTI for 12 months. At the end of 12 months, children continuing in the study crossed over to alternate treatment regimen and were followed for a further 12 months. The data could not be included in a

meta-analysis as the data were not provided separately for participants in the first and second parts of the cross over study. Thirty three children completed the two year study. In the PDN arm, 115 episodes of URTI were associated with 11 relapses (9.5%). In the placebo arm, 101 episodes of URTI were associated with 25 relapses (24.7%). This study was at high risk of attrition and reporting bias.

Conclusions

One study [14] concluded that there was no evidence that a short course of low dose daily PDN in children with URTI compared with placebo prevents relapse of SSNS. This study was adequately powered and at low risk of bias for all risk of bias attributes. However in the subgroup analyses, confidence intervals were wide reflecting the small numbers of participants in each subgroup.

Four studies [11-13, 17] concluded that a short course of low dose daily PDN compared with alternate day PDN or no PDN in children with URTI reduces the risk of relapse in SSNS. These studies were all at a high risk of bias for two or more study attributes as well as being at risk of imprecision because of small numbers of participants in each group.

Studies at increased risk of bias are more likely to show a benefit of treatment [18, 19]. However small numbers of participants in subgroups in the PREDNOS 2 and small numbers in the other studies result in imprecision. Therefore there is insufficient evidence to recommend routine use of low dose daily PDN for URTI-associated relapse prevention. However, such an approach may be considered in children already taking low dose alternate day PDN where there is a history of repeated infection-associated relapses.

Table S6: GRADE-based evidence for the different steroid-sparing agents used in children with FRNS/SDNS
Data from [20].

Rituximab compared with prednisone or placebo with/without calcineurin inhibitors in relapsing SSNS

Outcome	Study design: N (Total participants)	Findings and direction of effect	Strength of evidence (GRADE)	Conclusion
**Major outcomes**				
N with relapse at 3 months	RCT: 3 (132)<br>SDNS: 3 RCTs	Three RCTs with 132 participants with consistent and precise information on outcome.<br>RR for relapse at three months is 0.32 [95% CI 0.14, 0.70]<br>Absolute number with relapse: placebo 580 per 1000; RTX 170 per 1000.	Moderate (evidence downgraded for imprecision)	Risk of relapse is probably reduced by RTX compared with prednisone/placebo with/without CNIs.
N with relapse at 6 months	RCT: 6 (302)<br>SDNS: 5 RCTs<br>FRNS/SDNS: 1 RCT	Six RCTs with 302 participants with consistent and precise information on outcome.<br>RR for relapse at six months is 0.19 [95% CI 0.09, 0.39].<br>Absolute numbers with relapse: placebo 548 per 1000; rituximab 126 per 1000.	Moderate (evidence downgraded for imprecision)	Risk of relapse is probably reduced by RTX compared with PDN/placebo with/without CNIs.

N with relapse at 12 months	RCT: 4 (228)<br>SDNS: 3 RCTs<br>FRNS/SDNS: 1 RCT	Four RCTs with 228 participants with consistent but imprecise information on outcome, with heterogeneity between studies.<br>RR for relapse at 12 months is 0.50 [95% CI 0.28, 0.89].<br>Absolute numbers with relapse: placebo 606 per 1000; RTX 382 per 1000.	Moderate (evidence downgraded for imprecision)	Risk of relapse is probably reduced by RTX compared with PDN/placebo with/without CNIs.
**Adverse outcome**				
Infusion reactions	RCT: 4 (252)	Four RCTs with 252 participants with consistent but imprecise information on outcome.<br>RR for infusion reactions during studies<br>was 5.83 [95% CI 1.34, 25.29].<br>Absolute number with reactions: placebo 8 per 1000; RTX 46 per 1000.	Low (evidence downgraded for imprecision)	Risk of infusion reactions may be increased with RTX compared with PDN/placebo with/without CNIs.

Severe infections	RCT: 3 (222)	Three RCTs with 222 participants with consistent but imprecise information on outcome.<br>RR for severe infection is 0.90 [95% CI 0.26, 3.15]<br>Absolute number with infections: Placebo 180 per 1000; RTX 162 per 1000.	Low (evidence downgraded for imprecision)	Risk of severe infections may be increased with RTX compared with PDN/placebo with/without CNIs.
Arthropathy	RCT: 2 (84)	Two RCTs with 84 participants with consistent but imprecise information on outcome.<br>RR for arthropathy is 3.92 [95% CI 0.45, 33.98].<br>Absolute numbers with arthropathy: placebo 12 per 1000; RTX 47 per 1000.	Low (evidence downgraded for imprecision)	Risk of arthropathy may be increased with RTX compared with PDN/placebo with/without CNIs.

GRADE: Grading of Recommendations Assessment, Development and Evaluation used to assess certainty (or quality) of the body of evidence

RR = relative risk; 95% CI = 95% confidence intervals

MMF compared with calcineurin inhibitors (cyclosporin, tacrolimus) in relapsing SSNS

**Outcome**	**Study design: N (Total participants)**	**Findings & direction of effect**	**Strength of evidence (GRADE)**	**Conclusion**
**Major outcomes**				

Relapse by 12 months	RCT: 2 (82) FRNS/SDNS: 2 RCTs	Two RCTs with 82 participants with consistent but imprecise information on outcome.<br>RR for relapse is 1.90 [95% CI 0.66, 5.46] when comparing MMF with CsA.<br>Absolute numbers with relapse: CsA 238 per 1,000; MMF 452 per 1,000.	Low (evidence downgraded for risk of bias issues and imprecision)	Risk of relapse may not differ between MMF and CsA.
Relapse rate at 12 months	RCT: 3 (142) FRNS/SDNS: 3 RCTs	Three RCTs with 142 participants with consistent but imprecise information on outcome.<br>Mean relapse rate/year 0.83 higher with MMF when compared to CsA (can be 0.33 higher to 1.33 higher).	Low (evidence downgraded for risk of bias issues and imprecision)	Relapse rate/year may be higher with MMF compared with CsA.
**Other outcomes**				
GFR at 12 months	RCT: 1 (14)	One RCT with 24 participants with imprecise effects on eGFR.<br>RR for reduced GFR is 0.33 [95% CI 0.01, 7.45] when comparing MMF with CsA.<br>Absolute number with reduced eGFR: cyclosporin 83 per 1000; MMF 28 per 1000.	Very low (evidence downgraded for risk of bias issues and imprecision)	It is uncertain whether eGFR differs between treatments with MMF and CsA.
**Adverse effects**				

Hypertension	RCT: 3 (144)	Three RCTs with 144 participants with consistent but imprecise information on outcome.<br><br>RR for hypertension at 12 months is 0.30 [95% CI 0.09, 1.07] when comparing MMF with cyclosporin.<br><br>Absolute number with hypertension: cyclosporin 292 per 1000; MMF 88 per 1000.	Low (evidence downgraded for risk of bias issues and imprecision)	Risk of hypertension may not differ between MMF and CsA.
Hypertrichosis	RCT: 3 (140)	Three RCTs with 140 participants with consistent information on outcome showed increased risk of hypertrichosis.<br><br>RR for hypertrichosis at 12 months is 0.23 [95% CI 0.10, 0.50] when comparing MMF with CsA.<br><br>Absolute number with hypertrichosis: cyclosporin 426 per 1000; MMF 98 per 1000.	Low (evidence downgraded for risk of bias issues and small patient numbers)	Number with hypertrichosis may be higher with CsA.

Gum hypertrophy	RCT: 3 (144)	Three RCTs with 140 participants with consistent information on outcome showed increased risk of gum hypertrophy.<br>RR for gum hypertrophy at 12 months is 0.09 [95% CI 0.02, 0.47] when comparing MMF with CyA.<br>Absolute number with gum hypertrophy: CyA 208 per 1000; MMF 19 per 1000.	Low (evidence downgraded for risk of bias issues and small patient numbers)	Number with gum hypertrophy may be higher with CsA.

GRADE: Grading of Recommendations Assessment, Development and Evaluation used to assess certainty (or quality) of the body of evidence

RR = relative risk; 95% CI = 95% confidence intervals

MMF compared with levamisole in relapsing SSNS

Outcome	Study design: N (Total Participants)	Findings & direction of effect	Strength of evidence (GRADE)	Conclusion
**Major outcomes at 12 months**				
N with relapse	RCT: 1 (149) FRNS/SDNS	One RCT with 149 participants with imprecise information on outcome showing no difference in outcome between treatment groups.<br>RR for relapse at 12 months is 0.90 [95% CI 0.70, 1.16].<br>Absolute number with relapse: levamisole 658 per 1000; MMF 592 per 1000.	Low (evidence downgraded for single study and imprecision)	Risk of relapse during treatment may not differ between MMF and levamisole.

N with FRNS/SDNS	RCT: 1 (149) FRNS/SDNS	One RCT with 149 participants with imprecise information on outcome showed no difference in outcome between treatment groups.<br>RR for FRNS/SDNS at 12 months is 0.88 (95% CI 0.41, 1.87).<br>Absolute number with FRNS/SDNS: levamisole 164 per 1000; MMF 145 per 1000.	Low (evidence downgraded for single study and imprecision)	Risk of FRNS/SDNS may not differ between MMF and levamisole.
**Adverse effects**				
Abdominal pain	RCT: 1 (149)	One RCT with 149 participants with imprecise information on outcome showed no difference in outcome between treatment groups.<br>RR for abdominal pain is 1.26 (0.66 to 2.40)<br>Absolute number with abdominal pain: levamisole 178 per 1000; MMF 224 per 1000.	Low (evidence downgraded for single study and imprecision)	Risk of abdominal pain may not differ between MMF and levamisole.
Anaemia	RCT: 1 (149)	One RCT with 149 participants with very imprecise information on outcome showed no difference in outcome between treatment groups.<br>RR for anaemia RR 0.48 (95% CI 0.04, 5.18)<br>Absolute number with anaemia: levamisole 27 per 1000; MMF 13 per 1000.	Very low (evidence downgraded for single study and imprecision)	It is uncertain whether the risk for anaemia differs between MMF and levamisole.

GRADE: Grading of Recommendations Assessment, Development and Evaluation used to assess certainty (or quality) of the body of evidence

RR = relative risk; 95% CI = 95% confidence intervals

Levamisole compared with prednisone or placebo in relapsing SSNS

Outcome	Study design: N. (Total/type participants)*	Findings & direction of effect	Strength of evidence (GRADE)	Conclusions
**Major outcomes by 12 months**				
Relapse on treatment	RCTs: 7 (426)<br>FRNS: 2 RCTs<br>SDNS: 2 RCTs<br>FRNS/SDNS: 3 RCTs	Seven RCTs with 426 participants with imprecise information on outcome.<br>RR for relapse 0.52 [95% CI 0.33, 0.82]<br>Absolute number with relapse: PDN/placebo 764 per 1000; levamisole 398 per 1000.	Low (evidence downgraded for risk of bias issues and heterogeneity between study results)	Risk of relapse may be reduced with levamisole compared with PDN/ placebo.
**Adverse effects**				
Leucopenia	RCTs: 3 (214)	Three RCTs with 214 participants with imprecise information about uncommon outcome<br>RR for leukopenia during treatment is 4.18 [95% CI 0.72 to 24.21]<br>Absolute number with leukopenia: PDN/placebo 10 per 1000; levamisole 41 per 1000	Low (evidence downgraded for risk of bias issues and imprecision)	Risk of leucopenia may not differ between levamisole and PDN/placebo.
Positive ANA	RCT: 1 (100)	One RCT with 100 participants with imprecise information about uncommon outcome<br>RR for positive ANA during treatment RR 3.00 [0.13, 71.92]<br>Absolute number with positive ANA: PDN/placebo 10 per 1000; levamisole 30 per 1000	Low (evidence downgraded for risk of bias issues and imprecision)	Risk of positive ANA during treatment may be higher with levamisole but small numbers result in considerable uncertainty about the true estimate.

GRADE: Grading of Recommendations Assessment, Development and Evaluation used to assess certainty (or quality) of the body of evidence

RR = relative risk; 95% CI = 95% confidence intervals; ANA = antinuclear antibody;

*One study (Weiss 1993) excluded as participants received 2.5 mg/kg for two doses on consecutive days rather than 2.5 mg/kg on alternate days

Alkylating agents (cyclophosphamide and chlorambucil) compared with cyclosporin in relapsing SSNS

Outcome	Study design: N (Total participants)	Findings & direction of effect	Strength of evidence (GRADE)	Conclusions
**Major outcomes by 12 months**				
Relapse at end of therapy (6-9 months)	RCTs: 2 (95)<br>FRNS: 1 RCT<br>FRNS/SDNS: 1 RCT	Two RCTs with consistent but imprecise information about the outcome.<br>RR for relapse at end of therapy is 0.91 [95% CI 0.55, 1.48]<br>Absolute number with relapse: CsA 400 per 1000; alkylating agents 364 per 1000.	Low certainty evidence (downgraded for risk of bias issues and imprecision)	Number with relapse may not differ between CsA and alkylating agents at end of therapy (6-9 months).
Relapse at 12-24 mths after end of therapy	RCTs: 2 (95)<br>FRNS: 1 RCT<br>FRNS/SDNS: 1 RCT	Two RCTs with consistent but imprecise information about the outcome.<br>RR for relapse at 12-24 months after the end of therapy is 0.51 [95% CI 0.35, 0.74]<br>Absolute number with relapse: CsA 860 per 1000; alkylating agents 439 per 1000.	Low certainty evidence (downgraded for risk of bias issues and small numbers of participants).	Number with relapse at 12-24 months after the end of therapy may be higher with CsA.
**Adverse effects**				

Hypertrichosis	RCTs: 2 (106)	Two RCTs with consistent but imprecise information about the outcome.<br>RR for hypertrichosis is 0.06 [95% CI 0.01, 0.40]<br>Absolute number with hypertrichosis: CsA 339 per 1000; alkylating agents 20 per 1000	Low certainty evidence (downgraded for risk of bias issues and small number of participants)	Number with hypertrichosis may be higher with CsA.
Gum hypertrophy	RCTs: 2 (106)	Two RCTs with consistent but imprecise information about outcome<br>RR for gum hypertrophy is 0.08 [95% CI 0.01, 0.59].<br>Absolute number with gum hypertrophy: CsA 232 per 1000; alkylating agents 19 per 1000.	Low certainty evidence (downgraded for risk of bias issues and small number of participants)	Number with gum hypertrophy may be higher with CsA.
Number of participants with elevated creatinine levels	RCTs: 2 (106)	Two RCTs with consistent but imprecise information about the outcome.<br>RR for elevated creatinine levels is 0.20 [95% CI 0.02, 1.69]).<br>Absolute number with elevated creatinine: CsA 89 per 1000; alkylating agents 18 per 1000.	Low certainty evidence (downgraded for risk of bias issues and imprecision)	Numbers with elevated creatinine levels may not differ between alkylating agents and CsA.

Hypertension	RCTs: 1 (40)	One RCT with very imprecise information about the outcome.<br>RR for hypertension is 0.33 [95% CI 0.01, 7.72].<br>Absolute number with hypertension: CsA 50 per 1000; alkylating agents 17 per 1000.	Very low certainty evidence (downgraded for risk of bias issues and imprecision)	It is uncertain whether the numbers with hypertension differ between alkylating agents and CsA.
Leucopenia	RCT: 1 (66)	One RCT with very imprecise information about the outcome.<br>RR for leucopenia is 29.84 [95% CI 1.84, 483.93].<br>Absolute number with leucopenia: CsA no events; alkylating agents not calculated.	Very low certainty evidence (downgraded for risk of bias issues and imprecision)	It is uncertain whether the numbers with leucopenia differ between alkylating agents and CsA .

GRADE: Grading of Recommendations Assessment, Development and Evaluation used to assess certainty (or quality) of the body of evidence

RR = relative risk; 95% CI = 95% confidence intervals

Alkylating agents (cyclophosphamide and chlorambucil) compared with PDN in relapsing SSNS

Outcome	Study design<br>N studies (N)	Findings & direction of effect	Strength of evidence (GRADE)	Conclusion
**Major outcomes by 6-12 months**				
Cyclophosphamid; relapse at 6-12 mths	RCTs: 4 (141)<br>FRNS: 4 RCTs	Four RCTs with consistent and precise information about the outcome.<br>RR for relapse at 6-12 months is 0.47 [95% CI 0.34, 0.66]	Moderate certainty evidence (downgraded for risk of bias issues)	Numbers with relapse are probably fewer with CYC compared with PDN.

Chlorambucil; relapse at 6 mths	RCTs: 2 (41)<br>FRNS: 1 RCT<br>SDNS: 1 RCT	Two RCTs with consistent but imprecise information about the outcome.<br>RR for relapse at 6 months is 0.19 [95% CI 0.03, 1.09]	Low certainty evidence (downgraded for risk of bias issues and small numbers of participants)	Numbers with relapse may be fewer with chlorambucil compared with PDN.
Alkylating agents; relapse at 6-12 mths	RCTs: 6 (182)<br>FRNS: 5 RCT<br>SDNS: 1 RCT	Six RCTs with consistent and precise information about the outcome.<br>RR for relapse at 6-12 months is 0.44 (95% CI 0.32 to 0.60)<br>Absolute number with relapse: PDN/placebo 740 per 1000; alkylating agents 326 per 1000	Moderate certainty evidence (downgraded for risk of bias issues)	Numbers with relapse are probably reduced by CYC compared with PDN.
**Adverse effects**				
Leucopenia (CPA)	RCTs: 2 (78)	Two RCTs with with consistent but imprecise information about the outcome.<br>RR for leukopenia at 6-12 months is 10.63 [95% CI 1.45, 78.05] (risk lower with PDN)<br>Absolute number with leucopenia: No events with PDN; number with CYC cannot be calculated.	Very low certainty evidence (downgraded for risk of bias issues and imprecision)	It is uncertain whether the number with leucopenia are increased with CYC compared with PDN.

Leucopenia (CHL)	RCT: 1 (20)	One RCT with uncertain information on outcome because of small patient numbers.<br>RR of leukopenia at 6 months is 2.50 [95% CI 0.11, 54.87]<br>Absolute number with leucopenia: No events withPDN; number with CHL cannot be calculated.	Very low certainty evidence (downgraded for risk of bias issues and imprecision)	It is uncertain whether the number with leucopenia are increased with CHL compared with PDN.

GRADE: Grading of Recommendations Assessment, Development and Evaluation used to assess certainty (or quality) of the body of evidence

RR = relative risk; 95% CI = 95% confidence intervals; CPA = cyclophosphamide; CHL = chlorambucil

Intravenous cyclophosphamide compared with oral cyclophosphamide in relapsing SSNS

Outcome	Study design. N studies (N)	Findings & direction of effect	Strength of evidence (GRADE)	Conclusions
**Major outcomes**				
Relapse at 6 months	RCT: 2 (83)<br>SDNS: 2 RCTs	Two RCTs with consistent information for the outcome.<br>RR for relapse is 0.54 [95% CI 0.34, 0.88].<br>Absolute numbers with relapse: oral CPA 524 per 1,000; IV CPA 283 per 1,000.	Low certainty evidence (downgraded for risk of bias issues and imprecision due to small patient numbers)	Numbers with relapse at 6 months may not differ between IV and oral CYC.

Relapse at end of study (24 months)	RCT: 2 (83)<br>SDNS: 2 RCTs	Two RCTS with consistent information for the outcome.<br>RR for relapse at the end of study is 0.99 [95% CI 0.76, 1.29].<br>Absolute numbers with relapse: oral CPA 619 per 1,000; IV CPA 613 per 1,000.	Low certainty evidence (downgraded for risk of bias issues and imprecision due to small patient numbers)	Number with relapse at the end of study may not differ between IV and oral CYC.
**Adverse effects**				
Leucopenia	RCT: 2 (83)	Two RCTs with consistent but imprecise information for the outcome.<br>RR for leukopenia is 0.37 [95% CI 0.09, 1.51]<br>Absolute numbers with leucopenia: oral CYC 143 per 1000; IV CYC 53 per 1000.	Low certainty evidence (downgraded for risk of bias issues and imprecision due to small patient numbers)	Number with leucopenia may not differ between IV and oral CYC.
Hair loss	RCT: 2 (83)	Two RCTs with consistent but imprecise information for the outcome.<br>RR for hair loss is 0.19 [95% CI 0.04, 1.03]<br>Absolute numbers with hair loss: oral CPA 381 per 1000; IV CYC 72 per 1,000.	Low certainty evidence (downgraded for risk of bias issues and imprecision due to small patient numbers)	Number with hair loss may not differ between IV and oral CYC.

All infections	RCT: 2 (83)	Two RCTs with consistent but imprecise information for the outcome.<br>RR for infections is 0.14 [0.03, 0.72].<br>Absolute numbers with infections: oral CYC 238 per 1000; IV CYC 33 per 1000.	Low certainty evidence (downgraded for risk of bias issues and imprecision due to small patient numbers)	Number of infections may be lower with IV compared with oral CYC.

GRADE: Grading of Recommendations Assessment, Development and Evaluation used to assess certainty (or quality) of the body of evidence;

RR = relative risk; 95% CI = 95% confidence intervals; CYC = cyclophosphamide

Table S7: SSNS Randomised controlled trials – Steroid-sparing and immunomodulatory agents in FRNS, SDNS

Based on:

Larkins et al. [20]

Table S7.1 Outcome/ Relapses

Study	Population	Intervention	Comparator	Outcome	No. studies	Total Participants	RR*	95% CI*	Conclusions
**RITUXIMAB**									
Four studies 2011 to 2018<br><br>Ahn 2018 [21]<br>Iijma 2014a [22]<br>NEPHRUTIX 2018 [23]<br>Ravani 2011 [24]	SDNS with CNI dependence	Rituximab 1-4 doses	Placebo (2)<br>CNI (3)<br>Prednisone (4)	Relapse by 3 months<br><br>Relapse by 6 months<br><br>Relapse by 12 months	3<br><br>3<br><br>2	132<br><br>122<br><br>168	0.32<br><br>0.30<br><br>0.74	0.14 – 0.70<br><br>0.19 – 0.47<br><br>0.58 – 0.94	Reduced risk of relapse with rituximab compared with CNI/prednisone
Three studies 2015 to 2020<br><br>RITURNS 2018 [25]<br>Ravani 2015 [26]<br>Ravani 2020 [27]	SDNS/FRNS on prednisone	Rituximab 1 - 2 doses	Low dose prednisone (3)<br>CNI (1)	Relapse by 6 months<br><br>Relapse by 12 months	3<br><br>3	180<br><br>180	0.06<br><br>0.39	0.01 – 0.22<br><br>0.17 – 0.88	Reduced risk of relapse with RTX compared with CNI/prednisone
**CYCLOSPORIN**									
APN 2006 [28]	SSNS 1st episode	Cyclosporin 8 wks<br>Prednisone 12 wks	Prednisone 12 wks	Relapse by 6 months<br><br>Relapse by 12 months	1	104	0.33<br><br>0.72	0.13 – 0.83<br><br>0.46 – 1.13	Reduced risk of relapse with cyclosporin by 6 months<br>No difference in relapse by 12 months
Two studies 1992, 1993<br><br>Niaudet 1992 [29]<br>Ponticelli 1993 [30]	FRNS/SDNS SDNS	Cyclosporin 6-12 mths	Cyclophosphamide 8 wks or Chlorambucil 6 wks	Relapse by 6-9 months	2	95	0.91	0.55 – 1.48	No difference in risk of relapse
**CYCLOSPORIN DOSING**									
Ishikura 2008 [31]	FRNS	Cyclosporin	Cyclosporin	Relapse at 6	1	44	0.31	0.1 – 1.02	No difference in

Study	Population	Intervention	Comparator	Outcome	No. studies	Total Participants	RR*	95% CI*	Conclusions
		dosing after 6 months: aiming for trough levels at 60-80 ng/ml (mean dose 5.4 mg/kg/d to maintain remission)	dosing after 6 months: fixed dose 2.5 mg/kg/d	months<br><br>Relapse at 12 months<br><br>Relapse at 24 months	 <br>1<br><br><br>1	 <br>44<br><br><br>44	 <br>0.33<br><br><br>0.65	 <br>0.16 – 0.70<br><br><br>0.45 – 0.94	relapse at 6 months<br><br>Reduced risk of relapse with cyclosporine aiming for trough levels at 60-80 ng/ml
Iijima 2014 [32]	SDNS/FRNS	High-dose cyclosporine 6 months (target C2 levels 600-700 ng/ml), followed by 450-550 ng/ml for 18 months	Low-dose cyclosporine 6 months (target C2 level 450-550 ng/ml), followed by 300-400 ng/ml for 18 months	Relapse at 24 months	1	85	0.74	0.45 – 1.22	No difference in relapse at 24 months
**MYCOPHENOLATE MOFETIL**									
Two studies 2008, 2013<br><br>Dorresteijn 2008 [33]<br>Gellermann 2013 [34]	FRNS/SDNS	MMF 1 year	Cyclosporin 1 yr	Relapse by 12 months	2	82	1.90	0.66 – 5.46	No difference in risk of relapse (Gellermann - first part of cross-over study included)
One study 2019<br>Sinha 2019 [35]	FRNS/SDNS	MMF 1 year	Levamisole 1 yr	Relapse by 12 months	1	149	0.90	0.70 – 1.16	No difference in risk of relapse
**LEVAMISOLE**									
Eight studies 1991 to 2015<br><br>Abeyagunawardena 2006 [36]<br>Al-Saran 2006 [37]	FRNS/SDNS	Levamisole 4 to 12 months	Placebo, prednisone or no treatment	Relapse by 4 – 12 months	8	474	0.52	0.33 – 0.82	Reduced risk for relapse with levamisole

Study	Population	Intervention	Comparator	Outcome	No. studies	Total Participants	RR*	95% CI*	Conclusions
BAPN 1991<br>Dayal 1994<br>Gruppen 2015 [38]<br>Rashid 1996 [39]<br>Sural 2001 (group 1 and 3) [40]<br>Weiss 1993 [41]									
Two studies 2001, 2005<br><br>Donia 2005 [42]<br>Sural 2001 [40]	FRNS/SDNS	Levamisole 6 months	Cyclophosphamide<br>Prednisone	Relapse at 6 – 9 months after treatment	2	97	1.17	0.76 – 1.81	No difference in risk of relapse
**ALKYLATING AGENTS**									
Six studies<br>1970 to 2001<br><br>Alatas 1978 [43]<br>Barratt 1970 [44]<br>Chiu 1973 [45]<br>Grupe 1976 [46]<br>ISKDC 1974 [47]<br>Sural 2001 [40]	FRNS/SDNS	Cyclophosphamide or chlorambucil	Prednisone	Relapse at 6 months	6	202	0.44	0.32 – 0.60	Reduced risk of relapse with alkylating agents
Two studies 2004, 2006<br><br>Prasad 2004 [48]<br>Abeyagunawardena 2006 [49]	SDNS	IV Cyclophosphamide monthly for 6 months	Oral cyclophosphamide 12 weeks	Relapse 6 months after treatment<br><br>Relapse after 12 to 24 months	2	83	0.54<br><br>0.99	0.34 - 0.88<br><br>0.76 – 1.29	Reduced risk of relapse after 6 months with IV CPH<br><br>No difference in risk of relapse after 12-24 months
APN 1982 [50]	SDNS/FRNS	Cyclophosphamide	Chlorambucil	Relapse at 12 months<br><br>Adverse	1	50	1.15<br><br>0.65	0.69 – 1.94<br><br>0.42 – 1.01	No difference in risk of relapse<br><br>No difference of

Study	Population	Intervention	Comparator	Outcome	No. studies	Total Participants	RR*	95% CI*	Conclusions
				effects					frequency of adverse effects
Abeyagunawardena 2007 [51]	SDNS	Cyclophosph amide	Vincristine	Relapse at 12 months	1	39	0.54	0.54 – 1.12	No difference in risk of relapse
**CYCLOPHOSPHAMIDE DURATION/ DOSING**									
Barratt 1973 [52]	FRNS	Cyclophosph amide 8 weeks	Cyclophosphami de 2 weeks	Relapse at 12 months	1	32	0.15	0.04 – 0.57	Uncertain, very low certainty of evidence
Ueda 1990 [53]	SDNS	Cyclophosph amide 12 weeks	Cyclophosphami de 8 weeks	Relapse at 12 months<br><br>Relapse at 24 months	1	73	1.04<br><br>0.98	0.75 – 1.44<br><br>0.74 – 1.28	No difference in risk of relapse at 12 and 24 months
McCrory 1973 [54]	FRNS	Cyclophosph amide 5 mg/kg/day, 6 weeks	Cyclophosphami de 2.5 mg/kg/d, 12 weeks	Relapse at 12 months<br><br>Adverse effects	1	14	2.33	0.11 – 48.99	Uncertain, very low certainty of evidence
**CHLORAMBUCIL DOSING REGIMEN**									
Baluarte 1978 [55]	FRNS	Chlorambucil – increasing dosing regimen	Chlorambucil stable dosing regimen	Relapse at 12 months	1	21	0.18	0.01 – 3.41	Uncertain, very low certainty of evidence
**OTHER AGENTS**									
**MIZORBINE**									
Yoshioka 2000 [56]	FRNS	Mizoribine	Placebo	Relapse at 6 and 12 months<br><br>Adverse effects during treatment<br><br>Cumulative remission rate	1<br><br>1<br><br>1	197<br><br>197<br><br>197	 <br>1.56	 <br>0.97 – 2.49	No data<br><br>More adverse effects during treatment<br><br>Little/ no difference (HR 0.79, 95% CI

Study	Population	Intervention	Comparator	Outcome	No. studies	Total Participants	RR*	95% CI*	Conclusions
									0.57 – 1.22)
**AZITHROMYCIN**									
Zhang 2014 [57]	SSNS	Azithromycin	Prednisone	Relapse at 6 months	1	190	0.55	0.3 – 1.02	No difference in risk of relapse
**AZATHIOPRINE**									
2 studies 1970, 1977<br><br>ISKDC 1970 [58]<br>Barratt 1977	SDNS/FRNS	Azathioprine	Prednisone	Relapse at 6 months	2	60	0.9	0.59 – 1.38	No difference in risk of relapse
**ACTH gel**									
Wang C (ATLANTIS) 2018 [59]	FRNS/SDNS	ACTH gel	Prednisone for relapse only	Relapse at 6 months	1	31	1.00	0.83 – 1.20	No difference in risk of relapse. Frequent adverse effects
**FUSIDIC ACID**									
Cerkauskiene 2005 [60]	FRNS	Oral fusidic acid	Prednisone	Time to remission/ relapse<br>Adverse affects	1	18	Not perf.	Not performed	No meta-analyses performed<br><br>No differences in mean time to remission or time to relapse

*RR, Relative risk, 95% CI, 95% confidence intervals

Table S7.2 Adverse events

Intervention vs. Comparator	Adverse Event	N studies	Total Participants	Intervention N events/ N participants	Comparator N events/ N participants	RR*	95% CI*	I²
**Rituximab vs. Placebo or control**	Moderate to severe infusion reactions	4<br>Ijima 2011 [22]<br>Ravani 2011 [24]<br>Ravani 2015 [26]<br>RITURNS 2018 [25]	252	11/126	1/126	5.83	1.34 – 25.29	0%
	Severe Infection	3<br>Ijima 2011 [22]<br>Ravani 2011 [24]	222	13/111	20/111	0.9	0.26 – 3.15	46.21%
	Arthropathy	2<br>Ravani 2011 [24]<br>Ravani 2015 [26]	84	3/42	0/42	3.92	0.45 – 33.98	0%
**MMF vs. Levamisole**	Peritonitis	1<br>Sinha 2019 [35]	149	1/76	3/73	0.32	0.03 – 3.01	n.a.
	Abdominal pain	1<br>Sinha 2019 [35]	149	17/76	13/73	1.26	0.66 – 2.4	n.a.
	Anaemia	1<br>Sinha 2019 [35]	149	1/76	2/73	0.48	0.04 -5.18	n.a.
	Leucopenia	1<br>Sinha 2019 [35]	149	1/76	0/73	2.88	0.12 – 69.65	n.a.
**MMF vs. Cyclosporin**	Hypertension	3<br>Dorresteijn 2008 [33]<br>Uddin 2016 [61]<br>Gellermann 2013	144	6/72	21/72	0.3	0.09 – 1.07	40.26%
	Hypertrichosis	3<br>Dorresteijn 2008 [33]<br>Uddin 2016 [61]<br>Gellermann 2013 [34]	140	5/72	29/68	0.23	0.1 – 0.5	0%
	Lymphopenia	2<br>Dorresteijn 2008 [33]<br>Gellermann 2013 [34]	84	1/42	2/42	0.64	0.08 – 4.85	0%

Intervention vs. Comparator	Adverse Event	N studies	Total Participants	Intervention N events/ N participants	Comparator N events/ N participants	RR*	95% CI*	I^2
	Gum hypertrophy	3 Dorresteijn 2008 [33] Uddin 2016 [61] Gellermann 2013 [34]	144	0/72	15/72	0.09	0.02 – 0.47	0%
	Reduced GFR	1 Dorresteijn 2008 [33]	24	0/12	1/12	0.33	0.01 – 7.45	n.a.
	Pneumonia	1 Dorresteijn 2008 [33]	24	1/12	0/12	3.0	0.13 – 67.06	n.a.
	Diarrhea	1 Uddin 2016 [61]	60	4/60	0/60	9.0	0.51 – 160.17	n.a.
**Levamisole vs. steroids or placebo or both or no treatment**	Leucopenia	3 Al-Saran 2006 [37] Sural 2001 [40] Gruppen 2015 [38]	214	6/112	1/102	4.18	0.72 – 24.21	0%
	ANCA positive/ arthritis	1 Gruppen 2015 [38]	100	1/50	0/50	3.0	0.13 – 71.92	0%
**Levamisole vs. Cyclophosphamide**	Infection	1 Donia 2005 [42]	40	13/20	12/20	1.08	0.67 -1.75	n.a.
	Leucopenia	2 Donia 2005 [42] Sural 2001 [40]	97	1/50	5/47	0.25	0,04 – 1.48	0%
	Abnormal liver function tests	1 Donia 2005 [42]	40	0/20	1/20	0.33	0.01 – 7.72	n.a.
**CsA + Predn. vs. prednisolone alone**	Number needing cytotoxic agents	1 APN 2006 [28]	104	5/49	12/55	0.47	0.18 – 1.23	n.a.
	Creatinine at the end of study	1 APN 2006 [28]	87	Mean creatinine 48.2 ± 11.1 µmol/l	Mean creatinine 46.2 ± 10 µmol/l	Mean difference 2	-2.44, 6.44	n.a.

Intervention vs. Comparator	Adverse Event	N studies	Total Participants	Intervention N events/ N participants	Comparator N events/ N participants	RR*	95% CI*	I^2
**CsA dose:** changing vs. fixed dose	Hypertension	1<br>Ishikura 2008 [31]	44	6/24	2/20	2.5	0.57 – 11.05	n.a.
	Psychological disorder	1<br>Ishikura 2008 [31]	44	0/24	1/20	0.28	0.01 – 6.52	n.a.
	Obesity	1<br>Ishikura 2008 [31]	44	1/24	0/20	2.52	0.11 – 58.67	n.a.
	Hirsutism	1<br>Ishikura 2008 [31]	44	4/24	2/20	1.67	0.34 – 8.18	n.a.
	Transient elevated creatinine	1<br>Ishikura 2008 [31]	44	2/24	1/20	1.67	0.16 – 17.06	n.a.
	Gym hypertrophy	1<br>Ishikura 2008 [31]	44	2/24	4/20	0.42	0.08 – 2.04	n.a.
	GIT effects	1<br>Ishikura 2008 [31]	44	2/24	2/20	0.83	0.13 – 5.4	n.a.
	Convulsions	1<br>Ishikura 2008 [31]	44	1/24	0/20	2.52	0.11 – 58.67	n.a.
	Fatigue	1<br>Dorresteijn 2008 [33]	24	1/12	0/12	3	0.13 – 67.06	n.a.
**CsA dose:** High vs. lower C2 target level	Encephalopath y	1<br>Iijima 2014 [32]	85	2/43	1/42	1.95	0.18 – 20.74	n.a.
	Infection	1<br>Iijima 2014 [32]	85	15/43	13/42	1.13	0.61- 2.07	n.a.
	Pneumonia	1<br>Iijima 2014 [32]	85	3/43	1/42	2.93	0.32 – 27.06	n.a.
	Renal toxicity	1<br>Iijima 2014 [32]	85	2/43	0/42	4.89	0.24 – 98.85	n.a.
	Hirsutism	1<br>Iijima 2014 [32]	85	23/43	20/42	1.12	0.74 – 1.71	n.a.

Intervention vs. Comparator	Adverse Event	N studies	Total Participants	Intervention N events/ N participants	Comparator N events/ N participants	RR*	95% CI*	I^2
	Gum hypertrophy	1<br>Iijima 2014 [32]	85	4/43	7/42	0.56	0.18 – 1.77	n.a.
	Hypertension	1<br>Iijima 2014 [32]	85	7/43	5/42	1.37	0.47 – 3.97	n.a.
**Alkylating agents vs. CsA**	Serume creatinine	2<br>Niaudet 1992 [29]<br>Edefonti 1998	106	0/50	5/56	0.2	0.02 – 1.69	0%
	Hypertrichosis	2<br>Niaudet 1992 [29]<br>Edefonti 1998	106	0/50	19/56	0.06	0.01 – 0.40	0%
	Gum hypertrophy	2<br>Niaudet 1992 [29]<br>Edefonti 1998	106	0/50	13/50	0.08	0.01 – 0.59	0%
	Hypertension	1<br>Niaudet 1992 [29]	40	0/20	1/20	0.33	0.01 – 7.72	n.a.
	Leucopenia	1<br>Edefonti 1998	66	12/30	0/30	29.84	1.84 – 483.93	n.a.
**Alkylating agents vs. steroids or placebo or boths**	Leucopenia<br>**CPA:**<br><br>**CHL:**	3<br>2 CPA (Sural 2001 [40], Chiu 1973 [45])<br>1 CHL (Alatas 1978 [43])	78<br><br>20	10/39<br><br>1/11	0/39<br><br>0/9	10.63<br><br>2.5	1.45 – 78.05<br><br>0.11 – 54.87	0%<br><br>n.a.
**CPA duration long** (12 wks) **vs. short** (8 wks)	Leucopenia	1<br>Ueda 1990 [53]	73	14/41	9/32	1.21	0.6 – 2.44	n.a.
**CPA low dose** (2.5 mg/kg/d) **vs. high dose** (5 mg/kg/d)	Leucopenia	1<br>McCrory 1973 [54]	14	2/8	5/6	0.3	0.09 – 1.05	n.a.
	Lymphopenia	1<br>McCrory 1973 [54]	14	5/8	6/6	0.66	0.38 – 1.15	n.a.

Intervention vs. Comparator	Adverse Event	N studies	Total Participants	Intervention N events/ N participants	Comparator N events/ N participants	RR*	95% CI*	I^2
	Alopecia	1<br>McCrory 1973 [54]	14	0/8	4/6	0.09	0.01 – 1.35	n.a.
	Gastrointestinal	1<br>McCrory 1973 [54]	14	1/8	3/6	0.25	0.03 - 1.85	n.a.
	Genitourinary	1<br>McCrory 1973 [54]	14	1/8	3/6	0.25	0.03 – 1.85	n.a.
**IV CPA vs. oral CPA**	Leucopenia	2<br>Abeyagunawardena 2006 [49]<br>Prasad 2004 [48]	83	2/41	6/42	0.37	0.09 – 1.51	0%
	Hair loss	2<br>Abeyagunawardena 2006 [49]<br>Prasad 2004 [48]	83	2/41	16/42	0.19	0.04 – 1.03	0%
	All infections	2<br>Abeyagunawardena 2006 [49]<br>Prasad 2004 [48]	83	1/41	10/42	0.14	0.03 – 0.72	0%
	Nausea and vomiting	1<br>Prasad 2004 [48]	47	2/26	0/21	4.07	0.21 – 80.51	n.a.
**CHL dose:** increasing vs. stable	Leucopenia	1<br>Baluarte 1978 [55]	21	7/11	3/11	2.12	0.74 – 6.04	n.a.
	Thrombocytopenia	1<br>Baluarte 1978 [55]	21	2/11	0/11	4.58	0.25 – 85.33	n.a.
**CPA vs. CHL**	Leucopenia	1<br>APN 1982 [50]	50	3/26	3/24	0.92	0.21 – 4.14	n.a.
	Lymphopenia	1<br>APN 1982 [50]	50	7/26	15/24	0.43	0.21 – 0.87	n.a.
	Thrombocytopenia	1<br>APN 1982 [50]	50	7/26	15/24	0.43	0.21 – 0.87	n.a.

Intervention vs. Comparator	Adverse Event	N studies	Total Participants	Intervention N events/ N participants	Comparator N events/ N participants	RR*	95% CI*	I^2
	Severe infection	1<br>APN 1982 [50]	50	2/26	0/24	4.63	0.23 – 91.81	n.a.
	Hair loss	1<br>APN 1982 [50]	50	4/26	0/24	8.33	0.47 – 147-07	n.a.
	Hematuria	1<br>APN 1982 [50]	50	0/26	0/24	Not estimable	Not estimable	n.a.
**MZR vs. placebo**	Hyperuricaemia	1<br>Yoshioka 2000 [56]	197	16/99	4/98	3.96	1.37 – 11.42	n.a.
	Hepatic dysfunction	1<br>Yoshioka 2000 [56]	197	9/99	9/98	0.99	0.41 – 2.39	n.a.
	Leucopenia	1<br>Yoshioka 2000 [56]	197	2/99	1/98	1.98	0.18 – 21.48	n.a.

RR, Relative risk, 95% CI, 95% confidence intervals, I^2, measure of heterogeneity between studies (< 40%, not important, 30-60% moderate; >60% substantial). N.a. = not applicable

Table S8: SSNS observational studies – Steroid-sparing and immunomodulatory agents in FRNS, SDNS

CONTENT:

Table S8.1 Calcineurin-Inhibitors (CNI): Cyclosporin A (CsA) and Tacrolimus (TAC)
Table S8.2 Alkylating Agents: Cyclophosphamide (CPA) and Chlorambucil (CHL)
Table S8.3 Mycophenolate mofetil (MMF)/ Mycophenolate Sodium (MPS)
Table S8.4 Levamisol (LEV)
Table S8.5 Rituximab (RTX)
Table S8.6 Mizoribine (MZR)
Table S8.7 Other Agents (Vincristine, Saquinavir, ACTH, Azathioprine)

COMMENTS:
- Only studies within the past 20 years were included (since 2000)
- Only studies that evaluating 20 or more children with SSNS were included for CNI, CPA, MMF

ABBREVIATIONS:
NS = Nephrotic syndrome; FRNS = Frequently relapsing nephrotic syndrome; SDNS = Steroid dependent nephrotic syndrome; RR = relapse rate; MMF = Mycophenolate mofetil; EC-MPS = Enteric coated mycophenolate sodium; MPA = Mycophenolic acid; pred = prednisolone or prednisone; LEV = levamisole; CPA = cyclophosphamide; CHL = chlorambucil; CSA = cyclosporine A; TAC = Tacrolimus; MZR = mizoribine; VCR = Vincristine; AZA = Azathioprine; MCD = Minimal change disease; FSGS = Focal and segmental glomerulosclerosis; AE = adverse effects; GIT = gastrointestinal

Table S8.1: Calcineurin-Inhibitors (CNI): Cyclosporin A (CsA) and Tacrolimus (TAC)

1st author, year, country of origin	Title of Publication	Study design	Keywords	N	Population characteristics	Treatment	Outcomes
**CALCINEURIN-INHIBITOR**							
Kemper 2004 [62] Germany	Recurrence of severe steroid dependency in cyclosporine A-treated childhood nephrotic syndrome	Retrospective chart analysis 3 centers	Childhood cyclosporin A Long-term remission Maintenance therapy Steroid-dependent nephrotic syndrome	46	**Age at NS onset:** 3.0 (IQR 0.8-6.9) yrs<br>**Age at CsA:** not stated<br>**Duration of NS at CsA start:** 4.3 (0.9-12.9) yrs<br>**Duration of CsA at recurrence of SDNS**: 5.1 (1.2-11.5)<br>**Age at data analysis:** 20.4 (8.6-29.1) yrs<br>**FU:**<br>**Indications:** SDNS after	**CsA:** 5 mg/kg/d aiming for trough levels 80-120 µg/l, in case of frequent relapses: 150-250 µg/l<br>**Duration:**<br>**Other:** oral prednisone in tapering dose<br>**After CsA:**<br>**CHL:** 0.15 mg/kg for 12 wks (n=6)<br>**CPA**: 2mg/kg for 12 weeks (n=3)	**Recurrence of SDNS in CsA-treated patients:**14/46 (30%) after duration of CsA treatment of 5.1 (1.2-11.5) yrs<br><br>**6/14 with CHL:** 4 long-term remission, 1 SDNS then LEV, 1 SDNS then CsA again<br><br>**3/14 with CPA**: 0 long-term remission (1 relapse after 15 mths; 1 SDNS with maintenance

1st author, year, country of origin	Title of Publication	Study design	Keywords	N	Population characteristics	Treatment	Outcomes
					failure of CPA; SDNS recurrence despite CsA maintenance treatment	**LEV:** 2.5 mg/kg/48h (n=5)	steroids; 1 SDNS with CsA, LEV, MMF)<br><br>**5/14 with LEV:** 1 long-term remission, 1 SDNS with remission after CHL, 1 with5 relapses o n 2 yrs, 1 SDNS with CPA, 1 SDNS with Tac, CHL, CsA)
Iyengar A, 2004 [63] India	Cyclosporine in Steroid dependent and resistant childhood nephrotic syndrome	Retrospectiv e single center study	Chronic renal failure FSGS Minimal change nephrotic syndrome	41, o those 30 SDN S	**Age at NS onset:** 22 (11-148) mths<br>**Age at CsA:** 93 (48-936) mths<br>**Duration of NS before CsA**: 24 (6-72) mths<br>**FU:** 71 (20-205) mths<br>30 M, 11 F<br>**Indications:** SDNS (n=30), SRNS (n=11) despite prior treatment with cytotoxic treatments	**CsA:** 6-7 mg/kg/d in 2 divided doses, target levels 100-200 ng/ml, maintenance 3-4 mg/kg/day<br>**Duration:** 24 (6-72) mths<br>**Other:** oral steroids in tapering dose	**SDNS:**<br>**CsA responder:** 86.2%<br>**CsA-non-responder:** 13.8%<br>**RR:** not stated<br>**Time to relapse:** not stated<br>**AE:** infections 8/41, Chronic renal failure 7/41
El-Husseini 2005 [64] Egypt	Long-term effects of cyclosporine in children with idiopathic nephrotic syndrome: a single-centre experience	Retrospectiv e chart review Single center	Cyclosporine Long-term Nephrotic syndrome Pathology treatment	74	**Age at NS onset:** not stated<br>**Age at CsA:** 11 ± 3.6 yrs<br>**FU:** 5.8 ± 3 yrs before CsA, 6.1 ± 1.9 yrs after CsA<br>M 54, F 20<br>**Indications:** SDNS (N=74) with CPA previously (n=32, CPA-responsive 9/32) and/or steroid toxicity (n=32), SRNS (N=43)	**CsA:** 5 mg/kg/d, adjusted to maintain trough levels 100-150 ng/ml for 2 mths, thereafter 50-100 ng/ml<br>**Duration:** 34 ± 12 mths, maintenance dose 1.3 ± 0.8 mg/kg/d<br>**Other:** prednisone on tapering dose	**Remission:** 66 (82%)<br>**Time to remission**: 4.4 ± 1.4 wks<br>**Relapses:** 19/66 during pred. tapering or within 1 mth after stop of predn.<br>**Time to relapse:** not stated<br>**AE:** gym hyperplasia 25 (33.8%), hypertrichosis 51 (68.9%), hypertension 4 (5.4%), renal dysfunction 2 (2.7%)
Rinaldi 2005 [65] Italy	Cyclosporine therapy monitored with abbreviated area under curve in nephrotic syndrome	Retrospectiv e analysis	Cyclosporine monitoring abbreviated area under the curve Nephrotic syndrome Cyclosporine phropathy	18	**Age at NS onset:** 4.7 (1.6-13.5) yrs<br>**Age at CsA:** 7.8 (2.5-14.4) yrs<br>**Duration of NS at start of CsA**: 3.3 (0.2-12.6) yrs<br>**FU (after CsA discontinuation):** 2.3 (0.6-3.3 yrs)<br>12 M, 6 F<br>**Indications:** SDNS (n=15)	**CsA:** 5 mg/kg/d divided into 2 doses, adjusting to maintain mean CsA blood concentration 250-350 ng/ml (obtained from abbreviated AUC: 2 and 6 hrs post-CsA); mean dose 4.4 (3.6-5.8) mg/kg/day<br>**Duration:** 4.9 (2.2-6.9)	**RR:** decreased from 4/year to 0.8/yr (p<0.0001)<br>**Time to relapse:**<br>**Predn.dose:** decreased from 0.9 mg/lg/day to 0.2 mg/kg/d (p<0.0001).<br>**CsA Nephropathy (CsAN) in renal biopsy**: clear-lesions: none; Lesions suggestive of CsAN 5/15 -> CsA treatment was

1st author, year, country of origin	Title of Publication	Study design	Keywords	N	Population characteristics	Treatment	Outcomes
					despote LEV and/or cytotoxic agents, partially SSNS (n=3)	yrs, at least 2 yrs<br>**Other:** oral steroid in tapering dose	discontinued<br>**AE: CsAN:** see above; reversible gum hypertrophy (53%), reversible hypertrichosis (31%), reversible hypertension (16%).
Mahmoud I 2005 [66] Egypt	Single-centre experience with cyclosporine in 106 children with idiopathic focal segmental glomerulosclerosis	Retrospective chart review<br>Single center	Cyclosporine<br>Focal segmental glomerulosclerosis	106, of those 61 SDNS	**Age at NS onset:** 5.8 ± 4.3 yrs<br>**Age at CsA:** 10.5 ± 3 yrs<br>**Duration of NS before CsA:** 5.8 ± 2.8 yrs<br>**FU:** 6.1 ± 1.9 yrs after CsA start<br>44 M, 17 F<br>**Indications:** SDNS (n=61) with prior CPA (29/61), SRNS (n=45)	**CsA:** 5 mg/kg/day in 3 divided doses, adjusted to trough levels 100-150 ng/ml for 2 mths; thereafter 50-100 ng/ml; maintenance dose 1.9 ± 0.9 mg/kg/d<br>**Duration:** 22.6 ± 10 mths<br>**Other:** oral steroids in tapering dose; 81/106 received ketoconazol	**Remission:** 56/61 (91.8%)<br>**Steroid withdrawal:** all 56 in remission, 13/56 with relapses<br>**CsA discontinued:** 12/56, relapse in 11/12<br>**RR:** not stated<br>**Time to relapse:** not stated<br>**AE:** hypertrichosis 33/61 (54%), gum hyperplasia 15/61 (24%), hypertension 4/61 (6.6%), 2/61 (3.3%)
Wasilewska 2005 [67] Poland<br><br>Only abstract available (Article in Polish)	The effect of cyclosporine A in steroid-dependent nephrotic syndrome in children	Retrospective analysis		21	**Age at NS onset:**<br>**Age at CsA:** 12.1 ± 4.6 yrs<br>**FU:** 12 months of CsA<br>16 M, 5 F<br>**Indications:** SDNS	**CsA:**<br>**Duration:** 12 mths<br>**Other:** oral steroids, ACEi	Decrease of protein excretion. Increase of serum creatinine. Disturbs 24 hr rhythm of arterial blood pressure (nocturnal fall of systolic and diastolic BP dcreased to < 10% from 14-15%)<br>**Remission:**<br>**RR:**<br>**Time to relapse:**<br>**AE:**
El-Husseini 2006 [68] Egypt	Impact of the cyclosporine-ketoconazole interaction in children with steroid-dependent idiopathic nephrotic syndrome	Retrospective chart review<br>2 centers	Ketoconazole<br>Cyclosporine<br>Children<br>Steroid-dependent<br>Nephrotic syndrome<br>Treatment	102	**Ketoconazole group:** n=78<br>**Non-ketoconazole group:** n=24<br><br>**Age at NS onset:** not stated<br>**Age at CsA:** 5.4 ± 3.6 yrs<br>**FU:** not stated<br>77 M, 25 F<br>**Indications:** SDNS	**CsA:** 4-5 mg/kg/d in 2 divided doses (> 6 yrs); 5-6 mg/kg/d in 3 divided doses (<6 yrs), trough level target 100-150 ng/ml for 2 mths, thereafter 50-100 ng/ml<br>**Ketoconazol:** 50 mg/day, accompanied with initial 1/3 decrease in CsA dose<br>**Duration:** 23.72 ± 12.22 mths (Keto); 19.31±11.78	**Remission:**<br>Keto: 72/78 (92.3%)<br>Non-keto: 17/24 (70.8%)<br>**Steroid withdrawal:**<br>Keto: 58/72 (74.4%), all maintained remission<br>Non-keto: 11/24 (45.8%), 10 maintained remission<br>**CsA discontinuation:**<br>Keto: 19/72 while in remission, 18 with relapses<br>Non-Keto: 6/17 while in remissin, 6 with relapses

1st author, year, country of origin	Title of Publication	Study design	Keywords	N	Population characteristics	Treatment	Outcomes
						mths (non-keto)<br>**Other:** oral steroids in tapering dose, ACEi (captopril) (n=32)	Reduction of costs of CsA by using ketoconazole: 40% after 1mth, 48% at 1 yr<br>**RR:** not stated<br>**Time to relapse:** not stated<br>**AE** (Keto/non-Keto): hirsutism 35 (44.9%)/ 10 (41.6%); gingival hyperplasia 18 (23.1%)/ 5 (20.8%), hypertension 6 (7.7%)/ 3 (12.5%), renal dysfunction 0/2 (12.5%)
Sinha 2006 [69]<br>UK	Treatment of severe steroid-dependent nephrotic syndrome (SDNS) in children with tacrolimus	Retrospective case series	Calcineurin inhibitors<br>Children<br>Cyclosporine A, nephrotic syndrome<br>Steroid dependency<br>tacrolimus	10	**Age at NS onset:** 2.9 (1.6-12.9) yrs<br>**Age at TAC:** 10.9 (3.6-21.4) yrs<br>**Age at CsA:** 4.9 (2.6-13.4) yrs<br>**FU:**<br>8M, 2 F<br>**Indications:** severe SDNS despite sequential treatment with CPA (10), CsA (10), 2nd CPA (7)	**TAC (after failure of CsA or adverse effects on CsA):** 0.1 mg/kg/d in 2 divided doses, target trough level 5-10 µg/l.<br>**CsA:** 5 mg/kg/d in 2 divided doses, target trough level 50-100 µg/l.<br>**Duration:**<br>TAC: 5 (1-7) yrs<br>CsA: 2 (1-7) yrs<br>**Other:**	**Remission:** 6/10 on TAC<br>**RR:** TAC: 1 (0-5)/yr; CsA 2 (0-6)/yr (p=0.79)<br>**Time to relapse:** not stated<br>**Cumulative steroid dosage:** TAC: 73.9 (2.1-468.9) vs. CsA: 105 (17.3-602.7) mg/kg/d (p=0.54)<br>**% change in eGFR**: Tac: -11.7 (-34.3, +3.5), CsA -5.8 (-37.7, +38.3)<br>**CNI toxicity (histology):** Tac: 1/13 (1pt.), CsA: 3/16 (2 pts).<br>**AE:** hypertension 7 (CsA, Tac), insulin-dependent diabetes mellitus 1 (Tac)
Sheashaa H 2007 [70]<br>Egypt	Does cyclosporine achieve a real advantage for treatment of idiopathic nephrotic syndrome in children? A long-term efficacy and safety study	Retrospective analysis	Nephrotic syndrome<br>Cyclosporine<br>Pediatric	197, of those 103 SDNS	**Age at NS onset:** 4 (1-13) yrs<br>**Age at CsA:** 5 (1-12) yrs<br>**FU:**<br>**Indications:** SDNS (n=103), SRNS (n=94), prior treatment with CPA n= 104 (53%)	**CsA:** 4-5 mg/kg/d in 2 divided doses (> 6 yrs); 5-6 mg/kg/d in 3 divided doses (<6 yrs), trough level target 100-150 ng/ml for 2 mths, thereafter 50-100 ng/ml<br>**Duration:** 22.2 ± 12.3 mths<br>**Other:** oral steroids in tapering dose, ketoconazole 50-100 mg/d to achieve target	**Remission:** 90/103 (87.4%)<br>**RR:** not stated<br>**Time to relapse:** not stated<br>**AE** (all 197 pts): renal impairment 18 (9.1%), hypertension 37 (18.8%), gym hyperplasia 50 (25.4%), hypertrichosis 103 (52.3%), hyperkalemia 2 (1%), cholestasis 1 (0.5%), seizure 1 (0.5%)

1st author, year, country of origin	Title of Publication	Study design	Keywords	N	Population characteristics	Treatment	Outcomes
						CsA level with the least possible CsA dose	
Kranz 2008 [71]<br>Germany	Cyclosporine-A-induced nephrotoxicity in children with minimal-change nephrotic syndrome: long-term treatment up to 10 yrs	Retrospective chart review	Minimal-change nephrotic syndrome<br>Cyclosporine A	20	**CsA (after CPA):** n=20<br>**CPA-Controls:** n=15<br><br>**Age at NS onset:** 4.4 ± 2.2 yrs (CsA), 4.0 ± 2.9 yrs (CPA)<br>**Age at start of initial CPA:** 5.7 ± 2.2 yrs (CsA), 6.0 ± 3.2 yrs (CPA-Controls)<br>**Age at start of CsA**: 8.4 ± 3.0 yrs<br>**FU:** 5.4 ± 2.2 yrs (CsA), 4.9 ± 3.4 yrs (CPA-controls)<br>12M, 8 F<br>**Indications:** SDNS, all treated with CPA prior CsA	**CsA:** 100-150 mg/m^2 BSA in 2 divided doses; target trough level 80-120 ng/ml<br>**Duration:** 5.4 ± 2.2 yrs, 10 pts: 5-11 yrs<br>**Other:** oral prednisone in tapering dose	**Sustained Remission or reduction of relapses to an infrequent relapsing NS:** 19/20<br>**RR:** not stated<br>**CsA toxicity:** 5 renal biospies performed without CsA toxicity<br>**eGFR:** fell from 136.3 ± 10.0 at CsA start to 114.5 ± 14.5 ml/min*1.73m^2 at latest follow-up eGFR at lates FU in CPA-controls 126.4 ± 19.8<br>**AE:** hypertension 3/20 (CsA), otherwise not stated
Kengne-Wafo 2009 [72]<br>Italy	Risk Factors for Cyclosporin A Nephrotoxicity in Children with Steroid-Dependant nephrotic syndrome	Retrospective chart review	SDNS<br>Cyclosporin<br>Nephrotoxicity<br>Children	53	Evaluation of CsAN in 71 renal biopsies of 53 pts.<br><br>**Age at NS onset:** 3.5 (0.7 to 13.2) yrs<br>**Age at CsA:** 6.5 (2.2-14.2) yrs<br>**Duration of NS before CsA**: 1.1 (0.4-11.2) yrs<br>**Age at biopsy:** 11.5 (5.6-20.3) yrs<br>**FU:**<br>36 M, 17 F<br>**Indications:** SDNS on CsA	**CsA:** 4.2 ± 1.2 mg/kg/d or 125 ± 28 mg/m^2/d; C2 levels 454 ± 122 ng/ml<br>**Duration:** 4.7 ± 2.0 yrs before renal biopsy; total CsA 5.9 (2.9-12.5 yrs)<br>**Other:** prednisone in tapering dose, amlodipine (15), ramipril or losartan (11), aldactone (3), labetolol (1)	**Relapse rate:** decreased from 2.0 ± 1.1 (range 1-6) to 0.5 ± 0.5 (range 0.0-3.0) (p<0.0001)<br>**Steroid dose:** decreased from 11.6 (range 6.5-22.5) to 5.0 (0-15.5) mg/m^2/d (p<0.0001).<br>**CsAN:** 22/71 (31%)<br>Mild lesions: 17/22, 5/22 moderate, no severe lesions<br>Tubular/vascular lesions:<br>Isolated: 11<br>Combined: 11<br>**eGFR (Schwartz) change:** reduced by 10.2 ± 15.5 % from baseline, remained stable at biopsy: -7.3 ± 22.4% from baseline<br>**Risk factors for CsAN:** CsA-C2-Levels > 600 ng/ml
Leroy V 2009 [73]<br>France	Growth in boys with idiopathic nephrotic	Retrospective analysis	Growth retardation<br>Steroid	64	**Age at NS onset:** 2.7 (0.8-10.9) yrs<br>**Age at CsA:** not stated	**CsA:** 150 mg/m^2/d in 2 divided doses, target trough level 100-150	**Remission:** not stated<br>**RR:** not stated<br>**Time to relapse:** not stated

1st author, year, country of origin	Title of Publication	Study design	Keywords	N	Population characteristics	Treatment	Outcomes
	syndrome on long-term cyclosporine and steroid treatment		therapy<br>Nephrotic syndrome<br>Minimal change disease<br>Cyclosporin A<br>Adult height		**Duration of NS before CsA:** 15 mths (1 mth to 14.8 yrs)<br>**FU:** 10 (3-17) yrs, at least 3 yrs, all male<br>**Indications:** SDNS with long-term combined CsA and steroid treatment; 46 with prior treatment with alkylating agents	ng/ml<br>**Duration:** not stated<br>**Other:** oral steroids in tapering dose/ low-dose Steroid exposure: 9.9 (2.6-16.4) yrs	**AE:** growth retardation 17, otherwise not stated<br><br>**Normal growth:** 47 (73.4%)<br>Height-SDS at diagnosis: 0.4 (-1.7 to 1.8)<br>H-SDS at last FU: -0.5 (-1.8 to 1.8)<br>**Growth retardation:** 17 (26.6%)<br>H-SDS at diagnosis: -0.4 (-3.0 to -0.9) ($p<0.01$ compared to normal growth)<br>H-SDS at last FU: -2.4 (-3.9 to 0.1) ($p<0.001$ compared to normal growth)<br><br>**Steroid exposure:** 9.2 (2.6-15.8) vs. 10.8 (4.0-16.4) yrs<br>**Cum. Steroid dose over FU period**: 37.3 (14.2-95.4) vs. 55.3 (30.1-1000.0) mg/m^2
Suzuki K 2010 [74] Japan	Benefits of Once-Daily Administration of Cyclosporine A for Children with steroid-dependent, relapsing syndrome	Retrospective chart review	Children<br>Cyclosporine A<br>Frequently relapsing nephrotic syndrome<br>Single-daily dose administration<br>Steroid-sparing effect	19	**Single daily dose group (SDD):** n=10<br>**Twice-daily dose (TDD):** n=9<br><br>**Age at NS onset:**<br>SDD: 8.9 ± 5.6 yrs<br>TDD: 7.8 ± 3.8 yrs<br>**Age at CsA:**<br>SDD: 10.7 ± 5.0 yrs<br>TDD: 9.6 ± 3.8 yrs<br>**FU since CsA start:**<br>SDD: 34.9±17.2 mths<br>TDD: 67.0±30.5 mths<br>**Indications:** SDNS, FRNS despite prior CPA (n=12)	**CsA:**<br>**SDD:** 1.5 ± 0.4 mg/kg/d<br>**Duration:** 26.7 ± 9.9 mths<br>**TDD:** 3.7 ± 0.7 mg/kg/d ($p<0.001$)<br>**Duration:** 34.9 ± 17.2 yrs<br>**Other:** oral steroids in tapering dose	**Sustained remission without medication:**<br>SDD 3/10, TDD 3/9<br>**Relapses:**<br>SDD: 7/10. TDD 6/9<br>**RR:**<br>SDD: decreased from 4.7±2.0/yr to 0.5±0.2/yr<br>TDD: decreased from 5.1±2.3/yr to 0.2±0.2/yr<br>**AE:** mild biopsy-proven CsAN 1/9 (TDD), 0/10 (SDD)
Ishikura 2010 [75] Japan	Treatment with microemulsified cyclosporine in	Prospective multicenter trial	Clinical trial microemulsified	62	**Age at NS onset:** 3.0 (1.3-14.5) yrs<br>**Age at CsA:** 5.4 (1.7-15.3) yrs	**CsA:** maintaining trough levels 80-100 ng/ml for 6 mths (mean dose 5.1	**FRNS-free survival at 24 mths:** 58.1% (95% CI, 45.8-70.3%)<br>**RR:** decreased from 4.6 ± 1.4 to

1st author, year, country of origin	Title of Publication	Study design	Keywords	N	Population characteristics	Treatment	Outcomes
	children with frequently relapsing nephrotic syndrome		cyclosporine<br>Nephrotic syndrome<br>Pediatric nephrology		**FU:** 24 mths<br>48 M, 14 F<br>**Indications:** FRNS	mg/kg/d) , 60-80 ng/ml for 18 mths (4.5 mg/kg/d).<br>**Duration:** 24 mths<br>**Other:** low-dose oral steroids, ACEi (4), HMG-CoA reductase inhibitors (1), antihypertensive drugs (4)	0.7 ± 1.5/ year (p<0.0001)<br>**CsA toxicity** (58/62 biopsies at 24 mths): mild 5/58 (8.6%)<br>**AE:** hypertrichosis 20 (32.2%), hypertension 8 (12.9%), gingival hypertrophy 7 (11.3%), Elevation of AP 5 (8.1%), herpes zoster 2 (3.2%), transient elevated serum creatinine 1 (1.6%), general fatigue 1 (1.6%)
Ishikura 2012 [76]<br>Japan	Two-year follow-up of a prospective clinical trial of cyclosporine for frequently relapsing nephrotic syndrome in children	Prospective follow-up analysis of Ishikura 2010		46	FU study 24 mths after CsA discontinuation<br><br>**Group A (n=32):** no relapses during initial CsA treatment<br>**Group B (n=12):** with relapses during initial CsA<br><br>**Age at NS onset:**<br>**Age at CsA discontinuation:** 6.5 (5.5-9.6) yrs<br>**FU:** 48 mths<br>39M, 7F<br>**Indications:** FRNS with 2yrs CsA treatment	**CsA:** maintaining trough levels 80-100 ng/ml for 6 mths (mean dose 5.1 mg/kg/d) , 60-80 ng/ml for 18 mths (4.5 mg/kg/d).<br>**Duration:** 24 mths, tapered and stopped after 24 mths<br>**Other:**	**Relapses:** 37/46<br>Group A: No relapse 6/32 (19%), infrequent relapses 9/32 (28%), regressed again to FRNS 17 (53%)<br>Group B: no relapse 1/12 (8.3%), infrequent relapses 2/12 (16.7%), regressed again to FRNS 9 (75%).<br>**Time to relapse after CsA disc.:** Group A: 4.3 (1.5-15.6) mths; group B: 0.5 (0.0-1.1) mths<br>Time to regression of FRNS: Group A: 16.6 mths, group B: 3.8 mths<br>**Relapse-free survival** at 24 mths after CsA discontinuation: 15.3%(all), 17.9% (group A), 8.3% (group B) (p<0.0001).<br>**FRNS-free survival** 40.8%<br>**AE:** 6/46 (GIT discomfort 3, hypertension 3)
Wang 2012 [77]<br>China	Treatment of Tacrolimus or cyclosporine A in children with idiopathic nephrotic syndrome	Prospective single center study	Idiopathic nephrotic syndrome<br>Therapy<br>Cyclosporine A<br>Tacrolimus	74 of those 40 SDNS/FRNS	**CsA:** n=24, of those FRNS/SDNS 16<br>TAC: 50, of those FRNS/SDNS 24<br><br>**Age at NS onset:**<br>CsA: 7.6 ± 4.5 yrs,	**CsA:** 3-4 mg/kg/day, divided into 2 doses, dose adjustment to trough level, target 100-150 ng/ml; overall final dose: 2.72 ± 0.59 mg/kg/day	**Remission at 6 mths:**<br>CsA: 14/16, TAC 22/24<br>**Relapses within 1st year:**<br>CsA: 4/14, 2/4 with >3/yr<br>TAC: 10/22, 5/10 with >3/yr<br>**Relapses within 2nd year:**<br>CsA: 6/14, 2/6 with > 3/yr

1st author, year, country of origin	Title of Publication	Study design	Keywords	N	Population characteristics	Treatment	Outcomes
					TAC: 8.3 ± 4.8 yrs<br>**Age at CsA/TAC start:**<br>CsA: 7.7 ± 5.0 yrs,<br>TAC: 8.6 ± 5.8 yrs<br>**FU:** at least 24 mths,<br>51 M, 23 F<br>**Indications:** SRNS, SDNS, FRNS, previous agents MMF (n=17), CPA (n=8)	**TAC:** 0.05-0.15 mhg/kg/day, divided into 2 doses, target trough level 5-12 ng/ml; overall final dose 0.087±0.027 mg/kg/day<br>**Duration:** at least 24 mths<br>**Other:**	TAC: 12/22, 5/12 wth >3/yr<br>**AE:**<br>Nephrotoxicity: 4/24 (CsA) vs. 0/50 (Tac) (p=0.002); hirsutism: 8/24 (CsA) vs. 0/50 Tac (p<0.001).<br>ALT/AST elevation: 5/24 (CsA), 8/50 (TAC); GIT symptoms 5/24 (CsA), 11/50 (TAC), diabetes 0/24 (CsA), 1/50 (TAC); psychiatric symptoms 0/24 (CsA), 2/50 (TAC); severe infections 9/24 (CsA), 15/50 (TAC); nutritional anemia 0/24 (CsA); 2/50 (TAC)
Supavekin 2013 [78] Thailand<br><br>Only abstract available	Tacrolimus in steroid resistant and steroid dependent childhood nephrotic syndrome	Retrospective chart analysis<br>Single center		18, of those 9 SDNS	**Age at NS onset:** 6.0 (1-14.4) yrs<br>**Age at Tac:**<br>**Duration of NS before Tac:** 3.5 (0.2-14) yrs<br>**FU:** 3.1 (0.2-6.4) yrs<br>12 M, 6 F<br>**Indications:** SDNS (9), SRNS (9)	**Tac:** 0.09 (0.03-0.2) mg/kg/day with trough level 4.1 (1.3-9.9 mcg/l)<br>**Duration:** 1.3 (0.3-6.2) yrs<br>**Other:**	**Remission:** 9/9, 4/9 (44.4%) with relapses<br>**RR:**<br>**Time to relapse:**<br>**AE:**
Bock ME 2013 [79]<br><br>Only abstract available	Treatment of childhood nephrotic syndrome with long-term, low-dose tacrolimus	Retrospective chart review<br>Single center		40	**Age at NS onset:**<br>**Age at Tac:**<br>**FU:**<br>**Indications:** SDNS, SRNS (not differentiated in abstract) despite prior second-line agents	**Tac:**<br>**Duration:** 25.2 (3-80) mths<br>**Other:** oral steroids	Not differentiated SDNS-SRNS:<br>**Remission:**<br>26% (at 1 yr), 48% (at 2yrs), 29% (3yrs)<br>**Time to remission:** 41 (10-270) days.<br>**RR:** not stated<br>**Time to relapse:**<br>**AE:**
Hamasaki 2017 [80] Japan	Nephrotoxicity in children with frequently relapsing nephrotic syndrome receiving long-	Retrospective chart review<br>Single center	Cyclosporine<br>Pediatrics<br>Nephrotic syndrome<br>Kidney biopsy<br>Nephrotoxicit	36	**Renal biopsies** 33/36<br><br>**Age at NS onset:** 3.6 (1.2-13.9) yrs<br>**Age at CsA:** 9.4 (2.9-18.5) yrs<br>**FU:**<br>28 M, 8 F	**CsA:**<br>Trough level 80-100 ng/ml for 6 mths, 60-80 ng/ml for 18 mths, 50-60 ng/ml thereafter<br>**Duration:** at least 3 yrs; 4.5 (3.0-11.9) yrs	**FRNS free survival rates:** 81% (at 2yrs), 27% (4 yrs)<br>**Mild to moderate CsAN:** 13/36 (36.1%)<br>**Risk factors of CsAN:**<br>Duration of CsA treatment (2-5 yrs: OR 3.84 (95% CI, 0.79-18.74)

1st author, year, country of origin	Title of Publication	Study design	Keywords	N	Population characteristics	Treatment	Outcomes
	term cyclosporine treatment		y		**Indications:** FRNS, prior treatment with CPA n=19	**Other:** low dose oral steroids: mizoribine (14), MMF (7), RTX (3)	vs. 0-2 yrs; > 5yrs 6.6 (1.18-36.94) vs. 0-2 yrs)<br>**AE:** apart from CsAN not stated
Kuroyanagi Y 2018 [81]<br>Japan	Effectiveness and nephrotoxicity of a 2-year medium dose of cyclosporine in pediatric patients with steroid-dependet nephrotic syndrome: determination of the need for follow-up kidney biopsy	Retrospective analysis	Childhood<br>Cyclosporine A<br>Steroid-dependent nephrotic syndrome<br>Kidney biopsy	38	**Age at NS onset:** 5.2 ± 2.9 yrs<br>**Age at CsA:** 7.1 ± 3.5 yrs<br>**Duration of NS before CsA**: 21.2 ± 23.7 mths<br>**FU:** 2 yrs after CsA start<br>17 M, 21 F<br>**Indications:** SDNS	**CsA:** 3-5 mg/kg/d (average: 3.6±0.9 mg/kg/d), adjusted to C2 target level of 450 ng/ml (average: 422 ± 133.5 ng/ml)<br>**Duration:** 25.9 ± 2.5 mths<br>**Other:** oral steroid in tapering dose, MMF (n=3), mizoribine (n=12)	**Remission:**<br>**RR:** decreased from 3.0/yr to 0.47/yr<br>**Time to relapse:** not stated<br>Steroid dose: decreased from 354.4 (204.6-438.9) mg/kg/yr to 48.9 (11.5-55.3) mg/kg/yr (p<0.01)<br>**AE:** mild CsAN at 2yrs 1/38
Yang 2019 [82]<br>Korea	Tacrolimus for children with refractory nephrotic syndrome: a one-year prospective, multicenter, and open-label study of Tacrobell®, a generic formula	1-yr prospective open-label, single-arm multicenter trial	Generic drugs<br>Nephrotic syndrome<br>tacrolimus	44	**Age at NS onset:** 5.2 ± 3.5 yrs<br>**Age at TAC:** 11.4 ± 4.2 yrs<br>**Duration of NS before TAC**: 6.2 ± 3.7 yrs<br>**FU:** 12 mths after TAC start<br>35 M, 9 F<br>**Indications:** SDNS, previous agents: CPA 26, CsA 40, LEV 9, Bredinin 12, Azathioprine 2, MMF 1, RTX 1	**TAC:** 0.1-0.2 mg/kg/d (Tacrobell®), trough level 5-10 ng/ml<br>**Duration:** 12 mths<br>**Other:** oral steroids in tapering dose	**Remission at 12 mths:** 34/44 (77.3%)<br>**Sustained remission at 12 mths:** 19 (43.2%)<br>**RR:** fell from 2.8± 1.3/yr to 0.9±1.0/yr<br>**Time to relapse:** 4.6±2.9 mths<br>**Cumulative steroid dose:** reduced from 139.7 ±151.9 mg/kg/yr to 102.2±120.8 mg/kg/yr<br>**AE:** GIT symptoms 1/44, headache 2, dizziness 1, hand tremor 2, transient hyperglycemia 1, transient glycosuria 3.
Delbet 2019 [83]<br>France	Infrequent Tacrolimus-induced nephrotoxicity in French patients with steroid-dependent nephrotic	Retrospective analysis<br>Single center	Idiopathic nephrotic syndrome<br>Tacrolimus<br>Children<br>Nephrotoxicity<br>Cyclosporine	21	TAC: n=15<br>CsA: n=6, of those 4 later TAC<br>**Age at NS onset:** 49 (29-66) mths<br>**Age at CNI:** 5.5 (3.6-10.8) yrs<br>**Age at kidney biopsy:** 108 (78-170) mths<br>**FU:**	**TAC:** 0.12 (0.10-0.19) mg/kg/day; trough level 5 (3.5-5.5) ng/ml<br>**CsA:** 4.5 (4.25-4.75) mg/kg/day; trough level 116.5 (96.5-123.5) ng/ml<br>**Duration:** >12 mths, median 30 (20-45) mths	**Remission:** not stated<br>**RR:** not stated<br>**AE:**<br>1/21 FSGS<br>21/22 MCGN<br>Evaluation of chronic CNI nephrotoxicity: 1/21 (required high CsA doses, initially up tp 10

1st author, year, country of origin	Title of Publication	Study design	Keywords	N	Population characteristics	Treatment	Outcomes
	syndrome				15 M, 6 F<br>**Indications:** SDN, prior agents MMF	**Other:** oral steroids in tapering dose, MMF (n=7)	mg/kg/day, trough levels 150-175 ng/ml);<br>Acute nephrotoxicity: 1/21
Fujinaga S, 2021 [84]<br>Japan	Efficacy of once-daily cyclosporine in Japanese children with steroid-dependent minimal change nephrotic syndrome	Retrospective analysis<br>Single center	SDNS<br>cylosporine	30	**Age at NS onset:** 6.6 (1.8-14.5) yrs<br>**Age at CsA:** 9.2 (3.1-15.8) yrs<br>**FU:**<br>26 M, 4 F<br>**Indications:** SDNS despite prior treatment MZR (10) and CPA (11)	**CsA:** single daily dose adjusted to C2 target level of 600 ng/ml; mean CsA dose: 2.6 ± 0.7 mg/kg/d to maintain C2 606±87 ng/ml<br>**Duration:**<br>**Other:** oral steroids in tapering dose	**Responder:** 20/30<br>**Relapses:** 11/20<br>**RR:** fell from 4.0/yr to 0.3/yr<br>**Non-responder:** 10/30, after switching to twice-daily CsA: 5/10 remained with treatment failure, requiring RTX<br>**RR:** not stated<br>**Time to relapse:**<br>**AE:** mild CsA-induced tubulointerstitial lesions 1/30
**Studies with CNI – comparing different steroid-sparing agents**							
**TAC vs. MMF**							
Wang 2016 [85]<br>China	Evaluation of mycophenolate mofetil or Tacrolimus in children with steroid sensitive but frequently relapsing or steroid-dependent nephrotic syndrome	Prospective single center study	Children<br>Mycophenolate mofetil<br>Primary nephrotic syndrome<br>tacrolimus	72	**MMF Group:** n=34, completed protocol n=30<br>**TAC Group:** n= 38, complete study protocol n=35<br><br>**Age at NS onset:** 43.1 ± 25.6 mths (MMF), 50.8 ± 31.1 mths (TAC)<br>**Age at initiation:** 64.2 ± 32.2 mths (MMF), 72.2 ± 32.3 mths (TAC)<br>**FU:**<br>51 M, 21 F<br>**Indications:** FRNS, SDNS	**MMF:** 20-30 mg/kg/d in 2 divided doses (max. 1g)<br>**TAC:** 0.05-0.15 mg/kg/d in 2 divided doses; trough levels 5-10 ng/ml<br>**Duration:** 12 mths<br>**Other:** low-dose oral steroids	**Remission at 12 mths:** MMF 24 (90%); TAC 31 (97%)<br>**RR at 6 and 12 mths:**<br>MMF: decreased from 2.56/6mths before MMF to 0.76/1st 6 mths and 0.67/2nd 6 mths (p<0.001)<br>TAC: decreased from 2.38/6mths to 0.41/ 1st 6 mths and 0.42/ 2nd 6 mths (p<0.001)<br>**Steroid dose: decreased from 0.61** ± 0.06 mg/kg/d (MMF)/ 0.66 ± 0.05 mg/kg/d (TAC) to 0.16 ± 0.02 mg/kg/d (MMF)/ 0.17 ± 0.03 mg/kg/d.<br>**AE:** MMF discontinued due to leucopenia/GIT symptoms and chickenpox 2/34; TAC discontinued due to severe infection/ refractory anemia 1 and neurologic symptoms 1/38; new onset of hypertension 2 (TAC).<br>Infections: 11.8% (MMF), 7.9%

1st author, year, country of origin	Title of Publication	Study design	Keywords	N	Population characteristics	Treatment	Outcomes
							(TAC).<br>Reversible leucopenia (1 MMF, 1 TAC), acute kidney injury (1 MMF, no Tac)
**TAC vs. MMF – after RTX**							
Fujinaga S 2013 [86] Japan	Cyclosporine versus Mycophenolate mofetil for maintenance of remission of steroid-dependent nephrotic syndrome after a single infusion of rituximab	Prospective study<br>Single center	Cyclosporine<br>Mycophenolate mofetil<br>Rituximab<br>Steroid-dependent nephrotic syndrome	29	**CsA after RTX:** n=13<br>**MMF after RTX:** n=16<br><br>**Age at onset of NS:** 6.4 ± 3.9 yrs<br>**Age at RTX:** 11.8 ± 4.3 yrs<br>**FU:**<br>19M, 10 F<br>**Indication:** severe SDNS despite CsA (for 49±35 mths) and/or MMF<br>Immunosuppr. agents at RTX: CsA 13, MMF 11, CsA and MMF 4, CsA and MZR 1	**RTX:** Single dose of 375 mg/m^2 (max. 500 mg)<br>**Duration of B-cell depletion**: 5 mths<br><br>**After RTX:**<br>**CsA group**: dose adjusted to C2 level of 400-500 ng/ml; mean dose 3.7 mg/kg/d. MMF and MZR discontinued.<br>**Duration** after RTX: 18 (5-29) mths<br>**MMF group:** adjusted to target MPA levels of 2-5 µg/ml. CsA discontiinued<br>**Duration** after RTX: 19 (7-44) mths<br>**Other:** Tapering dose of steroids	**Relapse after RTX:**<br>CsA: 3/13<br>MMF: 12/16<br>**Treatment failure:**<br>CsA: 2/13<br>MMF: 7/16<br><br>**RR:**<br>CsA: decrease from 4.4 ± 1.9 to 0.6±1.4/yr (86%, p<0.01)<br>MMF: decrease from 2.3±0.8 to 1.0/±0.9yr (58%; p<0.01)<br><br>**Steroid dose:**<br>CsA: decrease from 0.35±0.16 to 0.057±0.14 mg/kg/day (p<0.01)<br>MMF: decrease from 0.38±0.26 to 0.15±0.21 mg/kg/d (p<0.01)<br><br>**Dose of agent after RTX:**<br>CsA: decrease from 4.6 to 3.7 mg/kg/d (21%, p<0.01).<br>MMF:<br><br>**AE:**<br>RTX: transient infusion reaction: 13/29 (45%),<br>CsA. Hypertrichosis all. Mild CsAN 3<br>MMF: diarrhea 2, bacterial pneumonia 1
Fujinaga S 2015 [87] Japan	Positive role of rituximab in switching from	Prospective study<br>Single center	SDNS<br>Rituximab<br>Cyclosporine	26	**Age at NS onset:** 7.0 ± 4.0 yrs<br>**Age at CsA:** 8.3 ± 4.1 yrs	**MMF** started at initial dose 250 mg/12h, adkusted to MPA trough	**Remission** despite CsA withdrawal: 11/26 (42%)<br>**MMF Failure and RTX:** 11/26

1st author, year, country of origin	Title of Publication	Study design	Keywords	N	Population characteristics	Treatment	Outcomes
	cyclosporine to mycophenolate mofetil for children with high-dose steroid-dependent nephrotic syndrome		MMF		**Age at MMF:** 12.1 ± 4.0 yrs<br>**FU:** after MMF start: 28.8 ± 9.9 mths<br>16M, 10F<br>**Indication:** complicated SDNS despite CsA (for 46.5±27.2 mths), CsAN in 11 pts (42%) with CsA > 24 mths	level of 2-5 µg/ml (max. 1g bd).<br>After MMF start; CsA doseage gradually tapered<br>Duration:<br>Other: tapering dose of steroids, steroids fro relapses.<br>**In case of MMF treatment failure: RTX** (n=11)**:** single dose of 375 mg/m^2 (max. 500 mg)<br>**Duration**:<br>**Other:** tapering dose of steroids, steroids for relapse	(42%)<br>**Sustained remission > 1 yr** without steroids: 22/26 (85%)<br>**Discontinuation of MMF:** 15/26 (58%)<br>**RR:** with CsA: 1.0±0.9/yr; with MMF and RTX 0.7±0.5/yr (p=0.07)<br>AE: MMF: mild gastrointesinal symptoms 2, herpes simplex 2<br>RTX: mild infusion reactions 5/11, late-onset neutropenia requiring G-CSF 1/11
**CsA vs. CPA**							
Sümegi 2008 [88] Hungary	Long-term follow-up after cyclophosphamide and cyclosporine-A therapy in steroid-dependent and –resistant nephrotic syndrome	Retrospective study	Cyclosporine A, cyclophosphamide, immunosuppressive therapy, steroid reistant, steroid dependent	37, of those SDNS 23	**CPA:** n=22, of those SDNS n=15<br>**CsA:** n=15, of those SDNS n=8<br><br>**Age at NS onset:**<br>CPA: 7.7 ± 3.8 yrs<br>CsA: 9.5 ± 4.7 yrs<br>**FU:** 7.1 (5-13) yrs<br>25 M, 12 F<br>**Indications:** SDNS, SRNS	**CPA:** 2-2.5 mg/kg/d<br>Duration: 2.5 ± 0.5 mths<br>**CsA:** 3-5 mg/kg/day, trough levels 100-200 ng/ml<br>**Duration:** 28 ± 15 mths<br><br>**Other:** oral steroids in taopering dose	**Remission at 5 yrs:**<br>CPA: 20/22 (90.9%)<br>CsA: 10/15 (66.6%)<br>**RR:** decreased in both groups:<br>CPA: from 3.4±2.8/yr to 0.1±0.2/yr<br>CsA: from 3.7±3.1 to 0.6±0.8/yr<br>**Time to relapse:**<br>CPA: 29.9 ± 21.5 mths vs. CsA: 28.1 ± 22.4 mths<br>**AE:**<br>CPA: nausea 3/22, reversible hair loss 2/22, leucopenia 1/22, alopecia 1/22<br>CsA: hirsutism 3/15, tremor 2/15, gingival hyperplasia 2/15, nausea and appetite loss 1/15
**CsA – CPA - LEV**							
Abeyagunawardena 2003 [89]	The use of steroid-sparing agents in steroid-	Retrospective cohort study	Nephrotic syndrome Steroid		**CPA**: n=178 (1st agent)<br>**LEV**: n=113, of those 1st agent n=65	**CPA:** 3 mg/kg daily<br>**Duration:** 8 wks	**CPA:** sustained remission at 1 yr: 94/178 (54%), at 2 yrs: 44%, at 5 yrs: 32%, 2nd course of CPA:

1st author, year, country of origin	Title of Publication	Study design	Keywords	N	Population characteristics	Treatment	Outcomes
UK	sensitive nephrotic syndrome		sensitive Corticosteroi d Relapse Remission Steroid-sparing		**CsA:** n=61 as 2nd agent in SDNS following CPA treatment; 1st agent n=8<br>**Chlorambucil**: n=15 when other therapies failed<br><br>**Age at NS onset:** not stated<br>**FU:** 6.1 yrs (IQR 1-17.4) yrs<br>**Indications:** SDNS, FRNS	**LEV:** 2.5 mg/kg on alternate days<br>**Duration**: 3.2 (1-7) yrs<br><br>**CsA:** 3-5 mg/kg in 2 divided doses (12htrough level 50-150 µg/l)<br><br>**Other:** all: oral steroids in tapering dose	18/178 (10%)<br>**LEV:** sustained remission at 1 yr 19/65 (30%) as 1st agent, relapse after stop of LEV 4. 32/48 (66%) with sustained remission when used after unsuccessful CPA<br>**CsA:** 43/61 (70%) with sustained remission; relapse following discontinuation 32 (51%).<br>**CHL:** sustained remission at 1 yr: 7.<br>**AE:**<br>**CPA:** neutropenia 16/178, requiring interruption of med., serious infection 4/178<br>**LEV:** reversible neutropenia 4, skin rash 2 (requiring end of treatment)<br>**CsA:** nephrotoxicity 4<br>**CHL:** not stated
Chen SY 2010 [90] Taiwan	Treatment course of steroid-dependent nephrotic syndrome: emphasized on treatment effect	Retrospectiv e single center study	Chlorambucil Cyclophosph amide Cyclosporine Levamisol	Total 46, LEV:1 5	**LEV:** n=15<br>**CHL:** n=22<br>**CsA:** n=8<br>**2nd CPA:** n=6<br><br>**Age at NS onset: 4**.5 (1-15.5) yrs<br>**Age at medication:** not stated<br>**FU:** 96 (22-244) mths<br>33M, 13 F<br>**Indications:** SDNS despite one course of CPA as 1st line agent	**LEV:** 2-3.3 mg/kg/day for 3-20 mths<br>**CHL:** 0.1-0.2 mg/kg/d for 8 weeks<br>**CsA:** 3.5-5 mg/kg/d for 6-14 mths, then tapering for 12-23 mths<br>**2nd CPA:** 2-3 mg/kg/day for 8 wks<br><br>**Other:** oral steroids in tapering dose	**Relapses after 1st CPA course:** 25/46<br>**Relapses after additional treatment**:<br>LEV: 1/15 remission, 1 with SSNS relapses, 1 relapse-free period, 1 LEV dependency, 10 no response<br>CHL: 7/22 complete remission, 5 with relapses, 4 disease-free for 6.8 (2-11) mths, 3 no response<br>CsA: 1/8 rem., 1 eith SSNS relapses, 1 disease-free period, 4 CsA dependency<br>2nd CPA: 3/6 disease-free period, 1 decreased steroid threshold, 2 no response<br>**AE:** not stated
**TAC – LEV - MMF**							
Basu B [91]	Long-term efficacy	Retrospectiv	Nephrotic	Total	**LEV:** n=129	**LEV:** 2.5 mg/kg on	**Change in relapse rate at 12**

1st author, year, country of origin	Title of Publication	Study design	Keywords	N	Population characteristics	Treatment	Outcomes
2017<br>India	and safety of common steroid sparing agents in idiopathic nephrotic syndrome	e cohort study	Syndrome<br>Mycophenolate mofetil<br>Levamisol<br>Tacrolimus	340,<br>**TAC 81**	**Age at NS onset:** 76.8 ± 32.0 mths<br>**Age at LEV:** not stated<br>**Duration of NS:** 3.3 ± 2.1 yrs<br>**FRNS/SDNS**: 78/51<br><br>**MMF:** n=130<br>**Age at NS onset:** 85.2 ± 28.8 mths<br>**Duration of NS:** 3.4 ± 2.6 yrs<br>**FRNS/SDNS**: 74/56<br><br>**TAC:** n=81<br>**Age at NS onset**: 79.2 ± 26.2 mths<br>**Duration of NS**: 3.1 ± 1.6 ysr<br>**FRNS/SDNS**: 47/34<br><br>**FU:** at least 30 mths<br>210M, 130F<br>**Indications:** SDNS (preferred MMF or TAC), FRNS (preferred LEV or MMF), no previous exposure to steroid-sparing agents	alternate days<br>Or<br>MMF: 1200 mg/m^2 daily<br>Or<br>Tacrolimus 0.1-0.2 mg/kg/d<br><br>**Duration:** steroid-sparig agent continued for 1 yr following complete stop of steroids<br>**Other:** oral steroids in tapering dose	**mths from previous year:**<br>LEV: -3.1 ± 1.1<br>MMF: -4.5 ±1.3<br>TAC: -5.1 ± 1.3<br>All p<0.001 relative to pre-study period.<br>**RR at 12 mths and 24 mths** (after stop of agent)<br>LEV: 1.7/yr; 2.8/yr<br>MMF: 0.9/yr; 1.4/yr<br>TAC: 0.9/yr; 1.8/yr<br>**Relapse-free survival at 30 mths:**<br>**TAC vs. MMF:** 61.7 vs. 38.5%, p<0.001<br>**TAC vs. LEV:** 61.7% vs. 24%, p<0.0001<br>**Time to relapse:**<br>LEV: 21 days<br>MMF: 23 days<br>TAC: 26 days<br>**Cumulative predn. dose at 12 mths:**<br>**TAC vs MMF and LEV: 82.7** ± 26.4 mg/kg/yr vs. 136.8±65.4 mg/kg/yr and 108.8 ± 35.7 mg/kg/yr (p<0.001)<br>**Cum. pred. dose between 18-30 mths:**<br>**MMF vs. TAC and LEV:** 74.4 mg/kg/yr vs. 96.4 mg/kg/yr (p=0.004); 74.4 vs. 117.6 mg/kg/yr (p<0.001).<br>**Predictors for relapse:**<br>SDNS vs. FRNS: HR 2.14 (95% CI 1.79-2.96, p<0.001)<br>**AE:**<br>LEV (n=3): malaria 1, transient mood changes 2

1st author, year, country of origin	Title of Publication	Study design	Keywords	N	Population characteristics	Treatment	Outcomes
							MMF (n=15): minor, temporary reduction of dose 3, acute resp. infec. 3, UTI 1, acute hepatitis 1, abdominal chronic pain 2, rec. vomiting 1, raised liver enzymes 1, muscle pain 1<br>TAC (n=33): temporary drug discontinuation 8, complete drug stop: 1<br>SAE: gram-pos. pneumonia with admission 2, gram-neg. peritonitis 1, pancreatitis 1<br>Minor: 5 acute resp. inf., gastroenteritis 4, UTI 2, herpes simplex 1, abscess 2, stomatitis 2, convulsion 1, alopecia 2, hirsutism 1, eczema 2, hyperglycemia 3, leukopenia 2
**CsA/Tac – CPA – LEV - MMF**							
Moorani 2019 [92] Pakistan	Immunosuppressive therapy in children with primary nephrotic syndrome: single center experience, Karachi, Pakistan	Retrospective chart review	Nephrotic syndrome<br>Minimal change disease<br>Oral prednisolone<br>Levamisole<br>Cyclophosphamide<br>Cyclosporin<br>Mycophenolate mofetil	130	**CPA:** n=90<br>**CsA:** n=88<br>**LEV:** n=55<br>**MMF:** n=39<br>**CsA+MMF:** n=20<br>**TAC+/-MMF** n=11<br><br>**Age at NS onset:** 4.78 ± 3.23 yrs<br>**Age at medication:** not stated<br>**FU:** not stated, at least 6 mths<br>**Indication:** SDNS (n=55), FRNS (n=75), steroid toxicity	**Sequential use:**<br>**CPA:** 2-3 mg/kg/d for 8-12 wks (2nd line)<br>**CNI:** (3rd line), dose not stated<br>**LEV:** 2-2.5 mg/kg on a.d. for 6-24 mths (1st line)<br>**MMF:** (3rd line): dose not stated<br><br>**Other:** oral steroids	**Remission (complete; partial) at 6 mths:**<br>CPA: 45/90; 13/90<br>CsA: 30/88; 18/88<br>LEV: effective 44/55<br>MMF: 4/39; 16/39<br>CsA+MMF: 4/20; 7/20, CsA dependent 9/20<br>**Outcome last FU:**<br>Compl. Rem. Off treatment: 65/130<br>Compl. Rem. ON treatment: 34/130<br>Partial rem. ON treatment: 12/130<br>**AE:**<br>CPA: severe infection (disseminated chicken pox 9, BMS 5, alopecia 3)<br>CsA: gum hyperplasia 5, hypertrichosis 6, renal dysfunction

1st author, year, country of origin	Title of Publication	Study design	Keywords	N	Population characteristics	Treatment	Outcomes
							7, deafness 1<br>LEV: pancytopenia 1, allergic rash 1<br>MMF: none

Table S8.2: Alkylating agents - Cyclophosphamide/ Chlorambucil

1st author, year, [Ref.] country of origin	Title of Publication	Study design	Key words	N	Population Characteristics	Treatment	Outcomes
**ALKYLATING AGENTS – Cyclophosphamide (CPA)/ Chlorambucil (CHL)**							
Latta 2001 [93]<br>Germany	A meta-analysis of cytotoxic treatment for frequently relapsing nephrotic syndrome in children	Meta-analysis of observational studies of efficacy and harms	NS<br>FRNS<br>SDNS<br>CYC<br>CHL<br>AE	866<br><br><br><br>638	38 articles on Rx of 866 pts given CYC (902 courses) and 638 given CHL (671 courses). Includes all articles published between 1960 & 2000.<br>65 articles on AE<br>AE: 38 articles; 1504 pts; 1573 courses of CYC or CHL	Protocols varied. Most started CYC or CHL after remission with Pred.<br>Cumulative doses:<br>CYC 105 - 588 mg/kg<br>CHL 5.6 – 32.8 mg/kg	**Remission rates** 0% at 12 mths to 90% at 5 yrs with CYC/CHL<br>**Overall relapse-free survival** 50% after 4 yrs with CPA/CHL<br>FRNS: 72% at 2 yrs; 36% at 5 yrs<br>SDNS: 40% at 2 yrs; 24% at 5 yrs<br>**AE: CYC:** Death 0.8%, hair loss 17.8%, low WBC 32.4%, low PLTs 2.1%, Infections 1.5%, Malignancies 0.2%, haemorrhagic cystitis 2.2%<br>**AE: CHL:** Death 1.1%, hair loss 2.1%, Low WBC 33%, low PLTs 5.9%, infections 6.3%, malignancies 0.6%, seizures 3.4%, haemorrhagic cystitis 0%<br>Total dose & duration negatively correlated with sperm count.<br>Avoid repeat courses CYC & dose >17 & total dose > 168 mg/kg
Vester 2003 [94]<br>Germany	Cyclophosphamide in steroid sensitive nephrotic syndrome: outcome and outlook	Retrospective single centre study	NS<br>MCD<br>FRNS<br>SDNS<br>CYC<br>Remission	106	**Age at NS onset:** 5.3±3.2yrs<br>**Age at CYC: 7.3**±3.8yrs<br>FU**: 5.9**±4.8yrs<br>71M;35F<br>**Indication:** FRNS. SDNS, No prior therapy with cytotoxics;	CYC dose: 2.0±0.2 mg/kg/d; cumulative dose 165±33 mg/kg; duration 83±15 days<br>Pred: QOD tapered	**Sustained remission:**<br>1 yr: 44%<br>2 yr: 34%<br>10 yr: 24%<br>**FRNS**: 54% at 5 yr<br>**SDNS**: 17% at 5 yr<br>**Age** < 5.5 yrs 80% relapsed in 1 yr<br>No diff in % sustained remission with total dose < or > 168 mg/kg BUT 45% who received >5040 mg/m^2 vs 11% who received <5040 mg/m^2 remained in remission.<br>Those with WBC < 3000, more

1st author, year, [Ref.] country of origin	Title of Publication	Study design	Key words	N	Population Characteristics	Treatment	Outcomes
							likely to remain in remission.
Kyrieleis 2007 [95]<br>The Netherlands	Long-term outcome after cyclophosphamide treatment in children with steroid-dependent or frequently relapsing minimal change nephrotic syndrome	Retrospective single centre study	NS<br>MCD<br>FRNS<br>SDNS<br>CYC<br>Remission	93	**Age at NS onset:** Median 3 yrs (range 1-14 yrs)<br>**Age at CYC:** Not stated<br>FU: Median time 8 yrs (1-39 yrs)<br>No FU available in 13.<br>**Indication:** Biopsy proven MCD receiving CYC from 1971-2003. FRNS, SDNS	CYC dose: 3mg/kg x 8 wks	**No relapse:** 33 (35%); median FU 6 yr (2-27 yr) after CYC<br>**≤ 5 relapses**: 19; 3 given CSA<br>**>6 relapses**: 28; 18 given CSA<br>**Cumulative remission**:<br>35% at 2 yrs; 52% at 6 yrs; 71% at 15 yrs<br>Age < 3 yrs & Males associated with more relapses<br>23 continued with relapses at last FU; most in group with > 6 relapses. 29% with relapses 15 yrs + after CYC. 25% had relapses as adults after CPA.
Azib 2011 [96]<br>France	Cyclophosphamide in steroid dependent nephrotic syndrome	Retrospective single centre study	NS<br>SDNS<br>CYC<br>Remission<br>AE	90	**Age at NS onset**: Median 3.2 yrs (IQR 2.4-4.7)<br>**Age at CYC**: Median 5.3 (IQR 3.2-9.1)<br>FU: 5.5 yrs (3.2-8.5 yrs)<br>67M, 23F<br>**Indication**: SDNS. 39 had received LEV, 1 CSA	CYC 2 mg/kg x 10-12 wks after remission (single course). Cumulative dose 160 (149-170) mg/kg<br>Pred: Reduced over 6 mths; some remained on low dose qod	**Sustained remission:**<br>1 yr; 57% (95% CI 47-68)<br>2 yr: 42% (95% CI 32-53)<br>5 yr: 31% (95% CI 21-41)<br>**Pred** ceased in 45 over median 0.9 yrs<br>**FU**: No further IS needed 26 (46%), 23 needed CNI, 25 MMF, 9 RTX.<br>Age at CYC > 7.5 yrs associated with sustained remission<br>**AE**: Leucopenia 4, alopecia 1

1st author, year, [Ref.] country of origin	Title of Publication	Study design	Key words	N	Population Characteristics	Treatment	Outcomes
Zagury 2011 [97]<br>Brazil	Long-term follow up after cyclophosphamide therapy in steroid-dependent nephrotic syndrome	Retrospective single centre study	NS<br>SDNS<br>CYC<br>Remission	108	**Age at NS onset**: Median 2.95 yrs (range 1.1-14.1)<br>**Age at CYC**: 4.92 yrs (range 2-15)<br>**FU**; median 9.5 yrs (5-29); all ≥ 5 yrs<br>69M, 39F<br>**Indication**: SDNS. No previous therapy other than prednisone	CYC: Mean dose 2.41±0.32 mg/kg/d (in group with sustained remission (>5yrs)<br>2.45±0.34 mg/kg/d in group without sustained remission)<br>Max dose 168 mg/kg<br><br>Pred for relapse: 2 mg/kg/d x 4 wks, QOD for 4 wks then 25% reduction every 2 wks	**Remission: 2 yr**: 34%; 5 yr: 25%; 10 yr: 22%<br>**Sustained remission > 5 yrs** in 27/108.<br>**Sustained remission:** Pred dose at relapse was 0.96 ± 0.51 mg/kg<br>**No sustained remission:** Pred dose at relapse was 1.29 ± 0.59 mg/kg<br>Median CYC doses (mg/kg) did not differ<br>Higher sustained remission in children aged ≥ 7 yrs at CPA & age at NS onset ≥ 2.2 yrs<br>**AE**: No information
Cammas 2011 [98]<br>France<br>Algeria	Long-term effects of cyclophosphamide therapy in steroid-dependent or frequently relapsing idiopathic nephrotic syndrome	Retrospective multi-centre study	NS<br>FRNS<br>SDNS<br>CYC<br>Remission	143	**Age at NS onset:** median 3.7 yrs (IQR 2.3-5.9)<br>**CYC** started after median 4 relapses (IQR 3-8) & median 1.7 yr (IQR 0.7 – 5.9) after onset<br>**FU:** 88 had other Rx before or after CYC<br>**Indication:** FRNS, SDNS in 75%; steroid toxicity 25%**.** Those treated with another alkylating agent previously were excluded	CYC: 2-2.5 mg/kg/day for 10-12 wks<br>Pred for relapse<br>Sustained remission defined as 2+yrs	**Median time to pred withdrawal**: 10 mths (IQR 3-34)<br>**Ongoing remission**: 20.5% after median 1.9 yrs<br>**Relapse free survival (KM analysis)**<br>1 yr: 45% (CI 35-55)<br>2 yr: 28% (CI 15-41)<br>5 yr: 13% (CI 0-26)<br>10 yr: 11% (CI 0-22)<br>Age < 5 yr: higher risk of relapse<br>**Median time without relapse**: FRNS 15 mths; SDNS 8 mths<br>**Predictors of sustained remission**:<br>CYC dose >170 mg/kg; age > 5 yrs; Female; FRNS<br>**AE:** No Information
Bajeer<br>2018 [99]<br>Pakistan	Histological spectrum and short-term outcome of treatment with cyclophosphamide	Retrospective single centre study	NS<br>SSNS<br>Relapse<br>Kidney biopsy<br>Remission	74	**Age at NS onset:** Median 5 yr (IQR 4-7)<br>**Start of CYC**: median 17 mths (13-27) after NS onset	CYC 2-2.5 mg/kg/d (max 180 mg/kg)<br>Relapsing NS defined as >2 relapses/yr.	**Remission at 1yr:** 41 (55%) (rest CNI dependent).<br>**Remission at 2 yrs**: 27 (37%)<br>**Duration of Remission**:<br>MCD (54): 12 mths (5.5-22)

1st author, year, [Ref.] country of origin	Title of Publication	Study design	Key words	N	Population Characteristics	Treatment	Outcomes
	in relapsing steroid-sensitive nephrotic syndrome				**FU:** 2 yrs after CYC<br>47M; 27F<br>**Indication:** Patients 2012-2014 with relapsing SSNS with biopsy. Excluded adolescent, infantile NS, previous use of non-steroid IS, loss to FU	Pred 1 mg/kg qod	FSGS (13): 20.4 ± 8.4 mths<br>MesPGN (6): 15.6 ± 9.8 mths<br>**AE:** leucopenia 6 (all able to complete CYC);<br>anaemia 1; alopecia 1.
**Studies with CPA – comparing different steroid-sparing agents**							
**CPA vs. CsA**							
Sümegi 2008 [88]<br>Hungary	Long-term follow-up after cyclophosphamide and cyclosporine-A therapy in steroid-dependent and –resistant nephrotic syndrome	Retrospective study	Cyclosporine A, cyclophosphamide, immunosuppressive therapy, steroid reistant, steroid dependent	37, of those SDNS 23	**CPA:** n=22, of those SDNS n=15<br>**CsA:** n=15, of those SDNS n=8<br><br>**Age at NS onset:**<br>CPA: 7.7 ± 3.8 yrs<br>CsA: 9.5 ± 4.7 yrs<br>**FU:** 7.1 (5-13) yrs<br>25 M, 12 F<br>**Indications:** SDNS, SRNS	**CPA:** 2-2.5 mg/kg/d<br>Duration: 2.5 ± 0.5 mths<br>**CsA:** 3-5 mg/kg/day, trough levels 100-200 ng/ml<br>**Duration:** 28 ± 15 mths<br><br>**Other:** oral steroids in tapering dose	**Remission at 5 yrs:**<br>CPA: 20/22 (90.9%)<br>CsA: 10/15 (66.6%)<br>**RR:** decreased in both groups:<br>CPA: from 3.4±2.8/yr to 0.1±0.2/yr<br>CsA: from 3.7±3.1 to 0.6±0.8/yr<br>**Time to relapse:**<br>CPA: 29.9 ± 21.5 mths vs. CsA: 28.1 ± 22.4 mths<br>**AE:**<br>CPA: nausea 3/22, reversible hair loss 2/22, leucopenia 1/22, alopecia 1/22<br>CsA: hirsutism 3/15, tremor 2/15, gingival hyperplasia 2/15, nausea and appetite loss 1/15
**CPA vs. LEV**							
Alsaran K 2001 [100]<br><br>Only abstract available	Levamisole vs. cyclophosphamide for frequently relapsing steroid-dependent syndrome	Retrospective analysis	SDNS<br>Levamisol<br>cyclophosphamide<br>Children<br>relapse	Total 51, LEV: 24	**LEV** n=24<br>**CPA** n=27<br><br>**Age at NS onset:**<br>**Age at LEV:**<br>**FU:**<br>**Indications:**	**LEV:**<br>**CPA:**<br><br>**Duration:**<br>**Other:**	**RR:** Reduced by 0.28 relapses/patient/year **(LEV)** and 0.32 relapses/pt-/yr **(CPA)**<br>**Cumulative dose of pred.:** reduced by 336mg/m^2/mth **(LEV)** and 387 mg/m^2/month **(CPA).**<br>**AE:**
**CPA – LEV - CsA**							
Abeyagunawardena 2003 [89]<br>UK	The use of steroid-sparing agents in steroid-sensitive nephrotic syndrome	Retrospective cohort study	Nephrotic syndrome<br>Steroid sensitive		**CPA**: n=178 (1st agent)<br>**LEV**: n=113, of those 1st agent n=65<br>**CsA:** n=61 as 2nd agent in	**CPA:** 3 mg/kg daily<br>**Duration:** 8 wks<br><br>**LEV:** 2.5 mg/kg on	**CPA:** sustained remission at 1 yr: 94/178 (54%), at 2 yrs: 44%, at 5 yrs: 32%, 2nd course of CPA: 18/178 (10%)

1st author, year, [Ref.] country of origin	Title of Publication	Study design	Key words	N	Population Characteristics	Treatment	Outcomes
			Corticosteroid<br>Relapse<br>Remission<br>Steroid-sparing		SDNS following CPA treatment; 1st agent n=8<br>**Chlorambucil**: n=15 when other therapies failed<br><br>**Age at NS onset:** not stated<br>**FU:** 6.1 yrs (IQR 1-17.4) yrs<br>**Indications:** SDNS, FRNS	alternate days<br>**Duration**: 3.2 (1-7) yrs<br><br>**CsA:** 3-5 mg/kg in 2 divided doses (12htrough level 50-150 µg/l)<br><br>**Other:** all: oral steroids in tapering dose	**LEV:** sustained remission at 1 yr 19/65 (30%) as 1st agent, relapse after stopf of LEV 4. 32/48 (66%) wigh sustained remission when used after unsuccessful CPA<br>**CsA:** 43/61 (70%) with sustained remission; relapse following discontinuation 32 (51%).<br>**CHL:** sustained remission at 1 yr: 7.<br>**AE:**<br>**CPA:** neutropenia 16/178, requiring interruption of med., serious infection 4/178<br>**LEV:** reversible neutropenia 4, skin rash 2 (requiring end of treatment)<br>**CsA:** nephrotoxicity 4<br>**CHL:** not stated
Chen SY<br>2010 [90]<br>Taiwan	Treatment course of steroid-dependent nephrotic syndrome: emphasized on treatment effect	Retrospective single center study	Chlorambucil<br>Cyclophosph amide<br>Cyclosporine<br>Levamisol	Total 46, LEV:15	**LEV:** n=15<br>**CHL:** n=22<br>**CsA:** n=8<br>**2nd CPA:** n=6<br><br>**Age at NS onset:** 4.5 (1-15.5) yrs<br>**Age at medication:** not stated<br>**FU:** 96 (22-244) mths<br>33M, 13 F<br>**Indications:** SDNS despite one course of CPA as 1st line agent	**LEV:** 2-3.3 mg/kg/day for 3-20 mths<br>**CHL:** 0.1-0.2 mg/kg/d for 8 weeks<br>**CsA:** 3.5-5 mg/kg/d for 6-14 mths, then tapering for 12-23 mths<br>**2nd CPA:** 2-3 mg/kg/day for 8 wks<br><br>**Other:** oral steroids in tapering dose	**Relapses after 1st CPA course:** 25/46<br>**Relapses after additional treatment**:<br>LEV: 1/15 remission, 1 with SSNS relapses, 1 relapse-free period, 1 LEV dependency, 10 no response<br>CHL: 7/22 complete remission, 5 with relapses, 4 disease-free for 6.8 (2-11) mths, 3 no response<br>CsA: 1/8 rem., 1 with SSNS relapses, 1 disease-free period, 4 CsA dependency<br>2nd CPA: 3/6 disease-free period, 1 decreased steroid threshold, 2 no response<br>**AE:** not stated
**CPA – CsA – MMF – LEV - AZA**							
Moustafa BH 2016 [101]	Immunosuppressive therapy in children	Retrospective chart review	Childhood Nephrotic	79	**CPA:** n=28<br>**CsA:** n=6	**CPA:** 2-3 mg/kg/d orally for 8-12 wks	**Remission for 6 mths:**<br>**CPA:** 24/28 (85.7%)

1st author, year, [Ref.] country of origin	Title of Publication	Study design	Key words	N	Population Characteristics	Treatment	Outcomes
Egypt	with steroid-resistant, frequently-relapsing, and steroid-dependent idiopathic nephrotic syndrome: a single center experience	Single center	Syndrome<br>Steroid<br>Resistance<br>Steroid<br>Dependence<br>Relapse<br>immunosuppr essants		**MMF:** n=2<br>**LEV:** n=40<br>**AZA:** n=10<br><br>**Age at NS onset:** 3.7 (1.3-10.5) yrs<br>**Age at medication:** not stated<br>**FU:**<br>44 M, 35 F<br>**Indications:** SDNS/ FRNS, steroid toxicity	or IV as monthly bolus of 500-750 mg/m^2 for 6 mths<br>**CsA:** 4-6 mg/kg/d divided into 2 doses for at least 12 mths<br>**MMF:** 1200 mg/m^2/day divided into doses<br>**LEV:** 2-2.5 mg/kg/dose twice weekly for 6-24 mths<br>AZA: 2 mg/kg/d for 8 wks.<br><br>Either given as 1st or 2nd line drug; some given in double- or triple-combination therapy<br><br>**Other:** oral steroids	**CsA:** 5/6 (83.3%)<br>**MMF:** ½ (50%)<br>**LEV:** 22/40 (55%)<br>**AZA:** 8/10 (80%)<br><br>**AE** (not differentiated between SDNS (n=79) and SRNS (n=51)):<br>CPA: Leukopenia 15/63 (23.8%), hemorrhagic cystitis 2/63 (3.2%)<br>CsA: gym hyperplasia 8/31 (25.8%), hirsutism 7/31 (22.6%), nephrotoxicity 2/31 (6.4%), hypertension 2/31 (6.4%)<br>MMF: diarrhea 7/12 (58.4%), nausea 3/12 (25%), abdominal pain 1 (8.%), cough 1/12 (8.3%)<br>LEV: none<br>AZA: Leukopenia 2/10, diarrhea 2/10, abdominal pain 2/10, arthralgia 1/10
**CsA/Tac – CPA – LEV - MMF**							
Moorani 2019 [92]<br>Pakistan	Immunosuppressive therapy in children with primary nephrotic syndrome: single center experience, Karachi, Pakistan	Retrospective chart review	Nephrotic syndrome<br>Minimal change disease<br>Oral prednisolone<br>Levamisole<br>Cyclophosph amide<br>Cyclosporin<br>Mycophenolat e mofetil	130	**CPA:** n=90<br>**CsA:** n=88<br>**LEV:** n=55<br>**MMF:** n=39<br>**CsA+MMF:** n=20<br>**TAC+/-MMF** n=11<br><br>**Age at NS onset:** 4.78 ± 3.23 yrs<br>**Age at medication:** not stated<br>**FU:** not stated, at least 6 mths<br>**Indication:** SDNS (n=55), FRNS (n=75), steroid toxicity	**Sequential use:**<br>**CPA:** 2-3 mg/kg/d for 8-12 wks (2nd line)<br>**CNI:** (3rd line), dose not stated<br>**LEV:** 2-2.5 mg/kg on a.d. for 6-24 mths (1st line)<br>**MMF:** (3rd line): dose not stated<br><br>**Other:** oral steroids	**Remission (complete; partial) at 6 mths:**<br>CPA: 45/90; 13/90<br>CsA: 30/88; 18/88<br>LEV: effective 44/55<br>MMF: 4/39; 16/39<br>CsA+MMF: 4/20; 7/20, CsA dependent 9/20<br>**Outcome last FU:**<br>Compl. Rem. Off treatment: 65/130<br>Compl. Rem. ON treatment: 34/130<br>Partial rem. ON treatment: 12/130<br>**AE:**

1st author, year, [Ref.] country of origin	Title of Publication	Study design	Key words	N	Population Characteristics	Treatment	Outcomes
							CPA: severe infection (disseminated chicken pox 9, BMS 5, alopecia 3)<br>CsA: gum hyperplasia 5, hypertrichosis 6, renal dysfunction 7, deafness 1<br>LEV: pancytopenia 1, allergic rash 1<br>MMF: none

Table S8.3: Mycophenolate Mofetil (MMF)/Mycophenolate Sodium (MPS)

1st author, year, [Ref.], country of origin	Title of Publication	Study design	Key words	N	Population Characteristics	Treatment	Outcomes
**Mycophenolate Mofetil (MMF)/Mycophenolate Sodium (MPS)**							
Bagga 2003 [102]<br>India	Mycophenolate Mofetil and prednisolone therapy in children with steroid-dependent nephrotic syndrom	Prospective analysis<br>single center	MMF<br>Prednisolone<br>Minimal change disease<br>Focal segmental glomerulosclerosis	19	**Age at NS onset:** 35.6 (13-92) mths<br>**Age at MMF:** 99.1 (32-134) mths<br>**Duration of NS before MMF**: 53.5 (10-94) mths<br>**FU:** 17 (14-20) mths<br>13M, 6F<br>**Indication:** SDNS despite prior LEV (n=16), CPA (n=15), steroid toxicity (cushingoid features: all, growth retardation: 7)	**MMF**: 29 mg/kg/d (20.8-33.3) in 2 divided doses<br>**Duration:** 11.8 (9-12.8 mths)<br>**Other:** oral steroids in tapering dose	**RR:** decreased from 6.6 (95% CI, 5.4-7.7) to 2 (95% CI, 1.2-2.7)/yr (p<0.0001)<br>> 50% reduction in RR in 14/19<br>Increase of RR to 4.2/yr (95% CI, 2.8-5.5) after MMF discontinuation<br>**Steroid dose:** reduction from 0.7 (95% CI, 0.6-0.8) to 0.3 (95% CI, 0.2-0.4) mg/kg/d (p<0.0001)<br>**MMF treatment failure:** 3/19<br>**AE:** None relevant
Novak 2005 [103]<br>USA	Efficacy of mycophenolate mofetil in pediatric patients with steroid-dependent nephrotic syndrome	Retrospective chart review<br>Single centre	NS<br>SDNS<br>Relapse<br>MMF<br>AE	21	**Age at NS onset:** 3.9 ± 3.1yrs.<br>**Age at MMF** 8.2 ± 4.4yrs<br>FU 1.9 yr ± 1.0 yrs<br>18M, 3F<br>**Indication**: SDNS 17; steroid toxicity 4	**MMF** 600 mg/m² bd, max 1000 mg bd<br>**Duration:** 1.0 ± 0.5 yrs (0.2-2 yrs)<br>**Other**: steroids 20 for relapse, CSA 1	**RR**: 0.80 ± 0.41 fell to 0.47± 0.43/mth<br>**Time to relapse**: 3.8±4.2 mths<br>> 50% reduction in RR in 12 pts<br>**AE**: Infection 1, GIT transient upset 2
Mendizabal 2005 [104]<br>Spain	Mycophenolate mofetil in steroid/cyclosporine-dependent/resistant nephrotic syndrome	Prospective cohort study	Mycophenolate mofetil<br>nephrotic syndrome<br>FSGS<br>MCGN<br>Children<br>Cyclosporine	21	**Age at NS onset:** 2.8 (1.2-12.5) yrs<br>**Age at MMF:** 11.4 (5-17) yrs<br>**FU:**<br>**Indication:** SDNS, despite CPA, CsA	**MMF** 600 mg/m² bd, adjusted to maintain trough MPA levels at 2.5-5 µg/ml.<br>**Duration:** 6 mths<br>**Other:** Tapering dose of steroids	**Steroid sparing effect** on MMF**:** 15/21, of those 10 with complete withdrawal of steroids<br>**Probability of remaining in remission at 3 and 6 mths**: 76% and 51%<br>**Sustained remission** on MMF: 9<br>**Relapses on MMF:** 12 (23 relapses)<br>**Immediate relapse after MMF:** 7/15

1st author, year, [Ref.], country of origin	Title of Publication	Study design	Key words	N	Population Characteristics	Treatment	Outcomes
							**AE**: not stated
Hogg 2006 [105]<br>USA	Mycophenolate mofetil in children with frequently relapsing nephrotic syndrome: A Report from the Southwest Pediatric Nephrology Study Group	Multicentre prospective single arm study<br>13 centres	NS<br>FRNS<br>SDNS<br>Relapse<br>MMF<br>AE	32	**Age at NS onset:** Not stated<br>**Age at MMF:** 6.8 ± 2.7yrs<br>**FU**: Up to 30 mths<br>18M, 14F<br>No previous Rx with LEV, CSA, TAC<br>**Indication**: FRNS 26; SDNS 6	**MMF** 600 mg/m² bd, max 1000 mg bd<br>**Duration;** 28 wks<br>**Other**: Tapering dose of steroids	**Relapse** on MMF: 8<br>**Relapse** within 6 mths of stopping MMF: 12<br>**Relapse** > 6+ mths after ceasing MMF: 12<br>**AE:** leucopenia 5 (1 ceased MMF), GIT upset 1,
Afzal 2007 [106]<br>India	Treatment with mycophenolate mofetil and prednisolone for steroid-dependent nephrotic syndrome	Retrospective chart review of patients who received MMF for ≥ 6 mths<br>Single centre	NS<br>SDNS<br>Relapse<br>MMF<br>AE	42	**Age at NS onset**: 3.1 yrs (range 1.1-7.7 yrs)<br>**Age at MMF**: 8.8 yrs (range 2.7 -15.6 yrs)<br>**FU:** Up to 45 mths<br>27M, 15F<br>**Indication**: SDNS despite LEV (6 mths) &/or CPA (12 wks)	**MMF** 27 mg/kg/d (range 17-31 mg/kg/d)<br>**Duration**: 1.2 yrs (range 0.5-3.8 yrs)<br>**Other**: Tapering dose of steroids	**RR**: 6.0 (95% CI 5.2-6.7) fell to 2.2 (95% CI 1.4-2.9) per pt/yr<br>**Pred dose**: 0.6 mg/k/d (95% CI 0.5-0.7) fell to 0.3 mg/kg/d (95% CI 0.3-0.4)<br>**AE**: Transient GIT upset 9. Infections 4
Fujinaga 2009 [107]<br>Japan	Mycophenolate mofetil therapy for childhood-onset steroid dependent nephrotic syndrome after long term cyclosporine: extended experience in a single centre		NS<br>SDNS<br>Relapse<br>MMF<br>MPA levels<br>AE	26	**Age at NS onset:** 5.1 ± 3.3 yrs<br>Age at MMF: 13.1 ± 4.1 yrs<br>**FU:** Mean FU 19mths (7-42 mths)<br>19M, 7F<br>**Indication:** SDNS (19 MCD, 7 FSGS) & received MMF for ≥6mths. Pts had received CSA for mean 56 mths. Used ISKDC definitions.	**MMF** 250 mg/12 hr increased to 1 gm/12 hr adjusted for pre-dose MPA level of 2-5 µg/ml. Mean dose 34 ± 6 mg/kg<br>**Duration**: Mean FU 19mths (7-42 mths)<br>**Other**: CSA tapered, MZR discontinued, steroids for relapse	**RR: 2.5** ± 1.4 fell to 0.8 ± 1.2/pt/yr<br>**Pred & CSA**: Tapered in 20; 15 off CSA; 11 off pred. CSA reintroduced/increased in 6.<br>**MPA levels:** < 3 µg/ml more likely to relapse<br>**AE:** Anaemia 1, herpes labialis 1. GIT upset 0
Baudouin 2012 [108]<br>France	Mycophenolate mofetil for steroid dependent nephrotic syndrome: a phase	Open single-arm phase II prospective study	NS<br>SDNS<br>Relapse<br>Height	23	**Age at NS** onset: median 2.1 yrs (range 2.0-3.5)<br>**Age at MMF**: median 6.0 yrs (range 6.9-11.0)	**MMF** 600mg/m²/d for 7 days & then 1200mg/m²/d in 2 doses.	**Relapse** in 6 mths: 4<br>**Relapse** in 12 mths: 2<br>**No Relapse** in 12 mths:17<br>**Median pred dose**: Fell

1st author, year, [Ref.], country of origin	Title of Publication	Study design	Key words	N	Population Characteristics	Treatment	Outcomes
	II Bayesian trial	Nine centres	Weight<br>MMF<br>AE		**FU**: 12 months<br>19M, 5F<br>**Indication**: SDNS despite previous CPA (12 wks) or CHL (40 days); not on TAC, CSA, LEV	**Duration**: 1 year<br>**Other**: Steroids to treat relapse, then tapered to 7.5 mg/m^2/d	from 25mg/m^2/d to 9mg/m^2/d at 6 mths in 19 without relapse<br>**Weight** fell by 1 SDS; no change in height SDS<br>**AE**: transient GIT upsets 6, mild infections 18, reduced dose for anaemia 1
Banerjee 2013 [109]<br>India	Outcome of severe steroid-dependent nephrotic syndrome treated mycophenolate mofetil	Multicentre observational study<br>Four centres	NS<br>SDNS<br>Relapse<br>MMF<br>AE	46	Age at NS onset: 2.2 ± 0.97 yrs<br>Age at MMF: 6.57 ± 3.0 yrs<br>FU: 3.56 ± 1.76 yrs<br>30M, 16F<br>**Indication**: SDNS requiring > 0.5 mg/kg Pred alt day despite LEV (6 mths), CPA (8-12 wks)	**MMF** 20-30 mg/kg/d in 2 doses after remission<br>**Duration**: 12 mths<br>**Other**: Steroids tapered over 6 mths	**Remission:** pred ceased ≥ 1 mth: 20<br>**Remission:** pred reduced to < 0.5 mg/kg qod: 12<br>**Relapse** when pred < 0.5 mg/kg/d: 14<br>**AE**: Transient GIT upsets 3 Neutropenia 1, Elevated transaminases 1
Dehoux 2016 [110]<br>France	Mycophenolate mofetil in steroid-dependent idiopathic nephrotic syndrome	Retrospective single centre study	NS<br>SDNS<br>Relapse<br>MMF<br>AE	96	**Age at NS onset**: median 3.1 yrs (IQR 2.3-4.1)<br>**Age at MMF**: median 7.3 yrs(IQR 4.2-10.4)<br>**FU**: Median 4.7 yrs (IQR 3.0-6.0)<br>71M, 25F<br>**Indication**: SDNS in pts not on other immunosuppressives; no previous treatment with RTX	**MMF**: 600 mg/m^2 for 7 days, then 1200 mg/m^2/dose in 2 doses.<br>**Duration**: Median 32 mths (IQR 22-46 mths)<br>**Other**: Pred ceased in 49/96 after median 18.1 mth (IQR 7.8-30.0 mths)	**Remission**: Median relapse free survival 15 mths (IQR 5.6 – 37.1 mths)<br>**Relapse** when pred ceased 22<br>Responders (≥50% fall in RR &/or ≥60% fall in cumulative pred dose in 1 yr): 74<br>Non-responders: 22. Non-responder older, slower to remission in 1st episode, more relapses pre MMF<br>**Last FU**: 58/96 in remission without treatment<br>**AE**: Transient GIT upset 6; severe GIT upset 5 (I ceased MMF). Leucopenia 2. Depression 1 (MMF ceased)
Kapoor 2017 [111]<br>India	Mycophenolate sodium in children	Retrospective single centre	NS<br>FRNS	40	**Age at NS onset:** Not stated	**EC-MPS:** Mean dose at start 796 ±	**Remission:** 30 had CR; 2 PR; 2 no response at 6

1st author, year, [Ref.], country of origin	Title of Publication	Study design	Key words	N	Population Characteristics	Treatment	Outcomes
	with frequently relapsing or steroid dependent nephrotic syndrome	study	SDNS<br>EC-MPS<br>MPA<br>AE		**Age at EC-MPS**: 7.52 ± 3.38 yrs<br>**FU:** Unclear<br>**Gender:** Not stated<br>**Indication:** FRNS or SDNS with/without previous treatment with LEV, CPA	157 mg/m^2/day of MPA equivalent. Mean maximum dose 870 ± 191 mg/m^2/day of MPA equivalent. Duration: 17 treated for > 2 yrs	mths. 17 treated for > 2 yrs<br>**RR**: improved from 1 every 3 mths to 1 every 14.6 mths<br>**Pred** ceased in 20 without relapse.<br>**AE**: Sepsis 1
Nandi 2019 [112]<br>India	Efficacy of mycophenolate mofetil as a remission maintaining agent in idiopathic childhood nephrotic syndrome	Prospective single centre study	NS<br>FRNS<br>SDNS<br>Relapse<br>MMF	32	**Age at NS onset:** 2.72 ± 1.3 yrs<br>**Age at MMF:** 7.17 ± 2.2 yrs<br>**FU:** Unclear<br>22M, 10F<br>**Indication**: FRNS 6, SDNS 26 requiring continuous medication for ≥12 mths (pred, LEV, CPA)	**MMF**: 1000-1200 mg/m^2/d; max dose 1000 mg in 2 doses<br>**Duration**: 28 wks<br>**Other**: Pred dose reduced	**RR**: 3.43 ± 1.26 fell to 1.62 ± 1.14/pt/yr<br>**Pred dose**: 190.9 ± 47.81 fell to 119.09 ± 60.09 mg/kg/yr<br>**AE**: Transient GIT upset 2
Karunamoorthy 2020 [113]<br>India	The safety and efficacy of mycophenolate mofetil in children and adolescents with steroid-dependent nephrotic syndrome: a single centre study	Retrospective single centre study	NS<br>FRNS<br>SDNS<br>Relapse<br>MMF<br>AE	87	**Age at onset of NS**: median 3 yrs (95% CI 1-8)<br>**Age at MMF**: median 7 yrs (95% CI 2-12)<br>**FU:** median 3 yr 6 mth (95% CI 1 yr 3 mth to 6 yrs 6 mths)<br>58M, 29F<br>**Indication**: FRNS 56, SDNS 31 who relapsed or did not achieve remission with IV CPA	**MMF**: 30 mg/kg in 2 doses. Pts included if treated with MMF ≥ 12 mths. MMF dose tapered after 2 yrs if sustained remission.<br>**Duration**: median 2 yrs 6 mths (95% CI 1 yr 3 mths to 6 yrs 6 mths)<br>**Other**: Pred duration unclear	**Remission**: 72 were MMF sensitive. Pred dose fell from 1.28 mg/kg to 0.35 mg/kg. 63 had 1 relapse, 16 had 2 relapses, 15 had 3+ relapses.<br>Of 31 pts on MMF for 2yrs without relapse, all relapsed when MMF ceased<br>**Treatment failure**: 15 continued frequent relapses on MMF<br>**AE**: Infection 13, low white count 3, transient GIT upset 2
**Studies with MMF – comparing different steroid-sparing agents**							
**MMF vs. TAC**							
Wang 2016 [85]<br>China	Evaluation of mycophenolate mofetil or	Prospective single center study	Children<br>Mycophenolate mofetil	72	**MMF Group:** n=34, completed protocol n=30<br>**TAC Group:** n= 38,	**MMF:** 20-30 mg/kg/d in 2 divided doses	**Remission at 12 mths:** MMF 24 (90%); TAC 31 (97%)

1st author, year, [Ref.], country of origin	Title of Publication	Study design	Key words	N	Population Characteristics	Treatment	Outcomes
	Tacrolimus in children with steroid sensitive but frequently relapsing or steroid-dependent nephrotic syndrome		Primary nephrotic syndrome<br>tacrolimus		complete study protocol n=35<br><br>**Age at NS onset:** 43.1 ± 25.6 mths (MMF), 50.8 ± 31.1 mths (TAC)<br>**Age at initiation:** 64.2 ± 32.2 mths (MMF), 72.2 ± 32.3 mths (TAC)<br>**FU:**<br>51 M, 21 F<br>**Indications:** FRNS, SDNS	(max. 1g)<br>**TAC:** 0.05-0.15 mg/kg/d in 2 divided doses; trough levels 5-10 ng/ml<br>**Duration:** 12 mths<br>**Other:** low-dose oral steroids	**RR at 6 and 12 mths:**<br>MMF: decreased from 2.56/6mths before MMF to 0.76/1st 6 mths and 0.67/2nd 6 mths (p<0.001)<br>TAC: decreased from 2.38/6mths to 0.41/ 1st 6 mths and 0.42/ 2nd 6 mths (p<0.001)<br>**Steroid dose: decreased from 0.61** ± 0.06 mg/kg/d (MMF)/ 0.66 ± 0.05 mg/kg/d (TAC) to 0.16 ± 0.02 mg/kg/d (MMF)/ 0.17 ± 0.03 mg/kg/d.<br>**AE:** MMF discontinued due to leucopenia/GIT symptoms and chickenpox 2/34; TAC discontinued due to severe infection/ refractory anemia 1 and neurologic symptoms 1/38; new onset of hypertension 2 (TAC).<br>Infections: 11.8% (MMF), 7.9% (TAC).<br>Reversible leucopenia (1 MMF, 1 TAC), acute kidney injury (1 MMF, no Tac)
**MMF vs. CsA – after RTX**							
Fujinaga S 2013 [86]<br>Japan	Cyclosporine versus Mycophenolate mofetil for maintenance of remission of steroid-dependent nephrotic syndrome after a single infusion of rituximab	Prospective study<br>Single center	Cyclosporine<br>Mycophenolate mofetil<br>Rituximab<br>Steroid-dependent nephrotic syndrome	29	**CsA after RTX:** n=13<br>**MMF after RTX:** n=16<br><br>**Age at onset of NS:** 6.4 ± 3.9 yrs<br>**Age at RTX:** 11.8 ± 4.3 yrs<br>**FU:**<br>19M, 10 F<br>**Indication:** severe SDNS	**RTX:** Single dose of 375 mg/m^2 (max. 500 mg)<br>**Duration of B-cell depletion**: 5 mths<br><br>**After RTX:**<br>**CsA group**: dose adjusted to C2 level of 400-500 ng/ml;	**Relapse after RTX:**<br>CsA: 3/13<br>MMF: 12/16<br>**Treatment failure:**<br>CsA: 2/13<br>MMF: 7/16<br><br>**RR:**<br>CsA: decrease from 4.4 ± 1.9 to 0.6±1.4/yr (86%,

1st author, year, [Ref.], country of origin	Title of Publication	Study design	Key words	N	Population Characteristics	Treatment	Outcomes
					despite CsA (for 49±35 mths) and/or MMF<br>Immunosuppr. agents at RTX: CsA 13, MMF 11, CsA and MMF 4, CsA and MZR 1	mean dose 3.7 mg/kg/d. MMF and MZR discontinued.<br>**Duration** after RTX: 18 (5-29) mths<br>**MMF group:** adjusted to target MPA levels of 2-5 µg/ml. CsA discontiinued<br>**Duration** after RTX**:** 19 (7-44) mths<br>**Other:** Tapering dose of steroids	p<0.01)<br>MMF: decrease from 2.3±0.8 to 1.0/±0.9yr (58%; p<0.01)<br><br>**Steroid dose:**<br>CsA: decrease from 0.35±0.16 to 0.057±0.14 mg/kg/day (p<0.01)<br>MMF: decrease from 0.38±0.26 to 0.15±0.21 mg/kg/d (p<0.01)<br><br>**Dose of agent after RTX:**<br>CsA: decrease from 4.6 to 3.7 mg/kg/d (21%, p<0.01).<br>MMF:<br><br>**AE:**<br>RTX: transient infusion reaction: 13/29 (45%),<br>CsA. Hypertrichosis all. Mild CsAN 3<br>MMF: diarrhea 2, bacterial pneumonia 1
Fujinaga S 2015 [87], Japan	Positive role of rituximab in switching from cyclosporine to mycophenolate mofetil for children with high-dose steroid-dependent nephrotic syndrome	Prospective study<br>Single center	SDNS<br>Rituximab<br>Cyclosporine<br>MMF	26	**Age at NS onset:** 7.0 ± 4.0 yrs<br>**Age at CsA:** 8.3 ± 4.1 yrs<br>**Age at MMF:** 12.1 ± 4.0 yrs<br>**FU:** after MMF start: 28.8 ± 9.9 mths<br>16M, 10F<br>**Indication:** complicated SDNS despite CsA (for 46.5±27.2 mths), CsAN in 11 pts (42%) with CsA > 24 mths	**MMF** started at initial dose 250 mg/12h, adjusted to MPA trough level of 2-5 µg/ml (max. 1g bd).<br>After MMF start; CsA dosage gradually tapered<br>Duration:<br>Other: tapering dose of steroids, steroids fro relapses.<br>**In case of MMF**	**Remission** despite CsA withdrawal: 11/26 (42%)<br>**MMF Failure and RTX:** 11/26 (42%)<br>**Sustained remission > 1 yr** without steroids: 22/26 (85%)<br>**Discontinuation of MMF:** 15/26 (58%)<br>**RR:** with CsA: 1.0±0.9/yr; with MMF and RTX 0.7±0.5/yr (p=0.07)<br>AE: MMF: mild gastrointesinal symptoms 2, herpes simplex 2

1st author, year, [Ref.], country of origin	Title of Publication	Study design	Key words	N	Population Characteristics	Treatment	Outcomes
						**treatment failure:**<br>**RTX** (n=11)**:** single dose of 375 mg/m^2 (max. 500 mg)<br>**Duration**:<br>**Other:** tapering dose of steroids, steroids for relapse	RTX: mild infusion reactions 5/11, late-onset neutropenia requiring G-CSF 1/11
**MMF - LEV - TAC**							
Basu 2017 [91]<br>India	Long-term efficacy and safety of common steroid sparing agents in idiopathic nephrotic syndrome	Retrospective cohort study	Nephrotic Syndrome<br>Mycophenolate mofetil<br>Levamisol<br>Tacrolimus	Total 340, MMF 130	**LEV:** n=129<br>**Age at NS onset:** 76.8 ± 32.0 mths<br>**Age at LEV:** not stated<br>**Duration of NS:** 3.3 ± 2.1 yrs<br>**FRNS/SDNS**: 78/51<br><br>**MMF:** n=130<br>**Age at NS onset:** 85.2 ± 28.8 mths<br>**Duration of NS:** 3.4 ± 2.6 yrs<br>**FRNS/SDNS**: 74/56<br><br>**TAC:** n=81<br>**Age at NS onset**: 79.2 ± 26.2 mths<br>**Duration of NS**: 3.1 ± 1.6 ysr<br>**FRNS/SDNS**: 47/34<br><br>**FU:** at least 30 mths<br>210M, 130F<br>**Indications:** SDNS (preferred MMF or TAC), FRNS (preferred LEV or MMF), no previous exposure to steroid-sparing agents	**LEV:** 2.5 mg/kg on alternate days<br>Or<br>MMF: 1200 mg/m^2 daily<br>Or<br>Tacrolimus 0.1-0.2 mg/kg/d<br><br>**Duration:** steroid-sparig agent continued for 1 yr following complete stop of steroids<br>**Other:** oral steroids in tapering dose	**Change in relapse rate at 12 mths from previous year:**<br>LEV: -3.1 ± 1.1<br>MMF: -4.5 ±1.3<br>TAC: -5.1 ± 1.3<br>All p<0.001 relative to pre-study period.<br>**RR at 12 mths and 24 mths** (after stop of agent)<br>LEV: 1.7/yr; 2.8/yr<br>MMF: 0.9/yr; 1.4/yr<br>TAC: 0.9/yr; 1.8/yr<br>**Relapse-free survival at 30 mths:**<br>**TAC vs. MMF:** 61.7 vs. 38.5%, p<0.001<br>**TAC vs. LEV:** 61.7% vs. 24%, p<0.0001<br>**Time to relapse:**<br>LEV: 21 days<br>MMF: 23 days<br>TAC: 26 days<br>**Cumulative predn. dose at 12 mths:**<br>**TAC vs MMF and LEV:**<br>**82.7** ± 26.4 mg/kg/yr vs. 136.8±65.4 mg/kg/yr and 108.8 ± 35.7 mg/kg/yr (p<0.001)<br>**Cum. pred. dose between**

1st author, year, [Ref.], country of origin	Title of Publication	Study design	Key words	N	Population Characteristics	Treatment	Outcomes
							**18-30 mths:**<br>**MMF vs. TAC and LEV:** 74.4 mg/kg/yr vs. 96.4 mg/kg/yr (p=0.004); 74.4 vs. 117.6 mg/kg/yr (p<0.001).<br>**Predictors for relapse:** SDNS vs. FRNS: HR 2.14 (95% CI 1.79-2.96, p<0.001)<br>**AE:**<br>LEV (n=3): malaria 1, transient mood changes 2<br>MMF (n=15): minor, temporary reduction of dose 3, acute resp. infec. 3, UTI 1, acute hepatitis 1, abdominal chronic pain 2, rec. vomiting 1, raised liver enzymes 1, muscle pain 1<br>TAC (n=33): temporary drug discontinuation 8, complete drug stop: 1<br>SAE: gram-pos. pneumonia with admission 2, gram-neg. peritonitis 1, pancreatitis 1<br>Minor: 5 acute resp. inf., gastroenteritis 4, UTI 2, herpes simplex 1, abscess 2, stomatitis 2, convulsion 1, alopecia 2, hirsutism 1, eczema 2, hyperglycemia 3, leukopenia 2
**CsA/Tac – CPA – LEV - MMF**							
Moorani 2019 [92]<br>Pakistan	Immunosuppressive therapy in children with primary nephrotic syndrome: single center	Retrospective chart review	Nephrotic syndrome<br>Minimal change disease<br>Oral prednisolone	130	**CPA:** n=70<br>**CsA:** n=49<br>**LEV:** n=55<br>**MMF:** n=21<br>**CsA+MMF:** n=12	**Sequential use:**<br>**CPA:** 2-3 mg/kg/d for 8-12 wks (2nd line)<br>**CNI:** (3rd line), dose	**Remission (complete; partial) at 6 mths:**<br>CPA: 45/70; 13/70<br>CsA: 30/49; 18/49<br>LEV: effective 44/55

1st author, year, [Ref.], country of origin	Title of Publication	Study design	Key words	N	Population Characteristics	Treatment	Outcomes
	experience, Karachi, Pakistan		Levamisole<br>Cyclophosphamide<br>Cyclosporin<br>Mycophenolate mofetil		**TAC+/-MMF** n=11<br><br>**Age at NS onset:** 4.78 ± 3.23 yrs<br>**Age at medication:** not stated<br>**FU:** not stated, at least 6 mths<br>**Indication:** SDNS (n=55), FRNS (n=75), steroid toxicity	not stated<br>**LEV:** 2-2.5 mg/kg on a.d. for 6-24 mths (1st line)<br>**MMF:** (3rd line): dose not stated<br><br>**Other:** oral steroids	MMF: 4/21; 16/21<br>CsA+MMF: 4/12; 7/12, CsA dependent 9/12<br>**Outcome last FU:**<br>Compl. Rem. Off treatment: 65/130<br>Compl. Rem. ON treatment: 34/130<br>Partial rem. ON treatment: 12/130<br>**AE:**<br>CPA: severe infection (disseminated chicken pox 9, BMS 5, alopecia 3)<br>CsA: gum hyperplasia 5, hypertrichosis 6, renal dysfunction 7, deafness 1<br>LEV: pancytopenia 1, allergic rash 1<br>MMF: none

Table S8.4: Levamisole (LEV)

1st author, year, [Ref.], country of origin	Title of Publication	Study design	Keywords	N	Population characteristics	Treatment	Outcomes
**LEVAMISOLE (LEV)**							
Fu 2000 [114] Taiwan<br><br>Only abstract available	Levamisole in steroid-sensitive nephrotic syndrome: children with steroid-dependency and/or frequent relapses	Prospective single center study	SDNS Levamisol Children relapse	27	**Age at NS onset:**<br>**Age at LEV:**<br>**FU:** 12.2 (6-24) mths<br>**Indications:** SDNS, FRNS	**LEV:** 2-3 mg/kg daily or a.d., depending on patients response<br>**Duration:**<br>**Other:** oral steroids in tapering dose	**Relapses:** 20/27<br>**RR:** decreased from 5.74 ± 3.24/yr to 1.91 ± 2.0/yr (p<0.05)<br>**Steroid dose:** decreased form 0.62±0.42 mg/kg/d to 0.21 ± 0.35 mg/kg/d (p<0.05)<br>**AE:** transient leukopenia 7
Alshaya 2002 [115] Saudi Arabia<br><br>Only abstract available	Levamisole treatment in steroid sensitive nephrotic syndrome	Retrospective single center study	SDNS Levamisol Children relapse	9	**Age at NS onset:**<br>**Age at LEV:** 6 (3.5-10) yrs<br>**FU:**<br>**Indications:**	**LEV:** 3 mg/kg/48 hrs<br>**Duration:** 6-24 mths<br>**Other:** oral steroids in tapering dose	**Remarkable reduction in no. of relapses and the steroid maintenance dose:** 4/9<br><br>**AE:** no significant observed
Donia A 2002 [116] Egypt	Levamisole: Adjunctive therapy in steroid-dependent minimal change nephrotic children	Prospective single center study	Levamisole Children Steroid dependent Minimal change nephrotic syndrome	20	**Age at NS onset:** 7.4 ± 2.89 yrs<br>**Age at LEV:** not stated<br>**FU**: 12 mths<br>16 M, 4 F<br>**Indications:** SDNS with steroid toxicity, no previous steroid-sparing agent	**LEV:** 2.5 mg/kg a.d.<br>**Duration:** 6 mths<br>**Other:** oral steroids in tapering dose	**Remission at 6 mths (end of LEV)/ 12 mths (6-mths off-LEV):** 11/20 (55%)/ 5/20 (25%)<br>Relapses during 12 mths study period: 15/20 (75%)<br>**Time to relapse:** 6.83 (0.23-11.67) mths<br>**AE:** no significant observed
Al-Ibrahim 2003 [117] Saudi-Arabia	Levamisole therapy as a second-line immunosuppressive agent in corticosteroid-sensitive nephrotic syndrome in children	Retrospective chart review	Nephrotic syndrome Steroid-sensitive, levamisole, cyclophosphamide	24	**Age at NS onset:** 32 mths (range 18 mths to 9.5 yrs)<br>**Age at LEV:** not stated<br>**FU:** range 18 mths to 5 yrs<br>18M, 6 F<br>**Indications:** SDNS (13, 54%), FRNS (11, 46%)	**LEV:** 2.5 mg/kg a.d.<br>**Duration:** min. 8 mths, mean 8.5 mths<br><br>**Other:** prednisolone a.d. in tapering dose for min. 8 mths.<br>Non-responder to LEV after 6 mths (7) and relapsers after LEV has been stopped (6): CPA	**LEV:**<br>**Remission:** 17/24 (71%)<br>**Sustained remission (>6mths, mean 19.4 mths):** 11/17 after LEV stopped<br>**RR:** decreased from 4 (3.5-5)/year to 1.3 (0-2)/yr in 17 responders<br>**AE (LEV):** mild transient rash 2, mild GI symptomatic 2, transient leucopenia 1

1st author, year, [Ref.], country of origin	Title of Publication	Study design	Keywords	N	Population characteristics	Treatment	Outcomes
						2.5 mg/kg/day for 12 weeks	**CPA after LEV:**<br>Sustained remission for mean of 10 mths: 8/13<br>**AE (CPA after LEV):** transient leucopenia 3
Sümegi 2004 [118] Hungary	Long-term effects of levamisole treatment in childhood nephrotic syndrome	Retrospective single center study	Nephrotic Syndrome<br>Levamisole<br>Prednisolone<br>Relapse rate<br>Cumulative steroid dose	34	**Age at NS onset:** not stated<br>**Age at LEV:** not stated<br>**FU:** 60 mth<br>21 M, 13 F<br>**Indications:** FRNS (n=15), SSNS (n=13), 6 developed secondary SRNS before LEV; 19/34 with previous steroid-sparing agents: CPA 9, CHL 10), steroid toxicity	**LEV:** 2 mg/kg/day<br>**Duration:** 17 ± 7 mths (range 5-36 mths)<br>**Other:** oral steroids in tapering dose	**Relapses:** 11/34 during LEV, another 6 post-LEV<br>**RR:** fell from 4.41/yr to 0.41/yr by the end of LEV (p<0.001); 0.22/yr in FU-period of 24 mths post-LEV<br>**Cumulative steroid dose:** decreased from 7564.1 ± 3467.1 mg/yr to 1472±729.9 mg/yr (p<0.0001);<br>Steroids were stopped in 23/34 pts.<br>**Time to relapse:**<br>**AE:** reversible neutropenia 5/34, requiring intermittend LEV stop, re-start was possible
Fu 2004 [119] Taiwan	Levamisole in steroid-sensitive nephrotic syndrome with frequent relapses and/or steroid dependency	Prospective single center study	Levamisole<br>Steroid-sensitive nephrotic syndrome<br>Steroid dependency	36	LEV every other day: n=20<br>LEV daily after relapse after 3 mths: n=16<br>**Age at NS onset:**<br>Group 1: 4.58 ± 3.15 yrs<br>Group 2: 5.85 ± 3.32 yrs<br>**Age at LEV**:<br>Group 1: 8.02 ± 5,31 yrs<br>Group 2: 8.82 ± 5.12 yrs<br>**FU:** 20.4 ± 9.2 mths<br>25 M, 11F<br>**Indication:** SDNS (22), FRNS (14), of those 11 with previous CPA treatment	**LEV:**<br>Group 1: 2-3 mg/kg every other day for 4-24 mths<br>Group 2: 3 mths every other day, then mths changed to daily for 6 mths (n=11) or 4-18 mths (n=5)<br><br>**Other:** oral steroids in tapering dose	**Relapses after therapy:**<br>Group 1: 4.82 ± 3.15 vs. 2.01 ± 2.5 (p<0.05)<br>Group 2: 5.97 ± 3.38 vs. 1.34 ± 2.1 (p<0.05)<br>**Pred. dose – reduction:**<br>Group 1: 0.57 ± 0.37 vs. 0.15 ± 0.33 mg/kd/day) (p<0.05)<br>Group 2: 0.61 ± 0.42 vs. 0.19 ± 0.35 mg/kg/day (p<0.05)<br>**Discountinuation of LEV:**<br>Group 1: 6, Group: 4 after 15-24 mths<br>**AE**: Transient leukopenia: 9/36
Hafeez F 2006 [120] Pakistan<br><br>Only	Levamisole in steroid dependent and frequently relapsing nephrotic	Retrospective single center		70	**Age at NS onset:** 5.50 ± 2.97 yrs<br>**Age at LEV:** not stated<br>**FU:** at least 1 yr<br>**M/F ratio:** 4:1	**LEV:** 2.5 mg/kg a.d.<br>**Duration:** 1 yr<br>**Other:** oral steroids in tapering dose	**Remission:** No relapses on LEV 19/70 (27.1%); ineffective 11/70 (15.7%)<br>**Relapses:** 40/70 (57.1%) on LEV<br>**Steroid dose:** significantly

1st author, year, [Ref.], country of origin	Title of Publication	Study design	Keywords	N	Population characteristics	Treatment	Outcomes
abstract available	syndrome				**Indications:** FRNS, SDNS		reduced<br>**AE:** transient rash, occasional vomiting
Boyer O 2008 [121] France	Short- and long-term efficacy of levamisole as adjunctive therapy in childoohd nephrotic syndrome	Prospective single center study	Children<br>Pediatric<br>Minimal-change disease<br>Steroids<br>Side effects<br>Growth<br>Height	10	**Age at NS onset:** 4.3 (1.4-12.8) yrs<br>**Median duration of NS**: 6.4 (2.1-10.3) yrs<br>**Age at LEV:** not stated<br>**FU:** not stated, at least 2 yrs after LEV start<br>6M, 4F<br>**Indications:** SDNS	**LEV:** 2.5 mg/kg 3 alternate days a week (Mo-Wed-Fri)<br>**Duration:** 12 mths<br>**Other:** oral steroids in tapering dose if required	**Relapse-free:** 6/10 during LEV, 5/10 off-LEV<br>**Relapse frequency:**<br>Fell from 6.0 (4.0-9.0)/pt./yr pre-LEV to 0 (0.0-4.0)/pt./yr. on LEV (p=0.002) and was 0.5 (0.0-8.0)/pt/yr. 1 year post-LEV (p=0.002)<br>**Cumulative pred. dose/year:**<br>Fell from 6,067 (1.660-5,271) mg/m^2/yr to 2.920 (782-5,271) on LEV and was 716 (0-3,367) off-LEV.<br>**Median height velocity:**<br>improved from 3.0 (0.3-6.0)cm/yr to 3.7 (0.0-8.0) (p=0.058) on LEV and 5.4 (0.0-9.1) post-LEV) (p=0.19)<br>**AE:** no serious reported
Madani A 2010 [122] Iran	Effect of levamisole in steroid-dependent nephrotic syndrome	Retrospective study<br>Single center	Levamisole<br>Steroids<br>Nephrotic syndrome<br>Immunosuppressive agents	304	**Age at NS onset:** 4.8 ± 3.1 yrs<br>**Age at LEV:**<br>**FU:** at least 6 mths; 6.7 ± 3.9 yrs<br>208 M, 96 F<br>**Indications:** SDNS, FRNS, previous treatment in 62 (20.4%) pts. with CPA (18.1%) and/or CsA (7.2%)	**LEV:** 2.5 mg/kg a.d.<br>**Duration:** 17.87 ± 11.22 mths<br>**Other:** oral steroids in tapering dose	**Remission:**<br>84/304 (27.6%)<br>**RR:** decreased from 2.02 ± 1.2/ yr to 0.92 ± 0.98/yr on LEV and 1.07 ± 1.2/yr 1 yr post-LEV<br>**Steroid dose:** reduction by 53.24 ± 45.97%<br>**Rate of LEV resistance:**<br>LEV as 1st agent: 31.4%<br>LEV as 2nd/3rd agent: 42.5% (CPA), 57.1% (CsA), 46.7% (both)<br>**AE:** in 2, both reversible, neutropenia 1, vertigo 1
Elmas AT 2013 [123] Turkey	Short- and lon-term efficacy of levamisole in children with	Retrospective study	Child<br>Levamisole<br>Frequently relapsing	29	**Age at NS onset:** 4.0 (2.0-12.0) yrs<br>**Age at LEV:** 9.0 (4.0-16.0) yrs<br>**Duration of NS**: 5.0 (1.5-14.0)	**LEV:** 2.5 mg/kg 3 alternative days<br>**Duration:** 12 mths<br>**Other:** oral steroids in	**Relapse free:** 23/29 during LEV<br>**Sustained remission 1r end-LEV:** 18/29<br>**RR:** fell from 4.0 (3.0-8.0)/yr pre-

1st author, year, [Ref.], country of origin	Title of Publication	Study design	Keywords	N	Population characteristics	Treatment	Outcomes
	steroid-sensitive nephrotic syndrome		nephrotic syndrome<br>SDNS<br>Remission rate		yrs<br>**FU:** at least 12 mths<br>19 M, 10 F<br>**Indications:** SDNS (14, 48.3%), FRNS (15, 51.7%)	tapering dose	LEV to 0.0 (0.0-2.0)/yr during-LEV and 0.0 (0.0-1.0)/yr end-LEV (p=0.0001)<br>**Cumulative steroid dose:** reduced from 5582.0 (2137.0-17340.0) $mg/m^2/yr$ pre-LEV to 2166.0 (840-0-9325.0) $mg/m^2/yr$ during-LEV to 0.0 (0.0-5386.0) $mg/m^2/yr$ end-LEV.<br>**AE:** no significant observed
Ekambaram S [124]<br>2014<br>India	Efficacy of levamisole in children with frequently relapsing and steroid-dependent syndrome	Retrospective chart review<br>Single center	Treatment<br>Steroids<br>Outcome<br>Relapse<br>Nephrotic syndrome	97	**Age at NS onset:**<br>SDNS: 2.5 ± 1.1 yrs<br>FRNS: 3.1 ± 1.8 yrs<br>**Age at LEV:**<br>SDNS: 3.9 ± 1.7 yrs<br>FRNS: 4.8 ± 2.3 yrs<br>**FU:** at least 6 mths<br>53 M, 44 F<br>**Indications:** SDNS (35, 36%)), FRNS (62, 64%)	**LEV:** 2 mg/kg daily<br>**Duration:** at least 6 mths (18.7 ± 6.4 mths); 1 yr (n=65)<br>**Other:** oral steroids in tapering dose, stopped after 11.84 ± 1.3 mths.	**Relapse-free** at 1yr post-therapy (n=34): 25/34 (73.5%) during treatment, 22/34 (64.7%) 1 yr post-LEV.<br>**RR:** fell from 2.41 ± 0.5/year to 1.3 ± 0.7/ year on LEV and to 0.48 ± 0.8/yr post-LEV<br>**Cumulative steroid dose:** reduced from 4109.29 ± 1154 $mg/m^2/yr$ to 2491.8 ± 694 on LEV and to 660.7 ± 10.7 $mg/m^2/yr$ post-LEV<br>**AE:** not stated
Kuzma-Mroczkowska E, 2016<br>[125]<br>Poland	Levamisole therapy in children with frequently relapsing and steroid-dependent nephrotic syndrome	Retrospective single center chart review	Children<br>Immunomodulation<br>Nephrotioc syndrome<br>Levamisole	53	**Age at NS onset:** 3.1 ± 2.0 yrs<br>**Age at LEV:** 6.5 ± 3.0 yrs<br>**Duration of NS:** 3.4 ± 2.9 yrs<br>**FU:**<br>31 M, 22 F<br>**Indications:** FRNS, SDNS, steroid toxicity, previous treatments with MP pulses (13, 24.5%), CPA (10, 18.9%), CHL 8 (15.1%), CsA 1 (1.9%), Azathioprine (1, 1.9%)	**LEV:** 2.5 mg/kg a.d. for 1 mth, then twice weekly<br>**Duration:** at least 6 mths, 15.0 ± 7.3 mths<br>**Other:** oral steroids in tapering dose (duration: 8.7 ± 8.5) mths	**Relapses during LEV:** 34/53 (64.2%), of those 23 during 1st 6 mths of LEV<br>**Time to 1st relapse:** 8.8 ± 8.1 mths<br>**RR:** fell from 2.7 ± 2.0 /yr to 1.8 ± 2.1/yr (p=0.02).<br>**AE: in** 16, all transient; allergic rash 9, abdominal pain 3, hypertransaminasemia 3, arthralgia 2, leukopenia 1, thrombocytopenia 1
Abeyagunawardena AS, 2017<br>[126]	Efficacy of higher-dose levamisole in maintaining remission in	Single center pilot study	Low-dose levamisol<br>Steroid-dependent	58	**Age at NS onset:** 7.95 yrs<br>**Age at LEV:** not stated<br>**FU:** at least 12 mths<br>33 M, 25 F	**LEV:** 2.5 mg/kg daily<br>**Duration:** 1yr<br>**Other:** low-dose alternate day predn. for 1	**Relapse-free within 12 mths:** 12/58 (20.7%)<br>**Relapses at 12 mths:**<br>A.d. LEV: 2.8 ± 0.8/yr

1st author, year, [Ref.], country of origin	Title of Publication	Study design	Keywords	N	Population characteristics	Treatment	Outcomes
Sri Lanka	steroid-dependent nephrotic syndrome		nephrotic syndrome relapse		**Indications:** SDNS, patients on LEV a.d.+low-dose pred. and > 2 relapses during the 12 mths before enrolment; no use of other previous steroid-sparing agents	yr (0.1-0.6 mg/kg)	Daily LEV: 1.3 ± 0.9/yr<br>P<0.001<br>**Pred. dose:** 254.16 (210.68-281.81) (within 1 yr LEV a.d.) vs. 154.05 (116.75-197.0) mg/kg/yr (1 yr daily LEV)<br>**AE:** no serious infections, minor infections not counted.
Kiruba 2017 [127]<br>India	Levamisole in Frequently-relapsing and steroid-dependet nephrotic syndrome	Retroespective chart review	Corticosteroids<br>Immunomodulators<br>Proteinuria<br>Outcome<br>Treatment	95	**Age at NS onset:**<br>FRNS: 2.5 (1.9-4.0) yrs<br>SDNS: 2 (1.8-4.0) yrs<br>**Age at LEV:**<br>FRNS: 5 (3-8) yrs<br>SDNS: 6 (3-8) yrs<br>**FU:** at least 1yr<br>55M, 40F<br>**Indications:** FRNS (62), SDNS (33)	**LEV:** 2—2.5 mg/kg a.d., if failure (n=25) daily<br>**Duration:** 24 mths<br>**Other:** oral steroids in tapering dose	**Remission on LEV a.d.:**<br>FRNS: 82%, SDNS: 58%<br>**Remission on LEV a.d. and daily:** FRNS: 93.5%, SDNS 79%<br>**No sustained rem. After LEV stop**: 41/84 (48.8%), requiring other agents, e.g. CPA (22), MMF (19)<br>**RR:** decreased from 4.22 ± 0.46/yr to 1.35 ± 0.36/yr (p<0.01): increased to 2.57/yr after stop of LEV<br>**Cumulative steroid dose:** reduced from 4200 (3200-4300) mg/m^2 to 1100 (500-2900) mg/m^2 (p<0.001).<br>**AE:** no significant reported
Moorani K, 2020 [128]<br>Pakistan	Efficacy of Levamisole in children with Frequent relapsing and steroid dependent nephrotic syndrome at tertiary care center - Karachi	Retrospective single center study	Remission of proteinuria<br>Frequent relapsing<br>Steroid dependent nephrotic syndrome<br>Relapses<br>Levamisol	81	**Age at NS onset:** 3.72 ± 2.33 yrs<br>**Age at LEV:** 8.44±3.70 yrs<br>**FU:** 11.70 ± 11.23 mths post-LEV<br>48 M, 33 F<br>**Indications:** FRNS (n=66), SDNS (n=15)	**LEV:** 2-2.5 mg/kg daily (48; 59.3%) or a.d. (33; 40.7%)<br>**Duration:** 15.69 ± 9.39 mths (6-36 mths)<br>**Other:** oral steroids in tapering dose	**RR:** fell from 3.30±0.50/yr to 0.98±1.1/yr during-LEV and to 0.79±1.27/yr post-LEV (p=0.001)<br>**Cumulative steroid dose:**<br>Pre-LEV: 3389±2785.22 mg/m^2/yr<br>During LEV: 2471.71±2024.98<br>Post-LEV: 661.37±905.37<br>(p=0.001)<br>**AE:** pancytopenia and allergic rash 1
**Studies with LEV – comparing different steroid-sparing agents**							
**LEV vs. CPA**							
Alsaran K 2001 [100]	Levamisole vs. cyclophosphamid	Retrospective analysis	SDNS<br>Levamisol	Total	**LEV** n=24<br>**CPA** n=27	**LEV:**<br>**CPA:**	**RR:** Reduced by 0.28 relapses/patient/year **(LEV)** and

1st author, year, [Ref.], country of origin	Title of Publication	Study design	Keywords	N	Population characteristics	Treatment	Outcomes
Canada<br><br>Only abstract available	e for frequently relapsing steroid-dependent syndrome		cyclophosphamide<br>Children<br>relapse	51, LEV: 24	**Age at NS onset:**<br>**Age at LEV:**<br>**FU:**<br>**Indications:**	**Duration:**<br>**Other:**	0.32 relapses/pt-/yr **(CPA)**<br>**Cumulative dose of pred.:** reduced by 336mg/m^2/mth **(LEV)** and 387 mg/m^2/month **(CPA).**<br>**AE:**
**LEV - CPA/ CHL - CsA**							
Chen SY 2010 [90]<br>Taiwan	Treatment course of steroid-dependent nephrotic syndrome: emphasized on treatment effect	Retrospective single center study	Chlorambucil<br>Cyclophosphamide<br>Cyclosporine<br>Levamisol	Total 46, LEV:15	**LEV:** n=15<br>**CHL:** n=22<br>**CsA:** n=8<br>**2nd CPA:** n=6<br><br>**Age at NS onset: 4**.5 (1-15.5) yrs<br>**Age at medication:** not stated<br>**FU:** 96 (22-244) mths<br>33M, 13 F<br>**Indications:** SDNS despite one course of CPA as 1st line agent	**LEV:** 2-3.3 mg/kg/day for 3-20 mths<br>**CHL:** 0.1-0.2 mg/kg/d for 8 weeks<br>**CsA:** 3.5-5 mg/kg/d for 6-14 mths, then tapering for 12-23 mths<br>**2nd CPA:** 2-3 mg/kg/day for 8 wks<br><br>**Other:** oral steroids in tapering dose	**Relapses after 1st CPA course:** 25/46<br>**Relapses after additional treatment**:<br>LEV: 1/15 remission, 1 with SSNS relapses, 1 relapse-free period, 1 LEV dependency, 10 no response<br>CHL: 7/22 complete remission, 5 with relapses, 4 disease-free for 6.8 (2-11) mths, 3 no response<br>CsA: 1/8 rem., 1 eith SSNS relapses, 1 disease-free period, 4 CsA dependency<br>2nd CPA: 3/6 disease-free period, 1 decreased steroid threshold, 2 no response<br>**AE:** not stated
Abeyagunawardena 2003 [89]<br>UK	The use of steroid-sparing agents in steroid-sensitive nephrotic syndrome	Retrospective cohort study	Nephrotic syndrome<br>Steroid sensitive<br>Corticosteroid<br>Relapse<br>Remission<br>Steroid-sparing		**CPA**: n=178 (1st agent)<br>**LEV**: n=113, of those 1st agent n=65<br>**CsA:** n=61 as 2nd agent in SDNS following CPA treatment; 1st agent n=8<br>**Chlorambucil**: n=15 when other therapies failed<br><br>**Age at NS onset:** not stated<br>**FU:** 6.1 yrs (IQR 1-17.4) yrs<br>**Indications:** SDNS, FRNS	**CPA:** 3 mg/kg daily<br>**Duration:** 8 wks<br><br>**LEV:** 2.5 mg/kg on alternate days<br>**Duration**: 3.2 (1-7) yrs<br><br>**CsA:** 3-5 mg/kg in 2 divided doses (12htrough level 50-150 µg/l)<br><br>**Other:** all: oral steroids in tapering dose	**CPA:** sustained remission at 1 yr: 94/178 (54%), at 2 yrs: 44%, at 5 yrs: 32%, 2nd course of CPA: 18/178 (10%)<br>**LEV:** sustained remission at 1 yr 19/65 (30%) as 1st agent, relapse after stop of LEV 4. 32/48 (66%) wigh sustained remission when used after unsuccessful CPA<br>**CsA:** 43/61 (70%) with sustained remission; relapse following discontinuation 32 (51%).<br>**CHL:** sustained remission at 1 yr: 7.<br>**AE:**<br>**CPA:** neutropenia 16/178,

1st author, year, [Ref.], country of origin	Title of Publication	Study design	Keywords	N	Population characteristics	Treatment	Outcomes
							requiring interruption of med., serious infection 4/178<br>**LEV:** reversible neutropenia 4, skin rash 2 (requiring end of treatment)<br>**CsA:** nephrotoxicity 4<br>**CHL:** not stated
**LEV – CPA – CsA - MMF**							
Alsaran K, 2017 [129] Saudi Arabia	Experience with second line drugs in frequently relapsing and steroid dependent childhood nephrotic syndrome in a large Saudi center	Retrospective single center study	Levamisole<br>Cyclocphosphamide<br>MMF<br>Cyclosporine<br>Steroid dependent<br>Frequently relapsing<br>Prednisolone	Total 60, LEV: 20	**LEV:** n=20<br>**MMF:** n=25<br>**CPA:** n=12<br>**CsA:** n=13<br><br>**Age at NS onset:** 3.75 ± 1.1 yrs<br>**Age at drug start:** 4.8 ± 1.0 yrs<br>**FU:**<br>M/F ratio: 1.9:1<br>**Indications:** SDNS, FRNS	**LEV:** 2.5 mg/kg/d for 1 yr<br>**MMF:** 1200 mg/m^2/d in 2 divided doses for 1 yr<br>**Oral CPA:** 2 mg/kg/d for 12 weks<br>**CsA:** 5 mg/kg/d in 2 divided doses for 1 yr (trough levels 80-100 mg/ml)<br><br>**Other:** oral steroids in tapering dose	**RR (1yr pre; 1yr during; 1yr post-drug):**<br>LEV: 3.6± 0.5/yr; 1.6± 0.6/yr; 1.0± 0.9/yr (p<0.0001)<br>MMF: 3.9± 0.6/yr; 1.5/± 0.7yr; 0.9± 0.8/yr (p<0.0001)<br>CPA: 4.0± 0.6/yr; 1.8± 0.7/yr, 1.3± 0.8/yr (p<0.0001)<br>CsA: 4.1± 0.4/yr; 1.5± 0.5/yr; 0.8± 0.7/yr (p<0.0001)<br>**Cumulative dose of steroids**<br>LEV: 4100±1500; 1700±650; ± 900±15 mg/m^2/yr (p<0.0001)<br>MMF: 4300±1010; 1610±595;850±20 mg/m^{2}yr (p<0.0001)<br>CPA: 3900±950; 1800±600; 920±18 mg/m^2/yr (p<0.0001)<br>CsA: 4350±1100; 1510±600;705±25 mg/m^2/yr (p<0.0001)<br>**AE:**<br>LEV: transient neutropenia 4<br>MMF: diarrhea 3<br>CPA: transient neutropenia 3, hemorrhagic cystitis 1<br>CsA: mild hirsutism 13, gum hypertrophy 13, mild hypertension 4
**LEV – MMF - TAC**							
Basu B,	Long-term efficacy	Retrospectiv	Nephrotic	Tot	**LEV:** n=129	**LEV:** 2.5 mg/kg on	**Change in relapse rate at 12**

1st author, year, [Ref.], country of origin	Title of Publication	Study design	Keywords	N	Population characteristics	Treatment	Outcomes
2017 [91]<br>India	and safety of common steroid sparing agents in idiopathic nephrotic syndrome	e cohort study<br>Single center	Syndrome<br>Mycophenolat e mofetil<br>Levamisol<br>Tacrolimus	al34 0,<br>LEV 129	**Age at NS onset:** 76.8 ± 32.0 mths<br>**Age at LEV:** not stated<br>**Duration of NS:** 3.3 ± 2.1 yrs<br>**FRNS/SDNS**: 78/51<br><br>**MMF:** n=130<br>**Age at NS onset:** 85.2 ± 28.8 mths<br>**Duration of NS:** 3.4 ± 2.6 yrs<br>**FRNS/SDNS**: 74/56<br><br>**TAC:** n=81<br>**Age at NS onset**: 79.2 ± 26.2 mths<br>**Duration of NS**: 3.1 ± 1.6 ysr<br>**FRNS/SDNS**: 47/34<br><br>**FU:** at least 30 mths<br>210M, 130F<br>**Indications:** SDNS (preferred MMF or TAC), FRNS (preferred LEV or MMF), no previous exposure to steroid-sparing agents	alternate days<br>Or<br>MMF: 1200 mg/m^2 daily<br>Or<br>Tacrolimus 0.1-0.2 mg/kg/d<br><br>**Duration:** steroid-sparig agent continued for 1 yr following complete stop of steroids<br>**Other:** oral steroids in tapering dose	**mths from previous year:**<br>LEV: -3.1 ± 1.1<br>MMF: -4.5 ±1.3<br>TAC: -5.1 ± 1.3<br>All p<0.001 relative to pre-study period.<br>**RR at 12 mths and 24 mths** (after stop of agent)<br>LEV: 1.7/yr; 2.8/yr<br>MMF: 0.9/yr; 1.4/yr<br>TAC: 0.9/yr; 1.8/yr<br>**Relapse-free survival at 30 mths:**<br>**TAC vs. MMF:** 61.7 vs. 38.5%, p<0.001<br>**TAC vs. LEV:** 61.7% vs. 24%, p<0.0001<br>**Time to relapse:**<br>LEV: 21 days<br>MMF: 23 days<br>TAC: 26 days<br>**Cumulative predn. dose at 12 mths:**<br>**TAC vs MMF and LEV: 82.7** ± 26.4 mg/kg/yr vs. 136.8±65.4 mg/kg/yr and 108.8 ± 35.7 mg/kg/yr (p<0.001)<br>**Cum. pred. dose between 18-30 mths:**<br>**MMF vs. TAC and LEV:** 74.4 mg/kg/yr vs. 96.4 mg/kg/yr (p=0.004); 74.4 vs. 117.6 mg/kg/yr (p<0.001).<br>**Predictors for relapse:**<br>SDNS vs. FRNS: HR 2.14 (95% CI 1.79-2.96, p<0.001)<br>**AE:**<br>LEV (n=3): malaria 1, transient mood changes 2

1st author, year, [Ref.], country of origin	Title of Publication	Study design	Keywords	N	Population characteristics	Treatment	Outcomes
							MMF (n=15): minor, temporary reduction of dose 3, acute resp. infec. 3, UTI 1, acute hepatitis 1, abdominal chronic pain 2, rec. vomiting 1, raised liver enzymes 1, muscle pain 1<br>TAC (n=33): temporary drug discontinuation 8, complete drug stop: 1<br>SAE: gram-pos. pneumonia with admission 2, gram-neg. peritonitis 1, pancreatitis 1<br>Minor: 5 acute resp. inf., gastroenteritis 4, UTI 2, herpes simplex 1, abscess 2, stomatitis 2, convulsion 1, alopecia 2, hirsutism 1, eczema 2, hyperglycemia 3, leukopenia 2
**CPA – CsA – MMF – LEV - AZA**							
Moustafa BH 2016 [101] Egypt	Immunosuppressive therapy in children with steroid-resistant, frequently-relapsing, and steroid-dependent idiopathic nephrotic syndrome: a single center experience	Retrospective chart review<br>Single center	Childhood Nephrotic Syndrome<br>Steroid Resistance<br>Steroid Dependence<br>Relapse<br>immunosuppressants	79	**CPA:** n=28<br>**CsA:** n=6<br>**MMF:** n=2<br>**LEV:** n=40<br>**AZA:** n=10<br><br>**Age at NS onset:** 3.7 (1.3-10.5) yrs<br>**Age at medication:** not stated<br>**FU:**<br>44 M, 35 F<br>**Indications:** SDNS/ FRNS, steroid toxicity	**CPA:** 2-3 mg/kg/d orally for 8-12 wks or IV as monthly bolus of 500-750 mg/m^2 for 6 mths<br>**CsA:** 4-6 mg/kg/d divided into 2 doses for at least 12 mths<br>**MMF:** 1200 mg/m^2/day divided into doses<br>**LEV:** 2-2.5 mg/kg/dose twice weekly for 6-24 mths<br>AZA: 2 mg/kg/d for 8 wks.<br><br>Either given as 1st or 2nd line drug; some given in double- or triple-combination therapy	**Remission for 6 mths:**<br>**CPA:** 24/28 (85.7%)<br>**CsA:** 5/6 (83.3%)<br>**MMF:** ½ (50%)<br>**LEV:** 22/40 (55%)<br>**AZA:** 8/10 (80%)<br><br>**AE** (not differentiated between SDNS (n=79) and SRNS (n=51)):<br>CPA: Leukopenia 15/63 (23.8%), hemorrhagic cystitis 2/63 (3.2%)<br>CsA: gym hyperplasia 8/31 (25.8%), hirsutism 7/31 (22.6%), nephrotoxicity 2/31 (6.4%), hypertension 2/31 (6.4%)<br>MMF: diarrhea 7/12 (58.4%), nausea 3/12 (25%), abdominal pain 1 (8.%), cough 1/12 (8.3%)<br>LEV: none<br>AZA: Leukopenia 2/10, diarrhea

1st author, year, [Ref.], country of origin	Title of Publication	Study design	Keywords	N	Population characteristics	Treatment	Outcomes
						**Other:** oral steroids	2/10, abdominal pain 2/10, arthralgia 1/10
**CsA/Tac – CPA – LEV - MMF**							
Moorani 2019 [92] Pakistan	Immunosuppressive therapy in children with primary nephrotic syndrome: single center experience, Karachi, Pakistan	Retrospective chart review	Nephrotic syndrome<br>Minimal change disease<br>Oral prednisolone<br>Levamisole<br>Cyclophosphamide<br>Cyclosporin<br>Mycophenolate mofetil	130	**CPA:** n=90<br>**CsA:** n=88<br>**LEV:** n=55<br>**MMF:** n=39<br>**CsA+MMF:** n=20<br>**TAC+/-MMF** n=11<br><br>**Age at NS onset:** 4.78 ± 3.23 yrs<br>**Age at medication:** not stated<br>**FU:** not stated, at least 6 mths<br>**Indication:** SDNS (n=55), FRNS (n=75), steroid toxicity	**Sequential use:**<br>**CPA:** 2-3 mg/kg/d for 8-12 wks (2nd line)<br>**CNI:** (3rd line), dose not stated<br>**LEV:** 2-2.5 mg/kg on a.d. for 6-24 mths (1st line)<br>**MMF:** (3rd line): dose not stated<br><br>**Other:** oral steroids	**Remission (complete; partial) at 6 mths:**<br>CPA: 45/90; 13/90<br>CsA: 30/88; 18/88<br>LEV: effective 44/55<br>MMF: 4/39; 16/39<br>CsA+MMF: 4/20; 7/20, CsA dependent 9/20<br>**Outcome last FU:**<br>Compl. Rem. Off treatment: 65/130<br>Compl. Rem. ON treatment: 34/130<br>Partial rem. ON treatment: 12/130<br>**AE:**<br>CPA: severe infection (disseminated chicken pox 9, BMS 5, alopecia 3)<br>CsA: gum hyperplasia 5, hypertrichosis 6, renal dysfunction 7, deafness 1<br>LEV: pancytopenia 1, allergic rash 1<br>MMF: none

Table S8.5: Rituximab (RTX)

1st author, year, [Ref.], country of origin	Title of Publication	Study design	Keywords	N	Population characteristics	Treatment	Outcomes
**RITUXIMAB (RTX)**							
Kamei 2009 [130] Japan	Single dose of rituximab for refractory steroid-dependent nephrotic syndrome in children	Multicenter prospective trial	Refractory steroid-dependent nephrotic syndrome Children Clinical trial Rituximab Pharmacokinetics	12	**Age at NS onset:** 5.8 ± 4.3 yrs<br>**Age at RTX:** 12.7 ± 3.9 yrs;<br>8M, 4F<br>**FU:** not stated, at least until 6 months after RTX<br>**Indications:** refractory SDNS, steroid toxicity	**RTX:** single dose of 375 mg/m^2<br>**Duration:**<br>**Other:** tapering dose of steroids, CNI 9, Mizoribine 5, MMF 3	**Remission:** 3/12 at 12 months<br>**RR:** 2.83 ± 1.19 fell to 1.08 ± 1.08 at 6 months after RTX **Time to Relapses:** 9/12 at 129 (8-353) days after RTX;<br>**Pred. dose:** stopping pred. at 74 (IQR, 55-172) days after RTX: 12/12; Steroid-free period reduced from 7.0 ± 13.5 to 68.0 ± 30.7 days at 6 months after RTX<br>**Withdrawal of other IS:** 8/12 pts.<br>**B-cell depletion:** complete 10/12 pts. After single dose<br>**B-Cell recovery:** after 146.5 (IQR, 84-245) days<br>**AE:** mild infusion reactions 5/12 (42%)
Gulati 2010 [131] India, USA	Efficacy and Safety of Treatment with RTX for difficult Steroid-resistant and –dependent nephrotic syndrome: A multicentric report	Retrospective cohort study/ chart review 3 centers	SRNS SDNS Rituximab children	24 SDNS	**Age at NS onset:** 2.8 ± 0.9 yrs<br>**Age at RTX:** 11.7 ± 2.9 yrs<br>**FU:** 16.8 ± 5.9 months; 19M, 5F<br>**Indication:** SDNS; non-responsive to CPH, CNI, levamisole, MMF; SDNS with CNI toxicity	**RTX**: 375 mg/m^2 once every week, 2 doses<br>**Duration:** 2 weeks<br>**Other:** tapering dose of steroids, enalapril (SDNS with hypertension), furosemide	**Remission:** 20 (83.3%) at 12 months<br>**RR:** 4.0 ± 0.4 fell to 0.2 ± 0.3 episodes/pt. per year at 12 months after RTX<br>**Time to relapse:** 11.2 ± 2.7 months<br>**Pred. dose:** tapered to 0.3 to 0.5 mg/kg every other day: 8 pts.<br>**Withdrawal of other IS:** 1 or more in 12 pts.<br>**B-cell depletion:** CD19 counts: 12.6 ± 3.4% at baseline, 0.2 ± 0.1 % after 2 doses<br>**AE:** Mild infusion reactions: 3/24
Prytula 2010 [132] International (IPNA)	Rituximab in refractory nephrotic syndrome	Retrospective multicenter chart reviews	Pediatric NS Remission Renal	28	**Age at NS onset:** 4 yrs (range 18 mths to 17 yrs)<br>**Age at RTX:** not stated<br>**FU:** n.a.; 25M, 3F	**RTX:** 4 doses of 375 mg/m^2 weekly or 2 doses of 750 mg/m^2 every 14 days<br>**Duration:** 4 weeks	**Remission**: ongoing 10/28 for 4.5 (IQR, 1-10) mths at end of study<br>**Time to relapse**: 13/28 after 6 (IQR, 1-16) months

1st author, year, [Ref.], country of origin	Title of Publication	Study design	Keywords	N	Population characteristics	Treatment	Outcomes
		25 centers (IPNA, questionnaires)	transplantation		**Indications:** refractory SDNS, FRNS	**Other:** tapering dose of steroids: all, MMF 2, CNI 4, , CNI+MMF 1, ACE-I 2, CPH 2, plasma exchange 1	**B-cell depletion:** 19/21 with < 1% of lymphocyte count<br>**AE:** Acute infusion reactions 8, infection 1, hypogammaglobulinemia 1
Sellier-Leclerc 2010 [133] France	Rituximab efficiency in children with steroid-dependent nephrotic syndrome	Retrospective single center case series	Rituximab<br>Idiopathic nephrotic syndrome<br>CD19<br>B cell depletion<br>Steroid dependency<br>Immunosuppressive treatment	22	**Age at NS onset:** 2.96 ± 0.49 yrs (range 0.7 to12.6 yrs)<br>**Age at RTX:** 13.5 ± 0.68 yrs<br>**Duration of NS at RTX:** 10.6 ± 0.78 yrs (range 2.6 to 17.5 yrs)<br>**FU:** n.a., at least 12 months after RTX<br>17M, 5F<br>**Indications:** SDNS despite CNI (22 pts), LEV (7 pts.), CPH (15 pts.); disease duration > 8 yrs (18), steroid and/or CNI toxicity (13), non-compliance (12)	**RTX:** 1-4 doses of 375 mg/m^2 weekly (4x: 15 pts.; 3x: 2, 2x: 4; 1x: 1), subsequent infusions were given<br>**Duration:** 1-4 weeks<br>**Other:** tapering dose of steroids: all, CNI+MMF 17, CNI 3, MMF 2,	**Remission:** 9/22 at 12 months<br>**RR:** n.a.<br>**Time to relapse:** n.a.<br>**Withdrawal Pred. and IS:** 15/22 at 12 months<br>**B-cell depletion:** after 1st infusion: all<br>**Duration of B-cell depletion:** 7.1 ± 1.0 mths (1-2 doses) and 8.2 ± 0.7 mths (3-4 doses of RTX)<br>**AE:** infusion reaction 2, severe neutropenia 1, thrombosis peripheral veine 1, transient hepatic cytolysis 1, transient thrombocytopenia 1, Infections 2
Kemper 2012 [134] Germany, Austria (GPN)	Long-term follow-up after rituximab for steroid-dependent idiopathic nephrotic syndrome	Retrospective multicenter analyses	Nephrotic syndrome<br>Rituximab<br>Steroid sensitive	37	**Age at NS onset:** 2.9 (IQR, 1.3-12.5) yrs<br>**Age at RTX:** 13.4 (IQR, 6.4-18) yrs<br>**FU:** 29.4 (IQR, 9.2-92.8) mths after RTX; 25M, 12F<br>**Long-term FU:** 36 (IQR, 24-92.8) mths for 29/37 pts.<br>**Indications:** refractory SDNS despite LEV (8), CPH (25), CsA (34), MMF (26)	**RTX:** 1-4 doses of 375 mg/m^2/week (4x: 9; 3x: 1; 2x: 6; 1x: 21), subsequently repeated infusions<br>**Duration:** 1-4 weeks<br>**Other:** steroids in tapering dose, CNI. MMF	**Remission:** 26/37 (70.3%) at 12 months; 12/29 (41.4%) at 24 months<br>**Relapses:** 24/37 (64.8%) after initial RTX course<br>**Time to relapse:** 10.3 ± 3.5 mths (1-2 doses) and 23.3 ± 18.7 mths (3-4 doses) (p<0.05)<br>**Pred. dose:** discontinued in 35/37 (94.5%) after 1.3 (0.37-6) mths<br>**Withdrawal of IS:** 22/37 (59%)<br>**Time to relapse:** 9.6 (IQR, 5.2-64.1) months<br>**B-cell depletion:** n.a.<br>**AE:** mild infusion reaction 2
Tellier 2013 [135]	Long-term outcome of	Retrospective multicenter	Anti-CD20 monoclonal	18	**Age at NS onset:** 2.8 (IQR, 1.6-7.4) yrs	**RTX:** 1-4 doses 375 mg/m^2/week (4x: 10, 3x: 1,	**Remission:** 8/18 at 24 months,<br>**Duration of remission:**

1st author, year, [Ref.], country of origin	Title of Publication	Study design	Keywords	N	Population characteristics	Treatment	Outcomes
France	children treated with rituximab for idiopathic nephrotic syndrome	study	antibody<br>Steroid dependent idiopathic syndrome<br>Efficacy<br>Side effects<br>Follow-up<br>Children		**Age at RTX:** 13.5 (IQR, 5.9-18) yrs<br>**Duration of NS until RTX:** 10.4 (IQR, 3.5-16) yrs<br>**FU:** 3.2 (IQR, 2-5.3) yrs, at least 24 mths after 1st RTX infusion;<br>9M, 9F<br>**Indications:** SDNS, frequent relapses despite CNI, MMF, steroid and/or CNI toxicity, non-compliance	2x: 4; 1x: 3), subsequent doses given due to CD-10 cell recovery (54%) or relapse (41%), systematically (5%)<br>**Duration:** 1-4 weeks<br>**Other:** steroids in tapering dose: all, CNI+MMF (13), CNI (5)	depending on re-treatment with RTX; no: 19 months, 1: 17 mths; 2: 30 mths; 3: 23.5 mths; 6: 32 mths<br>**Relapses:** 10/18 at 24 mths<br>**Time to relapse:** 13 (IQR, 5-22) months after first RTX in 10/18 pts. and with CD19 count of 14 (IQR, 4-59)%.<br>**Withdrawal of Pred. and IS** at 24 mths: completely stopped (4), decrease of doses in all pts.: prednisone by 72% (29.8 vs. 8.5 $mg/m^2/day$), CNI by 63% (CsA 4.85 vs. 1.78 mg/kg/day), MMF by 20% (1306 vs. 1046 $mg/m^2/day$)<br>**B-cell depletion:** all<br>**AE:** infections 4, neutropenia 1, flare of psoriasis 1, behavioral disorders 1
Ruggenenti 2014 [136] Italy	Rituximab in steroid-dependent or frequent relapsing idiopathic nephrotic syndrome	Prospective multicenter study<br>5 centers		10 children (20 adults)	**Age at NS onset:** 3.1 (IQR, 2.2-5.7) yrs<br>**Age at RTX:** 11.7 (IQR, 9.5-13.6) yrs<br>**FU:** at least 12 mths after RTX<br>5M, 5F<br>**Indications:** SDNS, relapses despite CNI (9), MMF (7), Azathioprine (3), CPH (4); steroid toxicity	**RTX:** 1-2 doses of 375 mg/m^2/ week (based on B-cell depletion after 1 week); no repated infusions within 12 mths<br>**Duration:** 1-2 weeks<br>**Other:** steroids in tapering doses, CNI, CPH, diuretics, antihypertensives (incl. ACE-I, ARB)	**Remission:** 3/10 at 12 mths<br>**Relapses:** 7/10 (70%) at 12 mths<br>**RR:** All: decreased from 2.5 (IQR, 2-4) to 0.5 (IQR, 0-1) after RTX<br>**Time to relapse:**<br>**Pred. dose:** decreased from 0.27 mg/kg (IQR, 0.19-0.6) to 0 mg/kg (IQR, 0-0.23) ($p<0.001$) at 12 mths<br>**Other IS:** Decreasing cumulative yearly doses: CsA and MMF<br>**B-cell depletion:** completely after 1-2 doses; recovery from mth 6 to 1 yr.<br>**AE:** none
Sun 2014 [137] China	Efficacy of rituximab therapy in children with refractory	Prospective single center study	Refractory nephrotic syndrome, rituximab	12, of those 9 SDN	**Age at NS onset:** 1.6-8.9 yrs<br>**Age at RTX:** not stated<br>**FU:** 4-16 mths (average: 8	**RTX:** 1-2 doses of 375 mg/m^2/week (max. 500 mg) (2x 3; 1x 6 pts.)<br>**Duration:** 1-2 weeks	**Remission:** 8/10 at 6 mths<br>**RR:** decreased from 1.45 ± 0.52 to 0.18 ± 0.40 during 6 mths after RTX

1st author, year, [Ref.], country of origin	Title of Publication	Study design	Keywords	N	Population characteristics	Treatment	Outcomes
	nephrotic syndrome: a prospective observational study in Shanghai			S/FR NS	± 4 mths) after RTX<br>**Indications:** SDNS/ FRNS despite CPH, CNI, MMF	**Other:**	**Pred. doses:** stopped in 5/9 pts., reduced by > 50%: 2/4, < 50%: 2/4 at 6 mths.<br>**Other IS:** stopped after 6 mths in 9/9 pts<br>**B-cell depletion:** all after 2 weeks; B-cell count increased at average of 4.38 mths<br>**AE:** acute infusion reactions 5, mild hypogammyglonulinemia 1 (incl. infections)
Kamei K 2016 [138] Japan	Risk factors for relapse and long-term outcome in steroid-dependent nephrotic syndrome treated with rituximab	Retrospective study<br>Single center	Rituximab<br>Steroid-dependent nephrotic syndrome<br>SRNS<br>B-cell<br>Children	81	**Age at NS onset:** 4.2 (IQR 1.2-17.3) yrs, 5.9 ± 4.3 yrs<br>**Age at RTX:** 11.4 (3.1-21.8) yrs, 11.3 ± 4.9 yrs<br>**Duration of disease before RTX:** 4.0 (0.6-19.4) yrs, 5.4 ± 4.2 yrs<br>**FU:** at least 12 months after RTX; 38 (13-90) mths<br>57M, 24F<br>**Indications:** refractory SDNS (steroid dependence under IS), history of SRNS and later acquired steroid sensitivity and SDNS (n=39 (48%), primary n=16, secondary n=23), severe steroid toxicity	**RTX:** single dose of 375 mg/m^2, additional doses administered in case of relapses after B-cell recovery in 51 pts. (63%), 7 (9%) more than 5 additional doses<br><br>**Other:** oral steroids, continuing IS, IS at initial RTX:<br>Predn. 81 (100%)<br>CsA 67 (82.7%)<br>Mizoribine 51 (63%)<br>MMF 12 (14.8%)<br>Tacrolimus 4 (4.9%)<br>CPH/Chlorambucil/ Azathiopine: 0	**50%-relapse free survival:** 482 days<br>**Relapses:** 59 (73%) at 309 (0-1201) days after initial RTX<br>59 (73%) had 1-16 relapses during observation period (total: 260)<br>7 (9%) had relapses during B-cell depletion<br>8 (10%) had relapses with steroid resistance<br>**Relapse rate:**<br>Before RTX: 4.5 ± 1.9/yr<br>At 1yr after RTX: 0.9 ± 1.4/yr<br>At 2 yrs: 1.0 ± 1.4/yr<br>**Predn. Dose:**<br>Discontinuation possible in 69 (85%) without relapses at 66.5 (26-409) days<br>**IS:** all continued with IS, 44 started in MMF. 34 with MZR switched to MMF. No of pat. requiring decreased from 72 (89%) to 50 (62%) at 6 mths, and 43 (53% at 12 mths)<br>**B-cell depletion:** 78 of 81 after initial RTX<br>**Time to B-cell recovery:** 160

1st author, year, [Ref.], country of origin	Title of Publication	Study design	Keywords	N	Population characteristics	Treatment	Outcomes
							(IQR 39-311) days<br>**AE:** Mild infusion reactions 117 of 215 infusions (54%), late adverse events 15 pts (18.5%): agranulocytosis 9 (8 episodes with infections: antibiotics, G-CSF treatment), Pneum jirovecii 1, herpes stomatitis 1, chronic gastroenteritis 1, enterocolitos and diverticulitis 1, skin rash 1, chronic cough and gross hematuria 1.
Fujinaga 2017 [139] Japan	Predictors of relapse and long-term outcome in children with steroid-dependent nephrotic syndrome after rituximab treatment	Retrospective chart review	Rituximab<br>SDNS<br>Relapse<br>Long-term	43	**Age at NS onset:** 6.1 ± 3.6 yrs<br>**Age at RTX:** 11.0 ± 4.8 yrs<br>**FU:** 5.4 ± 1.6 yrs, at least > 2 yrs<br>29M, 14F<br>**Indications:** complicated SDNS despite use of IS incl. CsA, MMF, CPH, Mizoribine (24 (56%) had history of steroid-resistance)	**RTX:** Single dose of RTX (375 mg/m^2, max. 500 mg), additional doses administered in 28 (65%) pts. in case of relapse despite maintenance treatment (total 109 doses)<br><br>**Other:** prednisolone (in tapering dose for 6 mths), CsA (21) or MMF (11) or CsA+MMF (7) or mizoribine (4) (maintenance at least for 12 mths predn.-free remission, then tapered off)	**Treatment-free remission > 12 mths:** 5/43 (12%)<br>**1st relapse after RTX:** 39 (91%) after 586 (IQR 2-1450) days<br>**50% relapse-free survival:** 646 days<br>**Withdrawal of steroids:** 39 (91%) at 154 days after initial RTX<br>**B-cell depletion:** CD19 recovery at 150 (IQR 43-614) days;<br>Short B-cell depletion (<150 days) associated with high risk for 1st relapse after RTX (HR 2.5 (95% CI 0.92-5.71)<br>**AE:** neutropenia 3/43, 1of 3 required hospitalizytion due to acute infection
Topaloglu 2019 [140] Turkey	Rituximab for children with difficult-to-treat nephrotic syndrome: Its effects on disease progression and growth	Retrospective chart reviews<br>Single center	Rituximab<br>Nephrotic syndrome<br>Steroid<br>Cyclosporine<br>growth	41 (21 SSNS)	**Age at NS onset**: 5.8 ± 4.7 yrs<br>**Age at RTX:** 10.8 ± 5.1 yrs<br>**FU post-RTX:** 2.3 ± 1.6 yrs<br>10 M, 11 F<br><br>**Indication:** SDNS (n=21) despite CNI, SRNS (n=20)	**Only SSNS group:**<br>**1st course of RTX:** 375 mg/m^2 in<br>2 doses: 4<br>3 doses: 10<br>4 doses: 7<br>additional doses as maintenance in 10 (47.6%) every 6-12 mths	**Only SSNS group:**<br>**No. of relapses:** fell from 4 per 2yrs to 0 per 2 yrs after RTX (p<0.001)<br>**Relapses**: 8/21 (38%), independent from no. of initial RTX infusions<br>**Time to relapse:** 14.6 ± 11.7 mths

1st author, year, [Ref.], country of origin	Title of Publication	Study design	Keywords	N	Population characteristics	Treatment	Outcomes
						**Other:** after RTX maintenance IS with CNI 10/21, MMF 2/21	**Cumulative steroid dose**: fell from 117.8 mg/kg (75.6-152) (12 mths before RTX) to 34 mg/kg (20-48) (12 mths after RTX)<br>**Cumulative CsA dose:** fell from 811.5 mg/kg (594.8-937.5) to 583.5 mg/kg (305.8-903) (p=0.015)<br>**Median height z-score:** improved after RTX in 11/21 (52.3) from -1.2 (-2,5;-0.3) to -0.6 (-1.9; -0.1) (p=0.044)<br>**Median BMI z-score:** decreased after RTX in 15/21 (71.4%) from 1.6 (0.9-3.0) to 1.1 (-0.7; 2.5) (p=0.007)<br>**AE:** mild infusion reaction 1
**Different RTX regimen/ dosing of RTX**							
Hogan 2019 [141]<br>France	Effect of different rituximab regimens on B cell depletion and time to relapse in children with steroid-dependent nephrotic syndrome	Retrospective study	Idiopathic nephrotic syndrome<br>Children<br>B cell<br>Rituximab	61	54/61 analysed (monthly B cell count required)<br><br>Group 1: n=8<br>Group 2: n=35<br>Group 3: n=18<br><br>**Age at NS onset:** n.a.<br><br>**Age at RTX:**<br>Group 1: 7.6 (IQR 6.4-10.8) yrs, 5M, 3F<br>Group 2: 13.8 (9.8-15.3) yrs, 24M, 11F<br>Group 3: 10.1 (8.3-12.8) yrs, 8M, 10 F<br><br>**FU:** at least 12 mths after RTX<br><br>**Indications:** SDNS despite	**RTX:**<br>**Group 1:** 1 dose of 100 mg/m^2<br>**Group 2:** 1 dose of 375 mg/m^2<br>**Group 3:** 2 doses of 375 mg/m^2<br><br>**Duration:** see above<br><br>**Other:** steroids in tapering dose, MMF, CNI	**Relapse-free survival at 1yr:** Total cohort: 60% (48-72), associated with time to B-cell reconstitution: HR of relapse 0.78 (0.63-0.97) per month of B cell depletion)<br>Group 1: 50% (58-77)<br>Group 2: 59% (42-76)<br>Group 3: 72% (46-87)<br>**B-cell depletion:** complete after initial RTX, otherwise pat. excluded<br>**Time to B-cell reconstitution:**<br>Group 1: 2.5 (IQR 1.8-3.5) mths<br>Group 2: 5.0 (3.9-6.0) mths<br>Group 3: 6.6 (4.6-7.8) mths<br>**Factors associated with time to B-cell recovery:**<br>Age at RTX: HR per year 0.87 (0.80-0.95)<br>RTX regimen (ref. group 3):

1st author, year, [Ref.], country of origin	Title of Publication	Study design	Keywords	N	Population characteristics	Treatment	Outcomes
					prior treatments (Methylpredn. Pulses 21%, MMF 62%, CPA 20%, CNI 62%)		HR group 1: 6.19 (1.42-7.2)<br>HR group 2: 1.94 (1.04-3.63)<br>**AE:** not stated
Maxted 2019 [142] UK	Low-dose rituximab is no less effective for nephrotic syndrome measured by 12-month outcome	Multicenter retrospective observational cohort study<br>3 center	Rituximab<br>Nephrotic syndrome<br>Dosing	60	**RTX (1st course):**<br>**Low dose:** n=40<br>**Intermediate dose:** n=5<br>**High dose:** n=14<br><br>Total of 143 RTX courses:<br>1 course: n=19<br>2 courses n=17<br>3 courses: n=11<br>4 courses: n=5<br>5 courses: n=8<br><br>**Age at NS onset:** 4 (IQR 1-14) yrs<br>**Age at RTX:** 11 (4-17) yrs<br>**FU:** 6-24 mths after 1st RTX course<br>38M, 22F<br>**Indications:** SDNS, FRNS – received at least one dose of RTX, additional IS administered	**RTX:**<br>**Low dose:** 375 mg/m^2, given once<br>**Intermediate dose:** all other regimens<br>**High dose:** total of 1.5 g/m^2 (750 mg/m^2 in 2 doses or 375 mg/m^2 in 4 doses) given in 4-week period<br><br>26 received subsequent doses prophylactically at 179 (51-540) days)<br><br>**Duration:** 4-week period<br>**Other:** prednisolone in tapering dose, CNI, MMF, CNI+MMF	**Relapse-free survival:**<br>6 mths: Low 30/37 (81%), Interm. 5/5 (100%), High 12/14 (85.7%)<br>**12 mths**: Low 13/34 (47%), interm. 4/5 (71%), high 7/14 50% (p=0.06)<br>24 mths: low: 2/30 (6.7%), Interm. ¾ (75%), 4/14 (38.6%)<br>**Time to relapse:**<br>Low: 334 days, Interm. >720 days, high: 344 days<br>**Time to B-cell reconstitution:**<br>Total cohort: 295 days; low: 290, interm.: 304, high: 259 days)<br>Excluding courses (54 of 117 courses) without B-cell recovery: 226 days<br>**AE:** minimal infusion reactions: several, clinically relevant 1; persistent hypogammaglobulinemia 2 8of those requiring IVIG 1)
Takahashi T 2019 [143] Japan	Periodically repeated rituximab administrations in children with refractory nephrotic syndrome: 2-year multicenter observational study	Prospective study<br>5 centers	Nephrotic syndrome<br>Children<br>Rituximab<br>Repeated administration<br>Mizoribine<br>Calcineurin inhibitor	22	**Age at NS onset:** 3.9 (2.7-5.4) yrs<br>**Age at RTX:** 11.2 (9.0-13.0) yrs<br>**Duration of NS before RTX**: 7.3 (3.8-8.9) yrs<br>**FU:** 2yrs after initial RTX<br>14 M, 8 F<br>**Indication:** refractory FRNS or SDNS despite CNI (all, for 5.8 (3.5-8.4) yrs) and/or other IS agents (MZR 19, CPA 11), steroid	**RTX:** single dose of 375 mg/m^2 (max. 500 mg); repeated 4 times at 6-month intervals<br>**Duration:**<br>**Other:** start of tapering of CNI with 1st RTX dose, discontinued 1 week later; tapering dose of oral steroids; MZR twice a week (500 mg/550 mg) after RTX**,** prophylaxis during B-cell depletion with	**Relapse-free survival rate** at 1yr and 2 yrs: 50% and 46%<br>**Survival rate without FRNS/SDNS** at 1 yr and 2yrs: 91% and 86%<br>**RR:** decreased from 5.8/pt./2yrs to 1/pt/2yrs (p<0.001)<br>**AE:** mild infusion reactions after 47% (41/88) of RTX administration; agranulocytosis 1/22 (4.5%), transient neutropenia 3 (13.6%), exacerbation of atopic dermatitis 3

1st author, year, [Ref.], country of origin	Title of Publication	Study design	Keywords	N	Population characteristics	Treatment	Outcomes
					toxicity	Sulfamethoxazole-trimethoprim and fluconazole; antihypertensive agents	(13.6%), steroid withdrawal syndrome 1(4.5%), electrocardiographic change 3 (13.6%) (neg. conversion of T wave 2; ST elevation 1) abnormalities 3; infectious episode (influenza 8, mycoplasma 1, other viral 16)
Chan 2020 [144] International	Both the rituximab dose and maintenance immunosuppression in steroid-dependent/ frequently-relapsing nephrotic syndrome have important effects on outcomes	Multicenter retrospective study<br>11 centers (Asia, Europe, North America)	Biologics<br>Children<br>Immunosuppression<br>Nephrotic syndrome<br>Rituximab<br>Steroid-dependent nephrotic syndrome	511	**Low dose:** n=191<br>**Medium dose:** n=208<br>**High dose:** n= 112<br><br>**Age at NS onset:** 3 (IQR 2.1-4.9) yrs<br>**Age at RTX:** 11.5 (IQR 8.1-14.3) yrs<br>**FU:** at least 18 mths; 4.3 (IQR, 2.7-5.9) yrs after RTX<br>342M, 168 F<br>**Indications:** complicated SDNS, FRNS despite IS with CNI, CPA, levamisole, MMF	**RTX:**<br>**Low:** 375 mg/m^2 (with mIS: n=145, without: 46)<br>**Medium**: 750 mg/m^2 (with mIS: n=91, without n=117)<br>**High**: 1125-1500 mg/m^2 (with mIS: n=47, without: n=65)<br><br>**Other:** with (283, 55%) or without (228, 45%) maintenance immunosuppression (mIS) (including oral steroids, MMF, CNI) at first relapse or for at least 6 mths after RTX	**Relapse-free survival:**<br>Similar among 3 dosing groups (log-rank test p=0.36)<br>Low: 11.8 (10.1-15.8) mths<br>Medium: 11.9 (10.4-14.3) mths<br>High: 13.0 (11.8-17.4) mths<br>Shorter in low dose group without mIS than in medium/high+mIS: 8.5 (7.2-13.3) mths vs. 12.7 (10.4-16.9)/14.3 (12.0-18.4) mths (log-rank, p=0.03)<br>**Relapses:** in 412 pts. (81%) after RTX<br>**Relapse-free period:** 12.5 mths (95% CI, 11.3-14)<br>**Risk factors of relapse:**<br>Low dose without mIS: HR_{adj} 0.50-0.6 (95% CI, 0.33-0.94, $p \le 0.023$)<br>Age at RTX: HR_{adj} 0.95 (95% CI, 0.93-0.98; p=0.002) per 1-yr increase in age<br>Previous IS: HR_{adj} 1.19 (95% CI, 1.05-1.35; p=0.006) for each additional IS agent prior RTX<br>**Other IS:**<br>**Without mIS:** withdrawal of steroids after RTX at 3 (2-4) mths, CNI at 2 (1-3) mths, MMF 0 (0-0.5) mths<br>**With mIS:** 1 mIS (165, 58%), 2 mIS (81, 29%), 3 mIS (37, 13%).

1st author, year, [Ref.], country of origin	Title of Publication	Study design	Keywords	N	Population characteristics	Treatment	Outcomes
							Steroids (159, 56%), CNI (135, 47%), MMF (144, 51%) given for 7 (5.8-10.2) mths.<br>**B-cell depletion:** in 97% (442 of 454) after 7 and 14 days, similar among different regimens (p=0.47)<br>**Additional RTX courses:** required in 327 (67%) after 1.2 yrs (IQR, 0.8-2 yrs) due to relapses (87%), B-cell-recovery (7%), non-compliance/ IS toxicity (6%). Of those, 245 on mIS.<br>**AE:** in 85 of 511 (16%). Acute infusion reaction: 67, early infusion termination: 12, Infection 20 (1 pneumocystis jirovecii), neutropenia 13, hypogammaglobulinemia 56
**Observational studies comparing RTX with other steroid-sparing agents**							
**RTX vs. TAC**							
Sinha 2012 [145] India	Short-term efficacy of rituximab versus Tacrolimus in steroid-dependent nephrotic syndrome	Prospective single center comparison study	Focal segmental glomerulosclerosis Minimal change disease proteinuria	23	**RTX Group: n=10**<br>**Tac Group: n=13**<br><br>**Age at NS onset:** 3.6 ± 1.5 yrs (RTX); 3.6 ± 2.2 yrs (Tac)<br>**Age at SDNS:** 5.0 ± 2.0 yrs (RTX); 4.4 ± 2.2 yrs (Tac)<br>**Age at RTX/Tac:** 12.2 ± 2.3 yrs (RTX), 12.3 ± 3.0 yrs (Tac)<br>**FU:** 15.9 ± 4.7 mths (RTX); 19.7 ± 4.8 yrs (Tac)<br>18 M, 5 F<br>**Indications:** SDNS despite therapy with at least 2 different agents (LEV 1-2	**RTX:** 1-3 doses of 375 mg/m^2/ week aiming for complete B-cell depletion<br><br>**Tac:** 0.1-0.2 mg/kg/day in 2 divided doses for 12 mths, targeting trough levels 4-7 ng/ml<br><br>**Co-treatment:** steroids in tapering dose	**Remission:** similar in both groups at 12 mths. (RTX: 50%, Tac 46.2%; p=0.63)<br>**No. of relapses:** similar in both groups at 6 and 12 mths<br>**RR:** similar decline in both groups: RTX: from 3.1 ± 1.1 to 0.8 ± 1.0 relapses/yr (95% CI 1.3-3.3, p<0.001)<br>Tac: from 3.5 ± 1.6 to 0.9 ± 1.1 relapses/yr (95% CI 1.7-3.7; p<0.001)<br>**Time to relapse:** shorter in RTX than in Tac (8.5 ± 5.1 vs. 9.8 ± 5.6 mths) (95% CI –4.7; 7.3; p=0.65)<br>**Steroid dose:** similar reduction of cumulative in both groups; discontinued in 8/10 RTX pts. And

1st author, year, [Ref.], country of origin	Title of Publication	Study design	Keywords	N	Population characteristics	Treatment	Outcomes
					yrs; CPH 12 wks; MMF 1-2 yrs), “difficult-to-treat NS”, steroid toxicity		6/13 Tac pts.<br>**B-cell depletion (RTX):** data available in 3 pts, recovery at 4-12 mths<br>**AE: RTX:** infusion reaction 3, **Tac:** reversible nephrotoxicity 1
**RTX vs. CPA**							
Webb 2016 [146] UK	Cyclophosphamide and rituximab in frequently relapsing/ steroid-dependent nephrotic syndrome	Retrospective chart reviews<br>Single center	FRNS<br>SDNS<br>Cyclophosphamide<br>Rituximab<br>Outcome	102	CPA only: n=59<br>RTX: n=42, of those 34 after CPH, 8 RTX only<br><br>**Age at treatment:**<br>CPA: 6 yrs<br>RTX: 7.5 yrs<br>CPA+RTX: 11 yrs<br><br>79 M, 34 F<br><br>**Indication:** FRNS/ SDNS despite LEV, CNI, MMF, steroid toxicity	**CPA:** dosage not stated<br><br>**RTX:** Single (n=10) or two doses (n=32) of 750 mg/m^2, 2 weeks apart<br><br>**Co-treatment**: steroids in tapering dose, LEV, CNI, MMF	**Remission at 24 mths:** CPH: 24%, RTX: 32%<br>**RR:**<br>**Time to relapse:**<br>CPH 7 mths<br>RTX 14 mths<br>**Withdrawal of steroids:**<br>CPH: 67 (84%) weanded off at 3 mths<br>RTX: 36 (86%) weaned off at 3 mths<br>**Time off-predn. To relapse**:<br>CPH: 3 mths<br>RTX: 12 mths<br>**B-cell depletion (RTX): n.a.**<br>**AE:**<br>**CPH:** neutropenia 3, hair loss, hemorrhagic cystitis 1<br>**RTX:** allergic infusion reaction 2
Kari 2020 [147] Saudi-Arabia	Rituximab versus Cyclophosphamide as first steroid-sparing agent in childhood frequently relapsing and steroid-dependent nephrotic syndrome	Prospective non-randomized study<br><br>2 center	Nephrotic syndrome<br>Rituximab<br>Cyclophosphamide	46	CPA n=27<br>RTX n=19<br><br>**Age at NS onset:** n.a.<br>**Age at medication start:**<br>**CPA:** 68.2 (55.1-81.2) mths<br>**RTX:** 86.2 (66.7-105.6) mths<br>**FU:** at least 12 months after CPA/RTX<br>**Indications:** FRNS/SDNS, only steroids with/without	**CPA:** 3 mg/kg/day orally for 8 weeks<br><br>**RTX:** 2 doses of 375 mg/m^2/dose, 2 weeks apart<br><br>**Duration:** see above<br><br>**Other:** oral steroids a.d., ACEi (CPA: 47.4%, RTX 74.1%)	**Relapse-free survival at 1yr:**<br>CPA 17/27 (58.6%)<br>RTX 16/19 (84.2%)<br>**Withdrawal of steroids at 3 mths:**<br>CPA 8 (29.6%), RTX 14 (73.7%) (p<0.003)<br>**Reduction of a.d. steroid dose:**<br>CPA: from 1.02 to 0.36 mg/kg (p<0.001)<br>RTX: from 0.86 to 0.08 mg/kg (p<0.001)<br>**Time to relapse:**

1st author, year, [Ref.], country of origin	Title of Publication	Study design	Keywords	N	Population characteristics	Treatment	Outcomes
					levamisole before		CPA: 6.9 mths (10/27, 37%), RTX (3/19, 15.8%): 6.3 mths<br>**AE:**<br>CPA: transient leucopenia (6, 22%), acute hepatotoxicity (1, 3.5%)<br>RTX: mild infusion reaction (1, 5.2%)
**Different agents including RTX**							
**After RTX: CsA vs. MMF**							
Fujinaga S 2013 [86], Japan	Cyclosporine versus Mycophenolate mofetil for maintenance of remission of steroid-dependent nephrotic syndrome after a single infusion of rituximab	Prospective study<br>Single center	Cyclosporine<br>Mycophenolate mofetil<br>Rituximab<br>Steroid-dependent nephrotic syndrome	29	**CsA after RTX:** n=13<br>**MMF after RTX:** n=16<br><br>**Age at onset of NS:** 6.4 ± 3.9 yrs<br>**Age at RTX:** 11.8 ± 4.3 yrs<br>**FU:**<br>19M, 10 F<br>**Indication:** severe SDNS despite CsA (for 49±35 mths) and/or MMF<br>Immunosuppr. agents at RTX: CsA 13, MMF 11, CsA and MMF 4, CsA and MZR 1	**RTX:** Single dose of 375 mg/m^2 (max. 500 mg)<br>**Duration of B-cell depletion**: 5 mths<br><br>**After RTX:**<br>**CsA group**: dose adjusted to C2 level of 400-500 ng/ml; mean dose 3.7 mg/kg/d. MMF and MZR discontinued.<br>**Duration** after RTX: 18 (5-29) mths<br>**MMF group:** adjusted to target MPA levels of 2-5 µg/ml. CsA discontiinued<br>**Duration** after RTX**:** 19 (7-44) mths<br>**Other:** Tapering dose of steroids	**Relapse after RTX:**<br>CsA: 3/13<br>MMF: 12/16<br>**Treatment failure:**<br>CsA: 2/13<br>MMF: 7/16<br><br>**RR:**<br>CsA: decrease from 4.4 ± 1.9 to 0.6±1.4/yr (86%, p<0.01)<br>MMF: decrease from 2.3±0.8 to 1.0/±0.9yr (58%; p<0.01)<br><br>**Steroid dose:**<br>CsA: decrease from 0.35±0.16 to 0.057±0.14 mg/kg/day (p<0.01)<br>MMF: decrease from 0.38±0.26 to 0.15±0.21 mg/kg/d (p<0.01)<br><br>**Dose of agent after RTX:**<br>CsA: decrease from 4.6 to 3.7 mg/kg/d (21%, p<0.01).<br>MMF:<br><br>**AE:**<br>RTX: transient infusion reaction: 13/29 (45%),<br>CsA. Hypertrichosis all. Mild CsAN 3

1st author, year, [Ref.], country of origin	Title of Publication	Study design	Keywords	N	Population characteristics	Treatment	Outcomes
							MMF: diarrhea 2, bacterial pneumonia 1
Fujinaga S 2015 [87], Japan	Positive role of rituximab in switching from cyclosporine to mycophenolate mofetil for children with high-dose steroid-dependent nephrotic syndrome	Prospective study<br>Single center	SDNS<br>Rituximab<br>Cyclosporine<br>MMF	26	**Age at NS onset:** 7.0 ± 4.0 yrs<br>**Age at CsA:** 8.3 ± 4.1 yrs<br>**Age at MMF:** 12.1 ± 4.0 yrs<br>**FU:** after MMF start: 28.8 ± 9.9 mths<br>16M, 10F<br>**Indication:** complicated SDNS despite CsA (for 46.5±27.2 mths), CsAN in 11 pts (42%) with CsA > 24 mths	**MMF** started at initial dose 250 mg/12h, adkusted to MPA trough level of 2-5 µg/ml (max. 1g bd).<br>After MMF start; CsA doseage gradually tapered<br>Duration:<br>Other: tapering dose of steroids, steroids fro relapses.<br>**In case of MMF treatment failure: RTX** (n=11)**:** single dose of 375 mg/m^2 (max. 500 mg)<br>**Duration**:<br>**Other:** tapering dose of steroids, steroids for relapse	**Remission** despite CsA withdrawal: 11/26 (42%)<br>**MMF Failure and RTX:** 11/26 (42%)<br>**Sustained remission > 1 yr** without steroids: 22/26 (85%)<br>**Discontinuation of MMF:** 15/26 (58%)<br>**RR:** with CsA: 1.0±0.9/yr; with MMF and RTX 0.7±0.5/yr (p=0.07)<br>AE: MMF: mild gastrointesinal symptoms 2, herpes simplex 2<br>RTX: mild infusion reactions 5/11, late-onset neutropenia requiring G-CSF 1/11
**Immunological aspects of RTX (B cell recovery, hypogammaglobulinemia**)							
Delbe-Bertin 2013 [148] France	Does rituximab induce hypogammaglobulinemia in patients with idiopathic nephrotic syndrome?	prospective single-center study	Rituximab<br>Immunoglobulin G<br>Hypogammaglobulinemia<br>Nephrotic syndrome<br>Immunosuppression	12	**RTX group** with minimal B cell depletion of 3 mths, aiming for 18 mths: n=12<br>**Oral IS Group;** n=16<br><br>**Age at NS onset:**<br>RTX: 7.9 ± 5.6 yrs<br>Oral IS: 5.7 ± 3.7 yrs<br><br>**FU**: at least 6 mths after 1st RTX<br><br>**Indication:** SDNS, CNI dependency	**RTX group:** 1-4 doses of 375 mg/m^2, CD19 depletion controlled after 7 days with flow cytometry assay;<br>In case of reappearance: additional dose of RTX (mean 4.2 ± 2.48 infusions)<br>**Other:** MMF/CsA/CPA: 12/12<br><br>**Oral IS Group:** oral steroids, MMF and/or CsA/ Tac	**Serum IgG-Levels:**<br>**RTX:** 8/12 decreased IgG level before RTX, of those 7 with persistent Hypo-IgG during B-cell depletion.<br>4/12 with normal IgG levels before RTX and during B-cell depletion.<br>**Oral IS:** normal IgG levels at baseline and last FU. Transient episodes of low IgG levels 5/16, prolonged > 3 mths in 2.<br>**Infection risk/ IVIG:**<br>**RTX:** 2/12 received IVIG during Hypo-IgG as prevention for infections. 1 aseptic meningitis<br>Oral IS: no infectious complications requiring hospitalization. Single IVIG 1.

1st author, year, [Ref.], country of origin	Title of Publication	Study design	Keywords	N	Population characteristics	Treatment	Outcomes
Delbet 2019 [149]<br>France	Idiopathic nephrotic syndrome and rituximab: may we predict circulating B lymphocytes recovery	Retrospective study<br>Single center	Nephrotic syndrome<br>Rituximab<br>Children<br>B cell depletion	22	**Age at NS onset:** 4.15 (1.4-14.6) yrs<br>**Disease duration before 1st RTX**: 3.1 (0.4-14.8) yrs<br>**Age at RTX**: 10.4 (4-16.6) yrs<br>**FU:**<br>17 M, 5 F<br><br>**Indication:** SDNS (n=18) or SRNS (n=4) despite previous IS (prednisone 22, MP Pulses 14, LEV 11, CsA 18, MMF 17, oral CPA 9)	**RTX:** 1-4 doses of 375 mg/m^2<br><br>**Other:** oral steroids in tapering dose (21), CNI+MMF (14), CsA (3), MMF (2), Tac (1)<br>-> stopped 2 mths after RTX	**Total durationof B cell depletion**: 26 (6-66) mths<br>**Individual B cell depletion duration post-RTX:** 5.1 (1.6-14) mths<br>**Comparison in each patient (1st and next B cell depletion)**:<br>59% of next B cell depletion: same duration within interval ± 1 mth<br>70% within interval ± 2 mths<br><br>No difference in B cell depletion duration between groups who received 1-2 and 3-4 infusions
Colucci 2016 [150]<br>Italy	B Cell reconstitution after rituximab treatment in idiopathic nephrotic syndrome	Retrospective comparative study<br>Single center	Rituximab<br>Nephrotic syndrome<br>B cell reconstitution	28, compared to 28 healthy controls	**Age at NS onset:** 5.22 ± 0.72 yrs<br>**Age at RTX**: 13.68 ± 0.77 yrs<br>**FU:** 11.2 (8-17.7) mths<br>18 M, 10 F<br><br>**Indication**: SDNS, FRNS despite previous IS (CNIs 17 (60.7%), MMF 21 (75%), Azathioprine (1 (3.6%)	**RTX:** 1 dose (24, 85.7%) or 2 doses (4, 14.3%) of 375 mg/m^2<br><br>**Other:** steroids in tapering dose, IS (MMF, CNI) gradually tapered<br><br>**Aim:** Determining by flow cytometry levels of B and T cell subpopulations before and after RTX	**Relapses during 24 mths after RTX:** 14 pts. (50%), of those 4 before 9 mths, 4 9-12 mths, 6 > 12 mths<br><br>**% of total lymphocytes for each B cell subpopulation**:<br>**Baseline:** no differences in total B cells, memory cells, igM memory, switched memory B cells; but lower transitional and mature B cells.<br>**Day 2-7 and 1 mth after RTX:** complete depletion (<1% of total lymphocytes) after single RTX infusion<br><br>**B-Cell recovery:** total B-cells reappeared at median 6 mths after RTX; completely recovered after 12 mths (transitional, mature and finally memory B cells; very slow recovery of total memory, IgM memory, switched memory B

1st author, year, [Ref.], country of origin	Title of Publication	Study design	Keywords	N	Population characteristics	Treatment	Outcomes
							cells), more delayed in non-relapsers<br><br>**Total CD3+ Tcells** at baseline: normal range, no variations at 12 months<br>**$CD4^+/CD8^+$ cell ratio:** lower range of normal at baseline, normalized 12 mths after RTX<br><br>**Predictors of Relapse:**<br>Significant association with recovery of switched memory cells and time to relapse (HR 3.45, 95% CI 1.39-8.54, p<0.01).<br>At 9 mths: Pts. with switched memory B cells <0.067% of total lymphocytes had a significantly lower risk of relapse within 24 mths (p<0.001)
Colucci M 2019 [151], Italy	Prolonged Impairment of Immunological Memory after anti-CD20 treatment in pediatric nephrotic syndrome	Retrospective observational study	Immunologic memory, hypogammaglobulinemia, B cells, clinical immunology, pediatric nephrology, idiopathic nephrotic syndrome	27	**Anti-CD-20 treatment:** n=27<br>**Prolonged oral IS:** n=21 (= “control group”)<br><br>**Age at NS onset:** 5.1 (2.0-13.7) yrs<br>**Age at 1st anti-CD20 treatment:** 12.9 (5.8-21.2) yrs<br>**Age at last FU:** 19.1 (9.6-27.0) yrs<br>**FU:** at least 4 yrs after 1st RTX or Ofatumumab (74 (48-118) mths), and 2 yrs after last infusion (70 (26-113) mths)<br>18 M, 9F<br>**Indication**: SDNS (n=25),	**Anti-CD20-treatment:**<br>**RTX** 1st dose with 375 mg/m^2<br>Additional doses given in case of relapses, 11/27 with multiple infusions (≥ 2), of those received 2 ofatumumab (1500 $mg/1.73m^2$)<br><br>**Other:** steroids in tapering dose, IS gradually tapered	**Relapses** within 3 yrs (6-32 mths): 20 (74%)<br>**Relapse rate at last FU:** reduced from 3.4 (1-5) relapses/yr to 0.6 (0-2) per year (p<0.001).<br>**Withdrawal of steroids/IS at last FU:** 10/27 discontinued all IS, 6/27 on prednisone, 4/27 on CNI, 14/27 on MMF, 2/27 on prednisone and 2 agents.<br><br>**B-cell recovery:** at last FU all recovered, but distributions of B cells subsets altered (anti-CD 20 pts. reduced proportion of memory B-cell compartment) compared to “control groups”.<br>Delayed switched memory B-cell reconstitution more in

1st author, year, [Ref.], country of origin	Title of Publication	Study design	Keywords	N	Population characteristics	Treatment	Outcomes
					FRNS (n=2), despite previous IS (CNI, MMF, CNI+MMF)		nonrelapsers.<br><br>**Serum Ig Levels (**at baseline and last FU): 6/13 had low IgG at baseline and last FU (<600 mg/dl), 1 developed severe hypogammaglobulinemia (IgG <160 mg/dl). 5/13 with *de novo* severe hypogammaglobulinemia, of those 3 requiring IVIG.<br>*De novo* IgA deficiency in 4/13 IgG/IgA-def. independent of no. of RTX infusions.<br>No low IgG or IgA levels in “control group” with only oral IS.<br>Risk factor for hypogammaglubulinemia: younger age at 1st RTX infusion (OR: 2.14/yr, 95% CI 1.25-3.68, p=0.006)<br>Severe hypogammaglobulinemia more frequent in nonrelapsers.<br><br>**Vaccine Competence:**<br>Reduced IgG levels against HV and tetanus at baseline, further declined at last FU. Antigen-specific memory B-cells were induced by re-immunizytion but specific IgG titers remained low.<br><br>**AE:** 18/27 (67%), infections 12, moderate low IgG level (<700 mg/dl) 7, severe low IgG (<160 mg/dl) 4.<br>Higher infection rate associated with CNI as maintenance IS but only slightly corralted with low IgG at last FU.

1st author, year, [Ref.], country of origin	Title of Publication	Study design	Keywords	N	Population characteristics	Treatment	Outcomes
Parmentier 2020 [152] France	Immunoglobulin serum levels in rituximab-treated patients with steroid-dependent nephrotic syndrome	Retrospective chart review	Hypogammaglobulinemia Immunoglobulin G Infection Nephrotic syndrome Rituximab	107	**Age at NS onset:** 3.1 (IQR, 2.24-5.45) yrs<br>**Age at RTX:** 11.7 (8.6-14.2) yrs<br>**FU:** 4.02 (2.7-5.8) yrs after 1st RTX<br>70 M, 37 F<br><br>**Indications:** difficult-to-treat SDNS despite prior IS (CPA 29%, MMF 74.8%, CNI 86.9%. LEV 19.6%)	**RTX: 375 mg/m^2/ dose**<br>Single dose: 11/107<br>Multiple doses: 96/107<br>No. of RTX infusions: 4 (IQR 3-5)<br><br>**Other:**	**Hypo-IgG:**<br>**Before RTX:** 21/107 (19.6%), Infections 4/21, remained 1 yr after B-cell recovery 8/21<br>**During RTX:** 25 (23.4%), 1st episode at 15 (7.4-36.2) mths after 1st RTX, Infections 9/25, remained 1 yr after B cell recovery 13/25<br><br>**Risk factor for Hypo-IgG after RTX initiation:** younger age at RTX initiation (<7 yrs)<br><br>**Risk for infections with Hypo-IgG:** younger age at RTX initiation: 6.5 (5.2-14.6) yrs in group with concomitant infections vs. 10.3 (7.2-12.4) yrs in group without inf.<br><br>**Duration B-cell depletion:** 19.8 (13.2-26.4) months with 1500 mg/m^2 (IQR 1125-1875) cumulative RTX dose<br><br>**AE:** Infections in 13/46 with Hypo-IgG: 7 with hospitalization. Pneumonia 5, fulminant viral myocarditis 1, viral meningitis 1, ENT infectiosn 4, Varicella 1, EBV 1.
**RTX – adverse events in nephrotic syndrome**							
Fujinaga S, 2016 [153] Japan Only abstract	Late-onset adverse effects after a single dose of rituximab in children with	Retrospective study Single center		60	**Age at NS onset:**<br>**Age at RTX:**<br>**FU**:<br>**Indication**: complicated SDNS	**RTX:** single dose of 375 mg/m^2<br>Additional doses given (total 126 doses) in case of relapse/ B-cell recovery	**Severe neutropenia** (neutrophil count < 500/mm^3): 3<br><br>**Hypo-IgG (< 500 mg/dl)**: 9, of those requiring hospitalizations 2

1st author, year, [Ref.], country of origin	Title of Publication	Study design	Keywords	N	Population characteristics	Treatment	Outcomes
available	complicated SDNS					Other: oral steroid in tapering dose for 6 mths, after RTX maintenance continued with CsA orMMF	due to bacterial infections<br><br>**B-cell depletion:** 5 (1-20) mths
Maeda R 2018 [154] Japan	Serum sickness with refractory nephrotic syndrome following treatment with rituximab	Case report	SDNS<br>Refractory nephrotic syndrome<br>Rituximab<br>Human anti-chimeric antibodies<br>Serum sickness	Case report	**Age at NS onset:** 7 yrs<br>**Age at RTX:** 14 yrs (after 9 relapses)<br>**Age at RISS**: 17 yrs<br>1F<br>**Indication**: refractory SDNS, treated with CsA and RTX and prednisolone (prior treatment with MZR)	**RTX:** 375 mg/m^2 as single dose<br>**Duration**: remission after RTX for 24 mths,then 1oh relapse, repeating RTX infusions)<br>**Other:** CsA, oral steroids	Symptoms 10 days after 5th RTX infusion: fever, rash, arthralgia (RTX-induced serum sickness: RISS, caused by elevated levels of human anti-chimeric antibodyes (HACAs), produced after antigen exposure

Table S8.6: Mizoribine (MZR)

1st author, year, [Ref.], country of origin	Title of Publication	Study design	Keywords	N	Population characteristics	Treatment	Outcomes
**MIZORIBINE (MZR)**							
Ohtomo Y, 2005 [155] Japan	High-dose mizoribine therapy for childhood-onset frequently relapsing steroid-dependent nephrotic syndrome with cyclosporine nephrotoxicity	Retrospective chart review Single center	Cyclosporin nephropathy Frequent relapsing steroid-dependent nephrotic syndrome Mizoribine	9	**Age at NS onset:** 38.0 (22-95) mths **Age at MZR:** 17.8 (13-20) yrs **FU:** at least 12 mths after MZR **Indications:** FRNS, SDNS despite prior CsA and after long-term CsA (108.4 (46-206 mths); CsA-dependence) with moderate to severe CsAN	**MZR:** 10.1 (6.97-16.44 mg/kg/day **Duration:** at least 12 mths **Other:** CsA, oral steroids	**Relapses:** 2/9 relapse-free**,** 5/9 steroid-responsive relapses, 2/7 MZR failure and developed SRNS **RR:** decreased from 2.33 ± 0.71/yr to 1.11 ± 0.78/yr **Cumulative CsA-dose:** 7/9 pts.: reduced from 3.2±0.83 to 1.75±0.79 mg/kg/day **Cumulative steroid dose:** 0.21±0.07 and 0.19±0.11 mg/kg/d **AE:** herpes zoster 1/9
Kawasaki Y, 2005 [156] Japan	Oral mizoribine pulse therapy for patients with steroid-resistant and frequently relapsing steroid-dependent nephrotic syndrome	Retrospectibve chart review Single center	Children Clinical Mizoribine oral pulse therapy Nephrotic syndrome Steroid-resistant NS	8	**Age at NS onset: 5.0** ± 2.4 yrs **Age at MZR:** not stated **Duration of NS before MZR:** 68 ± 25 mths **FU:** range 13-24 mths **Indications:** CsA-dependent SDNS, prior CPA (4)	**MZR:** 10 mg/kg/day in 3 divided doses (max. 500 mg/d) on 2 days a week **Duration:** 12-24 mths **Other:** oral steroids a.d., CsA	**Remission:** 4/8 without further relapses, 2/4 discontinued CsA and steroids, 2/4 CsA; 4/8 “non-responder” with further relapses **RR:** not stated **AE:** uricacidaemia 1 (controlled with allopurinol)
Fujinaga S, 2011 [157] Japan	Single daily high-dose mizoribine therapy for children with steroid-dependent nephrotic syndrome prior cyclosporine administration	Retrospective analysis 2 centers	High-dose mizoribine Steroid-dependent nephrotic syndrome Cyclosporine	10	**Age at NS onset:** 3.2 ± 1.3 yrs **Age at MZR:** 6.2 ± 2.6 yrs **FU:** 33 mths 9 M, 1F **Indications:** SDNS before CsA administration, prior agents CPM (4)	**MZR:** 5 mg/kg as single dosis, adjusted to 2-hr post-dose mZR level of 3 (2-5) µg/ml (max. 300 mg/day); mean dose; 8.4 mg/kg/day **Duration:** 22 (10-30) mths **Other:** oral steroids in tapering dose, CPA (1), no ACEi/ARB	**Remission:** **RR:** reduced from 3.0 (1-3)/yr to 0.4 (0-1.2)/yr **Time to relapse:** **Steroid dose:** reduced from 0.78 ± 0.32 to 0.31 ± 0.22 mg/kg alternate daily **AE:** not stated
Xia 2019 [158], China	Usefulness of mizoribine administration in children with frequently	Prosepctive multicenter observational study 4 centers	Frequently relapsing nephrotic syndrome Mizoribine	82	**Age at NS onset:** 5.0 ± 2.9 yrs **Age at MZR:** 8.1 ± 3.4 yrs **FU:** 12 mths after MZR start **Indications:** FRNS	**MZR:** 5 mg/kg/day (max. 150 mg) in a single dose **Duration:** **Other:** oral steroids. ACEi/ARB	**RR:** fell from 3.7±1.3/yr to 0.8±0.8/yr (p<0.001) **Steroid dose:** decreased from 41.5 ± 15.8 to 6.6±10.1 mg/day (p<0.01)

1st author, year, [Ref.], country of origin	Title of Publication	Study design	Keywords	N	Population characteristics	Treatment	Outcomes
	relapsing nephrotic syndrome, and the relationship netween pharmacokinetic parameters and efficacy: a multicenter prospective cohort study in China		Pharmacokinetic parameters				**AE:** 11/82 (13.4%) mild adverse events, of those 4 discontinued MZR due to allergy, nausea, vomiting, abdominal distension, 7 with hyperuricemia
Mizutani A, 2019 [159] Japan	Positive effects of single-daily high-dose mizoribine therapy after cyclophosphamide in young children with steroid-dependent nephrotic syndrome	Retrospective analysis	Mizoribine<br>Cyclophosphamide<br>SDNS<br>Young children	54	**A: MZR after CPA:** n=36<br>**B: CPA only:** n=18<br><br>**Age at NS onset:** 3.4 (1.1-8.5) yrs (A), 4.6 (1.3-7.9) yrs (B)<br>**Age at CPA:** 5.9 (1.5-9.5) yrs (A), 5.6 (2.9-8.4) yrs (B)<br>**FU:** > 2 yrs, 5.9 yrs<br>43 M, 11 F<br>**Indications:** SDNS	**A: MZR after CPA:**<br>**CPA:** 2-2.5 mg/kg/day for 12 wks<br>MZR: 5 mg/kg/day (max. 150 mg/d); 2-hr post-dose MZR levek >3 μg/ml<br>**Duration:** MZR 12 mths, then tapered off<br><br>**B: CPA only:** 2-2.5 mg/kg/day for 12 wks<br>**Other:** oral steroids in tapering dose	**Sustained remission at 2 yrs:** A: 21/36; B: 4/18 (58% vs. 22%, p<0.05)<br>**Rate of regression to SDNS**: 9/36 (A), 7/18 (B) (6% vs. 39%, p<0.05)<br>**RR:** not reported<br>**AE:** none
Kondoh T, 2019 [160] Japan	Assessment of factors associated with mizoribine responsiveness in children with steroid-dependent nephrotic syndrome	Retrospective analysis<br>2 centers	Mizoribine<br>Frequent-relapse nephrotic syndrome<br>Steroid-dependent NS<br>Children	47	**Age at NS onset:** 4.5 ± 2.4 yrs (responder)/ 5.4±3.4 yrsv (non-resp.)<br>**Age at MZR:** 7.4 ± 3.1 yrs/ 8.6± 4.8 yrs<br>**FU:**<br>43 M, 4 F<br>**Indications:** SDNS without prior treatment with steroid-sparing agents	**MZR:** 4 mg/kg twice a day (responder: 4.6 mg/kg, non-resp.: 4.9 mg/kg)<br>**Duration:**<br>**Other:**	**Remission:**<br>MZR Responder 16/47<br>Non-responder: 31/47<br>**RR:** MZR-responder: decreased from 3.5/yr to 1.8/yr<br>Non-resp.: 4 4/yr vs. 4.7/yr<br><br>No differences in clinical characteristics and pharmacokinetics between resp./ non-resp.<br>**AE:** not stated

Table S8.7: Other agents

1st author, year, country of origin	Title of Publication	Study design	Keywords	N	Population characteristics	Treatment	Outcomes
**VINCRISTINE (VCR)**							
Kausman 2005 [161] Australia	Vincristine treatment in steroid-dependent nephrotic syndrome		Vincristine Nephrotic syndrome	12	**Age at NS onset:** 3 (2.1-6.5) yrs<br>**Age at VCR:** 13.1 (9.9-14.4) yrs<br>**Duration of NS before VCR**: 8.2 (3.9-11.5) yrs<br>**FU:** 1.9 (1.2-4) yrs after VCR<br>8 M, 4 F<br>**Indications:** SDNS despite prior CPA (all), CsA (10), LEV (2), steroid toxicity	**VCR:** 1-1.5 mg/m^2 IV weekly for 4 wks, then monthly for 4 mths<br>**Duration:** 5 mths<br>**Other:** oral steroids, CsA	**Remission:** good response with achieving remission 7/12, poor response with still in relapses 5/12<br>**Duration of remission:** 0.4 (0.3-2.0) yrs<br>**RR:** fell from 4.0 (2-4)/yr to 1.5 (0-2.9)/yr (p=0.004)<br>**Time to relapse:** 5 mths<br>**AE:** abdominal pain 2 (at 1.5 mg/m^2, resolved with 1 mg/m^2); extravasation burn with no long-term injury 1, alopecia 1. Constipation (no. not stated)
Krishnan 2005 [162] UK	Is there a role for vincristine in nephrotic syndrome - letter to the editor	Retrospective chart review as letter to the editor	Vincristine Nephrotic synddrome	17, of those 8 SDNS	**Age at NS onset**: 5.3 (1.2-14.4) yrs<br>**Age at VCR**: not stated<br>**FU:** 2 yrs after VCR<br>11 M, 6 F<br>**Indication**: SDNS (n=8), SRNS (n=9); prior treatments CPA (12), LEV (4)	**VCR:** 1.5 mg/m^2 weekly for 8 weeks<br>**Duration**: 8 weeks<br>**Other:** low dose oral steroids	**Complete remission**:<br>SDNS: 2/8 (within 2 and 5 mths)<br>SRNS: 1/9 (within 2yrs of treatment)<br>**AE:** 4/17 jaw pain (3 requirining carbamazepine), 2/17 constipation, 1/17 seizure, 1/17 foot-drop lastin 6 mths
**SAQUINAVIR**							
Coppo 2012 [163] Italy	Saquinavir in steroid-dependent and –resistant nephrotic syndrome: a pilot study	Prospective pilot study 2 centers	Antiproteasome drugs; antiviral drugs; immunoproteasome; nuclear factor κB (NF-κB); treatment of nephrotic syndrome	6	**Age at NS onset:**<br>**Age at Saquinavir**: 13.5 (7-38) yrs<br>**Duration of NS before Saquinavir**: 6.3 (1.3-14.9) yrs<br>**FU:** 14.7 (6-68.7) mths after Saquinavor<br>4 M, 2 F<br>**Indication:** SDNS (3 with secondary steroid resistance), prior treatments with MP Pulses, CPA, CsA/ TAC, MMF, plasma exchange, RTX,	**Saquinavir:** 30 mg/kg/d<br>**Duration**: at least 6 mths<br>**Other:** low doses of CNI (CsA 2mg/kg/d or TAC 0.01-0.06 mg/kg/d); small doses of steroids	**Favourable effects:** 4/6 became infrequent relapsers, 1/6 frequent relapse wih 63% **prednisone reduction** (from 25.3 to 8.3 mg/kg/mth, p=0.015)<br><br>**AE**: mild diarrhea 2

1st author, year, country of origin	Title of Publication	Study design	Keywords	N	Population characteristics	Treatment	Outcomes
					ACTH: Toxicity of steroids/ other agents		
**ACTH**							
Chakraborty 2020 [164]	ACTH treatment for management of nephrotic syndrome: A systematic review and reappraisal	Systematic review	ACTH<br>Nephrotic syndrome		**FSGS (9 studies, 98 pts)**<br>Madan 2016<br>Tumlin 2013<br>Hogan 2013<br>Alhamad 2019<br>Bomback 2011 and 2012<br>Filippone 2016<br>Berg 1999<br>Lorusso 2015<br><br>**MCGN (7 studies, 14 pts**)<br>Madan 2016<br>Filippone 2016<br>Bomback 2011 and 2012<br>Khastgir 2015<br>Berg and Arnadottir 2004<br>Lorusso 2015<br><br>**MPGN (4 studies, 13 pts)**<br>Berg and Arnadottir 2004<br>Lorusso 2015<br>Bomback 2012<br>Madan 2016	**Acthar gel:** 40-80 units twice or three times a week for 4-6 mths<br>**Or**<br>**Synthetic ACTH**: 1 mg once a week for 12 mths	Studies in summary:<br>**FSGS:**<br>Compl. remission: 9/98 (9.2%)<br>Partial rem.: 33/98 (33.6%)<br>Some proteinuria reduction 6/98 (6.1%)<br>**AE:** increased swelling/edema 7, hyperglycemia 3, weight gain 4, myalgia 1, muscle cramps 4, hypertension 5, rash 2, temporary increase in skin pigmentation 1, dyspepsia 2, mood alteration 4, early termination of treatment 2<br><br>**MCGN:**<br>Compl. Rem.: 8/14 (57.1%)<br>Partial rem.: 3/14 (21.4%)<br>**AE:** none<br><br>**MPGN:**<br>Compl. Rem.: 7/13 (53.8%)<br>Partial rem.: 1/13 (7.7%)<br>**AE:** early termination of treatment 1, otherwise none
**AZATHIOPRIN**							
**CPA – CsA – MMF – LEV - AZA**							
Moustafa BH 2016 [101]<br>Egypt	Immunosuppressive therapy in children with steroid-resistant, frequently-relapsing, and steroid-dependent idiopathic	Retrospective chart review<br>Single center	Childhood Nephrotic Syndrome<br>Steroid Resistance<br>Steroid Dependence<br>Relapse	79	**CPA:** n=28<br>**CsA:** n=6<br>**MMF:** n=2<br>**LEV:** n=40<br>**AZA:** n=10<br><br>**Age at NS onset:** 3.7 (1.3-10.5) yrs	**CPA:** 2-3 mg/kg/d orally for 8-12 wks or IV as monthly bolus of 500-750 mg/m^2 for 6 mths<br>**CsA:** 4-6 mg/kg/d divided into 2 doses for at least 12 mths<br>**MMF:** 1200 mg/m^2/day	**Remission for 6 mths:**<br>**CPA:** 24/28 (85.7%)<br>**CsA:** 5/6 (83.3%)<br>**MMF:** ½ (50%)<br>**LEV:** 22/40 (55%)<br>**AZA:** 8/10 (80%)<br><br>**AE** (not differentiated between

1st author, year, country of origin	Title of Publication	Study design	Keywords	N	Population characteristics	Treatment	Outcomes
	nephrotic syndrome: a single center experience		immunosupp ressants		**Age at medication:** not stated<br>**FU:**<br>44 M, 35 F<br>**Indications:** SDNS/ FRNS, steroid toxicity	divided into doses<br>**LEV:** 2-2.5 mg/kg/dose twice weekly for 6-24 mths<br>AZA: 2 mg/kg/d for 8 wks.<br><br>Either given as 1st or 2nd line drug; some given in double- or triple-combination therapy<br><br>**Other:** oral steroids	SDNS (n=79) and SRNS (n=51)):<br>CPA: Leukopenia 15/63 (23.8%), hemorrhagic cystitis 2/63 (3.2%)<br>CsA: gym hyperplasia 8/31 (25.8%), hirsutism 7/31 (22.6%), nephrotoxicity 2/31 (6.4%), hypertension 2/31 (6.4%)<br>MMF: diarrhea 7/12 (58.4%), nausea 3/12 (25%), abdominal pain 1 (8.%), cough 1/12 (8.3%)<br>LEV: none<br>AZA: Leukopenia 2/10, diarrhea 2/10, abdominal pain 2/10, arthralgia 1/10

Table S9: Adverse effects and impact of alkylating agents on pubertal children

Please not that these studies are difficult to interpret as varying doses (mostly higher than 168mg/kg) were used, and stratification according to pubertal staging is rather unclear.

High FSH and LH levels are taken as surrogate markers of germinal epithelial injury intended to reflect azoo / oligo-spermia in some of the studies. In other testicular biopsies are done, or sperm counts checked several years after treatment.

Study	Disease	Cum. Dose	Effect
Lentz 1977 [165]	MLNS, FSGS, MGN, MGN, SLE		Data on boys only
	CP at ages:<br>PrePub 5 to 12<br>Pubertal 11-16<br>PostPub 13.5 to 17<br><br>Patients studied after puberty completion	High dose >365mg/kg	azoospermia:<br>2/2 PrePubertal<br>1/2 Pubertal<br>3/3 PostPuberal<br><br>"FSH and LH were normal in one patient in the high-dose group who had normal semen (Patient 15), and in one of the seven high dose patients with azoospermia (Patient 7)"
		Low dose < 365mg/kg	NO azoospermia<br>Oligospermia in:<br>1/2 PrePubetarl<br>2/6 Pubertal (one with multiple congt anomalies)<br>1/1 PostPubertal<br><br>FSH and LH were normal in all low-dose patients, including those with oligospermia
		No gonadal injury at doses <168mg/kg	
Pennisi 1975 [166]	INS<br><br>1.5 to 5.5 yrs after CP	Most patients rcvd >168mg/kg<br><br>One Pre-pub pt dosed 168mg/kg had normal Ts Bx	Boys: Pre or early Pubertal (16) : All had normal FSH, LH and Ts levels. Testicular bx was abnormal in 4/5<br><br>Boys: Pubertal (7) : 5 had increased FSH. Spermatogenesis was diminished in all 7, 1 had azoospermia.<br><br>Girls (11, 7 pre and 4 pubertal) :No evidence of gonadal dysfunction was detected in any of the girls

Kirkland 1976 [167]	NS<br><br>Studied 8 months to 7 yrs after CP course	Total dose 1.6 to 25.5 gm	Males<br>PrePubertal (15) - no abnormalities of basal serum levels of LH, FSH<br><br>Pubertal (5) elevated mean basal values of gonadotropins with normal testosterone levels and elevated LH responses to LRF; the FSH responses to LRF were elevated in four patients
Penso 1974 [168]	NS<br><br>Studied 1-4 years after CP	total doses 70 to 860mg/kg<br><br>6/7 treated for >6 months<br>1/7 treated for 9 weeks	Age 11-18 years<br><br>4 PrePubertal, 3 Pubertal<br><br>5/7 were oligospermic or azoospermic

Table S10 : Studies of long-term outcome of childhood-onset SSNS

Study	No (M:F)	Country	Selection	Diagnosis years	FU duration (yrs)	Relapse adulthood	Risk factors	ESRD (n)
Trompeter 1985 [169]	152 (108:44)	UK	Bx MCNS	1963-1969	21.3 (14-32)	6.8%	Onset age < 6 years	0
Lewis 1989 [170]	45 (>16 yrs)	UK	Bx MCNS	1963-1976	14 (10-21)	19.2 (>20yo) -26.7 (>16yo) %	none	0
Takeichi 1997 [171]	34 (24:10)	Japan	Bx MCNS	NA	>6	26%	Not analyzed	1
Fakhouri 2003 [172]	102	France	SSNS	NA	NA	42.2% > puberty	No. of relapse during childhood	0
Ruth 2005 [173]	42	Swiss	SSNS	1970-2003	22.0 (2.9-39)	33%	Use of CsA	0
Kyrieleis 2007 [95]	93 (adult 15)	Nether-lands	CPM, Bx MCNS	1971-2003	8 (1-39)	29% after CPM (adult ??)	Onset age < 3 years	0
Skrzypczyk 2014 [174]	55	Poland	SSNS	1970 - 2010	6 -38	16.4%	No. of relapse during childhood	
Korsgaard 2019 [175]	39	Denmark	SSNS	1998-2015	mean 14.4 (7.8–19.3)	31%	SD/FR	0
Aydin 2019 [176]	43	Germany	SSNS	1957-1995	33.6 (14.4–50.8)	9.3%	Not relevant	0
Carter (2020) [177]	301	Canada	INS	1993 – 2016	3.9 (IQR 2.1-6.6)	22.3%	Not relevant	1.2%

Table S11: Semiquantitative expression of typical dipstick results (van der Watt, Ped Neph 7th ed. 2016)

Dipstick results	Proteinuria
**Negative**	0 to <15 mg/dl
**Trace**	15 to <30 mg/dl
**1+**	30 to <100 mg/dl
**2+**	100 to <300 mg/dl
**3+**	300 to <1000 mg/dl
**If 4+**	>/=1000 mg/dl

Table S12 Future Research Recommendations

Topic	Subtopic	Research Question
**1st episode of NS**	Treatment with PDN	Compare the effectiveness of treatment with oral PDN for 8 (4+4) weeks or shorter duration vs. 12 (6+6) weeks (daily/alternate daily PDN) in terms of outcomes such as time to first relapse, FRNS and SDNS
		Compare the effectiveness of initial treatment with 30 mg/m^2 (1 mg/kg) for 4 weeks & alt day for 4 wks with 60 mg/m^2 (2 mg/kg) for 4 weeks & alt day for 4 wks
		Determine if initial PDN duration >12 weeks affects future disease course in very young children
	PDN Dosing	Evaluate the dosing of PDN by weight or BSA for outcomes of effectiveness such as inducing remission, time to first relapse, FRNS, SDNS and steroid toxicity
	Steroid-sparing agents	Assess combination therapy of PDN with a steroid-sparing agent at disease onset to determine effectiveness of reducing time to remission, increasing time to first relapse or development of FRNS or SDNS
	Pharmacology, Pharmacokinetics, Pharmacogenomics	Determine the mode of action of glucocorticoids and other immunosuppressive medications in SSNS
		Examine the pharmacokinetics of prednisone by age
		Determine the role of pharmacogenomics in guiding selection of dose and duration of prednisone, and second line immunosuppressive agents
	Risk stratification	Identify biomarkers or genetic risk haplotypes to stratify disease subgroups and to assist in selection of appropriate therapeutic agents
**Relapses**	Treatment with PDN	Determine the minimum dose and duration of PDN for treatment of steroid sensitive relapses in order to regain and maintain remission, reduce PDN exposure, toxicity, and improve quality of life
	PDN Dosing	Evaluate the effectiveness of dosing of prednisone by weight or BSA in inducing remission and reducing steroid toxicity
	Low dose daily versus low-dose alternate day PDN dosing	Compare the efficacy and safety of low-dose daily versus low-dose alternate day PDN dosing as long-term maintenance treatment to prevent relapses.
	Prevention of relapses	Optimize treatment protocols for SSNS after relapse according to clinical phenotypes addressing important demographic variables such as age, sex and ethnicity.
		Determine if administering PDN treatment at the start of an infection is effective to maintain remission and prevent

Topic	Subtopic	Research Question
		relapse
	Prevention of URTI associated relapses	Determine the effectiveness of short term escalation of immunosuppression for prevention of upper respiratory tract infection (URTI) associated relapses in IRNS, FRNS, SDNS<br>Determination of the risk of URTI-associated relapse for large cohorts of children to understand differences according to ethnicity, geography and disease course.
	Adrenal function	Evaluate prevalence and incidence of adrenal insufficiency in children with SSNS at different points in their disease history, in terms of both symptoms of adrenal insufficiency and its impact on risk of relapses.
	General impact of relapses	Measure the pattern of relapses in different populations to better understand the incidence of complex relapses and the impact of relapses on quality of life and health economics.
**FRNS/ SDNS**	Treatment with steroid-sparing agents	Compare the efficacy of different immunosuppressive therapies in maintaining sustained remission and reducing the frequency of relapses in order to determine how and when the different immunosuppressive therapies should be used.<br><br>Perform a RCT assessing antiCD20 agents, such as obinutuzumab, belimumab, daratumumab in comparison to RTX or as adjunctive therapy to other steroid-sparing agents.
	Duration of treatment	Determine the optimal duration of therapies of LEV, MMF, CNI
	Levamisole - side effect	Assess the risk of ANCA positive vasculitis
	MMF - drug monitoring	Determine the utility and benefits of drug monitoring
	Rituximab - safety, dosing, monitoring	Determine the safety of therapy with RTX, specifically the risk of transient or sustained hypogammaglobulinemia, and other serious adverse effects<br><br>Determine the optimal RTX individual dose in children<br><br>Examine the efficacy of sequential administration of RTX in maintaining remission and safety in order to determine the optimal number and timing of RTX retreatment<br><br>Examine the immune phenotype of B-lymphocytes following rituximab induced remission and during relapse<br><br>Evaluate the importance of monitoring for RTX plasma levels and anti-chimeric RTX antibodies

Topic	Subtopic	Research Question
**Drug toxicity**		Devise validated objective scores to measure acute and chronic corticosteroid toxicity.<br><br>Compare toxicity from corticosteroids and non-steroid immunosuppression to help guide changes in maintenance treatment.
**Genetics**	Familial SSNS	Examine the genetic basis of SSNS, focusing on families with steroid sensitive disease
**Adjunctive measures**	Vaccination	Determine the efficacy and safety of live attenuated vaccines in children on maintenance immunosuppressive therapy
	Edema	Determine the efficacy of albumin and/or diuretics in the management of severe edema
**Health outcomes**	Quality of life	Assess the quality of life in all clinical trials as a patient centered endpoint
	Long-term safety	Assess the cumulative risk of late side effects from NS immunosuppression therapy
	Adult outcomes	Assess the impact of childhood onset NS in adulthood

Table S13: Competencies expected in a young adult at the time of transition

- I understand my condition and can describe it to others
- I know my medications and what they are for
- I can make decisions for myself about my treatment
- I know what the adult clinic arrangements are and who will be reviewing me in clinic
- I know how to make my appointments
- I can make my own transport arrangements to get to the hospital for appointments
- I know who to call in a medical emergency
- I am able to talk about my worries concerning blood tests and other treatments
- I know the dietary advice that I have to follow and the importance of activity
- I have appropriate knowledge about sexual health matters
- I have discussed alcohol, smoking and drug issues

Adapted from [178]

Table S14 : The Transition scale, a tool to monitor the progression in transition competence.

The Transition Scale is a mechanism to assess and monitor progress in achieving the goals of transition: the ability for the adolescent/young adult to provide her/his own self-management and not be reliant on parental care.

**T**ype (of illness)
**R**x
**A**dherence
**N**utrition
**S**elf-management skills
**I**nformed reproductive health
**T**rade/school
**I**nsurance
**O**ngoing support
**N**ew health care providers

The score is determined by a professional member of the renal unit who designates for the young person a subscore of 0-1 (0=no ability, 0.5 = partial ability, 1=desired ability) for each component. The total score can be used to monitor progress over time, and the subscores can be used to identify gaps that need to be addressed.
https://www.readysteadygo.net/

https://www.readysteadygo.net/uploads/4/7/8/1/47810883/hello-to-childrens-services-ready-print-ready.pdf

Table S15: Definition differences between children and adults

Term	Definition in children < 16 years	Changes during transition
Nephrotic syndrome	Nephrotic-range proteinuria and either hypoalbuminemia (serum albumin <30 g/L (< 3 g/dL)) or edema when serum albumin is not available	Patients will be transferred to other hospitals, and must be made aware that laboratory assays differ between hospitals. Therefore, the values they are used to may change.<br>In the new KDIGO guideline, a remark with respect to **differences between albumin assays** were made, with a bias of 5-7 g/L between BCG and BCP/immunometric methods<br><br>In adults, 30g/l as cut off to define nephrotic syndrome and 3.5g/day of proteinuria (or > 3g/10mmol creatinine) is used.
Steroid sensitive nephrotic syndrome (SSNS)	Complete remission within 4 weeks of prednisone or prednisolone (PDN) at standard dose<br>(60 mg/m^2/day or 2 mg/kg/day, maximum 60 mg/day)	In adults, the usual dose to treat a nephrotic syndrome is 1 mg/kg/day, with a maximum of 80 mg.<br>The adult nephrologists suggest to allow more than 1 mg/kg/day in patients 16-18 years, but to use a maximum dose of 80 mg. e.g a 17 year old boy of 80 kg should not receive 60 mg
Relapse	Urine dipstick ≥ 3+ (≥300 mg/dl) or UPCR ≥ 200 mg/mmol (≥ 2 mg/mg) on a spot urine sample on 3 consecutive days, with or without reappearance of edema in a child who had previously achieved complete remission	Quantifying proteinuria in adults is preferred; fever and severe exercise can induce some proteinuria, and if there are no symptoms (no edema) a wait and see strategy is possible, hesitating to treat too early. This is especially relevant in patients with infrequent relapses. Not each episode of limited proteinuria should count as a serious and relevant relapse. Patients sometimes relapse in the period after steroid withdrawal, in such instance they can respond to a short course of steroids e.g 5 days 30 mg; such a relapse is considered as not serious and this should not be counted as relapse in the sense of defining FRNS.

Term	Definition in children < 16 years	Changes during transition
Remission	**Complete remission:** UPCR (based on first morning void or 24 hour urine sample) ≤ 20 mg/mmol (0.2 mg/mg) or <100 mg/m^2 per day, respectively, or negative or trace dipstick on three or more consecutive days<br><br>**Partial remission:** UPCR (based on first morning void or 24 h urine sample) > 20 but < 200 mg/mmol (>0.2 mg/mg but <2 mg/mg) and serum albumin ≥ 30 g/L (≥ 3 g/dL)	Patients with a longstanding history of treated nephrotic syndrome often develop persistent low grade proteinuria (micro-albuminuria). In experience of adult nephrologists this is not evidence of disease activity but rather reflects secondary FSGS as a result of podocyte loss incurred during the nephrotic episodes.<br>Therefore, it is necessary to quantify albuminuria or proteinuria in the period of "remission"

Evaluation system for competence of the patient for transition is required. The consensus statement of IPNA and ISN presented such a system, TRxansition scale [178].

REFERENCES:

1. Hahn D SS, Willis NS, Craig JC, Hodson EM (2020) Corticosteroid therapy for nephrotic syndrome in children. The Cochrane database of systematic reviews 2020(8):CD001533.
2. APN 1988. Short versus standard prednisone therapy for initial treatment of idiopathic nephrotic syndrome in children. Arbeitsgemeinschaft für Pädiatrische Nephrologie. Lancet 1988 1:380-383
3. Ekka BK, Bagga A, Srivastava RN (1997) Single- versus divided-dose prednisolone therapy for relapses of nephrotic syndrome. Pediatr Nephrol 11:597-599
4. Li X, Li Z, Cheng Z (1994) Treatment of children with simple nephrotic syndrom using prednison once per day. Acta Academiae Medicinae Hubei 15:386-388
5. Borovitz Y, Alfandary H, Haskin O, Levi S, Kaz S, Davidovits M, Dagan A (2020) Lower prednisone dosing for steroid-sensitive nephrotic syndrome relapse: a prospective randomized pilot study. Eur J Pediatr 179:279-283
6. Sheikh S, Mishra K (2021) Low-dose versus conventional-dose prednisolone for nephrotic syndrome relapses: a randomized controlled non-inferiority trial. Pediatr Nephrol 10:3143-3150.
7. Kansal A MM, Yadav S (2019) Effectiveness of a low dose prednisolone regimen for treatment of relapses in children with SSNS (abstract no: IPN10471-84). Pediatr Nephrol 2019; 34(10):1871.
8. Yadav M, Sinha A, Khandelwal P, Hari P, Bagga A (2019) Efficacy of low-dose daily versus alternate-day prednisolone in frequently relapsing nephrotic syndrome: an open-label randomized controlled trial. Pediatr Nephrol 34:829-835
9. Raman V, Krishnamurthy S, Harichandrakumar KT (2016) Body weight-based prednisolone versus body surface area-based prednisolone regimen for induction of remission in children with nephrotic syndrome: a randomized, open-label, equivalence clinical trial. Pediatr Nephrol 31:595-604
10. Basu B, Bhattacharyya S, Barua S, Naskar A, Roy B (2020) Efficacy of body weight vs body surface area-based prednisolone regimen in nephrotic syndrome. Clin Exp Nephrol 24:622-629
11. Abeyagunawardena AS, Trompeter RS (2008) Increasing the dose of prednisolone during viral infections reduces the risk of relapse in nephrotic syndrome: a randomised controlled trial. Arch Dis Child 93:226-228
12. Gulati A, Sinha A, Sreenivas V, Math A, Hari P, Bagga A (2011) Daily Corticosteroids Reduce Infection-associated Relapses in Frequently Relapsing Nephrotic Syndrome: A Randomized Controlled Trial. Clin J Am Soc Nephrol 6:63-69
13. Mattoo TK, Mahmoud MA (2000) Increased maintenance corticosteroids during upper respiratory infection decrease the risk of relapse in nephrotic syndrome. Nephron 85:343-345
14. Christian MT, Webb NJA, Mehta S, Woolley RL, Afentou N, Frew E, Brettell EA, Khan AR, Milford DV, Bockenhauer D, Saleem MA, Hall AS, Koziell A, Maxwell H, Hegde S, Prajapati H, Gilbert RD, Jones C, McKeever K, Cook W, Ives N (2021) Evaluation of Daily Low-Dose Prednisolone During Upper Respiratory Tract Infection to Prevent Relapse in Children With Relapsing Steroid-Sensitive Nephrotic Syndrome: The PREDNOS 2 Randomized Clinical Trial. JAMA pediatrics. 2022 Mar 1;176(3):236-243
15. Gargiulo A, Massella L, Ruggiero B, Ravà L, Ciofi Degli Atti M, Materassi M, Lugani F, Benetti E, Morello W, Molino D, Mattozzi F, Pennesi M, Maringhini S, Pasini A, Gianoglio B, Pecoraro C, Montini G, Murer L, Ghiggeri GM, Romagnani P, Vivarelli M, Emma F (2021) Results of the PROPINE randomized controlled study suggest tapering of prednisone treatment for relapses of steroid sensitive nephrotic syndrome is not necessary in children. Kidney Int 99:475-483
16. Kainth D, Hari P, Sinha A, Pandey S, Bagga A (2021) Short-Duration Prednisolone in Children with Nephrotic Syndrome Relapse: A Noninferiority Randomized Controlled Trial. Clin J Am Soc Nephrol 16:225-232
17. Abeyagunawardena AS, Thalgahagoda RS, Dissanayake PV, Abeyagunawardena S, Illangasekera YA, Karunadasa UI, Trompeter RS (2017) Short courses of daily prednisolone during upper respiratory tract infections reduce relapse frequency in childhood nephrotic syndrome. Pediatr Nephrol 32:1377-1382
18. Moher D, Pham B, Jones A, Cook DJ, Jadad AR, Moher M, Tugwell P, Klassen TP (1998) Does quality of reports of randomised trials affect estimates of intervention efficacy reported in meta-analyses? Lancet 352:609-613

19. Schulz KF, Chalmers I, Hayes RJ, Altman DG (1995) Empirical evidence of bias. Dimensions of methodological quality associated with estimates of treatment effects in controlled trials. JAMA 273:408-412
20. Larkins NG, Liu ID, Willis NS, Craig JC, Hodson EM (2020) Non-corticosteroid immunosuppressive medications for steroid-sensitive nephrotic syndrome in children. The Cochrane database of systematic reviews 4:Cd002290
21. Ahn YH, Kim SH, Han KH, Choi HJ, Cho H, Lee JW, Shin JI, Cho MH, Lee JH, Park YS, Ha IS, Cheong HI, Kim SY, Lee SJ, Kang HG (2018) Efficacy and safety of rituximab in childhood-onset, difficult-to-treat nephrotic syndrome: A multicenter open-label trial in Korea. Medicine 97:e13157
22. Iijima K, Sako M, Nozu K, Mori R, Tuchida N, Kamei K, Miura K, Aya K, Nakanishi K, Ohtomo Y, Takahashi S, Tanaka R, Kaito H, Nakamura H, Ishikura K, Ito S, Ohashi Y (2014) Rituximab for childhood-onset, complicated, frequently relapsing nephrotic syndrome or steroid-dependent nephrotic syndrome: a multicentre, double-blind, randomised, placebo-controlled trial. Lancet 384:1273-1281
23. Boumediene A, Vachin P, Sendeyo K, Oniszczuk J, Zhang SY, Henique C, Pawlak A, Audard V, Ollero M, Guigonis V, Sahali D (2018) NEPHRUTIX: A randomized, double-blind, placebo vs Rituximab-controlled trial assessing T-cell subset changes in Minimal Change Nephrotic Syndrome. J Autoimmun 88:91-102
24. Ravani P, Magnasco A, Edefonti A, Murer L, Rossi R, Ghio L, Benetti E, Scozzola F, Pasini A, Dallera N, Sica F, Belingheri M, Scolari F, Ghiggeri GM (2011) Short-Term Effects of Rituximab in Children with Steroid- and Calcineurin-Dependent Nephrotic Syndrome: A Randomized Controlled Trial. Clin J Am Soc Nephrol 6:1308-1315
25. Basu B, Sander A, Roy B, Preussler S, Barua S, Mahapatra TKS, Schaefer F (2018) Efficacy of Rituximab vs Tacrolimus in Pediatric Corticosteroid-Dependent Nephrotic Syndrome: A Randomized Clinical Trial. JAMA Pediatr 172:757-764
26. Ravani P, Rossi R, Bonanni A, Quinn RR, Sica F, Bodria M, Pasini A, Montini G, Edefonti A, Belingheri M, De Giovanni D, Barbano G, Degl'Innocenti L, Scolari F, Murer L, Reiser J, Fornoni A, Ghiggeri GM (2015) Rituximab in Children with Steroid-Dependent Nephrotic Syndrome: A Multicenter, Open-Label, Noninferiority, Randomized Controlled Trial. J Am Soc Nephrol 26:2259-2266
27. Ravani P, Lugani F, Pisani I, Bodria M, Piaggio G, Bartolomeo D, Prunotto M, Ghiggeri GM (2020) Rituximab for very low dose steroid-dependent nephrotic syndrome in children: a randomized controlled study. Pediatr Nephrol 35:1437-1444
28. Hoyer PF, Brodeh J (2006) Initial treatment of idiopathic nephrotic syndrome in children: prednisone versus prednisone plus cyclosporine A: a prospective, randomized trial. J Am Soc Nephrol 17:1151-1157
29. Niaudet P (1992) Comparison of cyclosporin and chlorambucil in the treatment of steroid-dependent idiopathic nephrotic syndrome: a multicentre randomized controlled trial. The French Society of Paediatric Nephrology. Pediatr Nephrol 6:1-3
30. Ponticelli C, Edefonti A, Ghio L, Rizzoni G, Rinaldi S, Gusmano R, Lama G, Zacchello G, Confalonieri R, Altieri P, et al. (1993) Cyclosporin versus cyclophosphamide for patients with steroid-dependent and frequently relapsing idiopathic nephrotic syndrome: a multicentre randomized controlled trial. Nephrol Dial Transplant 8:1326-1332
31. Ishikura K, Ikeda M, Hattori S, Yoshikawa N, Sasaki S, Iijima K, Nakanishi K, Yata N, Honda M (2008) Effective and safe treatment with cyclosporine in nephrotic children: a prospective, randomized multicenter trial. Kidney Int 73:1167-1173
32. Iijima K, Sako M, Oba MS, Ito S, Hataya H, Tanaka R, Ohwada Y, Kamei K, Ishikura K, Yata N, Nozu K, Honda M, Nakamura H, Nagata M, Ohashi Y, Nakanishi K, Yoshikawa N (2014) Cyclosporine C2 monitoring for the treatment of frequently relapsing nephrotic syndrome in children: a multicenter randomized phase II trial. Pediatr Nephrol 9:271-278
33. Dorresteijn EM, Kist-van Holthe JE, Levtchenko EN, Nauta J, Hop WC, van der Heijden AJ (2008) Mycophenolate mofetil versus cyclosporine for remission maintenance in nephrotic syndrome. Pediatr Nephrol 23:2013-2020
34. Gellermann J, Weber L, Pape L, Tönshoff B, Hoyer P, Querfeld U (2013) Mycophenolate mofetil versus cyclosporin A in children with frequently relapsing nephrotic syndrome. J Am Soc Nephrol 24:1689-1697
35. Sinha A, Puraswani M, Kalaivani M, Goyal P, Hari P, Bagga A (2019) Efficacy and safety of mycophenolate mofetil versus levamisole in frequently relapsing nephrotic syndrome: an open-label randomized controlled trial. Kidney Int 95:210-218

36. Abeyagunawardena A, Trompeter R (2006) Efficacy of levamisole as a single agent in maintaining remission in steroid dependant nephrotic syndrome. Pediatr Nephrol 21:1503
37. Al-Saran K, Mirza K, Al-Ghanam G, Abdelkarim M (2006) Experience with levamisole in frequently relapsing, steroid-dependent nephrotic syndrome. Pediatr Nephrol 21:201-205
38. Gruppen MP, Bouts AH, Jansen-van der Weide MC, Merkus MP, Zurowska A, Maternik M, Massella L, Emma F, Niaudet P, Cornelissen EAM, Schurmans T, Raes A, van de Walle J, van Dyck M, Gulati A, Bagga A, Davin JC (2018) A randomized clinical trial indicates that levamisole increases the time to relapse in children with steroid-sensitive idiopathic nephrotic syndrome. Kidney Int 93:510-518
39. Rashid H, Ahmed S, Fatima N, Khanam A (1996) Levamisole in the treatment of steroid dependent or frequent relapsing nephrotic syndrome in children. Bangladesh Renal Journal 15:6-8
40. Sural S, Pahari D, Mitra K, Bhattacharya S, Mondal S, Taraphder A (2001) Efficacy of levamisole compared to cyclophosphamide and steroid in frequently relapsing (FR) minimal change nephrotic syndrome (MCNS). J Am Soc Nephrol 12:126A
41. Weiss R (1993) Randomized double-blind placebo controlled, multi-center trial of levamisole for children with frequently relapsing/steroid dependent nephrotic syndrome. J Am Soc Nephrol 4:289
42. Donia AF, Ammar HM, El-Agroudy Ael B, Moustafa Fel H, Sobh MA (2005) Long-term results of two unconventional agents in steroid-dependent nephrotic children. Pediatr Nephrol 20:1420-1425
43. Alatas H, Wirya IG, Tambunan T, Himawan S (1978) Controlled trial of chlorambucil in frequently relapsing nephrotic syndrome in children (a preliminary report). J Med Assoc Thai 61 Suppl 1:222-228
44. Barratt TM, Soothill JF (1970) Controlled trial of cyclophosphamide in steroid-sensitive relapsing nephrotic syndrome of childhood. Lancet 2:479-482
45. Chiu J, McLaine PN, Drummond KN (1973) A controlled prospective study of cyclophosphamide in relapsing, corticosteroid-responsive, minimal-lesion nephrotic syndrome in childhood. J Pediatr 82:607-613
46. Grupe WE, Makker SP, Ingelfinger JR (1976) Chlorambucil treatment of frequently relapsing nephrotic syndrome. N Engl J Med 295:746-749
47. (1974) Prospective, controlled trial of cyclophosphamide therapy in children with nephrotic syndrome. Report of the International study of Kidney Disease in Children. Lancet 2:423-427
48. Prasad N, Gulati S, Sharma RK, Singh U, Ahmed M (2004) Pulse cyclophosphamide therapy in steroid-dependent nephrotic syndrome. Pediatr Nephrol 19:494-498
49. Abeyagunawardena ATRS (2006) Intravenous pulsed vs oral Cyclophosphamide therapy in steroid-dependent nephrotic syndrome (abstract no: COD.PP 54). Pediatr Nephrol 2006;21(10):1535.
50. Nephrologie* AfP (1982) Effect of cytotoxic drugs in frequently relapsing nephrotic syndrome with and without steroid dependence. N Engl J Med 306:451-454
51. Abeyagunawardena A (2007) Intravenous pulsed cyclophophamide versus vincristine therapy in steroid dependant nephrotic syndrome: a randomised controlled trial. Pediatr Nephrol. pp 1547-1547
52. Barratt T, Cameron J, Chantler C, Ogg C, Soothill J (1973) Comparative trial of 2 weeks and 8 weeks cyclophosphamide in steroid-sensitive relapsing nephrotic syndrome of childhood. Arch Dis Child 48:286-290
53. Ueda N, Kuno K, Ito S (1990) Eight and 12 week courses of cyclophosphamide in nephrotic syndrome. Arch Dis Child 65:1147-1150
54. McCrory WW, Shibuya M, Lu WH, Lewy JE (1973) Therapeutic and toxic effects observed with different dosage programs of cyclophosphamide in treatment of steroid-responsive but frequently relapsing nephrotic syndrome. J Pediatr 82:614-618
55. Baluarte HJ, Hiner L, Gruskin AB (1978) Chlorambucil dosage in frequently relapsing nephrotic syndrome: a controlled clinical trial. J Pediatr 92:295-298
56. Yoshioka K, Ohashi Y, Sakai T, Ito H, Yoshikawa N, Nakamura H, Tanizawa T, Wada H, Maki S (2000) A multicenter trial of mizoribine compared with placebo in children with frequently relapsing nephrotic syndrome. Kidney Int 58:317-324
57. Zhang B, Liu T, Wang W, Zhang X, Fan S, Liu Z, Liu Z, Wu X (2014) A prospective randomly controlled clinical trial on azithromycin therapy for induction treatment of children with nephrotic syndrome. Eur J Pediatr 173:509-515

58. Abramowicz M, Barnett HL, Edelmann CM, Jr., Greifer I, Kobayashi O, Arneil GC, Barron BA, Gordillo PG, Hallman N, Tiddens HA (1970) Controlled trial of azathioprine in children with nephrotic syndrome. A report for the international study of kidney disease in children. Lancet 1:959-961
59. Wang C-s, Travers C, McCracken C, Leong T, Gbadegesin R, Quiroga A, Benfield MR, Hidalgo G, Srivastava T, Lo M, Yadin O, Mathias R, Araya CE, Khalid M, Orjuela A, Zaritsky J, Al-Akash S, Kamel M, Greenbaum LA (2018) Adrenocorticotropic Hormone for Childhood Nephrotic Syndrome. Clin J Am Soc Nephrol 13:1859-1865
60. Cerkauskiene R, Kaltenis P (2005) Comparative study of prednisolone alone and prednisolone plus fusidic acid in the treatment of children with steroid-responsive nephrotic syndrome. Medicina 41 Suppl 1:26-30
61. Uddin GM RM, Rahman MH, Roy RR, Begum A, Huque SS. (2016) Comparative efficacy of mycophenolate mofetil and cyclosporine in children with frequent relapse nephrotic syndrome [abstract]. Pediatr Nephrol 31:1852-1853
62. Kemper MJ, Kuwertz-Broeking E, Bulla M, Mueller-Wiefel DE, Neuhaus TJ (2004) Recurrence of severe steroid dependency in cyclosporin A-treated childhood idiopathic nephrotic syndrome. Nephrol Dial Transplant 19:1136-1141
63. Iyengar A, Karthik S, Kumar A, Biswas S, Phadke K (2006) Cyclosporine in steroid dependent and resistant childhood nephrotic syndrome. Indian pediatrics 43:14-19
64. El-Husseini A, El-Basuony F, Mahmoud I, Sheashaa H, Sabry A, Hassan R, Taha N, Hassan N, Sayed-Ahmad N, Sobh M (2005) Long-term effects of cyclosporine in children with idiopathic nephrotic syndrome: a single-centre experience. Nephrol Dial Nephrol 20:2433-2438
65. Rinaldi S, Sesto A, Barsotti P, Faraggiana T, Sera F, Rizzoni G (2005) Cyclosporine therapy monitored with abbreviated area under curve in nephrotic syndrome. Pediatr Nephrol 20:25-29
66. Mahmoud I, Basuni F, Sabry A, El-Husseini A, Hassan N, Ahmad NS, Elbaz M, Moustafa F, Sobh M (2005) Single-centre experience with cyclosporin in 106 children with idiopathic focal segmental glomerulosclerosis. Nephrol Dial Transplant 20:735-742
67. Wasilewska A, Zoch-Zwierz W, Tomaszewska B, Tenderenda E (2005) [The effect of cyclosporine A in steroid-dependent nephrotic syndrome in children]. Polski merkuriusz lekarski : organ Polskiego Towarzystwa Lekarskiego 18:168-172
68. El-Husseini A, El-Basuony F, Mahmoud I, Donia A, Sheashaa H, Sabry A, Hassan N, Sayed-Ahmad N, Sobh M (2006) Impact of the cyclosporine-ketoconazole interaction in children with steroid-dependent idiopathic nephrotic syndrome. Eur J Clin Pharmacol 62:3-8
69. Sinha MD, MacLeod R, Rigby E, Clark AGB (2006) Treatment of severe steroid-dependent nephrotic syndrome (SDNS) in children with tacrolimus. Nephrol Dial Nephrol 21:1848-1854
70. Sheashaa H, Mahmoud I, El-Basuony F, El-Husseini A, Hassan N, El-Baz M, Ahmed NS, Sobh M (2007) Does cyclosporine achieve a real advantage for treatment of idiopathic nephrotic syndrome in children? A long-term efficacy and safety study. Int Urol Nephrol 39:923-928
71. Kranz B, Vester U, Büscher R, Wingen AM, Hoyer PF (2008) Cyclosporine-A-induced nephrotoxicity in children with minimal-change nephrotic syndrome: long-term treatment up to 10 years. Pediatr Nephrol 23:581-586
72. Kengne-Wafo S, Massella L, Diomedi-Camassei F, Gianviti A, Vivarelli M, Greco M, Stringini GR, Emma F (2009) Risk factors for cyclosporin A nephrotoxicity in children with steroid-dependant nephrotic syndrome. Clin J Am Soc Nephrol 4:1409-1416
73. Leroy V, Baudouin V, Alberti C, Guest G, Niaudet P, Loirat C, Deschenes G, Czernichow P, Simon D (2009) Growth in boys with idiopathic nephrotic syndrome on long-term cyclosporin and steroid treatment. Pediatr Nephrol 24:2393-2400
74. Suzuki K, Oki E, Tsuruga K, Aizawa-Yashiro T, Ito E, Tanaka H (2010) Benefits of once-daily administration of cyclosporine a for children with steroid-dependent, relapsing nephrotic syndrome. Tohoku J Exp Med 220:183-186
75. Ishikura K, Yoshikawa N, Hattori S, Sasaki S, Iijima K, Nakanishi K, Matsuyama T, Yata N, Ando T, Honda M (2010) Treatment with microemulsified cyclosporine in children with frequently relapsing nephrotic syndrome. Nephrol Dial Nephrol 25:3956-3962
76. Ishikura K, Yoshikawa N, Nakazato H, Sasaki S, Iijima K, Nakanishi K, Matsuyama T, Ito S, Yata N, Ando T, Honda M (2012) Two-Year Follow-Up of a Prospective Clinical Trial of Cyclosporine for Frequently Relapsing Nephrotic Syndrome in Children. Clin J Am Soc Nephrol 7:1576-1583

77. Wang W, Xia Y, Mao J, Chen Y, Wang D, Shen H, Fu H, Du L, Liu A (2012) Treatment of tacrolimus or cyclosporine A in children with idiopathic nephrotic syndrome. Pediatr Nephrol 27:2073-2079
78. Supavekin S, Surapaitoolkorn W, Kurupong T, Chaiyapak T, Piyaphanee N, Pattaragarn A, Sumboonnanonda A (2013) Tacrolimus in steroid resistant and steroid dependent childhood nephrotic syndrome. J Med Assoc Thai 96:33-40
79. Bock ME, Cohn RA, Ali FN (2013) Treatment of childhood nephrotic syndrome with long-term, low-dose tacrolimus. Clin Nephrol 79:432-438
80. Hamasaki Y, Komaki F, Ishikura K, Hamada R, Sakai T, Hataya H, Ogata K, Ando T, Honda M (2017) Nephrotoxicity in children with frequently relapsing nephrotic syndrome receiving long-term cyclosporine treatment. Pediatr Nephrol 32:1383-1390
81. Kuroyanagi Y, Gotoh Y, Kasahara K, Nagano C, Fujita N, Yamakawa S, Yamamoto M, Takeda A, Uemura O (2018) Effectiveness and nephrotoxicity of a 2-year medium dose of cyclosporine in pediatric patients with steroid-dependent nephrotic syndrome: determination of the need for follow-up kidney biopsy. Clin Exp Nephrol 22:413-419
82. Yang EM, Lee ST, Choi HJ, Cho HY, Lee JH, Kang HG, Park YS, Cheong HI, Ha IS (2016) Tacrolimus for children with refractory nephrotic syndrome: a one-year prospective, multicenter, and open-label study of Tacrobell®, a generic formula. World J Pediatr 12:60-65
83. Delbet JD, Aoun B, Buob D, Degheili J, Brocheriou I, Ulinski T (2019) Infrequent tacrolimus-induced nephrotoxicity in French patients with steroid-dependent nephrotic syndrome. Pediatr Nephrol 34:2605-2608
84. Fujinaga S, Nishino T, Urushihara Y (2021) Efficacy of once-daily cyclosporine in Japanese children with steroid-dependent minimal change nephrotic syndrome. Clin Exp Nephrol 25:213-214
85. Wang J, Mao J, Chen J, Fu H, Shen H, Zhu X, Liu A, Shu Q, Du L (2016) Evaluation of mycophenolate mofetil or tacrolimus in children with steroid sensitive but frequently relapsing or steroid-dependent nephrotic syndrome. Nephrology (Carlton, Vic) 21:21-27
86. Fujinaga S, Someya T, Watanabe T, Ito A, Ohtomo Y, Shimizu T, Kaneko K (2013) Cyclosporine versus mycophenolate mofetil for maintenance of remission of steroid-dependent nephrotic syndrome after a single infusion of rituximab. Eur J Pediatr 172:513-518
87. Fujinaga S, Sakuraya K, Yamada A, Urushihara Y, Ohtomo Y, Shimizu T (2015) Positive role of rituximab in switching from cyclosporine to mycophenolate mofetil for children with high-dose steroid-dependent nephrotic syndrome. Pediatr Nephrol 30:687-691
88. Sumegi V, Haszon I, Bereczki C, Papp F, Turi S (2008) Long-term follow-up after cyclophosphamide and cyclosporine-A therapy in steroid-dependent and -resistant nephrotic syndrome. Pediatr Nephrol 23:1085-1092
89. Abeyagunawardena AS, Dillon MJ, Rees L, van't Hoff W, Trompeter RS (2003) The use of steroid-sparing agents in steroid-sensitive nephrotic syndrome. Pediatr Nephrol 18:919-924
90. Chen SY, Wu CY, Tsai IJ, Tsau YK (2010) Treatment course of steroid-dependent nephrotic syndrome: emphasized on treatment effect. Nephrology (Carlton, Vic) 15:336-339
91. Basu B, Babu BG, Mahapatra TK (2017) Long-term efficacy and safety of common steroid-sparing agents in idiopathic nephrotic children. Clin Exp Nephrol 21:143-151
92. Moorani KN, Hotchandani HM, Zubair AM, Lohana NC, Veerwani NR (2019) Immunosuppressive therapy in children with primary nephrotic syndrome: single center experience, Karachi, Pakistan. BMC Nephrol 20:239
93. Latta K, von Schnakenburg C, Ehrich JH (2001) A meta-analysis of cytotoxic treatment for frequently relapsing nephrotic syndrome in children. Pediatr Nephrol 16:271-282
94. Vester U, Kranz B, Zimmermann S, Hoyer PF (2003) Cyclophosphamide in steroid-sensitive nephrotic syndrome: outcome and outlook. Pediatr Nephrol 18:661-664
95. Kyrieleis HAC, Levtchenko EN, Wetzels JFM (2007) Long-Term Outcome After Cyclophosphamide Treatment in Children With Steroid-Dependent and Frequently Relapsing Minimal Change Nephrotic Syndrome. Am J Kidney Dis 49:592-597
96. Azib S, Macher MA, Kwon T, Dechartres A, Alberti C, Loirat C, Deschênes G, Baudouin V (2011) Cyclophosphamide in steroid-dependent nephrotic syndrome. Pediatr Nephrol 26:927-932
97. Zagury A, de Oliveira AL, de Moraes CA, de Araujo Montalvão JA, Novaes RH, de Sá VM, Monteiro de Carvalho Dde B, Matuck T (2011) Long-term follow-up after cyclophosphamide therapy in steroid-dependent nephrotic syndrome. Pediatr Nephrol 26:915-920
98. Cammas B, Harambat J, Bertholet-Thomas A, Bouissou F, Morin D, Guigonis V, Bendeddouche S, Afroukh-Hacini N, Cochat P, Llanas B, Decramer S, Ranchin B (2010)

Long-term effects of cyclophosphamide therapy in steroid-dependent or frequently relapsing idiopathic nephrotic syndrome. Nephrol Dial Nephrol 26:178-184
99. Bajeer IA, Khatri S, Tresa V, Hashmi S, Mubarak M, Lanewala AA (2018) Histopathological Spectrum and Short-Term Outcome of Treatment with Cyclophosphamide in Relapsing Steroid-Sensitive Nephrotic Syndrome. J Coll Physicians Surg Pak 28:436-439
100. Alsaran K, Grisaru S, Stephens D, Arbus G (2001) Levamisole vs. cyclophosphamide for frequently-relapsing steroid-dependent nephrotic syndrome. Clin Nephrol 56:289-294
101. Moustafa BH, Tolba OA (2016) Immunosuppressive therapy in children with steroid-resistant, frequently-relapsing, and steroid-dependent idiopathic nephrotic syndrome: a single center experience. Electronic Physician 8:2039-2047
102. Bagga A, Hari P, Moudgil A, Jordan SC (2003) Mycophenolate mofetil and prednisolone therapy in children with steroid-dependent nephrotic syndrome. Am J Kidney Dis 42:1114-1120
103. Novak I, Frank R, Vento S, Vergara M, Gauthier B, Trachtman H (2005) Efficacy of mycophenolate mofetil in pediatric patients with steroid-dependent nephrotic syndrome. Pediatr Nephrol 20:1265-1268
104. Mendizabal S, Zamora I, Berbel O, Sanahuja MJ, Fuentes J, Simon J (2005) Mycophenolate mofetil in steroid/cyclosporine-dependent/resistant nephrotic syndrome. Pediatr Nephrol 20:914-919
105. Hogg RJ, Fitzgibbons L, Bruick J, Bunke M, Ault B, Baqi N, Trachtman H, Swinford R (2006) Mycophenolate mofetil in children with frequently relapsing nephrotic syndrome: a report from the Southwest Pediatric Nephrology Study Group. Clin J Am Soc Nephrol 1:1173-1178
106. Afzal K, Bagga A, Menon S, Hari P, Jordan SC (2007) Treatment with mycophenolate mofetil and prednisolone for steroid-dependent nephrotic syndrome. Pediatr Nephrol 22:2059-2065
107. Fujinaga S, Ohtomo Y, Hirano D, Nishizaki N, Someya T, Ohtsuka Y, Kaneko K, Shimizu T (2009) Mycophenolate mofetil therapy for childhood-onset steroid dependent nephrotic syndrome after long-term cyclosporine: extended experience in a single center. Clin Nephrol 72:268-273
108. Baudouin V, Alberti C, Lapeyraque AL, Bensman A, André JL, Broux F, Cailliez M, Decramer S, Niaudet P, Deschênes G, Jacqz-Aigrain E, Loirat C (2012) Mycophenolate mofetil for steroid-dependent nephrotic syndrome: a phase II Bayesian trial. Pediatr Nephrol 27:389-396
109. Banerjee S, Pahari A, Sengupta J, Patnaik SK (2013) Outcome of severe steroid-dependent nephrotic syndrome treated with mycophenolate mofetil. Pediatr Nephrol 28:93-97
110. Dehoux L, Hogan J, Dossier C, Fila M, Niel O, Maisin A, Macher MA, Kwon T, Baudouin V, Deschênes G (2016) Mycophenolate mofetil in steroid-dependent idiopathic nephrotic syndrome. Pediatr Nephrol 31:2095-2101
111. Kapoor K, Saha A, Kaur M, Dubey NK, Upadhyay AD (2017) Mycophenolate Sodium for Children with Frequently Relapsing or Steroid Dependent Nephrotic Syndrome. Indian Pediatri 54:885-886
112. Nandi M, Mandal SK, Samanta M, Majhi A, Das MK (2019) Efficacy of Mycophenolate Mofetil as a Remission Maintaining Agent in Idiopathic Childhood Nephrotic Syndrome. Indian J Nephrol 29:34-41
113. Karunamoorthy S, Thanigachalam D, Jeyachandran D, Ramanathan S, Natarajan G, Thoppalan B (2020) The safety and efficacy of mycophenolate mofetil in children and adolescents with steroid-dependent nephrotic syndrome: a single-centre study. Clin Kidney J 13:179-183
114. Fu LS, Chi CS (2000) Levamisole in steroid-sensitive nephrotic syndrome children with steroid-dependency and/or frequent relapses. Acta Paediatr Taiwan 41:80-84
115. Alshaya HO, Kari JA (2002) Levamisole treatment in steroid sensitive nephrotic syndrome. Saudi Med J 23:1101-1104
116. Donia AF, Amer GM, Ahmed HA, Gazareen SH, Moustafa FE, Shoeib AA, Ismail AM, Khamis S, Sobh MA (2002) Levamisole: adjunctive therapy in steroid dependent minimal change nephrotic children. Pediatr Nephrol 17:355-358
117. Al-Ibrahim AA, Al-Kharraz SM, Al-Sadoon DM, Al-Madani AJ, Al-Musallam SA (2003) Levamisole Therapy as a Second-line Immunosuppressive Agent in Corticosteroid-sensitive Nephrotic Syndrome in Children. Saudi J Kidney Dis Transplant 14:153-157
118. Sümegi V, Haszon I, Iványi B, Bereczki C, Papp F, Túri S (2004) Long-term effects of levamisole treatment in childhood nephrotic syndrome. Pediatr Nephrol 19:1354-1360

119. Fu LS, Shien CY, Chi CS (2004) Levamisole in steroid-sensitive nephrotic syndrome children with frequent relapses and/or steroid dependency: comparison of daily and every-other-day usage. Nephron Clin Pract 97:c137-141
120. Hafeez F, Ahmed TM, Samina U (2006) Levamisole in steroid dependent and frequently relapsing nephrotic syndrome. J Coll Physicians Surg Pak 16:35-37
121. Boyer O, Moulder JK, Grandin L, Somers MJ (2008) Short- and long-term efficacy of levamisole as adjunctive therapy in childhood nephrotic syndrome. Pediatr Nephrol 23:575-580
122. Madani A, Isfahani ST, Rahimzadeh N, Fereshtehnejad SM, Hoseini R, Moghtaderi M, Mohseni P, Ataiee N (2010) Effect of levamisole in steroid-dependent nephrotic syndrome. Iran J Kidney Dis 4:292-296
123. Elmas AT, Tabel Y, Elmas ON (2013) Short- and long-term efficacy of levamisole in children with steroid-sensitive nephrotic syndrome. Int Urol Nephrol 45:1047-1055
124. Ekambaram S, Mahalingam V, Nageswaran P, Udani A, Geminiganesan S, Priyadarshini S (2014) Efficacy of levamisole in children with frequently relapsing and steroid-dependent nephrotic syndrome. Indian Pediatr 51:371-373
125. Kuźma-Mroczkowska E, Skrzypczyk P, Pańczyk-Tomaszewska M (2016) Levamisole therapy in children with frequently relapsing and steroid-dependent nephrotic syndrome: a single-center experience. Cent Eur J Immunol 41:243-247
126. Abeyagunawardena AS, Karunadasa U, Jayaweera H, Thalgahagoda S, Tennakoon S, Abeyagunawardena S (2017) Efficacy of higher-dose levamisole in maintaining remission in steroid-dependant nephrotic syndrome. Pediatr Nephrol 32:1363-1367
127. Kiruba Samuel EM, Krishnamurthy S, Bhanudeep S, Muske S (2017) Levamisole in Frequently-relapsing and Steroid-dependent Nephrotic Syndrome. Indian Pediatr 54:831-834
128. Moorani KN, Zubair AM, Veerwani NR, Hotchandani HJ (2020) Efficacy of Levamisole in children with Frequent Relapsing and Steroid Dependent Nephrotic Syndrome at Tertiary Care Center-Karachi. Pak J Med Sci 36:1193-1198
129. Alsaran K, Mirza K, Al-Talhi A, Al-Kanani E (2017) Experience with second line drugs in frequently relapsing and steroid dependent childhood nephrotic syndrome in a large Saudi center. Int Journal Pediatr Adolesc Med 4:66-70
130. Kamei K, Ito S, Nozu K, Fujinaga S, Nakayama M, Sako M, Saito M, Yoneko M, Iijima K (2009) Single dose of rituximab for refractory steroid-dependent nephrotic syndrome in children. Pediatr Nephrol 24:1321-1328
131. Gulati A, Sinha A, Jordan SC, Hari P, Dinda AK, Sharma S, Srivastava RN, Moudgil A, Bagga A (2010) Efficacy and safety of treatment with rituximab for difficult steroid-resistant and -dependent nephrotic syndrome: multicentric report. Clin J Am Soc Nephrol 5:2207-2212
132. Prytula A, Iijima K, Kamei K, Geary D, Gottlich E, Majeed A, Taylor M, Marks SD, Tuchman S, Camilla R, Ognjanovic M, Filler G, Smith G, Tullus K (2010) Rituximab in refractory nephrotic syndrome. Pediatr Nephrol 25:461-468
133. Sellier-Leclerc AL, Macher MA, Loirat C, Guérin V, Watier H, Peuchmaur M, Baudouin V, Deschênes G (2010) Rituximab efficiency in children with steroid-dependent nephrotic syndrome. Pediatr Nephrol 25:1109-1115
134. Kemper MJ, Gellermann J, Habbig S, Krmar RT, Dittrich K, Jungraithmayr T, Pape L, Patzer L, Billing H, Weber L, Pohl M, Rosenthal K, Rosahl A, Mueller-Wiefel DE, Dötsch J (2012) Long-term follow-up after rituximab for steroid-dependent idiopathic nephrotic syndrome. Nephrol Dial Transplant 27:1910-1915
135. Tellier S, Brochard K, Garnier A, Bandin F, Llanas B, Guigonis V, Cailliez M, Pietrement C, Dunand O, Nathanson S, Bertholet-Thomas A, Ichay L, Decramer S (2013) Long-term outcome of children treated with rituximab for idiopathic nephrotic syndrome. Pediatr Nephrol 28:911-918
136. Ruggenenti P, Ruggiero B, Cravedi P, Vivarelli M, Massella L, Marasà M, Chianca A, Rubis N, Ene-Iordache B, Rudnicki M, Pollastro RM, Capasso G, Pisani A, Pennesi M, Emma F, Remuzzi G (2014) Rituximab in steroid-dependent or frequently relapsing idiopathic nephrotic syndrome. J Am Soc Nephrol 25:850-863
137. Sun L, Xu H, Shen Q, Cao Q, Rao J, Liu HM, Fang XY, Zhou LJ (2014) Efficacy of rituximab therapy in children with refractory nephrotic syndrome: a prospective observational study in Shanghai. World J Pediatr 10:59-63
138. Kamei K, Ogura M, Sato M, Sako M, Iijima K, Ito S (2016) Risk factors for relapse and long-term outcome in steroid-dependent nephrotic syndrome treated with rituximab. Pediatr Nephrol 31:89-95

139. Fujinaga S, Hirano D, Mizutani A, Sakuraya K, Yamada A, Sakurai S, Shimizu T (2017) Predictors of relapse and long-term outcome in children with steroid-dependent nephrotic syndrome after rituximab treatment. Clin Exp Nephrol 21:671-676
140. Topaloglu R, Gulhan B, Celegen K, Inozu M, Hayran M, Duzova A, Ozaltin F (2019) Rituximab for Children With Difficult-to-Treat Nephrotic Syndrome: Its Effects on Disease Progression and Growth. Front Pediatr 7:313
141. Hogan J, Dossier C, Kwon T, Macher MA, Maisin A, Couderc A, Niel O, Baudouin V, Deschênes G (2019) Effect of different rituximab regimens on B cell depletion and time to relapse in children with steroid-dependent nephrotic syndrome. Pediatr Nephrol 34:253-259
142. Maxted AP, Dalrymple RA, Chisholm D, McColl J, Tse Y, Christian MT, Reynolds BC (2019) Low-dose rituximab is no less effective for nephrotic syndrome measured by 12-month outcome. Pediatr Nephrol 34:855-863
143. Takahashi T, Okamoto T (2019) Periodically repeated rituximab administrations in children with refractory nephrotic syndrome: 2-year multicenter observational study 34:87-96
144. Chan EY, Webb H, Yu E, Ghiggeri GM, Kemper MJ, Ma AL, Yamamura T, Sinha A, Bagga A, Hogan J, Dossier C, Vivarelli M, Liu ID, Kamei K, Ishikura K, Saini P, Tullus K (2020) Both the rituximab dose and maintenance immunosuppression in steroid-dependent/frequently-relapsing nephrotic syndrome have important effects on outcomes. Kidney Int 97:393-401
145. Sinha A, Bagga A, Gulati A, Hari P (2012) Short-term efficacy of rituximab versus tacrolimus in steroid-dependent nephrotic syndrome. Pediatr Nephrol 27:235-241
146. Webb H, Jaureguiberry G, Dufek S, Tullus K, Bockenhauer D (2016) Cyclophosphamide and rituximab in frequently relapsing/steroid-dependent nephrotic syndrome. Pediatr Nephrol 31:589-594
147. Kari JA, Alhasan KA, Albanna AS, Safdar OY, Shalaby MA, Böckenhauer D, El-Desoky SM (2020) Rituximab versus cyclophosphamide as first steroid-sparing agent in childhood frequently relapsing and steroid-dependent nephrotic syndrome. Pediatr Nephrol 35:1445-1453
148. Delbe-Bertin L, Aoun B, Tudorache E, Lapillone H, Ulinski T (2013) Does rituximab induce hypogammaglobulinemia in patients with pediatric idiopathic nephrotic syndrome? Pediatr Nephrol 28:447-451
149. Delbet JD, Leclerc G, Ulinski T (2019) Idiopathic nephrotic syndrome and rituximab: may we predict circulating B lymphocytes recovery? Pediatr Nephrol 34:529-532
150. Colucci M, Carsetti R, Cascioli S, Casiraghi F, Perna A, Ravà L, Ruggiero B, Emma F, Vivarelli M (2016) B Cell Reconstitution after Rituximab Treatment in Idiopathic Nephrotic Syndrome. J Am Soc Nephrol 27:1811-1822
151. Colucci M, Carsetti R, Serafinelli J, Rocca S, Massella L, Gargiulo A, Lo Russo A, Capponi C, Cotugno N, Porzio O, Onetti Muda A, Palma P, Emma F, Vivarelli M (2019) Prolonged Impairment of Immunological Memory After Anti-CD20 Treatment in Pediatric Idiopathic Nephrotic Syndrome. Front Immunol 10:1653
152. Parmentier C, Delbet JD, Decramer S, Boyer O, Hogan J, Ulinski T (2020) Immunoglobulin serum levels in rituximab-treated patients with steroid-dependent nephrotic syndrome. Pediatr Nephrol 35:455-462
153. Fujinaga S, Ozawa K, Sakuraya K, Yamada A, Shimizu T (2016) Late-onset adverse events after a single dose of rituximab in children with complicated steroid-dependent nephrotic syndrome. Clin Nephrol 85:340-345
154. Maeda R, Kawasaki Y, Ohara S, Suyama K, Hosoya M (2018) Serum sickness with refractory nephrotic syndrome following treatment with rituximab. CEN C Rep 7:69-72
155. Ohtomo Y, Fujinaga S, Takada M, Murakami H, Akashi S, Shimizu T, Kaneko K, Yamashiro Y (2005) High-dose mizoribine therapy for childhood-onset frequently relapsing steroid-dependent nephrotic syndrome with cyclosporin nephrotoxicity. Pediatr Nephrol 20:1744-1749
156. Kawasaki Y, Hosoya M, Kobayashi S, Ohara S, Onishi N, Takahashi A, Isome M, Suzuki H (2005) Oral mizoribine pulse therapy for patients with steroid-resistant and frequently relapsing steroid-dependent nephrotic syndrome. Nephrol Dial Transplant 20:2243-2247
157. Fujinaga S, Hirano D, Nishizaki N, Someya T, Ohtomo Y, Ohtsuka Y, Shimizu T, Kaneko K (2011) Single daily high-dose mizoribine therapy for children with steroid-dependent nephrotic syndrome prior to cyclosporine administration. Pediatr Nephrol 26:479-483
158. Xia ZK, Gao YF, Rong LP, Dang XQ, Shen Q, Jiang XY, Yi ZW, Xu H (2019) Usefulness of mizoribine administration in children with frequently relapsing nephrotic syndrome, and the relationship between pharmacokinetic parameters and efficacy: a multicenter prospective cohort study in China. World J Pediatr 15:262-269.

159. Mizutani A, Fujinaga S (2019) Positive effects of single-daily high-dose mizoribine therapy after cyclophosphamide in young children with steroid-dependent nephrotic syndrome Clin Exp Nephrol 23:244-250
160. Kondoh T, Ikezumi Y (2019) Assessment of factors associated with mizoribine responsiveness in children with steroid-dependent nephrotic syndrome Clin Exp Nephrol 23:1154-1160
161. Kausman JY, Yin L, Jones CL, Johnstone L, Powell HR (2005) Vincristine treatment in steroid-dependent nephrotic syndrome. Pediatr Nephrol 20:1416-1419
162. Krishnan RG, Coulthard MG, Moghal NE (2006) Is there a role for vincristine in nephrotic syndrome? Pediatr Nephrol 21:597
163. Coppo R, Camilla R, Porcellini MG, Peruzzi L, Gianoglio B, Amore A, Dapra V, Loiacono E, Fonsato V, Dal Canton A, Esposito C, Esposito P, Tovo PA (2012) Saquinavir in steroid-dependent and -resistant nephrotic syndrome: a pilot study. Nephrol Dial Transplant 27:1902-1910
164. Chakraborty R, Mehta A, Nair N, Nemer L, Jain R, Joshi H, Raina R (2020) ACTH Treatment for Management of Nephrotic Syndrome: A Systematic Review and Reappraisal. Int J Nephrol 2020:2597079
165. Lentz RD, Bergstein J, Steffes MW, Brown DR, Prem K, Michael AF, Vernier RL (1977) Postpubertal evaluation of gonadal function following cyclophosphamide therapy before and during puberty. J Pediatr 91:385-394
166. Pennisi AJ, Grushkin CM, Lieberman E (1975) Gonadal function in children with nephrosis treated with cyclophosphamide. Am J Dis Child129:315-318
167. Kirkland RT, Bongiovanni AM, Cornfield D, McCormick JB, Parks JS, Tenore A (1976) Gonadotropin responses to luteinizing releasing factor in boys treated with cyclophosphamide for nephrotic syndrome. J Pediatr 89:941-944
168. Penso J, Lippe B, Ehrlich R, Smith FG, Jr. (1974) Testicular function in prepubertal and pubertal male patients treated with cyclophosphamide for nephrotic syndrome. J Pediatr 84:831-836
169. Trompeter RS, Lloyd BW, Hicks J, White RH, Cameron JS (1985) Long-term outcome for children with minimal-change nephrotic syndrome. Lancet 1:368-370
170. Lewis MA, Baildom EM, Davies N, Houston IB, Postlethwaite RJ (1988) Steroid-sensitive minimal change nephrotic syndrome. Long-term follow-up. Contrib Nephrol 67:226-228
171. Takeichi S, Tazawa M, Morooka M, Minowa S, Yasaki T (1997) [Long-term follow-up study of children with minimal change nephrotic syndrome]. Nihon Jinzo Gakkai Shi 39:155-160
172. Fakhouri F, Bocquet N, Taupin P, Presne C, Gagnadoux MF, Landais P, Lesavre P, Chauveau D, Knebelmann B, Broyer M, Grünfeld JP, Niaudet P (2003) Steroid-sensitive nephrotic syndrome: from childhood to adulthood. Am J Kidney Dis 41:550-557
173. Rüth EM, Kemper MJ, Leumann EP, Laube GF, Neuhaus TJ (2005) Children with steroid-sensitive nephrotic syndrome come of age: long-term outcome. J Pediatr 147:202-207
174. Skrzypczyk P, Panczyk-Tomaszewska M, Roszkowska-Blaim M, Wawer Z, Bienias B, Zajgzkowska M, Kilis-Pstrusinska K, Jakubowska A, Szczepaniak M, Pawlak-Bratkowska M, Tkaczyk M (2014) Long-term outcomes in idiopathic nephrotic syndrome: from childhood to adulthood. Clin Nephrol 81:166-173
175. Korsgaard T, Andersen RF, Joshi S, Hagstrøm S, Rittig S (2019) Childhood onset steroid-sensitive nephrotic syndrome continues into adulthood. Pediatr Nephrol 34:641-648
176. Aydin M, Franke I, Kurylowicz L, Ganschow R, Lentze M, Born M, Hagemann R (2019) The long-term outcome of childhood nephrotic syndrome in Germany: a cross-sectional study. Clin Exp Nephrol 23:676-68.
177. Carter SA, Mistry S, Fitzpatrick J, Banh T, Hebert D, Langlois V, Pearl RJ, Chanchlani R, Licht CPB, Radhakrishnan S, Brooke J, Reddon M, Levin L, Aitken-Menezes K, Noone D, Parekh RS (2020) Prediction of Short- and Long-Term Outcomes in Childhood Nephrotic Syndrome. Kidney Int Rep 5:426-434
178. Watson AR, Harden P, Ferris M, Kerr PG, Mahan J, Ramzy MF (2011) Transition from pediatric to adult renal services: a consensus statement by the International Society of Nephrology (ISN) and the International Pediatric Nephrology Association (IPNA). Pediatr Nephrol 26:1753-1757.
